# Lenvatinib starting doses below 20 mg versus 20 mg with pembrolizumab in advanced or recurrent endometrial cancer: a systematic review and meta-analysis of comparative observational evidence

**DOI:** 10.3389/fonc.2026.1965694

**Published:** 2026-10-16

**Authors:** Yu Wu, Juncheng Wei, Chuyue Chang, Mengfei Li, Runbing Xu, Shi Dong, Miao Jiang

**Affiliations:** 1 Dongzhimen Hospital, Beijing University of Chinese Medicine, Beijing, China; 2 School of Life Science, Beijing University of Chinese Medicine, Beijing, China

**Keywords:** dose reduction, endometrial cancer, lenvatinib, meta-analysis, pembrolizumab, starting dose

## Abstract

**Background:**

Lenvatinib plus pembrolizumab is an established treatment for patients with previously treated advanced or recurrent endometrial cancer. In routine practice, clinicians may select a lenvatinib starting dose below 20 mg for patients expected to have limited tolerance, but the comparative effectiveness and tolerability of that strategy remain uncertain.

**Methods:**

We searched PubMed/MEDLINE, Web of Science Core Collection, and Cochrane CENTRAL from inception to 11 July 2026, with source-specific updates completed on 13–14 July 2026. Comparative observational studies of adults receiving lenvatinib plus pembrolizumab were eligible. The primary estimand compared starting doses below 20 mg with 20 mg; a 10-mg threshold contrast and first-8-week relative dose intensity were kept separate. Binary outcomes were summarized as risk ratios (RRs) and time-to-event outcomes as hazard ratios (HRs). Risk of bias was assessed with ROBINS-I, missing-evidence bias with ROB-ME, and certainty with GRADE.

**Results:**

Ten studies representing 10 independent cohorts and 1,000 patients were included. Five cohorts (703 patients in total) informed the primary starting-dose comparison, with outcome-specific analysis denominators. For starting doses below 20 mg versus 20 mg, the pooled RR was 0.97 for objective response (95% confidence interval [CI], 0.70–1.34; I²=11.9%) and 0.96 for disease control (95% CI, 0.83–1.11; I²=0%). The RR for subsequent lenvatinib dose reduction was 0.66 (95% CI, 0.34–1.27; I²=27.4%). Estimates for interruption, permanent discontinuation, grade 3 or higher adverse events, and progression-free survival were imprecise; hospitalization and treatment persistence were not poolable. Every evaluated result had serious overall risk of bias, and certainty was very low for all pooled comparisons.

**Conclusion:**

Comparative observational evidence does not establish that starting lenvatinib below 20 mg preserves antitumor effectiveness or improves tolerability relative to a 20 mg starting dose. Prospective, confounder-aware comparative studies are needed before a reduced starting-dose strategy can be recommended broadly.

**Systematic review registration**

https://www.crd.york.ac.uk/PROSPERO/, identifier CRD420261458417.

## 1 Introduction

According to global estimates for 2022, endometrial cancer was the sixth most commonly diagnosed cancer in women worldwide, with an estimated 420,242 new cases and 97,704 deaths (1). Approximately 10%–15% of patients present with advanced disease (2), and 5-year survival for distant-stage disease is about 17% (3). Although treatment options have expanded, outcomes after recurrence or progression remain poor, and treatment decisions must account for molecular subtype, prior therapy, comorbidity, and treatment tolerance (4).

Lenvatinib is a multitargeted tyrosine kinase inhibitor with antiangiogenic and microenvironment-modulating activity. In combination with the programmed cell death-1 inhibitor pembrolizumab, it is an established option for previously treated advanced or recurrent endometrial cancer (5–9). In Study 309/KEYNOTE-775, the combination prolonged progression-free and overall survival relative to physician’s-choice chemotherapy; lenvatinib was started at 20 mg once daily, with protocol-directed dose modification as needed (8–10). In the first-line ENGOT-en9/LEAP-001 trial, however, the combination did not meet the prespecified progression-free-survival or overall-survival criteria versus chemotherapy in mismatch repair-proficient disease, underscoring the importance of treatment line and molecular context (11).

However, treatment-related toxicities frequently limit the ability to maintain therapy. Hypertension, gastrointestinal adverse events, fatigue, and other toxicities frequently require interruption or dose reduction (10, 12). In practice, clinicians may start lenvatinib below 20 mg in older or frail patients, in those with relevant comorbidities, or when tolerance is a concern. This comparison is difficult to interpret because starting-dose selection is not randomized and is related to baseline prognosis, whereas later dose reduction and early relative dose intensity are postbaseline exposures.

Previous reviews have described the overall efficacy and safety of lenvatinib plus pembrolizumab and the broader systemic-treatment landscape in recurrent or advanced endometrial cancer (12, 13), but they did not resolve whether starting below 20 mg reduces toxicity or treatment disruption without materially changing antitumor outcomes. We therefore reviewed comparative observational evidence and performed outcome-specific meta-analyses where the data were compatible. The primary estimand compared starting doses below 20 mg with 20 mg. A 10-mg threshold contrast and first-8-week relative dose intensity were evaluated separately because they represent different clinical questions.

## 2 Methods

This systematic review and meta-analysis was prospectively registered in PROSPERO (No. CRD420261458417) and conducted in accordance with PRISMA 2020 (14). The completed PRISMA and PRISMA-S checklists are provided in **Supplementary Informations A1** and **A2**, respectively.

### 2.1 Data sources and search strategy

We searched PubMed/MEDLINE, Web of Science Core Collection, and Cochrane CENTRAL from inception to 11 July 2026. Controlled vocabulary, where available, and text words covered endometrial or uterine cancer, lenvatinib, and pembrolizumab. Dose, outcome, relative-dose-intensity, and study-design terms were not required because these details may appear only in full text or tables. No date, study-design, or publication-type filter was applied at the database level; English-language eligibility was applied during screening. PROSPERO, ClinicalTrials.gov, meeting websites, reference lists, and forward citations were used as supplementary duplicate-risk and grey-literature sources. PubMed was rerun on 13 July 2026; Web of Science and CENTRAL updates were completed on 14 July 2026. No update changed the eligible evidence set. Full strategies and source-specific update records are reported in **Supplementary Information A3**.

### 2.2 Eligibility criteria

Eligible studies included adults with advanced or recurrent endometrial carcinoma treated with lenvatinib plus pembrolizumab and reported an eligible comparative exposure contrast with at least one prespecified effectiveness, tolerability, or treatment-persistence outcome. The primary contrast was a baseline lenvatinib starting dose below 20 mg versus 20 mg once daily. Outcomes included progression-free survival, overall survival, objective response, disease control, grade 3 or higher adverse events, subsequent lenvatinib dose reduction, treatment interruption, permanent discontinuation, hospitalization, and treatment persistence. Outcome definitions and ascertainment windows are summarized in **Supplementary Information C1**.

Patients remained classified by baseline starting dose regardless of later dose modification. Response was accepted according to the source definition, commonly RECIST-based (15), and adverse-event grades were mapped to the reported CTCAE version (16). Studies evaluating only dose reduction after treatment initiation and single-arm studies reporting only overall rates were excluded from the primary comparative estimand. Reports with an eligible contrast but no compatible effect estimate were retained for structured narrative synthesis. Comparisons of 10 mg or less versus more than 10 mg and first-8-week relative-dose-intensity analyses were retained as separate threshold-sensitivity and supplementary estimands.

### 2.3 Study selection and data extraction

Two reviewers independently screened titles and abstracts, assessed the retrieved full texts, and extracted study and outcome data. They also independently verified the mapping of linked reports to studies and cohorts, every raw value used in a meta-analysis, and the key ROBINS-I and GRADE judgments. Disagreements were resolved by consensus after the relevant full report and **Supplementary Material** had been rechecked. Extracted items included baseline characteristics, exposure definitions, events and denominators, effect estimates and confidence intervals, adjustment covariates, reference groups, effect direction, and source location. Companion reports and overlapping cohorts supplied supporting information but were counted only once.

### 2.4 Risk of bias, missing evidence, and certainty

ROBINS-I 2016 was applied at the result level rather than as a single study wide score (17). For starting-dose comparisons, prespecified confounders included age, performance status, frailty or comorbidity, organ function, disease burden, histologic and molecular features, prior treatment, center and calendar period, and clinician dose-selection preference. Relative dose intensity analyses additionally considered early toxicity, early response, treatment interruption, survivor selection, immortal-time risk, reverse causality, and time-varying confounding. Risk of bias due to missing evidence was assessed for each quantitative synthesis with ROB-ME (18). Certainty was rated by outcome using GRADE for nonrandomized evidence (19). Two reviewers independently reviewed key ROBINS-I and GRADE judgments and resolved disagreements by consensus.

### 2.5 Estimands and exposure framework

The three exposure contrasts were specified using a target-trial framework to clarify eligibility, treatment strategies, time zero, follow-up, effect of interest, and critical confounders. For both starting-dose estimands, time zero was combination-treatment initiation and the effect of interest was assignment to the baseline starting-dose strategy. First-8-week relative dose intensity was treated as a postbaseline exposure. A day-56 landmark analysis would begin follow-up at day 56 among patients alive and free of the outcome event, addressing a conditional survivor population; it would not recover effects on earlier events or remove confounding. A causal analysis of dynamic dosing strategies would instead require explicit dosing rules and appropriate longitudinal methods. The available aggregate studies were not described as target-trial emulations. Specifications and study-level confounding summaries are provided in **Supplementary Informations C2** and **C3**.

### 2.6 Statistical analysis

Analyses were performed in R version 4.3.2 (20) with the metafor package (21). Random-effects models summarized RRs for binary outcomes and HRs for time-to-event outcomes, with 95% CIs. Between-study variance was estimated by restricted maximum likelihood and Hartung–Knapp adjustment was used for interval estimation (22). Prespecified fixed-effect models were supporting analyses only. Binary syntheses used arm-level counts. We did not identify an extractable adjusted comparative RR or OR for an eligible binary contrast in the available full reports and retrieved **Supplementary Materials**. Effects were oriented as the reduced-dose exposure versus the higher-dose reference; for adverse outcomes, an estimate below 1 favored the reduced-dose exposure. Effect provenance and causal interpretation boundaries are detailed in **Supplementary Information C4**, and processing and direction harmonization in **Supplementary Information C5**.

Adjusted HRs and reported unadjusted HRs were analyzed in separate evidence layers. No HR was reconstructed from a Kaplan–Meier curve. Statistical heterogeneity was described with Cochran’s Q, I², and τ² (23), and prediction intervals were reported when estimable. Shared controls and multiple dose groups were handled through prespecified dependency clusters so that each cohort contributed no more than one effect to a given synthesis. Results that could not be pooled were summarized in accordance with SWiM, without vote counting by statistical significance (24).

We did not perform subgroup analyses or meta-regression because every quantitative synthesis contained fewer than 10 independent cohorts and compatible subgroup-specific effects were unavailable. Age, ECOG performance status, prior treatment, treatment line, histology, and mismatch-repair or molecular status were summarized descriptively. The absence of a pooled interaction should not be interpreted as evidence that these factors did not modify the observed associations.

## 3 Results

### 3.1 Study selection

The three database searches identified 487 records. After 181 duplicates were removed, 306 unique records were screened and 282 were excluded on title and abstract. Of the 24 remaining reports, eight were identified as duplicate or companion reports before retrieval. Sixteen reports from database searching and one additional report from citation chasing were retrieved and assessed in full. Ten independent studies were included; seven reports were excluded or resolved as nonindependent at full-text review (**Supplementary Information C6**). **Figure 1** shows the selection process. The evidence base comprised 10 studies, 10 independent cohorts, 14 linked reports, and 1,000 patients (25–34). Eligibility decisions for selected clinically relevant reports are provided in **Supplementary Table C13**.

**Figure 1:**
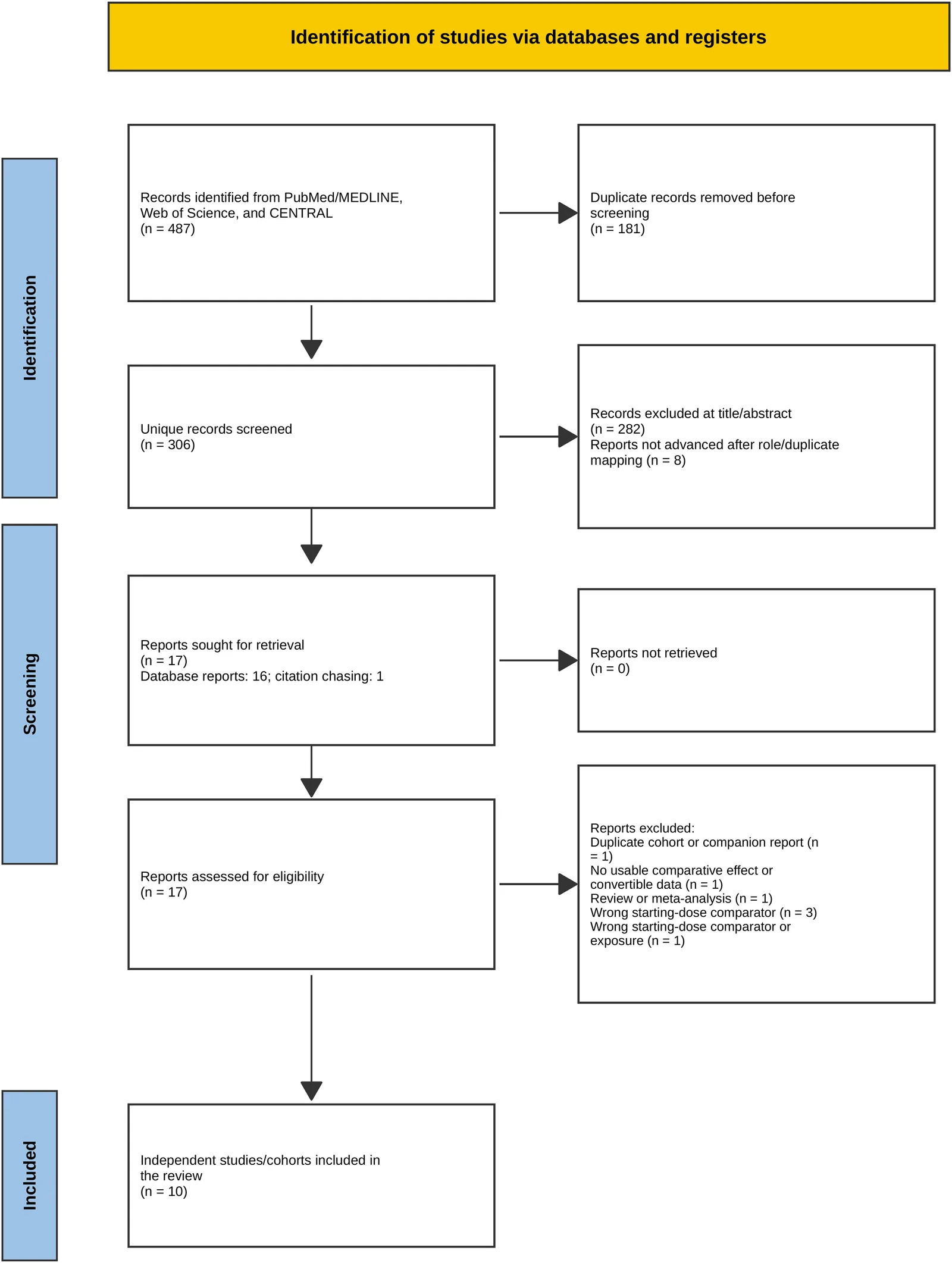
PRISMA 2020 flow diagram. Records identified through PubMed/MEDLINE, Web of Science Core Collection, and Cochrane CENTRAL are shown separately from citation chasing. Report counts are not treated as independent-study or independent-cohort counts.

### 3.2 Study and exposure characteristics

All included studies were retrospective observational cohorts conducted in the United States or Japan, with accrual periods spanning 2019–2025. Five cohorts directly informed the primary starting-dose comparison and included 703 patients in total (25–29); outcome-specific analysis denominators excluded ineligible arms and reflected response evaluability. Two cohorts informed the separate 10-mg threshold estimand (30, 31). Two independent Japanese cohorts reported first-8-week relative-dose-intensity evidence using noninterchangeable 50% and 56% thresholds (32, 34), and one small cohort contributed only non-8-week relative-dose-intensity evidence (33). Study roles are listed in **Supplementary Information C7**. **Table 1** summarizes study and exposure characteristics; participant and baseline characteristics are detailed in **Supplementary Information C8**.

**Table 1:** Included study and exposure characteristics.

Study	Country/region	Design	Setting	Period	N	Evidence role
How 2021 (25)	United States	retrospective observational cohort	MD Anderson Cancer Center; single academic center	2019–09 to 2020–10	70	Primary starting-dose contrast (<20 vs 20 mg)
Zammarrelli 2023 (26)	United States	retrospective observational cohort	Memorial Sloan Kettering Cancer Center; single academic center	2019–10–01 to 2021–11–30	43	Primary starting-dose contrast (<20 vs 20 mg)
Ta 2025 (27)	United States	retrospective observational cohort	Kaiser Permanente Northern and Southern California	2019–09–01 to 2023–06–30	369	Primary starting-dose contrast (<20 vs 20 mg)
Corr 2025 (28)	United States	multicenter retrospective observational cohort	ECMT2 national consortium; 14 institutions	2019–03 to 2022–12	188	Primary starting-dose contrast (<20 vs 20 mg; multiarm)
Boku 2026 (29)	Japan	retrospective observational cohort	Kansai Medical University Hospital; single center	2022–02 to 2025–08	33	Primary starting-dose contrast (<20 vs 20 mg)
Barbi 2025 (30)	United States	retrospective observational cohort	Northwell Cancer Institute; real-world cohort	2019 to 2022	92	Threshold sensitivity (≤10 vs >10 mg)
Dzienny 2026 (31)	United States	retrospective observational cohort	Yale; single academic tertiary center	2019–03 to 2023–02	59	Threshold sensitivity (≤10 vs >10 mg)
Nagase 2025 (32)	Japan	multicenter observational cohort	Gynecologic Oncology Group of Osaka; 15 institutions	2022–01 to 2024–02	105	First-8-week RDI (50% cutoff; supplementary)
Tochigi 2024 (33)	Japan	retrospective observational cohort	Tohoku University Hospital; single center	treatment initiated by 2022–12; follow-up through 2023–07	15	Non-8-week RDI (narrative)
Takahashi 2026 (34)	Japan	retrospective observational cohort	Jichi Medical University Hospital; single center	2021–12 to 2025–08	26	First-8-week RDI (56% cutoff; narrative)

RDI, relative dose intensity.

Across the five primary-comparison cohorts, the below-20-mg category did not represent a single reduced dose: reported daily starting doses included 10, 12, 14, 16, and 18 mg (25–29). Ta 2025 also classified one patient each receiving 4 and 8 mg and two receiving alternating 10/20 mg within its 154-patient reduced-dose group (27). That source-defined group was retained, because separate outcomes for these four patients were unavailable. Its distinct 20-mg five-days-per-week arm (11 patients) was retained only for descriptive accounting and excluded from the primary comparison, all quantitative syntheses, and shared-control calculations. Most cohorts treated patients after prior systemic therapy; where reported, cohort medians ranged from one to three prior regimens. Reporting of age, ECOG performance status, comorbidity, organ function, histology, mismatch-repair or molecular status, and the rationale for dose selection was incomplete. Compatible subgroup-specific effects were unavailable.

### 3.3 Objective response and disease control

Five independent cohorts informed the main objective-response analysis. The random-effects RR was 0.97 (95% CI, 0.70–1.34; I²=11.9%; prediction interval, 0.62–1.52) (**Figure 2**). The total-treatment-arm denominator sensitivity analysis was similar (RR, 0.97; 95% CI, 0.70–1.34; I²=11.4%) (**Supplementary Information C9**; **Supplementary Figure B1**). Five cohorts informed disease control (RR, 0.96; 95% CI, 0.83–1.11; I²=0%) (**Supplementary Figure B2**). These estimates do not establish equivalence, noninferiority, or preserved antitumor effectiveness. **Table 2** summarizes the quantitative syntheses.

**Figure 2:**
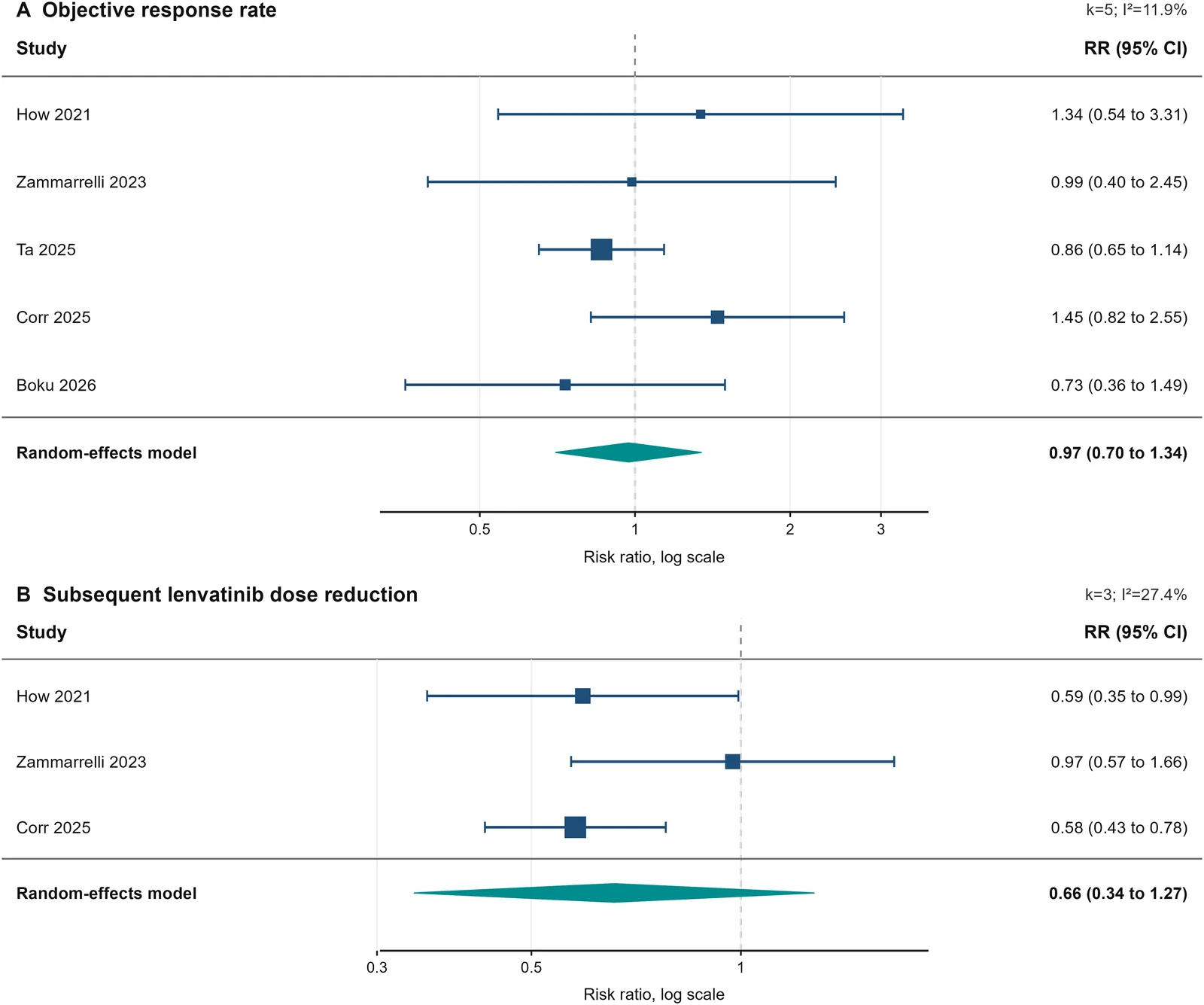
Meta-analyses of objective response rate **(A)** and subsequent lenvatinib dose reduction **(B)**. Effects are oriented as a starting dose below 20 mg versus 20 mg. Squares represent study-specific estimates, horizontal lines show 95% confidence intervals, and teal diamonds represent random-effects pooled estimates. Models used restricted maximum likelihood with Hartung–Knapp confidence intervals. The dashed vertical line marks 1.00. Confidence intervals crossing 1 do not establish equivalence, noninferiority, or preserved efficacy.

**Table 2:** Quantitative synthesis results and interpretation boundaries.

Outcome and role	k	Effect	Random-effects estimate (95% CI)	I²	Interpretation boundary
Objective response; main	5	RR	0.97 (0.70–1.34)	11.9%	Association only
Subsequent lenvatinib dose reduction; main	3	RR	0.66 (0.34–1.27)	27.4%	Association only
Disease control; supplementary	5	RR	0.96 (0.83–1.11)	0%	Association only
Progression-free survival; exploratory	2	HR	See **Supplementary Tables C9** and **C12**	See **Supplementary Table C9**	Quantitatively uninformative
Grade 3 or higher adverse events; exploratory	2	RR	1.13 (0.47–2.72)	0%	Sparse evidence
Interruption; supplementary	2	RR	0.89 (0.16–5.16)	14.4%	Sparse evidence
Combination-treatment toxicity discontinuation; exploratory	2	RR	0.78 (0.20–3.01)	0%	Sparse evidence
Objective response, total-arm denominator; sensitivity	5	RR	0.97 (0.70–1.34)	11.4%	Denominator sensitivity
Any-toxicity discontinuation, 10-mg threshold; sensitivity	2	RR	1.04 (0.66–1.66)	0%	Separate estimand

Random-effects restricted-maximum-likelihood models with Hartung–Knapp intervals are authoritative. Null-crossing intervals do not establish equivalence, noninferiority, or preserved efficacy.

### 3.4 Dose modification, interruption, and discontinuation

Three cohorts informed subsequent lenvatinib dose reduction after treatment initiation. The random-effects RR was 0.66 (95% CI, 0.34–1.27; I²=27.4%) (**Figure 2**). The prespecified fixed-effect supporting estimate was 0.64 (95% CI, 0.51–0.81), but the random-effects Hartung–Knapp result remained the primary estimate. Leave-one-out estimates ranged from 0.58 to 0.76, with a maximum I² of 63.9% (**Supplementary Information C10**).

For treatment interruption, the pooled RR was 0.89 (95% CI, 0.16–5.16; two cohorts) (**Supplementary Figure B3**). For permanent discontinuation of the combination because of toxicity or intolerance, the exploratory RR was 0.78 (95% CI, 0.20–3.01; two cohorts) (**Supplementary Figure B4**). In the separate 10-mg threshold analysis, any-toxicity discontinuation yielded an RR of 1.04 (95% CI, 0.66–1.66; two cohorts) (**Supplementary Figure B5**). All intervals remained compatible with clinically important effects in either direction.

### 3.5 Survival, adverse events, hospitalization, and persistence

Two cohorts reported unadjusted progression-free-survival HRs. The prespecified exploratory calculation is retained in **Supplementary Figure B6** and **Supplementary Tables C9** and **C12**. Its confidence interval spans more than four orders of magnitude, making the pooled estimate quantitatively uninformative; interpretation rests on the study-level estimates and their limitations. Adjusted progression-free-survival and overall-survival estimates were available from single cohorts and were not combined with the unadjusted estimates. No HR was reconstructed from a Kaplan–Meier curve.

Two cohorts informed grade 3 or higher adverse events (RR, 1.13; 95% CI, 0.47–2.72) (**Supplementary Figure B7**). Hospitalization estimates used incompatible observation windows and were not pooled, and no compatible comparative effect was available for treatment persistence. These outcomes were summarized narratively in accordance with SWiM; statistical significance was not used to classify study findings.

### 3.6 Relative-dose-intensity evidence

Nagase 2025 compared a first-8-week relative dose intensity of at least 50% with less than 50% and reported an adjusted progression-free-survival HR of 0.53 (95% CI, 0.31–0.91) (32). Takahashi 2026 included 26 patients, all of whom started lenvatinib at 20 mg, and therefore provided no starting-dose comparison (34). Relative dose intensity was calculated as the cumulative dose received during the first 56 days divided by the planned cumulative dose of 20 mg/day for 56 days. A 56% threshold was derived by receiver operating characteristic analysis within the same cohort (area under the curve, 0.55). Among 18 response-evaluable patients, objective response occurred in 4 of 11 patients with relative dose intensity of at least 56% and 1 of 7 below 56% (RR, 2.55; 95% CI, 0.35–18.36). The adjusted progression-free-survival HR for less than 56% versus at least 56% was 0.67 (95% CI, 0.19–2.45) after adjustment for histology, platinum-free interval, and relative dose intensity. The reported unadjusted HR was 0.96 (95% CI, 0.30–3.05), but its direction could not be verified because **Supplementary Figure S3B** was unavailable. Because the 56% threshold was outcome-derived and differed from the prespecified 50% contrast, these results were summarized narratively and not pooled. Both first-8-week analyses were vulnerable to early-treatment selection, survivor and immortal-time bias, reverse causality, and time-varying confounding. Tochigi 2024 reported 4-week and whole-course relative-dose-intensity associations (33), which could not be converted to the first-8-week estimand.

### 3.7 Risk of bias, missing evidence, and certainty

Fifty-five result-level ROBINS-I assessments were completed. All received an overall judgment of serious risk of bias; none was judged critical. The main concerns were confounding by indication, incomplete adjustment for baseline fitness and disease burden, and selective availability of outcome-specific models. Relative-dose-intensity analyses had additional risks because exposure was determined after treatment began and could be affected by early toxicity or response, treatment interruption, survivor selection, immortal time, reverse causality, and time-varying confounding. The Takahashi model was further limited by 16 progression-free-survival events for three covariates and by use of a threshold derived from the same cohort.

ROB-ME yielded a judgment of some concerns for each of the nine quantitative syntheses. Outcomes and analytic models were not consistently prespecified in the observational reports, and the small number of studies prevented meaningful pattern-based assessment (**Supplementary Information C11**). Formal ROB-ME assessment was not applicable to the two Takahashi outcomes that were summarized narratively. The direction of any bias from missing evidence could not be predicted. **Figure 3** shows the result-level ROBINS-I judgments. Certainty was very low for every pooled comparison (**Supplementary Information C12**).

**Figure 3:**
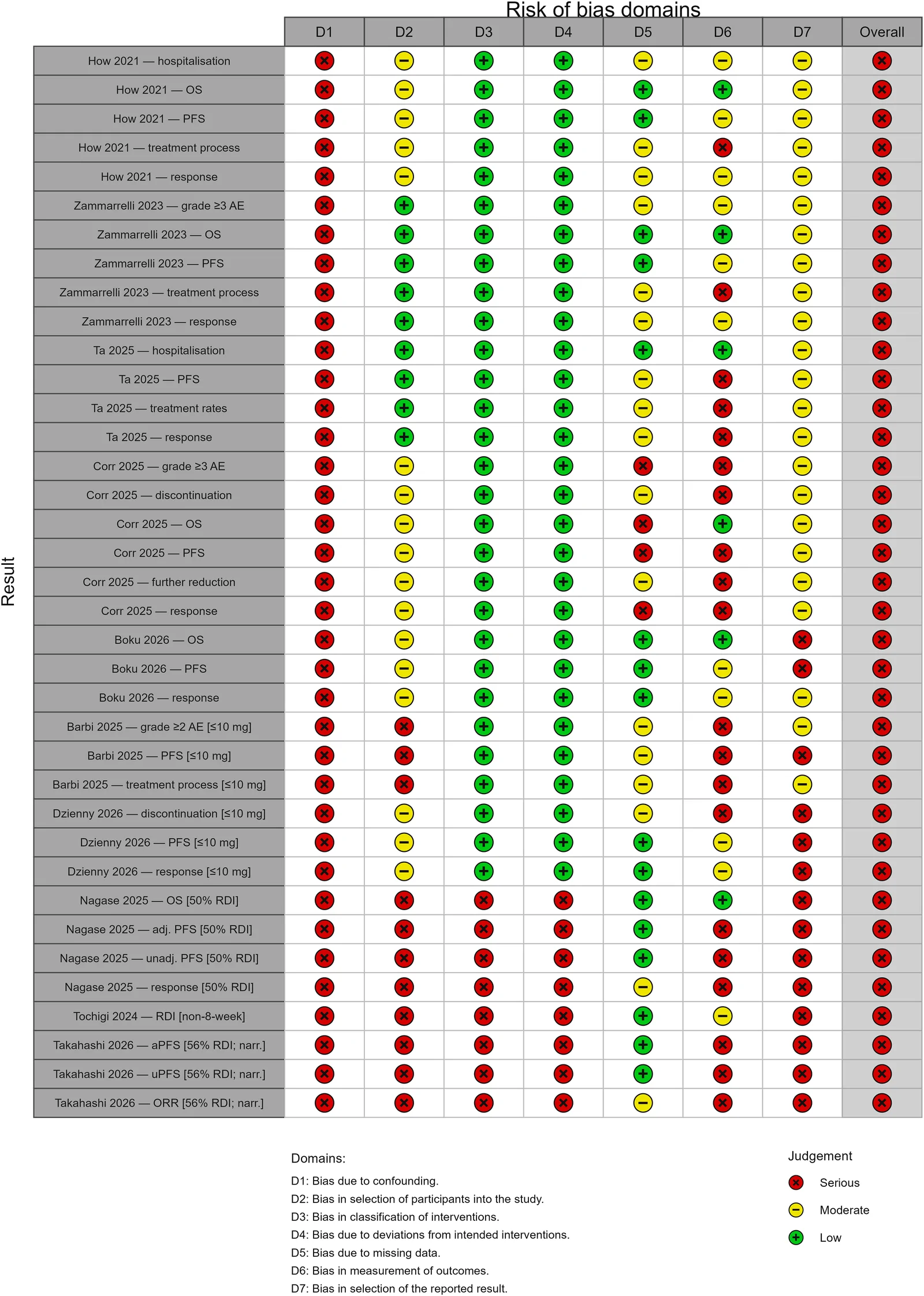
ROBINS-I 2016 traffic-light display at the result level. Each row represents a result-level assessment grouped by estimand. D1, confounding; D2, selection of participants; D3, classification of interventions; D4, deviations from intended interventions; D5, missing data; D6, measurement of outcomes; D7, selection of the reported result. Green plus signs indicate low risk, yellow minus signs moderate risk, and red crosses serious risk. Companion reports supplied supporting information but did not add independent cohorts.

## 4 Discussion

This review examined comparative real-world evidence for alternative lenvatinib starting doses rather than the overall activity of lenvatinib plus pembrolizumab covered in earlier reviews (12, 13). Ten independent observational cohorts met the eligibility criteria, but only five directly compared a starting dose below 20 mg with 20 mg. Pooled estimates for objective response and disease control were close to 1, while the estimate for subsequent dose reduction favored the lower starting-dose group but crossed 1 under the prespecified random-effects model. Evidence for survival, grade 3 or higher adverse events, interruption, permanent discontinuation, hospitalization, and treatment persistence was sparse, imprecise, or incompatible. The results do not show that a lower starting dose retains antitumor effectiveness or offers superior tolerability.

The pivotal trials established the effectiveness of lenvatinib plus pembrolizumab using a 20-mg starting dose with protocol-directed dose management in previously treated disease (7–10). The contrasting first-line findings from LEAP-001 reinforce that treatment setting and mismatch-repair status are clinically important (11). Real-world starting-dose selection addresses a different question. Clinicians may choose lower doses for patients with worse performance status, greater comorbidity, organ dysfunction, or anticipated toxicity, and those same factors may influence response, survival, and treatment duration (25–31). Conversely, patients given 20 mg may be fitter and more able to remain on therapy. The pooled RRs therefore estimate associations between observed prescribing strategies and outcomes, not the causal effect of assigning a reduced starting dose. No extractable adjusted comparative RR or OR for an eligible binary contrast was identified in the available source corpus, and adjustment in the few multivariable survival models remained incomplete. Residual confounding could therefore move estimates in either direction.

Objective response was the most stable comparative synthesis: measured heterogeneity was low, and the total-arm denominator sensitivity analysis produced a similar estimate. Nevertheless, both the confidence interval and prediction interval remained compatible with clinically important benefit and harm. For subsequent dose reduction, the supporting fixed-effect interval excluded 1, whereas the prespecified random-effects Hartung–Knapp interval did not. Interpretation therefore rests on the prespecified random-effects model. The two-study syntheses of interruption, discontinuation, and grade 3 or higher adverse events had wide intervals and remain exploratory. The exploratory PFS summary was also quantitatively uninformative because of substantial heterogeneity and an exceptionally wide confidence interval.

The exposure definitions are not interchangeable. Starting below 20 mg is a baseline prescribing strategy, subsequent dose reduction is an event during treatment, and first-8-week relative dose intensity is a postbaseline measure shaped by early toxicity, progression, and interruption (32–34). We therefore kept the 10-mg threshold comparison as a separate sensitivity estimand. Takahashi’s same-cohort 56% threshold was likewise summarized narratively rather than combined with the prespecified 50% relative-dose-intensity contrast. This separation preserves the clinical meaning of each comparison but leaves little compatible evidence for survival and treatment persistence. The retained source-defined reduced-dose groups also contained uncommon schedules, so these aggregate contrasts cannot isolate a specific daily dose or a dose-response relationship.

The limitations arise primarily from the evidence. All cohorts were retrospective, treatment allocation was not randomized, and adjustment for clinician dose-selection preference, frailty, organ function, disease burden, treatment line, histology, and molecular features was incomplete (25–34). Outcome definitions and follow-up windows varied, several syntheses included only two studies, and formal small-study-effect assessment was not informative. Classification by baseline starting dose addressed the prespecified estimand but did not capture subsequent dose modification or cumulative exposure. Relative dose intensity was defined after treatment initiation and was vulnerable to early toxicity, early progression, survivor selection, immortal-time bias, reverse causality, and time-varying confounding. In Takahashi 2026 (34), the 56% cutoff was selected within a small cohort, the adjusted model was sparse, and the unavailable supplementary figure left the direction of the reported unadjusted HR unresolved. The available aggregate data could not test whether associations differed by age, ECOG performance status, treatment line, histologic subtype, or molecular subgroup; the findings therefore should not be extrapolated to a specific subgroup or an unstudied treatment setting. English-language eligibility may also have excluded relevant reports.

These data may inform hypotheses and patient-specific discussions, but they do not define a universal reduced starting dose. Future comparative studies should document the reason for dose selection and measure baseline fitness, organ function, disease burden, histologic and molecular features, prior therapy, treatment setting, and early toxicity. They should also report treatment interruption, persistence, patient-reported outcomes, and cumulative dose intensity over prespecified intervals.

With individual participant data, baseline-dose comparisons could use propensity-score matching or weighting to balance measured pretreatment covariates. Stabilized inverse-probability weights target the overall eligible population but can remain extreme under poor overlap (35). Matching weights and overlap weights are bounded, downweight patients with extreme propensity scores, and can improve stability and efficiency while targeting populations with greater treatment overlap (36, 37). The target population should therefore be stated before choosing a method. Covariate balance, propensity-score overlap, weight distributions, effective sample size, and sensitivity to prespecified weight truncation should be reported. None of these methods resolves unmeasured confounding or absent common support.

For multiple starting-dose levels, generalized propensity scores could support coherent multi-arm comparisons and estimation of a dose-response function, provided consistency, positivity, and weak unconfoundedness hold: for each dose, its potential outcome must be independent of dose assignment conditional on measured pretreatment covariates (38). Adequate overlap is required across the dose levels being compared. The aggregate reports did not provide compatible dose-specific effects or their shared-control covariance, so such models were not fitted here.

For survival outcomes, overlap-weighted restricted mean survival time would express the mean survival-time difference up to a prespecified, clinically meaningful horizon in the population with treatment overlap (39, 40). This patient-centered contrast does not require proportional hazards and can improve efficiency under appropriate conditions. It still requires adequate support through the chosen horizon and appropriate handling of censoring, for example inverse-probability-of-censoring weights, in addition to confounding control.

For first-8-week relative dose intensity, a day-56 landmark would classify accumulated exposure and start subsequent follow-up among patients alive and event-free at day 56. It avoids assigning that future exposure to earlier person-time but changes the population and does not by itself address survivor selection, reverse causality, or treatment-confounder feedback. A target trial of dynamic dosing would align eligibility, strategy assignment, and time zero at initiation; explicitly define dosing and deviation rules; and use longitudinal g-methods, such as sequentially weighted marginal structural models, when toxicity both affects later dosing and is affected by earlier treatment (41). These approaches require patient-level longitudinal data and assumptions about sequential exchangeability, positivity, consistency, and censoring that cannot be established from the available aggregate reports.

## 5 Conclusion

Comparative real-world evidence for starting lenvatinib below 20 mg versus 20 mg with pembrolizumab is limited and at serious risk of bias. Pooled associations for objective response, disease control, subsequent dose reduction, and other tolerability outcomes were imprecise and remained compatible with clinically important benefit and harm. These data do not support an equivalence or noninferiority conclusion. Prospective, confounder-aware comparative evaluation is needed before a reduced starting-dose strategy can be recommended broadly.

## Data Availability

The original contributions presented in the study are included in the article/**Supplementary Material**. Further inquiries can be directed to the corresponding authors.
