# Supplementary material for: Lenvatinib starting doses below 20 mg versus 20 mg with pembrolizumab in advanced or recurrent endometrial cancer: a systematic review and meta-analysis of comparative observational evidence
Source: Front Oncol. 2026 Oct 16;16:1965694. doi: 10.3389/fonc.2026.1965694 (PMC13719303; doi:10.3389/fonc.2026.1965694)
Supplement: Supplementary file 1 [file DataSheet1.pdf]

Supplementary Information (SI)

Article title: Lenvatinib starting doses below 20 mg versus 20 mg with pembrolizumab in advanced or recurrent endometrial cancer: a systematic review and meta-analysis of comparative observational evidence

Author names: Yu Wu, Juncheng Wei, Chuyue Chang, Mengfei Li, Runbing Xu, Shi Dong, Miao Jiang

This file includes:

- Supplementary notes A1–A3
- Supplementary figures B1–B7
- Supplementary tables C1–C13

1 Supplementary notes

Supplementary Information A1. PRISMA 2020 checklist

Section/topic	Item #	Official checklist item	Location(s) reported
**TITLE**			
Title	1	Identify the report as a systematic review.	Title
**ABSTRACT**			
Abstract	2	See the PRISMA 2020 for Abstracts checklist.	Abstract
**INTRODUCTION**			
Rationale	3	Describe the rationale for the review in the context of existing knowledge.	Introduction
Objectives	4	Provide an explicit statement of the objective(s) or question(s) the review addresses.	Introduction, objective
**METHODS**			
Eligibility criteria	5	Specify the inclusion and exclusion criteria for the review and how studies were grouped for the syntheses.	Methods, eligibility criteria
Information sources	6	Specify all databases, registers, websites, organisations, reference lists and other sources searched or consulted to identify studies. Specify the date when each source was last searched or consulted.	Methods, Data sources and search strategy
Search strategy	7	Present the full search strategies for all databases, registers and websites, including any filters and limits used.	Methods and Supplementary Methods, search strategy
Selection process	8	Specify the methods used to decide whether a study met the inclusion criteria of the review, including how many reviewers screened each record and each report retrieved, whether they worked independently, and if applicable, details of automation tools used in the process.	Methods, study selection
Data collection process	9	Specify the methods used to collect data from reports, including how many reviewers collected data from each report, whether they worked independently, any processes for obtaining or confirming data from study investigators, and if applicable, details of automation tools used in the process.	Methods, data collection
Data items	10a	List and define all outcomes for which data were sought. Specify whether all results that were compatible with each outcome domain in each study were sought (e.g. for all measures, time points, analyses), and if not, the methods used to decide which results to collect.	Methods, outcomes and data items
Data items	10b	List and define all other variables for which data were sought (e.g. participant and intervention characteristics, funding sources). Describe any assumptions made about any missing or unclear information.	Methods, outcomes and data items
Study risk of bias assessment	11	Specify the methods used to assess risk of bias in the included studies, including details of the tool(s) used, how many reviewers assessed each study and whether they worked independently, and if applicable, details of automation tools used in the process.	Methods, ROBINS-I
Effect measures	12	Specify for each outcome the effect measure(s) (e.g. risk ratio, mean difference) used in the synthesis or presentation of results.	Methods, effect measures
Synthesis methods	13a	Describe the processes used to decide which studies were eligible for each synthesis (e.g. tabulating the study intervention characteristics and comparing against the planned groups for each synthesis (item #5)).	Methods, statistical synthesis
Synthesis methods	13b	Describe any methods required to prepare the data for presentation or synthesis, such as handling of missing summary statistics, or data conversions.	Methods, statistical synthesis
Synthesis methods	13c	Describe any methods used to tabulate or visually display results of individual studies and syntheses.	Methods, statistical synthesis
Synthesis methods	13d	Describe any methods used to synthesize results and provide a rationale for the choice(s). If meta-analysis was performed, describe the model(s), method(s) to identify the presence and extent of statistical heterogeneity, and software package(s) used.	Methods, statistical synthesis
Synthesis methods	13e	Describe any methods used to explore possible causes of heterogeneity among study results (e.g. subgroup analysis, meta-regression).	Methods, statistical synthesis
Synthesis methods	13f	Describe any sensitivity analyses conducted to assess robustness of the synthesized results.	Methods, statistical synthesis
Reporting bias assessment	14	Describe any methods used to assess risk of bias due to missing results in a synthesis (arising from reporting biases).	Methods, ROB-ME
Certainty assessment	15	Describe any methods used to assess certainty (or confidence) in the body of evidence for an outcome.	Methods, GRADE
**RESULTS**			
Study selection	16a	Describe the results of the search and selection process, from the number of records identified in the search to the number of studies included in the review, ideally using a flow diagram.	Results, search and study selection; Figure 1
Study selection	16b	Cite studies that might appear to meet the inclusion criteria, but which were excluded, and explain why they were excluded.	Results, search and study selection; Figure 1

Section/topic	Item #	Official checklist item	Location(s) reported
Study characteristics	17	Cite each included study and present its characteristics.	Results, study characteristics; Table 1
Risk of bias in studies	18	Present assessments of risk of bias for each included study.	Results, risk of bias; Figure 3
Results of individual studies	19	For all outcomes, present, for each study: (a) summary statistics for each group (where appropriate) and (b) an effect estimate and its precision (e.g. confidence/credible interval), ideally using structured tables or plots.	Results and supplementary study-level results
Results of syntheses	20a	For each synthesis, briefly summarise the characteristics and risk of bias among contributing studies.	Results, quantitative syntheses and sensitivity analyses
Results of syntheses	20b	Present results of all statistical syntheses conducted. If meta-analysis was done, present for each the summary estimate and its precision (e.g. confidence/credible interval) and measures of statistical heterogeneity. If comparing groups, describe the direction of the effect.	Results, quantitative syntheses and sensitivity analyses
Results of syntheses	20c	Present results of all investigations of possible causes of heterogeneity among study results.	Results, quantitative syntheses and sensitivity analyses
Results of syntheses	20d	Present results of all sensitivity analyses conducted to assess the robustness of the synthesized results.	Results, quantitative syntheses and sensitivity analyses
Reporting biases	21	Present assessments of risk of bias due to missing results (arising from reporting biases) for each synthesis assessed.	Results, ROB-ME
Certainty of evidence	22	Present assessments of certainty (or confidence) in the body of evidence for each outcome assessed.	Results and Supplement, certainty
**DISCUSSION**			
Discussion	23a	Provide a general interpretation of the results in the context of other evidence.	Discussion
Discussion	23b	Discuss any limitations of the evidence included in the review.	Discussion
Discussion	23c	Discuss any limitations of the review processes used.	Discussion
Discussion	23d	Discuss implications of the results for practice, policy, and future research.	Discussion
**OTHER INFORMATION**			
Registration and protocol	24a	Provide registration information for the review, including register name and registration number, or state that the review was not registered.	Methods, registration/protocol; Title Page
Registration and protocol	24b	Indicate where the review protocol can be accessed, or state that a protocol was not prepared.	Methods, registration/protocol
Registration and protocol	24c	Describe and explain any amendments to information provided at registration or in the protocol.	Methods, registration/protocol; Title Page
Support	25	Describe sources of financial or non-financial support for the review, and the role of the funders or sponsors in the review.	Title Page, funding
Competing interests	26	Declare any competing interests of review authors.	Title Page,Conflict of Interest
Availability of data, code and other materials	27	Report which of the following are publicly available and where they can be found: template data collection forms; data extracted from included studies; data used for all analyses; analytic code; any other materials used in the review.	Data and Code Availability

Checklist items are reproduced from the official PRISMA 2020 checklist; reported locations refer to the manuscript and this Supplementary Information

Supplementary Information A2. PRISMA-S checklist

Section/topic	Item #	Official checklist item	Location(s) reported
**INFORMATION SOURCES AND METHODS**			
Database name	1	Name each individual database searched, stating the platform for each.	Methods, information sources
Multi-database searching	2	If databases were searched simultaneously on a single platform, state the name of the platform, listing all of the databases searched.	Methods, databases searched separately
Study registries	3	List any study registries searched.	Supplementary Methods, registry checks
Online resources and browsing	4	Describe any online or print source purposefully searched or browsed (e.g., tables of contents, print conference proceedings, web sites), and how this was done.	Supplementary Methods, grey-literature sources
Citation searching	5	Indicate whether cited references or citing references were examined, and describe any methods used for locating cited/citing references (e.g., browsing reference lists, using a citation index, setting up email alerts for references citing included studies).	Methods, citation chasing
Contacts	6	Indicate whether additional studies or data were sought by contacting authors, experts, manufacturers, or others.	Supplementary Methods, no author contact
Other methods	7	Describe any additional information sources or search methods used.	Supplementary Methods, companion-report checks
**SEARCH STRATEGIES**			
Full search strategies	8	Include the search strategies for each database and information source, copied and pasted exactly as run.	Supplementary Methods; Table S1
Limits and restrictions	9	Specify that no limits were used, or describe any limits or restrictions applied to a search (e.g., date or time period, language, study design) and provide justification for their use.	Methods, limits and English eligibility
Search filters	10	Indicate whether published search filters were used (as originally designed or modified), and if so, cite the filter(s) used.	Supplementary Methods, no filter
Prior work	11	Indicate when search strategies from other literature reviews were adapted or reused for a substantive part or all of the search, citing the previous review(s).	Methods, search construction
Updates	12	Report the methods used to update the search(es) (e.g., rerunning searches, email alerts).	Source-specific update searches: PubMed rerun July 13, 2026 (0 new unique records); Web of Science and CENTRAL updates completed July 14, 2026 with no change to the eligible evidence set
Dates of searches	13	For each search strategy, provide the date when the last search occurred.	Initial searches: all three databases July 11, 2026; PubMed update July 13, 2026; Web of Science and CENTRAL updates July 14, 2026
**PEER REVIEW**			
Peer review	14	Describe any search peer review process.	Methods, PRESS-informed verification
**MANAGING RECORDS**			
Total Records	15	Document the total number of records identified from each database and other information sources.	Results, 181/231/75
Deduplication	16	Describe the processes and any software used to deduplicate records from multiple database searches and other information sources.	Results/Figure 1, 487 to 306
**PRISMA-S: An Extension to the PRISMA Statement for Reporting Literature Searches in Systematic Reviews**			
**Rethlefsen ML, Kirtley S, Waffenschmidt S, Ayala AP, Moher D, Page MJ, Koffel JB, PRISMA-S Group.**			
**Last updated February 27, 2020.**			

Checklist items are reproduced from the official PRISMA-S checklist; reported locations refer to the manuscript and this Supplementary Information.

Supplementary Information A3. Database search strategies

Database	Platform	Date searched	Coverage dates	One-line search string	Structured search blocks	Limits	Records retrieved	Search record	Notes
PubMed/MEDLINE	NCBI PubMed	2026–07–11; update 2026–07–13	Database inception to 2026–07–11 for the initial search; source-specific update completed through 2026–07–13	("Endometrial Neoplasms"[Mesh] OR "endometrial cancer"[tiab] OR "endometrial cancers"[tiab] OR "endometrial carcinoma"[tiab] OR "endometrial carcinomas"[tiab] OR "endometrium cancer"[tiab] OR "uterine cancer"[tiab] OR "uterine cancers"[tiab] OR "uterine carcinoma"[tiab] OR "uterine carcinomas"[tiab] OR "uterine carcinosarcoma"[tiab] OR "uterine carcinosarcomas"[tiab] OR "malignant mixed mullerian tumor"[tiab] OR "malignant mixed mullerian tumors"[tiab] OR "malignant mixed müllerian tumor"[tiab] OR "malignant mixed müllerian tumors"[tiab]) AND ("lenvatinib"[Supplementary Concept] OR lenvatinib[tiab] OR Lenvima[tiab] OR E7080[tiab] OR "E-7080"[tiab]) AND ("pembrolizumab"[Supplementary Concept] OR pembrolizumab[tiab] OR pembro*[tiab] OR Keytruda[tiab] OR "MK-3475"[tiab] OR MK3475[tiab] OR lambrolizumab[tiab])	"Endometrial Neoplasms"[Mesh] OR "endometrial cancer"[tiab] OR "endometrial cancers"[tiab] OR "endometrial carcinoma"[tiab] OR "endometrial carcinomas"[tiab] OR "endometrium cancer"[tiab] OR "uterine cancer"[tiab] OR "uterine cancers"[tiab] OR "uterine carcinoma"[tiab] OR "uterine carcinomas"[tiab] OR "uterine carcinosarcoma"[tiab] OR "uterine carcinosarcomas"[tiab] OR "malignant mixed mullerian tumor"[tiab] OR "malignant mixed mullerian tumors"[tiab] OR "malignant mixed müllerian tumor"[tiab] OR "malignant mixed müllerian tumors"[tiab] | "lenvatinib"[Supplementary Concept] OR lenvatinib[tiab] OR Lenvima[tiab] OR E7080[tiab] OR "E-7080"[tiab] | "pembrolizumab"[Supplementary Concept] OR pembrolizumab[tiab] OR pembro*[tiab] OR Keytruda[tiab] OR "MK-3475"[tiab] OR MK3475[tiab] OR lambrolizumab[tiab] | #1 AND #2 AND #3	No language, date, study-design or publication-type filter at database-search stage	181	PubMed/MEDLINE export, 11 July 2026	Dose/outcome terms were intentionally not mandatory to preserve sensitivity; English eligibility was applied during screening; retrieval counts refer to the initial search. The update search did not change the eligible evidence set; an update-specific count was not available and was not inferred.
Web of Science	Clarivate Web of Science Core Collection	2026–07–11; update 2026–07–14	Database inception to 2026–07–11 for the initial search; source-specific update completed through 2026–07–14	TS=(("endometrial cancer*" OR "endometrial carcinoma*" OR "endometrium cancer*" OR "uterine cancer*" OR "uterine carcinoma*" OR "uterine carcinosarcoma*" OR "malignant mixed mullerian tumor*" OR "malignant mixed müllerian tumor*") AND (lenvatinib OR Lenvima OR E7080 OR "E-7080" OR "E 7080") AND (pembrolizumab OR pembro* OR Keytruda OR "MK-3475" OR MK3475 OR lambrolizumab))	TS=("endometrial cancer*" OR "endometrial carcinoma*" OR "endometrium cancer*" OR "uterine cancer*" OR "uterine carcinoma*" OR "uterine carcinosarcoma*" OR "malignant mixed mullerian tumor*" OR "malignant mixed müllerian tumor*") | TS=(lenvatinib OR Lenvima OR E7080 OR "E-7080" OR "E 7080") | TS=(pembrolizumab OR pembro* OR Keytruda OR "MK-3475" OR MK3475 OR lambrolizumab) | #1 AND #2 AND #3	No language, date, study-design or publication-type filter at database-search stage	231	Web of Science export, 11 July 2026	Dose/outcome terms were intentionally not mandatory to preserve sensitivity; English eligibility was applied during screening; retrieval counts refer to the initial search. The update search did not change the eligible evidence set; an update-specific count was not available and was not inferred.
Cochrane CENTRAL	Cochrane Library	2026–07–11; update 2026–07–14	Database inception to 2026–07–11 for the initial search; source-specific update completed	(((endometrial NEXT cancer*) OR (endometrial NEXT carcinoma*) OR (endometrial NEXT neoplasm*) OR (endometrial NEXT tumor*) OR	MeSH descriptor: [Endometrial Neoplasms] explode all trees | ((endometrial NEXT cancer*) OR (endometrial NEXT carcinoma*) OR (endometrial	No language, date, study-design or publication-type filter at database-search stage	75	Cochrane CENTRAL export, 11 July 2026	Dose/outcome terms were intentionally not mandatory to preserve sensitivity; English eligibility

Database	Platform	Date searched	Coverage dates	One-line search string	Structured search blocks	Limits	Records retrieved	Search record	Notes
			through 2026–07–14	(endometrial NEXT tumour*) OR (endometrium NEXT cancer*) OR (uterine NEXT cancer*) OR (uterine NEXT carcinoma*) OR (uterine NEXT carcinosarcoma*) OR (malignant NEXT mixed NEXT mullerian NEXT tumor*) OR (malignant NEXT mixed NEXT mullerian NEXT tumour*) OR (malignant NEXT mixed NEXT müllerian NEXT tumor*) OR (malignant NEXT mixed NEXT müllerian NEXT tumour*)):ti,ab,kw) AND ((lenvatinib OR lenvima OR e7080 OR "e-7080" OR (e NEXT 7080)):ti,ab,kw) AND ((pembrolizumab OR pembro* OR keytruda OR "mk-3475" OR mk3475 OR lambrolizumab):ti,ab,kw) in Trials	NEXT neoplasm*) OR (endometrial NEXT tumor*) OR (endometrial NEXT tumour*) OR (endometrium NEXT cancer*) OR (uterine NEXT cancer*) OR (uterine NEXT carcinoma*) OR (uterine NEXT carcinosarcoma*) OR (malignant NEXT mixed NEXT mullerian NEXT tumor*) OR (malignant NEXT mixed NEXT mullerian NEXT tumour*) OR (malignant NEXT mixed NEXT müllerian NEXT tumor*) OR (malignant NEXT mixed NEXT müllerian NEXT tumour*)):ti,ab,kw | #1 OR #2 | (lenvatinib OR lenvima OR e7080 OR "e-7080" OR (e NEXT 7080)):ti,ab,kw | (pembrolizumab OR pembro* OR keytruda OR "mk-3475" OR mk3475 OR lambrolizumab):ti,ab,kw | #3 AND #4 AND #5 | #6 in Trials				was applied during screening; retrieval counts refer to the initial search. The update search did not change the eligible evidence set; an update-specific count was not available and was not inferred.

Searches covered PubMed/MEDLINE, Web of Science Core Collection, and Cochrane CENTRAL. Source-specific search dates, update dates, strategies, and retrieval counts are reported.

2 Supplementary Figures and Tables

2.1 Supplementary Figures

Supplementary Figure B1. Objective-response total-treatment-arm denominator sensitivity forest plot.

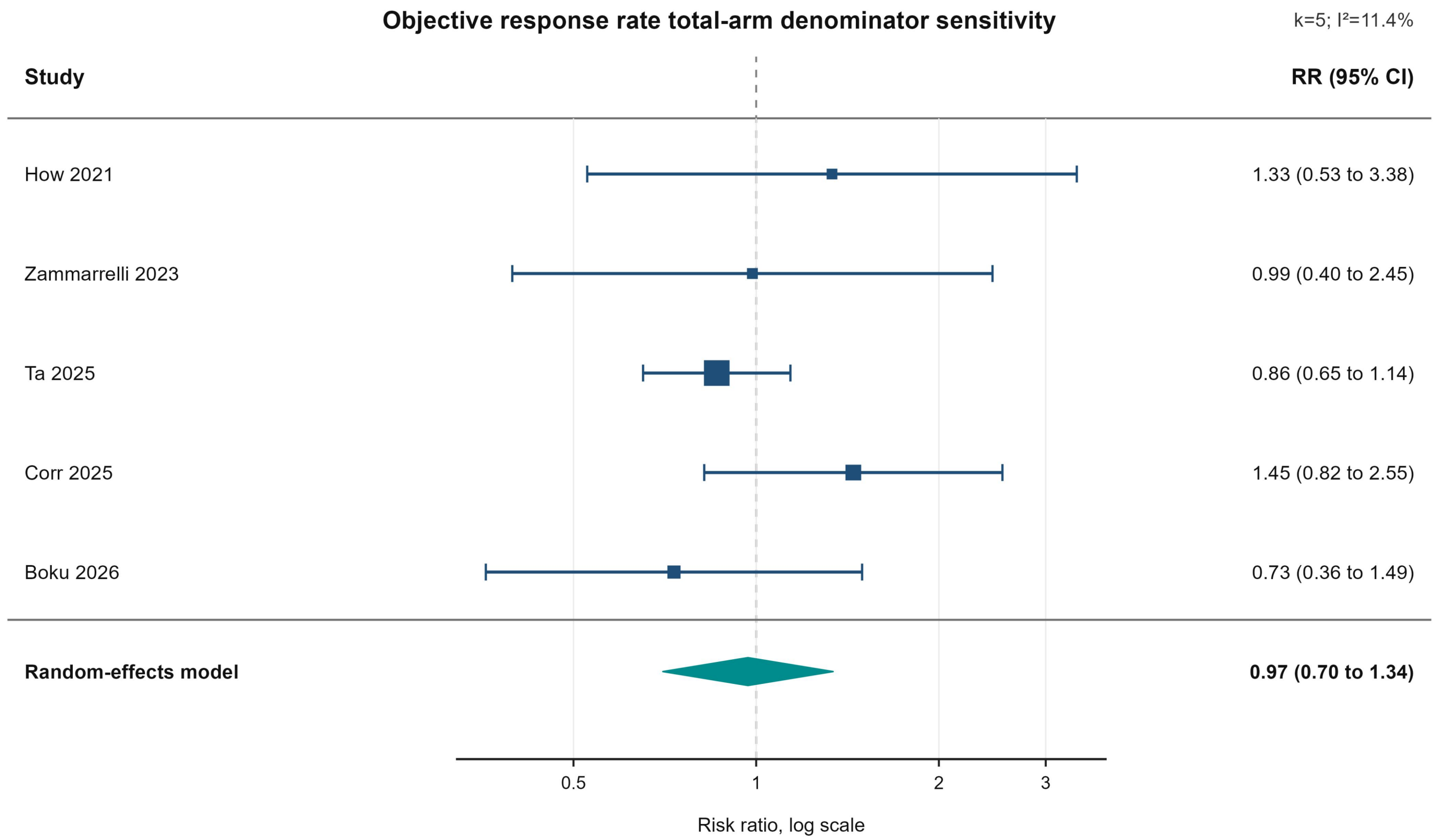

Sensitivity analysis using the total treated population as the denominator. Numerical similarity to the primary analysis is not evidence of equivalence, noninferiority, or preserved efficacy.

Supplementary Figure B2. Disease-control-rate forest plot.

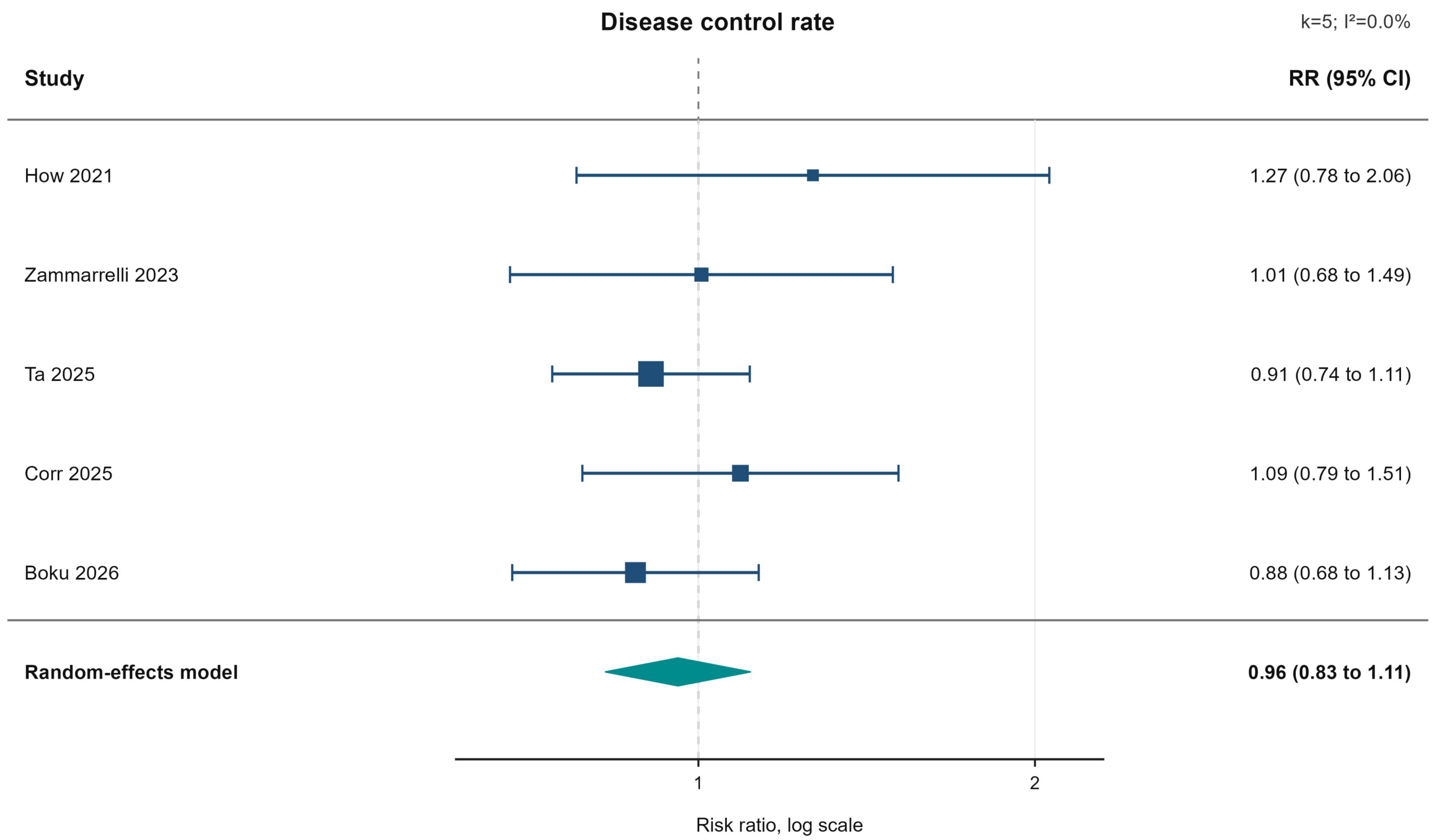

Random-effects supplementary analysis. Source-specific disease-control terminology was harmonized to the prespecified construct; disease control was not a co-primary outcome.

Supplementary Figure B3. Treatment-interruption forest plot.

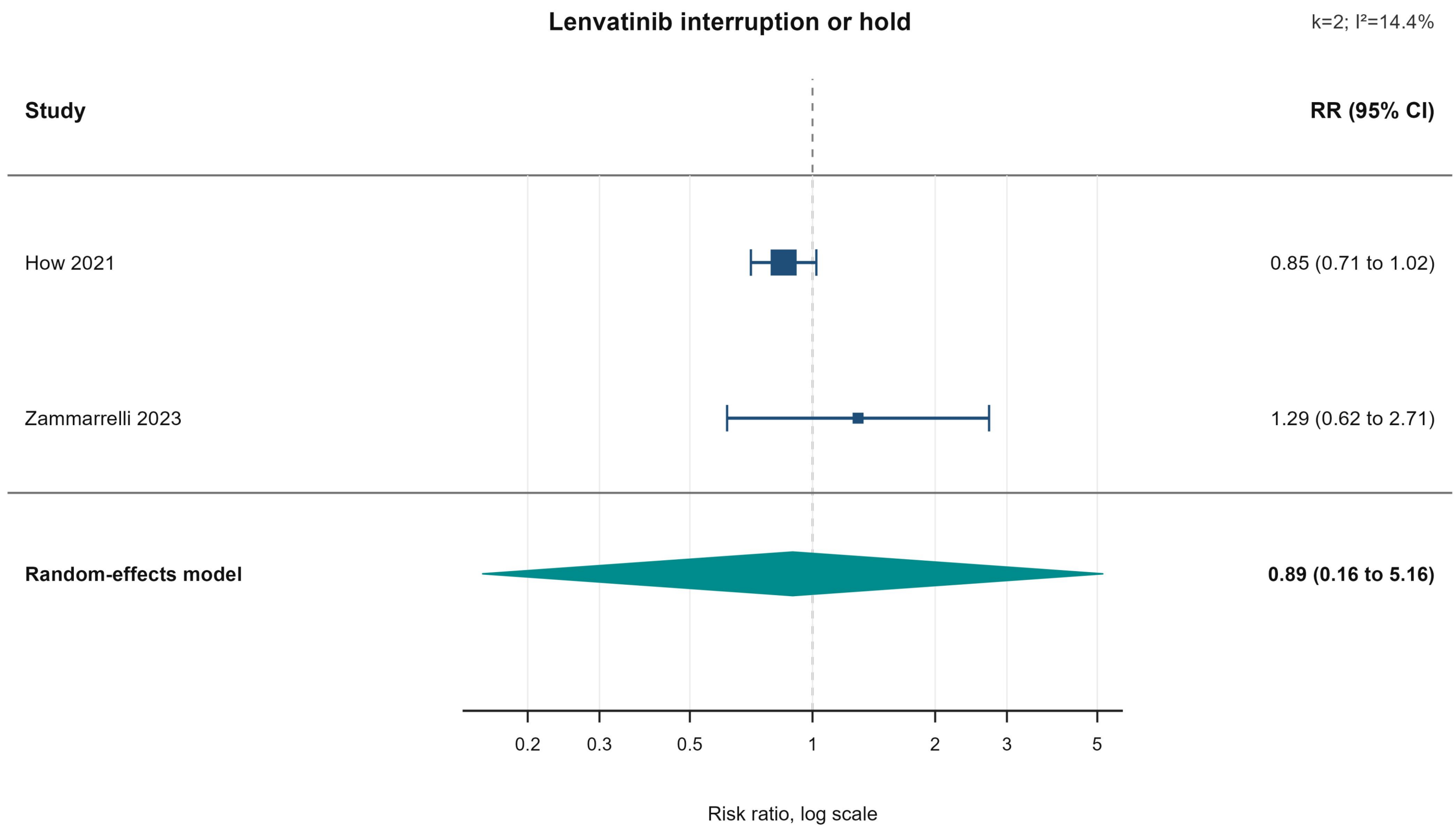

Random-effects supplementary analysis. Temporary interruption or treatment hold was kept separate from permanent discontinuation.

Supplementary Figure B4. Combination-treatment toxicity-discontinuation forest plot.

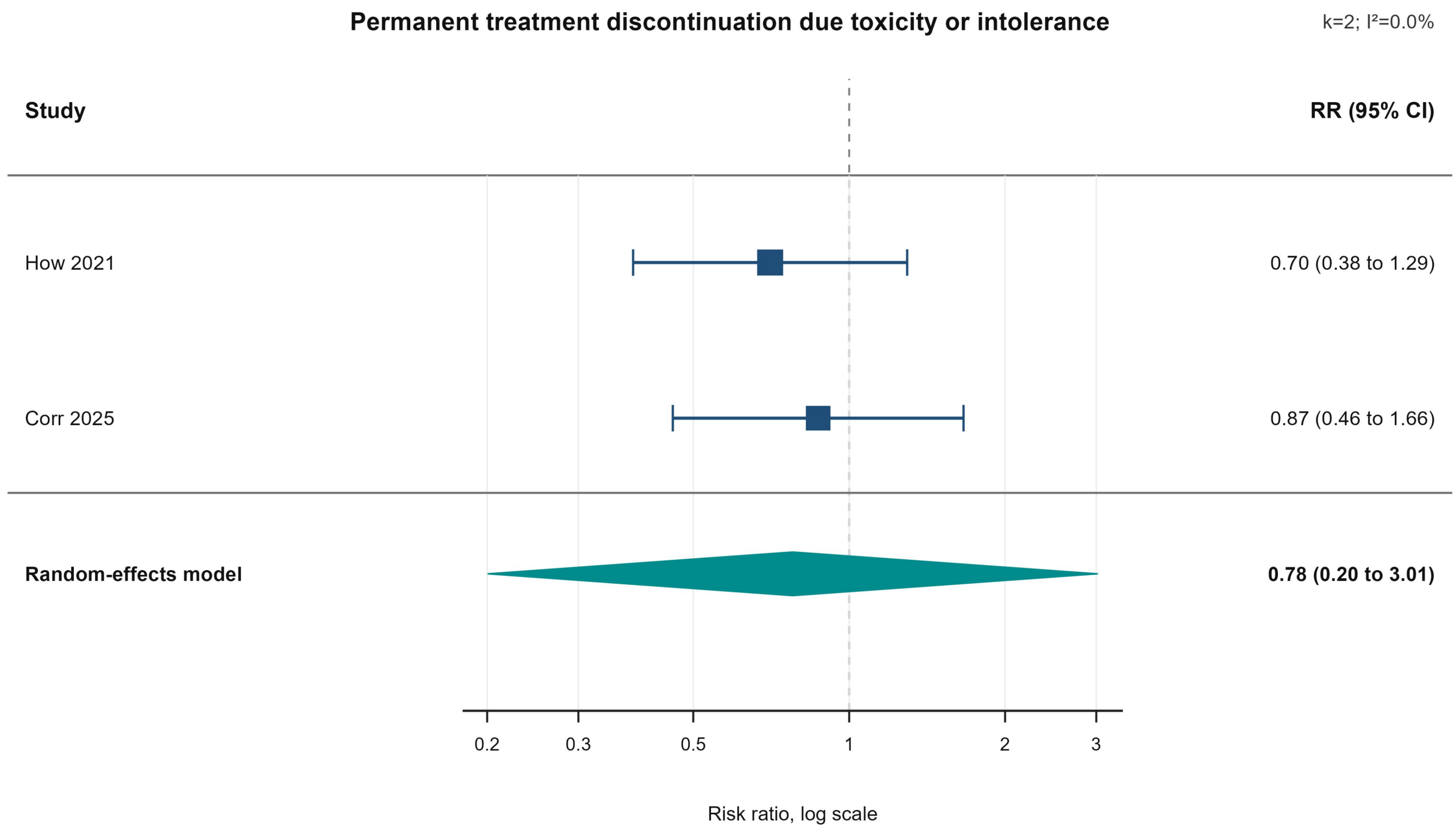

Random-effects exploratory analysis of combination-level discontinuation due to toxicity or intolerance. Lenvatinib-specific discontinuation was analyzed separately.

Supplementary Figure B5. Any-toxicity discontinuation forest plot for the 10-mg threshold sensitivity estimand.

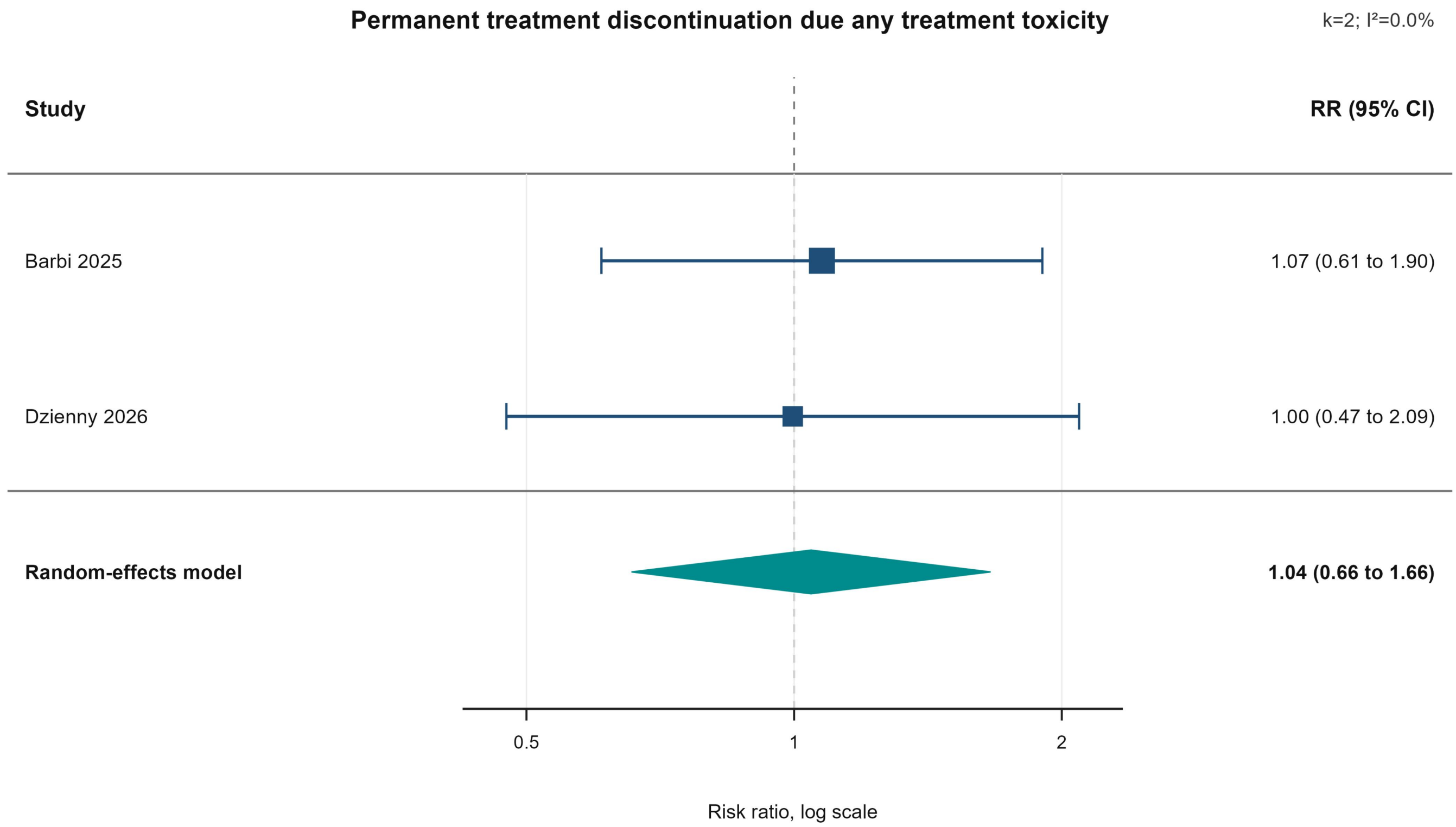

Sensitivity analysis of the separate starting-dose contrast of 10 mg or less versus more than 10 mg. This contrast was not combined with the primary comparison of below 20 mg versus 20 mg.

Supplementary Figure B6. Exploratory progression-free-survival forest plot.

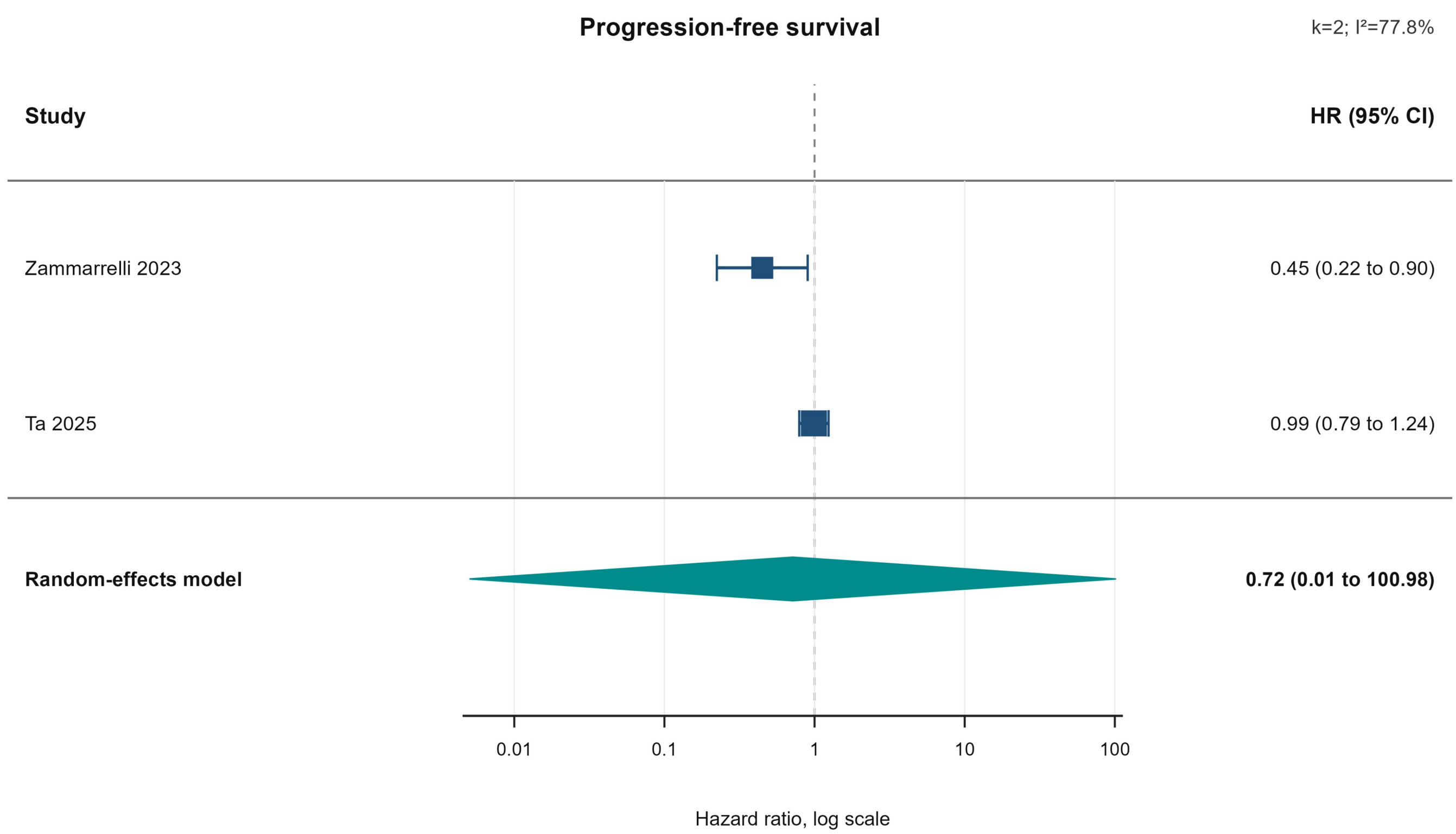

Exploratory synthesis of reported unadjusted hazard ratios from two cohorts. Adjusted and unadjusted effects remained separate. The 95% Hartung–Knapp interval (0.005–100.98) spans more than four orders of magnitude and is quantitatively uninformative. The pooled diamond is retained only to document the prespecified exploratory calculation; the study-level estimates are the interpretable elements. This analysis does not support equivalence, noninferiority, or preserved efficacy.

Supplementary Figure B7. Exploratory grade 3 or higher adverse-event forest plot.

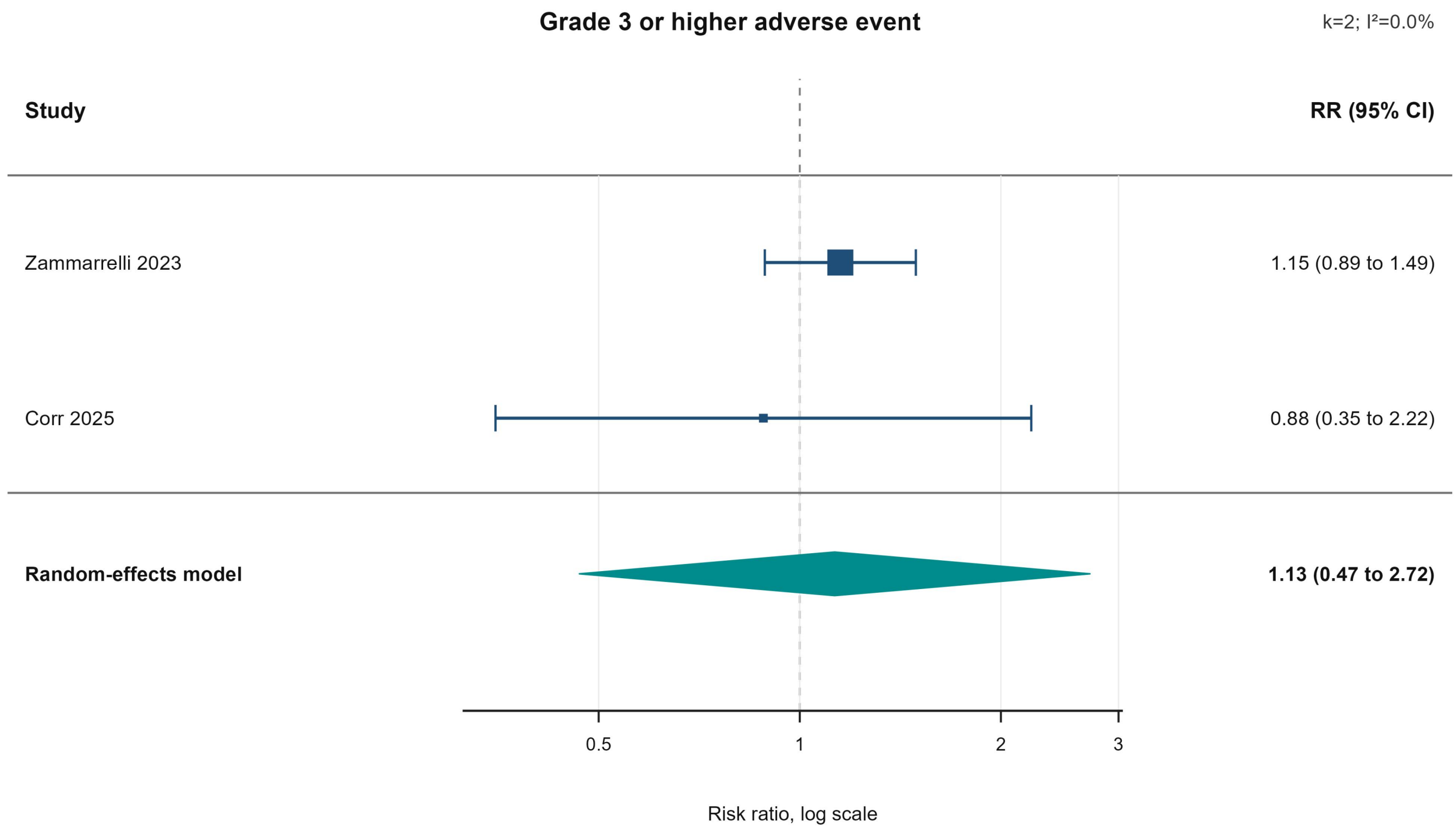

Random-effects exploratory safety analysis. The small number of studies and possible retrospective underascertainment limit interpretation.

2.2 Supplementary tables

Supplementary Table C1. Outcome definitions, cutoffs, and time points

Study	Outcome domain	Original outcome definition	Harmonized outcome label	Cutoff or threshold	Time point	Measurement instrument	Unit	Direction of beneficial effect	Compatibility decision	Source report and location
How 2021	Objective response rate	radiologically evaluable denominator	Objective response rate	No equivalence or noninferiority margin was prespecified	best response during treatment	chart abstraction; source-specific response/safety criteria	participants with event	benefit	Eligible for the main <20 mg versus 20 mg analysis.	How 2021, p. 19, Results, Table 3A
Zammarrelli 2023	Objective response rate	published percentages reproduce total-arm denominators; 2 response-missing patients in 20 mg arm retained and flagged for sensitivity	Objective response rate	No equivalence or noninferiority margin was prespecified	best response during treatment	chart abstraction; source-specific response/safety criteria	participants with event	benefit	Eligible for the main <20 mg versus 20 mg analysis.	Zammarrelli 2023, p. 18, Results, Table 3
Ta 2025	Objective response rate	CR+PR with total-arm denominators; 15 not-assessed in each arm	Objective response rate	No equivalence or noninferiority margin was prespecified	best response during treatment	chart abstraction; source-specific response/safety criteria	participants with event	benefit	Eligible for the main <20 mg versus 20 mg analysis.	Ta 2025, p. 4, Results, Table 2
Corr 2025	Objective response rate	harmonized CR+PR; one response-NOS in 14 mg arm excluded from prespecified objective-response definition	Objective response rate	No equivalence or noninferiority margin was prespecified	best response during treatment	chart abstraction; source-specific response/safety criteria	participants with event	benefit	Eligible for the main <20 mg versus 20 mg analysis; shared control handled once.	Corr 2025, p. 4, Results, Table 2
Boku 2026	Objective response rate	CR plus PR	Objective response rate	No equivalence or noninferiority margin was prespecified	best response during treatment	chart abstraction; source-specific response/safety criteria	participants with event	benefit	Eligible for the main <20 mg versus 20 mg analysis.	Boku 2026, p. Supplement DOCX, Results, Supplementary Table 1
How 2021	Disease control rate	source label clinical benefit rate; definition equals CR+PR+SD	Disease control rate	No equivalence or noninferiority margin was prespecified	best response during treatment	chart abstraction; source-specific response/safety criteria	participants with event	benefit	Eligible for the main <20 mg versus 20 mg analysis.	How 2021, p. 19, Results, Table 3A
Zammarrelli 2023	Disease control rate	source label clinical benefit rate; total-arm denominator	Disease control rate	No equivalence or noninferiority margin was prespecified	best response during treatment	chart abstraction; source-specific response/safety criteria	participants with event	benefit	Eligible for the main <20 mg versus 20 mg analysis.	Zammarrelli 2023, p. 18, Results, Table 3
Ta 2025	Disease control rate	calculated from mutually exclusive CR+PR+SD categories	Disease control rate	No equivalence or noninferiority margin was prespecified	best response during treatment	chart abstraction; source-specific response/safety criteria	participants with event	benefit	Eligible for the main <20 mg versus 20 mg analysis.	Ta 2025, p. 4, Results, Table 2
Corr 2025	Disease control rate	harmonized CR+PR+SD; response-NOS excluded	Disease control rate	No equivalence or noninferiority margin was prespecified	best response during treatment	chart abstraction; source-specific response/safety criteria	participants with event	benefit	Eligible for the main <20 mg versus 20 mg analysis; shared control handled once.	Corr 2025, p. 4, Results, Table 2
Boku 2026	Disease control rate	CR plus PR plus SD; non-CR/non-PD included only where source definition explicitly did so	Disease control rate	No equivalence or noninferiority margin was prespecified	best response during treatment	chart abstraction; source-specific response/safety criteria	participants with event	benefit	Eligible for the main <20 mg versus 20 mg analysis.	Boku 2026, p. Supplement DOCX, Results, Supplementary Table 1
Zammarrelli 2023	Grade 3 or higher adverse event	at least one grade >=3 adverse event	Grade 3 or higher adverse event	No equivalence or noninferiority margin was prespecified	during treatment	chart abstraction; source-specific response/safety criteria	participants with event	harm	Eligible for the main <20 mg versus 20 mg analysis.	Zammarrelli 2023, p. 18, Results, Table

Study	Outcome domain	Original outcome definition	Harmonized outcome label	Cutoff or threshold	Time point	Measurement instrument	Unit	Direction of beneficial effect	Compatibility decision	Source report and location
										3
Corr 2025	Grade 3 or higher adverse event	combined mutually exclusive 10/14/18 mg arms	Grade 3 or higher adverse event	No equivalence or noninferiority margin was prespecified	during treatment	chart abstraction; source-specific response/safety criteria	participants with event	harm	Eligible for the main <20 mg versus 20 mg analysis; shared control handled once.	Corr 2025, p. 5, Results, Table 4
How 2021	Subsequent lenvatinib dose reduction	>=1 further reduction derived as total minus zero reductions	Subsequent lenvatinib dose reduction	No equivalence or noninferiority margin was prespecified	during treatment	chart abstraction; source-specific response/safety criteria	participants with event	harm	Eligible for the main <20 mg versus 20 mg analysis.	How 2021, p. 18, Results, Table 2
Zammarrelli 2023	Subsequent lenvatinib dose reduction	at least one postbaseline reduction after the assigned starting dose	Subsequent lenvatinib dose reduction	No equivalence or noninferiority margin was prespecified	during treatment	chart abstraction; source-specific response/safety criteria	participants with event	harm	Eligible for the main <20 mg versus 20 mg analysis.	Zammarrelli 2023, p. 15, Results, Table 1
Corr 2025	Subsequent lenvatinib dose reduction	at least one postbaseline reduction after the assigned starting dose	Subsequent lenvatinib dose reduction	No equivalence or noninferiority margin was prespecified	during treatment	chart abstraction; source-specific response/safety criteria	participants with event	harm	Eligible for the main <20 mg versus 20 mg analysis; shared control handled once.	Corr 2025, p. 4, Results, Table 2
How 2021	Lenvatinib interruption or hold	at least one postbaseline interruption/hold	Lenvatinib interruption or hold	No equivalence or noninferiority margin was prespecified	during treatment	chart abstraction; source-specific response/safety criteria	participants with event	harm	Eligible for the main <20 mg versus 20 mg analysis.	How 2021, p. 18, Results, Table 2
Zammarrelli 2023	Lenvatinib interruption or hold	source term lenvatinib hold	Lenvatinib interruption or hold	No equivalence or noninferiority margin was prespecified	during treatment	chart abstraction; source-specific response/safety criteria	participants with event	harm	Eligible for the main <20 mg versus 20 mg analysis.	Zammarrelli 2023, p. 15, Results, Table 1
How 2021	Permanent discontinuation due to toxicity or intolerance	pembrolizumab and/or lenvatinib stopped due toxicity	Permanent discontinuation due to toxicity or intolerance	No equivalence or noninferiority margin was prespecified	during treatment	chart abstraction; source-specific response/safety criteria	participants with event	harm	Eligible for the main <20 mg versus 20 mg analysis.	Source report; exact location recorded during extraction toxicity-attributed outcome definition
Zammarrelli 2023	Permanent discontinuation due to toxicity or intolerance	lenvatinib discontinued; zero exposed events requires prespecified boundary rule	Permanent discontinuation due to toxicity or intolerance	No equivalence or noninferiority margin was prespecified	during treatment	chart abstraction; source-specific response/safety criteria	participants with event	harm	Eligible for the main <20 mg versus 20 mg analysis.	Source report; exact location recorded during extraction toxicity-attributed outcome definition
Corr 2025	Permanent discontinuation due to toxicity or intolerance	source term inability to tolerate treatment; reasons reported among discontinuers	Permanent discontinuation due to toxicity or intolerance	No equivalence or noninferiority margin was prespecified	during treatment	chart abstraction; source-specific response/safety criteria	participants with event	harm	Eligible for the main <20 mg versus 20 mg analysis; shared control handled once.	Source report; exact location recorded during extraction toxicity-attributed outcome definition
Corr 2025	Permanent discontinuation, all cause	treatment permanently discontinued for any reported cause	Permanent discontinuation, all cause	No equivalence or noninferiority margin was prespecified	during treatment	chart abstraction; source-specific response/safety criteria	participants with event	harm	Eligible for supplementary exploratory analysis within the <20 mg versus 20 mg estimand.	Source report; exact location recorded during extraction all-cause outcome definition

Study	Outcome domain	Original outcome definition	Harmonized outcome label	Cutoff or threshold	Time point	Measurement instrument	Unit	Direction of beneficial effect	Compatibility decision	Source report and location
How 2021	All-cause hospitalization	on-treatment follow-up	All-cause hospitalization	No equivalence or noninferiority margin was prespecified	reported follow-up window	chart abstraction; source-specific response/safety criteria	participants with event	harm	Eligible for narrative synthesis and exploratory pooling within the <20 mg versus 20 mg estimand.	Source report; exact location recorded during extraction all-cause outcome definition
Ta 2025	All-cause hospitalization	within 6 months; not directly time-compatible with How 2021	All-cause hospitalization	No equivalence or noninferiority margin was prespecified	6 months	chart abstraction; source-specific response/safety criteria	participants with event	harm	Eligible for narrative synthesis and exploratory pooling within the <20 mg versus 20 mg estimand.	Source report; exact location recorded during extraction all-cause outcome definition
How 2021	Toxicity-related hospitalization	at least one hospitalization attributed to treatment toxicity	Toxicity-related hospitalization	No equivalence or noninferiority margin was prespecified	during treatment	chart abstraction; source-specific response/safety criteria	participants with event	harm	Eligible for supplementary exploratory analysis within the <20 mg versus 20 mg estimand.	Source report; exact location recorded during extraction toxicity-attributed outcome definition
Ta 2025	Six-month progression-free survival	alive and progression-free at 6 months	Six-month progression-free survival	No equivalence or noninferiority margin was prespecified	6 months	chart abstraction; source-specific response/safety criteria	participants with event	benefit	Eligible for supplementary exploratory analysis within the <20 mg versus 20 mg estimand.	Ta 2025, p. 4, Results, Table 2
Dzienny 2026	Objective response rate	CR plus PR	Objective response rate	No equivalence or noninferiority margin was prespecified	best response during treatment	chart abstraction; source-specific response/safety criteria	participants with event	benefit	Eligible for the separate ≤10 mg versus >10 mg sensitivity estimand.	Dzienny 2026, p. 4; Supplement slide 2, Results, Table 2; Table S2
Dzienny 2026	Disease control rate	CR+PR+SD from reported categories	Disease control rate	No equivalence or noninferiority margin was prespecified	best response during treatment	chart abstraction; source-specific response/safety criteria	participants with event	benefit	Eligible for the separate ≤10 mg versus >10 mg sensitivity estimand.	Dzienny 2026, p. 4; Supplement slide 2, Results, Table 2; Table S2
Barbi 2025	Grade 2 or higher adverse event	cannot convert to prespecified grade ≥3 outcome	Grade 2 or higher adverse event	No equivalence or noninferiority margin was prespecified	during treatment	chart abstraction; source-specific response/safety criteria	participants with event	harm	Narrative only within the separate ≤10 mg versus >10 mg estimand.	Barbi 2025, p. 5, Results, Table 2
Barbi 2025	Subsequent lenvatinib dose reduction	at least one postbaseline reduction after the assigned starting dose	Subsequent lenvatinib dose reduction	No equivalence or noninferiority margin was prespecified	during treatment	chart abstraction; source-specific response/safety criteria	participants with event	harm	Eligible for the separate ≤10 mg versus >10 mg sensitivity estimand.	Barbi 2025, p. 5, Results, Table 2
Barbi 2025	Lenvatinib interruption or hold	at least one postbaseline interruption/hold	Lenvatinib interruption or hold	No equivalence or noninferiority margin was prespecified	during treatment	chart abstraction; source-specific response/safety criteria	participants with event	harm	Eligible for the separate ≤10 mg versus >10 mg sensitivity estimand.	Barbi 2025, p. 5, Results, Table 2
Barbi 2025	Permanent discontinuation due to toxicity or intolerance	report labels treatment discontinuation within toxicity table; exact attribution retained as reported	Permanent discontinuation due to toxicity or intolerance	No equivalence or noninferiority margin was prespecified	during treatment	chart abstraction; source-specific response/safety criteria	participants with event	harm	Eligible for the separate ≤10 mg versus >10 mg sensitivity estimand.	Source report; exact location recorded during extraction toxicity-attributed outcome definition

Study	Outcome domain	Original outcome definition	Harmonized outcome label	Cutoff or threshold	Time point	Measurement instrument	Unit	Direction of beneficial effect	Compatibility decision	Source report and location
Dzienny 2026	Permanent discontinuation due to toxicity or intolerance	lenvatinib-toxicity-specific discontinuation	Permanent discontinuation due to toxicity or intolerance	No equivalence or noninferiority margin was prespecified	during treatment	chart abstraction; source-specific response/safety criteria	participants with event	harm	Eligible for the separate ≤10 mg versus >10 mg sensitivity estimand.	Source report; exact location recorded during extraction toxicity-attributed outcome definition
Nagase 2025	Objective response rate	counts reconstructed exactly from 40.3% and 12.1%; denominators shown in Figure 3 risk table	Objective response rate	No equivalence or noninferiority margin was prespecified	best response during treatment	chart abstraction; source-specific response/safety criteria	participants with event	benefit	Eligible for the first-8-week relative-dose-intensity supplementary estimand.	Nagase 2025, p. 5, Results, Results
Nagase 2025	Disease control rate	counts reconstructed exactly from 84.7% and 57.6%; denominators shown in Figure 3 risk table	Disease control rate	No equivalence or noninferiority margin was prespecified	best response during treatment	chart abstraction; source-specific response/safety criteria	participants with event	benefit	Eligible for the first-8-week relative-dose-intensity supplementary estimand.	Nagase 2025, p. 5, Results, Results
Zammarrelli 2023	Progression-free survival	time from regimen initiation to progression or death	Progression-free survival	no equivalence or noninferiority threshold	full follow-up	Cox or reported Kaplan-Meier comparison	hazard ratio	benefit	Eligible for the main <20 mg versus 20 mg analysis.	Zammarrelli 2023, p. 19, Results, Table 4
Zammarrelli 2023	Overall survival	time from regimen initiation to death from any cause	Overall survival	no equivalence or noninferiority threshold	full follow-up	Cox or reported Kaplan-Meier comparison	hazard ratio	benefit	Eligible for the main <20 mg versus 20 mg analysis.	Zammarrelli 2023, p. 19, Results, Table 4
Ta 2025	Progression-free survival	time from regimen initiation to progression or death	Progression-free survival	no equivalence or noninferiority threshold	full follow-up	Cox or reported Kaplan-Meier comparison	hazard ratio	benefit	Eligible for the main <20 mg versus 20 mg analysis.	Ta 2025, p. 4, Results, Results/Kaplan-Meier comparison
Boku 2026	Progression-free survival	time from regimen initiation to progression or death	Progression-free survival	no equivalence or noninferiority threshold	full follow-up	Cox or reported Kaplan-Meier comparison	hazard ratio	benefit	Eligible for the main <20 mg versus 20 mg analysis.	Boku 2026, pp. 6,14; published XML, Results, Table 3
Boku 2026	Overall survival	time from regimen initiation to death from any cause	Overall survival	no equivalence or noninferiority threshold	full follow-up	Cox or reported Kaplan-Meier comparison	hazard ratio	benefit	Eligible for the main <20 mg versus 20 mg analysis.	Boku 2026, pp. 6,14; published XML, Results, Table 4/VOR XML
Barbi 2025	Progression-free survival	time from regimen initiation to progression or death	Progression-free survival	no equivalence or noninferiority threshold	full follow-up	Cox or reported Kaplan-Meier comparison	hazard ratio	benefit	Eligible for the separate ≤10 mg versus >10 mg sensitivity estimand.	Barbi 2025, p. 7, Results, Figure 3; Table 3
Dzienny 2026	Progression-free survival	time from regimen initiation to progression or death	Progression-free survival	no equivalence or noninferiority threshold	full follow-up	Cox or reported Kaplan-Meier comparison	hazard ratio	benefit	Eligible for the separate ≤10 mg versus >10 mg sensitivity estimand.	Dzienny 2026, p. 4, Results, Figure 2a
Nagase 2025	Progression-free survival	time from regimen initiation to progression or death	Progression-free survival	no equivalence or noninferiority threshold	full follow-up	Cox or reported Kaplan-Meier comparison	hazard ratio	benefit	Eligible for the first-8-week relative-dose-intensity supplementary estimand.	Nagase 2025, p. 5–6, Results, Figure 3A; Table 3
Nagase 2025	Overall survival	time from regimen	Overall survival	no equivalence or	full follow-up	Cox or reported Kaplan-	hazard ratio	benefit	Eligible for the first-8-	Nagase 2025,

Study	Outcome domain	Original outcome definition	Harmonized outcome label	Cutoff or threshold	Time point	Measurement instrument	Unit	Direction of beneficial effect	Compatibility decision	Source report and location
		initiation to death from any cause		noninferiority threshold		Meier comparison			week relative-dose-intensity supplementary estimand.	pp. 1,5–6, Results, Abstract; Figure 3B
Takahashi 2026	Objective response rate	complete response plus partial response among patients with measurable target lesions	Objective response rate	first-8-week RDI >=56% versus <56%; same-cohort ROC-derived threshold	best response during treatment	RECIST version 1.1; retrospective assessment	participants with event	benefit	Narrative only: the 56% threshold and response-evaluable denominator are incompatible with the prespecified 50% relative-dose-intensity analysis.	Takahashi 2026, p. PDF p.4 (journal 707), Results, Figure S3A cited
Takahashi 2026	Progression-free survival	time from lenvatinib plus pembrolizumab initiation to disease progression or death	Progression-free survival	first-8-week RDI >=56% versus <56%; same-cohort ROC-derived threshold	full follow-up after first-8-week exposure window	Kaplan-Meier and Cox regression	hazard ratio	benefit	Narrative only: adjusted and reported unadjusted estimates remain separate, and the 56% threshold is not combined with the prespecified 50% threshold.	Takahashi 2026, p. PDF p.6 (journal 709), Results, Table 3; Takahashi 2026, p. PDF p.4 (journal 707), Results, Figure S3B cited but unavailable

Note: Not reported indicates that the eligible source did not provide the item; missing values were not imputed.

Supplementary Table C2. Target-trial specifications and study-level exposure definitions

Record level	Study or estimand	Population or eligible cohort	Exposure strategy	Comparator	Time zero and follow-up	Measurement	Effect of interest	Key baseline or time-varying confounders	Harmonization or bias red line	Specification source
Estimand-level target-trial specification	Starting dose <20 mg versus 20 mg	Eligible adults with advanced or recurrent endometrial cancer initiating lenvatinib plus pembrolizumab	Assign lenvatinib starting dose <20 mg versus 20 mg at first administration	Defined within the strategy contrast	Time zero: combination-treatment initiation; follow-up: outcome-specific from treatment initiation	Baseline prescription assignment	effect of assignment to baseline starting-dose strategy	Baseline: age; frailty/comorbidity; ECOG; renal/hepatic function; BMI/nutrition; histology/grade; MMR/p53/molecular subtype; disease burden/metastatic sites; platinum-free interval; prior treatment lines; center/calendar time; physician prescribing preference; time-varying: not required for assignment effect; later reductions are outcomes/deviations in usual practice	Do not infer equivalence/noninferiority from non-significance; unadjusted and adjusted effects remain separate.	Target-trial specification: primary starting-dose estimand
Estimand-level target-trial specification	Starting dose ≤10 mg versus >10 mg	Eligible adults initiating lenvatinib plus pembrolizumab in threshold-comparison cohorts	Assign lenvatinib starting dose <=10 mg versus >10 mg at first administration	Defined within the strategy contrast	Time zero: combination-treatment initiation; follow-up: outcome-specific from treatment initiation	Baseline prescription assignment	effect of assignment to threshold-defined baseline starting-dose strategy	Baseline: age; frailty/comorbidity; ECOG; renal/hepatic function; BMI/nutrition; histology/grade; MMR/p53/molecular subtype; disease burden/metastatic sites; platinum-free interval; prior treatment lines; center/calendar time; physician prescribing preference; time-varying: not required for assignment effect; later reductions are outcomes	>10 mg is not equivalent to a 20 mg standard-dose control; post hoc noninferiority language is not adopted.	Target-trial specification: 10-mg threshold estimand
Estimand-level target-trial specification	First-8-week relative dose intensity	Eligible adults starting combination therapy with accurately monitored dosing	Sustain first-56-day lenvatinib RDI >=50% versus <50% under a defined adherence strategy	Defined within the strategy contrast	Landmark option: start follow-up at day 56 among patients alive and free of the outcome event, using exposure accumulated over days 1–56. Dynamic-strategy option: align eligibility, strategy assignment and	Observed cumulative dose in days 1–56 divided by the planned 20-mg/day cumulative dose; a postbaseline exposure.	Observed postbaseline exposure-outcome association. A landmark estimates a contrast conditional on survival/event-free status at day 56; a causal dynamic-strategy effect requires explicit dosing rules and	Baseline: age; frailty/comorbidity; ECOG; renal/hepatic function; BMI/nutrition; histology/grade; MMR/p53/molecular subtype; disease burden/metastatic sites; platinum-free interval; prior treatment lines; center/calendar time;	Do not treat achieved RDI as a baseline exposure or convert 4-/6-week RDI to 8-week RDI. Landmarking does not itself remove confounding, survivor selection or treatment-confounder feedback.	Target-trial specification: first-8-week RDI estimand

Record level	Study or estimand	Population or eligible cohort	Exposure strategy	Comparator	Time zero and follow-up	Measurement	Effect of interest	Key baseline or time-varying confounders	Harmonization or bias red line	Specification source
					time zero at initiation and model longitudinal dosing. The source analyses began follow-up at treatment initiation.		longitudinal identification assumptions.	physician prescribing preference; time-varying: early toxicity; early progression/response; dose interruption/reduction; adherence; hospitalization; evolving performance status		
Study-level observed exposure definition	How 2021	advanced or recurrent endometrial cancer receiving lenvatinib plus pembrolizumab; MD Anderson Cancer Center; single academic center	reduced starting dosage below 20 mg versus recommended 20 mg	<20 mg versus 20 mg	treatment initiation	prescription record; unit mg/day	Association with assignment to the <20-mg versus 20-mg baseline starting-dose strategy	See Supplementary Information C4 study-level confounding-control summary; adjustment was incomplete for the prespecified critical confounders	No; reduced: 14 mg n45; 10 mg n5; 16 mg n3; 12 mg n1	How 2021, p. 5, Methods/Results, Table 1
Study-level observed exposure definition	Zammarrelli 2023	advanced or recurrent endometrial cancer receiving lenvatinib plus pembrolizumab; Memorial Sloan Kettering Cancer Center; single academic center	10 mg or 14 mg versus 20 mg	10/14 mg versus 20 mg	treatment initiation	electronic medical record; unit mg/day	Association with assignment to the <20-mg versus 20-mg baseline starting-dose strategy	See Supplementary Information C4 study-level confounding-control summary; adjustment was incomplete for the prespecified critical confounders	Aggregate mutually exclusive reduced arms; 10 mg n10; 14 mg n10; 20 mg n23	Zammarrelli 2023, p. 15, Methods/Results, Table 1
Study-level observed exposure definition	Ta 2025	advanced or recurrent endometrial cancer receiving lenvatinib plus pembrolizumab; Kaiser Permanente Northern and Southern California	Source-defined reduced-dose group (n=154): 4 mg (n=1), 8 mg (n=1), 10 mg (n=83), 12 mg (n=13), 14 mg (n=50), alternating 10/20 mg (n=2), 16 mg (n=2), and 18 mg (n=2).	<20 mg daily versus 20 mg daily	treatment initiation	pharmacy/EHR; unit mg/day	Association with assignment to the <20-mg versus 20-mg baseline starting-dose strategy	See Supplementary Information C4 study-level confounding-control summary; adjustment was incomplete for the prespecified critical confounders	Distinct 20-mg five-days-per-week arm (n=11) excluded from every quantitative synthesis and shared-control calculation. Rare schedules remain within the published reduced group because separate outcomes were unavailable.	Ta 2025, p. 3, Methods/Results, Table 1
Study-level observed exposure definition	Corr 2025	advanced or recurrent endometrial cancer receiving lenvatinib plus pembrolizumab; ECMT2 national consortium; 14 institutions	10, 14, 18, or 20 mg	10/14/18 mg versus 20 mg	treatment initiation	multicenter chart abstraction; unit mg/day	Association with assignment to the <20-mg versus 20-mg baseline starting-dose strategy	See Supplementary Information C4 study-level confounding-control summary; adjustment was incomplete for the prespecified critical confounders	Aggregate mutually exclusive reduced arms once; shared 20 mg control must not be duplicated	Corr 2025, p. 3–4, Methods/Results, Tables 1–2
Study-level observed	Boku 2026	advanced or recurrent	less than 20 mg versus 20 mg	<20 mg versus 20 mg	treatment initiation	medical record; unit mg/day	Association with assignment to the	See Supplementary Information C4 study-	No; baseline confounding is marked	Boku 2026, pp. 5,14,

Record level	Study or estimand	Population or eligible cohort	Exposure strategy	Comparator	Time zero and follow-up	Measurement	Effect of interest	Key baseline or time-varying confounders	Harmonization or bias red line	Specification source
exposure definition		endometrial cancer receiving lenvatinib plus pembrolizumab; Kansai Medical University Hospital; single center					<20-mg versus 20-mg baseline starting-dose strategy	level confounding-control summary; adjustment was incomplete for the prespecified critical confounders		Methods/Results, Table 1; Tables 3–4; Supplementary Table 1
Study-level observed exposure definition	Barbi 2025	advanced or recurrent endometrial cancer receiving lenvatinib plus pembrolizumab; Northwell Cancer Institute; real-world cohort	10 mg or less versus more than 10 mg	<=10 mg versus >10 mg	treatment initiation	medical record; unit mg/day	Threshold sensitivity association for <=10 mg versus >10 mg; >10 mg is not a standard-dose control	See Supplementary Information C4 study-level confounding-control summary; adjustment was incomplete for the prespecified critical confounders	Threshold sensitivity only; >10 mg includes 12/14/20 mg; not a standard-dose control	Barbi 2025, p. 3–4, Methods/Results, Table 1
Study-level observed exposure definition	Dzienny 2026	advanced or recurrent endometrial cancer receiving lenvatinib plus pembrolizumab; Yale; single academic tertiary center	10 mg or less versus more than 10 mg	<=10 mg versus >10 mg	treatment initiation	medical record; unit mg/day	Threshold sensitivity association for <=10 mg versus >10 mg; >10 mg is not a standard-dose control	See Supplementary Information C4 study-level confounding-control summary; adjustment was incomplete for the prespecified critical confounders	Threshold sensitivity only; exact groups 4/8/10 versus 12/14/20 mg	Dzienny 2026, p. 3–4, Methods/Results, Table 1; Supplementary Tables S1-S2
Study-level observed exposure definition	Nagase 2025	advanced or recurrent endometrial cancer receiving lenvatinib plus pembrolizumab; Gynecologic Oncology Group of Osaka; 15 institutions	dose delivered during first 8 weeks divided by 20 mg/day for 8 weeks	>=50% versus <50%	first 56 days	dose administration records; unit %	Postbaseline first-8-week RDI exposure-response association; not a baseline starting-dose effect	See Supplementary Information C4 study-level confounding-control summary; adjustment was incomplete for the prespecified critical confounders	Separate postbaseline exposure-response estimand; ROC threshold 48%; prespecified analysis cutoff 50%	Nagase 2025, pp. 2,5–6, Methods/Results, Methods; Figure 3; Table 3
Study-level observed exposure definition	Tochigi 2024	advanced or recurrent endometrial cancer receiving lenvatinib plus pembrolizumab; Tohoku University Hospital; single center	RDI assessed during first 4 weeks and entire treatment	continuous versus continuous	first 4 weeks and whole course	delivered dose divided by 20 mg/day standard dose; unit %	Contextual non-8-week RDI association outside the prespecified first-8-week estimand	See Supplementary Information C4 study-level confounding-control summary; adjustment was incomplete for the prespecified critical confounders	Not harmonizable to first 8 weeks; 14 mg starter n1; 20 mg starters n14; no defensible starting-dose comparison	Tochigi 2024, pp. 2,5, Methods/Results, Methods; Figure 5
Study-level observed exposure definition	Takahashi 2026	recurrent endometrial cancer after platinum-containing chemotherapy; Jichi Medical University Hospital; all started lenvatinib at 20 mg/day	actual cumulative lenvatinib dose during first 8 weeks divided by recommended cumulative 20 mg/day dose	>=56% versus <56% first-8-week RDI	treatment initiation; RDI classified over first 56 days; PFS followed from treatment initiation	dose administration records; unit %	source-specific postbaseline first-8-week RDI association; not a starting-dose effect	Early toxicity, response, interruption, survivor selection, immortal-time risk, reverse causality and time-varying confounding; adjusted model included only histology, platinum-	Do not convert same-cohort ROC-derived 56% to prespecified 50%; do not mix adjusted and reported unadjusted HRs; narrative only	Takahashi 2026, p. PDF p.2 (journal 705), Patients and treatment; Relative dose intensity, none; Takahashi 2026, p. PDF p.4

Record level	Study or estimand	Population or eligible cohort	Exposure strategy	Comparator	Time zero and follow-up	Measurement	Effect of interest	Key baseline or time-varying confounders	Harmonization or bias red line	Specification source
								free interval and RDI		(journal 707), Results, Figure S3A cited; Takahashi 2026, p. PDF p.7 (journal 710), Discussion, none

Note: The framework clarifies the causal questions but does not imply that the retrospective studies emulated a target trial.

Supplementary Table C3. Study-level confounding-control summary and complete result-level ROBINS-I details

Study	Tool	Outcome or result	Domain	Judgment	Support for judgment	Information source	Overall judgment	Additional rationale	Figure link
How 2021	ROBINS-I 2016 (study-level confounding-control summary; result-level judgments remain below)	Starting dose <20 mg versus 20 mg; all eligible effects from this cohort	Confounding-control summary	Serious	Adjustment status: Unadjusted; raw counts. Reported model/covariates: No covariate adjustment reported. Source evidence: the starting dose has to be individualized by patient and practitioner	How et al. (2021) | PDF pp.8–9 | Discussion/limitations	Serious	Age, ECOG/performance status, frailty/comorbidity, organ function, disease burden, histologic/molecular features, prior treatment, center/calendar period, and clinician dose-selection preference were not all controlled. Result-level rationale: Dose choice changed over calendar time and was clinician individualized; comparisons were unadjusted and important residual confounding remained.. Predicted bias direction: Unpredictable.	Figure 3; result-level traffic-light data and signaling-domain records retained below
Zammarrelli 2023	ROBINS-I 2016 (study-level confounding-control summary; result-level judgments remain below)	Starting dose <20 mg versus 20 mg; all eligible effects from this cohort	Confounding-control summary	Serious	Adjustment status: Unadjusted; raw counts; reported unadjusted. Reported model/covariates: No covariate adjustment reported. Source evidence: patients were older and received more lines of treatment	Zammarrelli et al. (2023) | PDF pp.7–9 | Results/Discussion/limitations	Serious	Age, ECOG/performance status, frailty/comorbidity, organ function, disease burden, histologic/molecular features, prior treatment, center/calendar period, and clinician dose-selection preference were not all controlled. Result-level rationale: Reduced-dose recipients were older and more heavily treated; analyses of dose groups were unadjusted and reasons for reduction were incompletely captured.. Predicted bias direction: Unpredictable.	Figure 3; result-level traffic-light data and signaling-domain records retained below
Ta 2025	ROBINS-I 2016 (study-level confounding-control summary; result-level judgments remain below)	Starting dose <20 mg versus 20 mg; all eligible effects from this cohort	Confounding-control summary	Serious	Adjustment status: Unadjusted; raw counts; reported unadjusted. Reported model/covariates: No covariate adjustment reported. Source evidence: lower starting dose based on patient-specific factors affecting tolerability	Ta et al. (2025) | PDF pp.2,6 | Methods/limitations	Serious	Age, ECOG/performance status, frailty/comorbidity, organ function, disease burden, histologic/molecular features, prior treatment, center/calendar period, and clinician dose-selection preference were not all controlled. Result-level rationale: Starting dose reflected clinician assessment; ECOG was largely unknown, prior therapy differed, and the reported dose comparisons were unadjusted.. Predicted bias direction: Unpredictable.	Figure 3; result-level traffic-light data and signaling-domain records retained below
Corr 2025	ROBINS-I 2016 (study-level confounding-control summary; result-level judgments remain below)	Starting dose <20 mg versus 20 mg; all eligible effects from this cohort	Confounding-control summary	Serious	Adjustment status: Unadjusted; raw counts. Reported model/covariates: No covariate adjustment reported. Source evidence: starting doses ... were made at the treating providers' discretion	Corr et al. (2025) | PDF pp.2–4 | Methods/Results/Discussion	Serious	Age, ECOG/performance status, frailty/comorbidity, organ function, disease burden, histologic/molecular features, prior treatment, center/calendar period, and clinician dose-selection preference were not all controlled. Result-level	Figure 3; result-level traffic-light data and signaling-domain records retained below

Study	Tool	Outcome or result	Domain	Judgment	Support for judgment	Information source	Overall judgment	Additional rationale	Figure link
								rationale: Provider-selected dose, age imbalance, and absence of multivariable adjustment leave important confounding uncontrolled.. Predicted bias direction: Unpredictable.	
Boku 2026	ROBINS-I 2016 (study-level confounding-control summary; result-level judgments remain below)	Starting dose <20 mg versus 20 mg; all eligible effects from this cohort	Confounding-control summary	Serious	Adjustment status: Unadjusted; raw counts; adjusted. Reported model/covariates: age >=75 years; ECOG PS >=1; peritoneal metastasis; age >=75 years; starting dose. Source evidence: confounding by indication	Boku et al. (2026) | PDF pp.5–7; Supplementary Table 4 | Results/limitations	Serious	Age, ECOG/performance status, frailty/comorbidity, organ function, disease burden, histologic/molecular features, prior treatment, center/calendar period, and clinician dose-selection preference were not all controlled. Result-level rationale: Large imbalances in age, ECOG, nutrition and prior therapy remained; PFS adjustment included age only and OS modeling was sparse.. Predicted bias direction: Unpredictable.	Figure 3; result-level traffic-light data and signaling-domain records retained below
Barbi 2025	ROBINS-I 2016 (study-level confounding-control summary; result-level judgments remain below)	Starting dose ≤10 mg versus >10 mg; all eligible effects from this cohort	Confounding-control summary	Serious	Adjustment status: Unadjusted; raw counts; adjusted. Reported model/covariates: age at lenvatinib start. Source evidence: clinician-guided based on age, performance status, and anticipated tolerability	Barbi et al. (2025) | PDF p.3 | Statistical analysis/Results	Serious	Age, ECOG/performance status, frailty/comorbidity, organ function, disease burden, histologic/molecular features, prior treatment, center/calendar period, and clinician dose-selection preference were not all controlled. Result-level rationale: Dose was explicitly chosen using prognostic factors; backward elimination did not retain all important confounders.. Predicted bias direction: Unpredictable.	Figure 3; result-level traffic-light data and signaling-domain records retained below
Dzienny 2026	ROBINS-I 2016 (study-level confounding-control summary; result-level judgments remain below)	Starting dose ≤10 mg versus >10 mg; all eligible effects from this cohort	Confounding-control summary	Serious	Extracted RR/HR: unadjusted. Other reported analyses include matched ORR risk differences (dMMR, grade, platinum status, ECOG, age, BMI) and a Cox model with continuous dose; no adjusted binary RR/OR was extractable.	Dzienny et al. (2026) | PDF pp.3–4; Supplementary Tables S1 and S5 | Statistical analysis/Results	Serious	Age, ECOG/performance status, frailty/comorbidity, organ function, disease burden, histologic/molecular features, prior treatment, center/calendar period, and clinician dose-selection preference were not all controlled. Result-level rationale: Dose selection used strong prognostic factors and dose groups remained substantially imbalanced despite exploratory matching.. Predicted bias direction: Unpredictable.	Figure 3; result-level traffic-light data and signaling-domain records retained below
Nagase 2025	ROBINS-I 2016 (study-level confounding-control summary; result-level judgments remain below)	First-8-week relative dose intensity; all eligible effects from this cohort	Confounding-control summary	Serious	Adjustment status: Unadjusted; raw counts; reported unadjusted; adjusted. Reported model/covariates: age; ECOG PS; PFI; histology; FIGO stage; MMR status; 8w-RDI. Source evidence: decisions regarding dose reduction or interruption	Nagase et al. (2025) | PDF pp.2,5,8 | Study definitions/RDI results/limitations	Serious	Even with reported adjustment, early toxicity, early response, treatment interruption, survivor selection, immortal time, and time-varying confounding remain critical. Result-level rationale: Achieved 8-week RDI was determined by early toxicity, response and clinical	Figure 3; result-level traffic-light data and signaling-domain records retained below

Study	Tool	Outcome or result	Domain	Judgment	Support for judgment	Information source	Overall judgment	Additional rationale	Figure link
								decisions; no time-varying-confounding method was used.. Predicted bias direction: Unpredictable.	
Tochigi 2024	ROBINS-I 2016 (study-level confounding-control summary; result-level judgments remain below)	Contextual exposure record; all eligible effects from this cohort	Confounding-control summary	Serious	Adjustment status: No comparative effect extracted. Reported model/covariates: No covariate adjustment reported. Source evidence: all 15 patients required AE-related treatment interruptions	Tochigi et al. (2024) | PDF pp.2,5 | Methods/RDI results	Serious	The non-8-week RDI exposure is postbaseline and cannot be aligned to the prespecified first-8-week estimand; time-varying confounding remains critical. Result-level rationale: RDI was strongly determined by postbaseline toxicity and treatment duration; no baseline or time-varying confounding adjustment was performed.. Predicted bias direction: Unpredictable.	Figure 3; result-level traffic-light data and signaling-domain records retained below
How 2021	ROBINS-I 2016 2016 original cohort-type version (19 September 2016)	How 2021: hospitalization all-cause outcome definition	D1 Bias due to confounding	Serious	the starting dose has to be individualized by patient and practitioner	How et al. (2021) | PDF pp.8–9 | Discussion/limitations	Serious	Dose choice changed over calendar time and was clinician individualized; comparisons were unadjusted and important residual confounding remained.	Figure 3; complete result-level details retained in this table
How 2021	ROBINS-I 2016 2016 original cohort-type version (19 September 2016)	How 2021: hospitalization toxicity-attributed outcome definition	D1 Bias due to confounding	Serious	the starting dose has to be individualized by patient and practitioner	How et al. (2021) | PDF pp.8–9 | Discussion/limitations	Serious	Dose choice changed over calendar time and was clinician individualized; comparisons were unadjusted and important residual confounding remained.	Figure 3; complete result-level details retained in this table
How 2021	ROBINS-I 2016 2016 original cohort-type version (19 September 2016)	How 2021: hospitalization all-cause outcome definition	D2 Bias in selection of participants into the study	Moderate	excluded ... loss to follow-up without any documentation	How et al. (2021) | PDF pp.3–4 | Methods/Patient population	Serious	Eligibility required treatment initiation, but patients without post-initiation outcome documentation could be excluded.	Figure 3; complete result-level details retained in this table
How 2021	ROBINS-I 2016 2016 original cohort-type version (19 September 2016)	How 2021: hospitalization toxicity-attributed outcome definition	D2 Bias in selection of participants into the study	Moderate	excluded ... loss to follow-up without any documentation	How et al. (2021) | PDF pp.3–4 | Methods/Patient population	Serious	Eligibility required treatment initiation, but patients without post-initiation outcome documentation could be excluded.	Figure 3; complete result-level details retained in this table
How 2021	ROBINS-I 2016 2016 original cohort-type version (19 September 2016)	How 2021: hospitalization all-cause outcome definition	D3 Bias in classification of interventions	Low	lenvatinib starting dosage	How et al. (2021) | PDF pp.3–5 | Clinical data collection/Results	Serious	Starting dose was clearly defined and abstracted from contemporaneous electronic medical records.	Figure 3; complete result-level details retained in this table
How 2021	ROBINS-I 2016 2016 original cohort-type version (19 September 2016)	How 2021: hospitalization toxicity-attributed outcome definition	D3 Bias in classification of interventions	Low	lenvatinib starting dosage	How et al. (2021) | PDF pp.3–5 | Clinical data collection/Results	Serious	Starting dose was clearly defined and abstracted from contemporaneous electronic medical records.	Figure 3; complete result-level details retained in this table
How 2021	ROBINS-I 2016 2016 original cohort-type version (19 September 2016)	How 2021: hospitalization all-cause outcome definition	D4 Bias due to deviations from intended interventions	Low	based on initial lenvatinib dosage level	How et al. (2021) | PDF p.4 | Statistical analysis	Serious	The prespecified estimand is assignment to the starting strategy; usual-practice later reductions and interruptions are outcomes rather than protocol deviations.	Figure 3; complete result-level details retained in this table
How 2021	ROBINS-I 2016 2016 original cohort-type version (19 September 2016)	How 2021: hospitalization toxicity-attributed outcome definition	D4 Bias due to deviations from intended interventions	Low	based on initial lenvatinib dosage level	How et al. (2021) | PDF p.4 | Statistical analysis	Serious	The prespecified estimand is assignment to the starting strategy; usual-practice later reductions and interruptions are outcomes rather than	Figure 3; complete result-level details retained in this table

Study	Tool	Outcome or result	Domain	Judgment	Support for judgment	Information source	Overall judgment	Additional rationale	Figure link
								protocol deviations.	
How 2021	ROBINS-I 2016 2016 original cohort-type version (19 September 2016)	How 2021: hospitalization all-cause outcome definition	D5 Bias due to missing data	Moderate	on-treatment hospitalizations	How et al. (2021) | PDF pp.3–5 | Outcome availability/missingness	Serious	Hospitalization records were available but duration/opportunity and cause attribution may vary.	Figure 3; complete result-level details retained in this table
How 2021	ROBINS-I 2016 2016 original cohort-type version (19 September 2016)	How 2021: hospitalization toxicity-attributed outcome definition	D5 Bias due to missing data	Moderate	on-treatment hospitalizations	How et al. (2021) | PDF pp.3–5 | Outcome availability/missingness	Serious	Hospitalization records were available but duration/opportunity and cause attribution may vary.	Figure 3; complete result-level details retained in this table
How 2021	ROBINS-I 2016 2016 original cohort-type version (19 September 2016)	How 2021: hospitalization all-cause outcome definition	D6 Bias in measurement of outcomes	Moderate	on-treatment hospitalizations	How et al. (2021) | PDF pp.3–5 | Outcome measurement	Serious	Admission is objective, but toxicity attribution and follow-up opportunity were not blinded or standardized.	Figure 3; complete result-level details retained in this table
How 2021	ROBINS-I 2016 2016 original cohort-type version (19 September 2016)	How 2021: hospitalization toxicity-attributed outcome definition	D6 Bias in measurement of outcomes	Moderate	on-treatment hospitalizations	How et al. (2021) | PDF pp.3–5 | Outcome measurement	Serious	Admission is objective, but toxicity attribution and follow-up opportunity were not blinded or standardized.	Figure 3; complete result-level details retained in this table
How 2021	ROBINS-I 2016 2016 original cohort-type version (19 September 2016)	How 2021: hospitalization all-cause outcome definition	D7 Bias in selection of the reported result	Moderate	Subgroup analyses were performed for ... toxicity, response, and survival	How et al. (2021) | PDF p.4 | Statistical analysis	Serious	No prespecified public analysis plan was located; multiple outcomes and subgroup analyses were available.	Figure 3; complete result-level details retained in this table
How 2021	ROBINS-I 2016 2016 original cohort-type version (19 September 2016)	How 2021: hospitalization toxicity-attributed outcome definition	D7 Bias in selection of the reported result	Moderate	Subgroup analyses were performed for ... toxicity, response, and survival	How et al. (2021) | PDF p.4 | Statistical analysis	Serious	No prespecified public analysis plan was located; multiple outcomes and subgroup analyses were available.	Figure 3; complete result-level details retained in this table
How 2021	ROBINS-I 2016 2016 original cohort-type version (19 September 2016)	How 2021: hospitalization all-cause outcome definition	Overall Overall risk of bias	Serious	at least one domain was judged Serious	How et al. (2021) | See linked D1-D7 anchors | ROBINS-I overall algorithm	Serious	ROBINS-I 2016 overall judgment equals the highest domain when no domain is Critical.	Figure 3; complete result-level details retained in this table
How 2021	ROBINS-I 2016 2016 original cohort-type version (19 September 2016)	How 2021: hospitalization toxicity-attributed outcome definition	Overall Overall risk of bias	Serious	at least one domain was judged Serious	How et al. (2021) | See linked D1-D7 anchors | ROBINS-I overall algorithm	Serious	ROBINS-I 2016 overall judgment equals the highest domain when no domain is Critical.	Figure 3; complete result-level details retained in this table
How 2021	ROBINS-I 2016 2016 original cohort-type version (19 September 2016)	OS | Result-level assessment: How 2021: overall survival (Kaplan–Meier curve available; HR not derived)	D1 Bias due to confounding	Serious	the starting dose has to be individualized by patient and practitioner	How et al. (2021) | PDF pp.8–9 | Discussion/limitations	Serious	Dose choice changed over calendar time and was clinician individualized; comparisons were unadjusted and important residual confounding remained.	Figure 3; complete result-level details retained in this table
How 2021	ROBINS-I 2016 2016 original cohort-type version (19 September 2016)	OS | Result-level assessment: How 2021: overall survival (Kaplan–Meier curve available; HR not derived)	D2 Bias in selection of participants into the study	Moderate	excluded ... loss to follow-up without any documentation	How et al. (2021) | PDF pp.3–4 | Methods/Patient population	Serious	Eligibility required treatment initiation, but patients without post-initiation outcome documentation could be excluded.	Figure 3; complete result-level details retained in this table
How 2021	ROBINS-I 2016 2016 original cohort-type version (19 September 2016)	OS | Result-level assessment: How 2021: overall survival (Kaplan–Meier curve available; HR not derived)	D3 Bias in classification of interventions	Low	lenvatinib starting dosage	How et al. (2021) | PDF pp.3–5 | Clinical data collection/Results	Serious	Starting dose was clearly defined and abstracted from contemporaneous electronic medical records.	Figure 3; complete result-level details retained in this table
How 2021	ROBINS-I 2016 2016 original cohort-type version (19 September 2016)	OS | Result-level assessment: How 2021: overall survival (Kaplan–Meier curve available; HR not derived)	D4 Bias due to deviations from intended interventions	Low	based on initial lenvatinib dosage level	How et al. (2021) | PDF p.4 | Statistical analysis	Serious	The prespecified estimand is assignment to the starting strategy; usual-practice later reductions and interruptions are outcomes rather than	Figure 3; complete result-level details retained in this table

Study	Tool	Outcome or result	Domain	Judgment	Support for judgment	Information source	Overall judgment	Additional rationale	Figure link
								protocol deviations.	
How 2021	ROBINS-I 2016 2016 original cohort-type version (19 September 2016)	OS | Result-level assessment: How 2021: overall survival (Kaplan–Meier curve available; HR not derived)	D5 Bias due to missing data	Low	OS was defined from treatment start until death	How et al. (2021) | PDF pp.4,6 | Outcome availability/missingness	Serious	Vital-status follow-up was reported for the cohort.	Figure 3; complete result-level details retained in this table
How 2021	ROBINS-I 2016 2016 original cohort-type version (19 September 2016)	OS | Result-level assessment: How 2021: overall survival (Kaplan–Meier curve available; HR not derived)	D6 Bias in measurement of outcomes	Low	OS was defined from treatment start until death	How et al. (2021) | PDF pp.4,6 | Outcome measurement	Serious	Death is an objective outcome and ascertainment methods did not differ by dose group.	Figure 3; complete result-level details retained in this table
How 2021	ROBINS-I 2016 2016 original cohort-type version (19 September 2016)	OS | Result-level assessment: How 2021: overall survival (Kaplan–Meier curve available; HR not derived)	D7 Bias in selection of the reported result	Moderate	Subgroup analyses were performed for ... toxicity, response, and survival	How et al. (2021) | PDF p.4 | Statistical analysis	Serious	No prespecified public analysis plan was located; multiple outcomes and subgroup analyses were available.	Figure 3; complete result-level details retained in this table
How 2021	ROBINS-I 2016 2016 original cohort-type version (19 September 2016)	OS | Result-level assessment: How 2021: overall survival (Kaplan–Meier curve available; HR not derived)	Overall Overall risk of bias	Serious	at least one domain was judged Serious	How et al. (2021) | See linked D1-D7 anchors | ROBINS-I overall algorithm	Serious	ROBINS-I 2016 overall judgment equals the highest domain when no domain is Critical.	Figure 3; complete result-level details retained in this table
How 2021	ROBINS-I 2016 2016 original cohort-type version (19 September 2016)	PFS | Result-level assessment: How 2021: progression-free survival (Kaplan–Meier curve available; HR not derived)	D1 Bias due to confounding	Serious	the starting dose has to be individualized by patient and practitioner	How et al. (2021) | PDF pp.8–9 | Discussion/limitations	Serious	Dose choice changed over calendar time and was clinician individualized; comparisons were unadjusted and important residual confounding remained.	Figure 3; complete result-level details retained in this table
How 2021	ROBINS-I 2016 2016 original cohort-type version (19 September 2016)	PFS | Result-level assessment: How 2021: progression-free survival (Kaplan–Meier curve available; HR not derived)	D2 Bias in selection of participants into the study	Moderate	excluded ... loss to follow-up without any documentation	How et al. (2021) | PDF pp.3–4 | Methods/Patient population	Serious	Eligibility required treatment initiation, but patients without post-initiation outcome documentation could be excluded.	Figure 3; complete result-level details retained in this table
How 2021	ROBINS-I 2016 2016 original cohort-type version (19 September 2016)	PFS | Result-level assessment: How 2021: progression-free survival (Kaplan–Meier curve available; HR not derived)	D3 Bias in classification of interventions	Low	lenvatinib starting dosage	How et al. (2021) | PDF pp.3–5 | Clinical data collection/Results	Serious	Starting dose was clearly defined and abstracted from contemporaneous electronic medical records.	Figure 3; complete result-level details retained in this table
How 2021	ROBINS-I 2016 2016 original cohort-type version (19 September 2016)	PFS | Result-level assessment: How 2021: progression-free survival (Kaplan–Meier curve available; HR not derived)	D4 Bias due to deviations from intended interventions	Low	based on initial lenvatinib dosage level	How et al. (2021) | PDF p.4 | Statistical analysis	Serious	The prespecified estimand is assignment to the starting strategy; usual-practice later reductions and interruptions are outcomes rather than protocol deviations.	Figure 3; complete result-level details retained in this table
How 2021	ROBINS-I 2016 2016 original cohort-type version (19 September 2016)	PFS | Result-level assessment: How 2021: progression-free survival (Kaplan–Meier curve available; HR not derived)	D5 Bias due to missing data	Low	PFS was defined from treatment start to progression or death	How et al. (2021) | PDF pp.4,6 | Outcome availability/missingness	Serious	Survival follow-up was reported for the cohort.	Figure 3; complete result-level details retained in this table
How 2021	ROBINS-I 2016 2016 original cohort-type version (19 September 2016)	PFS | Result-level assessment: How 2021: progression-free survival (Kaplan–Meier curve available; HR not derived)	D6 Bias in measurement of outcomes	Moderate	PFS was defined from treatment start to progression or death	How et al. (2021) | PDF pp.4,6 | Outcome measurement	Serious	Progression assessment was radiologic and unblinded without a stated uniform RECIST schedule.	Figure 3; complete result-level details retained in this table

Study	Tool	Outcome or result	Domain	Judgment	Support for judgment	Information source	Overall judgment	Additional rationale	Figure link
How 2021	ROBINS-I 2016 2016 original cohort-type version (19 September 2016)	PFS | Result-level assessment: How 2021: progression-free survival (Kaplan–Meier curve available; HR not derived)	D7 Bias in selection of the reported result	Moderate	Subgroup analyses were performed for ... toxicity, response, and survival	How et al. (2021) | PDF p.4 | Statistical analysis	Serious	No prespecified public analysis plan was located; multiple outcomes and subgroup analyses were available.	Figure 3; complete result-level details retained in this table
How 2021	ROBINS-I 2016 2016 original cohort-type version (19 September 2016)	PFS | Result-level assessment: How 2021: progression-free survival (Kaplan–Meier curve available; HR not derived)	Overall Overall risk of bias	Serious	at least one domain was judged Serious	How et al. (2021) | See linked D1-D7 anchors | ROBINS-I overall algorithm	Serious	ROBINS-I 2016 overall judgment equals the highest domain when no domain is Critical.	Figure 3; complete result-level details retained in this table
How 2021	ROBINS-I 2016 2016 original cohort-type version (19 September 2016)	How 2021: treatment discontinuation toxicity-attributed outcome definition	D1 Bias due to confounding	Serious	the starting dose has to be individualized by patient and practitioner	How et al. (2021) | PDF pp.8–9 | Discussion/limitations	Serious	Dose choice changed over calendar time and was clinician individualized; comparisons were unadjusted and important residual confounding remained.	Figure 3; complete result-level details retained in this table
How 2021	ROBINS-I 2016 2016 original cohort-type version (19 September 2016)	How 2021: treatment interruption (risk ratio)	D1 Bias due to confounding	Serious	the starting dose has to be individualized by patient and practitioner	How et al. (2021) | PDF pp.8–9 | Discussion/limitations	Serious	Dose choice changed over calendar time and was clinician individualized; comparisons were unadjusted and important residual confounding remained.	Figure 3; complete result-level details retained in this table
How 2021	ROBINS-I 2016 2016 original cohort-type version (19 September 2016)	How 2021: subsequent lenvatinib dose reduction (risk ratio)	D1 Bias due to confounding	Serious	the starting dose has to be individualized by patient and practitioner	How et al. (2021) | PDF pp.8–9 | Discussion/limitations	Serious	Dose choice changed over calendar time and was clinician individualized; comparisons were unadjusted and important residual confounding remained.	Figure 3; complete result-level details retained in this table
How 2021	ROBINS-I 2016 2016 original cohort-type version (19 September 2016)	How 2021: treatment discontinuation toxicity-attributed outcome definition	D2 Bias in selection of participants into the study	Moderate	excluded ... loss to follow-up without any documentation	How et al. (2021) | PDF pp.3–4 | Methods/Patient population	Serious	Eligibility required treatment initiation, but patients without post-initiation outcome documentation could be excluded.	Figure 3; complete result-level details retained in this table
How 2021	ROBINS-I 2016 2016 original cohort-type version (19 September 2016)	How 2021: treatment interruption (risk ratio)	D2 Bias in selection of participants into the study	Moderate	excluded ... loss to follow-up without any documentation	How et al. (2021) | PDF pp.3–4 | Methods/Patient population	Serious	Eligibility required treatment initiation, but patients without post-initiation outcome documentation could be excluded.	Figure 3; complete result-level details retained in this table
How 2021	ROBINS-I 2016 2016 original cohort-type version (19 September 2016)	How 2021: subsequent lenvatinib dose reduction (risk ratio)	D2 Bias in selection of participants into the study	Moderate	excluded ... loss to follow-up without any documentation	How et al. (2021) | PDF pp.3–4 | Methods/Patient population	Serious	Eligibility required treatment initiation, but patients without post-initiation outcome documentation could be excluded.	Figure 3; complete result-level details retained in this table
How 2021	ROBINS-I 2016 2016 original cohort-type version (19 September 2016)	How 2021: treatment discontinuation toxicity-attributed outcome definition	D3 Bias in classification of interventions	Low	lenvatinib starting dosage	How et al. (2021) | PDF pp.3–5 | Clinical data collection/Results	Serious	Starting dose was clearly defined and abstracted from contemporaneous electronic medical records.	Figure 3; complete result-level details retained in this table
How 2021	ROBINS-I 2016 2016 original cohort-type version (19 September 2016)	How 2021: treatment interruption (risk ratio)	D3 Bias in classification of interventions	Low	lenvatinib starting dosage	How et al. (2021) | PDF pp.3–5 | Clinical data collection/Results	Serious	Starting dose was clearly defined and abstracted from contemporaneous electronic medical records.	Figure 3; complete result-level details retained in this table
How 2021	ROBINS-I 2016 2016 original cohort-type version (19 September 2016)	How 2021: subsequent lenvatinib dose reduction (risk ratio)	D3 Bias in classification of interventions	Low	lenvatinib starting dosage	How et al. (2021) | PDF pp.3–5 | Clinical data collection/Results	Serious	Starting dose was clearly defined and abstracted from contemporaneous electronic medical records.	Figure 3; complete result-level details retained in this table

Study	Tool	Outcome or result	Domain	Judgment	Support for judgment	Information source	Overall judgment	Additional rationale	Figure link
How 2021	ROBINS-I 2016 2016 original cohort-type version (19 September 2016)	How 2021: treatment discontinuation toxicity-attributed outcome definition	D4 Bias due to deviations from intended interventions	Low	based on initial lenvatinib dosage level	How et al. (2021) | PDF p.4 | Statistical analysis	Serious	The prespecified estimand is assignment to the starting strategy; usual-practice later reductions and interruptions are outcomes rather than protocol deviations.	Figure 3; complete result-level details retained in this table
How 2021	ROBINS-I 2016 2016 original cohort-type version (19 September 2016)	How 2021: treatment interruption (risk ratio)	D4 Bias due to deviations from intended interventions	Low	based on initial lenvatinib dosage level	How et al. (2021) | PDF p.4 | Statistical analysis	Serious	The prespecified estimand is assignment to the starting strategy; usual-practice later reductions and interruptions are outcomes rather than protocol deviations.	Figure 3; complete result-level details retained in this table
How 2021	ROBINS-I 2016 2016 original cohort-type version (19 September 2016)	How 2021: subsequent lenvatinib dose reduction (risk ratio)	D4 Bias due to deviations from intended interventions	Low	based on initial lenvatinib dosage level	How et al. (2021) | PDF p.4 | Statistical analysis	Serious	The prespecified estimand is assignment to the starting strategy; usual-practice later reductions and interruptions are outcomes rather than protocol deviations.	Figure 3; complete result-level details retained in this table
How 2021	ROBINS-I 2016 2016 original cohort-type version (19 September 2016)	How 2021: treatment discontinuation toxicity-attributed outcome definition	D5 Bias due to missing data	Moderate	toxicity data may not be fully captured	How et al. (2021) | PDF pp.3–5,8 | Outcome availability/missingness	Serious	Retrospective records may incompletely capture toxicity-management outcomes.	Figure 3; complete result-level details retained in this table
How 2021	ROBINS-I 2016 2016 original cohort-type version (19 September 2016)	How 2021: treatment interruption (risk ratio)	D5 Bias due to missing data	Moderate	toxicity data may not be fully captured	How et al. (2021) | PDF pp.3–5,8 | Outcome availability/missingness	Serious	Retrospective records may incompletely capture toxicity-management outcomes.	Figure 3; complete result-level details retained in this table
How 2021	ROBINS-I 2016 2016 original cohort-type version (19 September 2016)	How 2021: subsequent lenvatinib dose reduction (risk ratio)	D5 Bias due to missing data	Moderate	toxicity data may not be fully captured	How et al. (2021) | PDF pp.3–5,8 | Outcome availability/missingness	Serious	Retrospective records may incompletely capture toxicity-management outcomes.	Figure 3; complete result-level details retained in this table
How 2021	ROBINS-I 2016 2016 original cohort-type version (19 September 2016)	How 2021: treatment discontinuation toxicity-attributed outcome definition	D6 Bias in measurement of outcomes	Serious	toxicity data may not be fully captured	How et al. (2021) | PDF pp.3–5,8 | Outcome measurement	Serious	Unblinded clinicians made dose-management and toxicity-attribution decisions that define these outcomes.	Figure 3; complete result-level details retained in this table
How 2021	ROBINS-I 2016 2016 original cohort-type version (19 September 2016)	How 2021: treatment interruption (risk ratio)	D6 Bias in measurement of outcomes	Serious	toxicity data may not be fully captured	How et al. (2021) | PDF pp.3–5,8 | Outcome measurement	Serious	Unblinded clinicians made dose-management and toxicity-attribution decisions that define these outcomes.	Figure 3; complete result-level details retained in this table
How 2021	ROBINS-I 2016 2016 original cohort-type version (19 September 2016)	How 2021: subsequent lenvatinib dose reduction (risk ratio)	D6 Bias in measurement of outcomes	Serious	toxicity data may not be fully captured	How et al. (2021) | PDF pp.3–5,8 | Outcome measurement	Serious	Unblinded clinicians made dose-management and toxicity-attribution decisions that define these outcomes.	Figure 3; complete result-level details retained in this table
How 2021	ROBINS-I 2016 2016 original cohort-type version (19 September 2016)	How 2021: treatment discontinuation toxicity-attributed outcome definition	D7 Bias in selection of the reported result	Moderate	Subgroup analyses were performed for ... toxicity, response, and survival	How et al. (2021) | PDF p.4 | Statistical analysis	Serious	No prespecified public analysis plan was located; multiple outcomes and subgroup analyses were available.	Figure 3; complete result-level details retained in this table
How 2021	ROBINS-I 2016 2016 original cohort-type version (19 September 2016)	How 2021: treatment interruption (risk ratio)	D7 Bias in selection of the reported result	Moderate	Subgroup analyses were performed for ... toxicity, response, and survival	How et al. (2021) | PDF p.4 | Statistical analysis	Serious	No prespecified public analysis plan was located; multiple outcomes and subgroup analyses were available.	Figure 3; complete result-level details retained in this table
How 2021	ROBINS-I 2016 2016 original cohort-type version (19 September 2016)	How 2021: subsequent lenvatinib dose reduction (risk ratio)	D7 Bias in selection of the reported result	Moderate	Subgroup analyses were performed for ... toxicity, response, and survival	How et al. (2021) | PDF p.4 | Statistical analysis	Serious	No prespecified public analysis plan was located; multiple outcomes and subgroup analyses were available.	Figure 3; complete result-level details retained in this table
How 2021	ROBINS-I 2016 2016	How 2021: treatment	Overall Overall risk of bias	Serious	at least one domain was	How et al. (2021) | See linked	Serious	ROBINS-I 2016 overall	Figure 3; complete result-

Study	Tool	Outcome or result	Domain	Judgment	Support for judgment	Information source	Overall judgment	Additional rationale	Figure link
	original cohort-type version (19 September 2016)	discontinuation toxicity-attributed outcome definition			judged Serious	D1-D7 anchors | ROBINS-I overall algorithm		judgment equals the highest domain when no domain is Critical.	level details retained in this table
How 2021	ROBINS-I 2016 2016 original cohort-type version (19 September 2016)	How 2021: treatment interruption (risk ratio)	Overall Overall risk of bias	Serious	at least one domain was judged Serious	How et al. (2021) | See linked D1-D7 anchors | ROBINS-I overall algorithm	Serious	ROBINS-I 2016 overall judgment equals the highest domain when no domain is Critical.	Figure 3; complete result-level details retained in this table
How 2021	ROBINS-I 2016 2016 original cohort-type version (19 September 2016)	How 2021: subsequent lenvatinib dose reduction (risk ratio)	Overall Overall risk of bias	Serious	at least one domain was judged Serious	How et al. (2021) | See linked D1-D7 anchors | ROBINS-I overall algorithm	Serious	ROBINS-I 2016 overall judgment equals the highest domain when no domain is Critical.	Figure 3; complete result-level details retained in this table
How 2021	ROBINS-I 2016 2016 original cohort-type version (19 September 2016)	How 2021: disease control (risk ratio)	D1 Bias due to confounding	Serious	the starting dose has to be individualized by patient and practitioner	How et al. (2021) | PDF pp.8–9 | Discussion/limitations	Serious	Dose choice changed over calendar time and was clinician individualized; comparisons were unadjusted and important residual confounding remained.	Figure 3; complete result-level details retained in this table
How 2021	ROBINS-I 2016 2016 original cohort-type version (19 September 2016)	How 2021: objective response (risk ratio)	D1 Bias due to confounding	Serious	the starting dose has to be individualized by patient and practitioner	How et al. (2021) | PDF pp.8–9 | Discussion/limitations	Serious	Dose choice changed over calendar time and was clinician individualized; comparisons were unadjusted and important residual confounding remained.	Figure 3; complete result-level details retained in this table
How 2021	ROBINS-I 2016 2016 original cohort-type version (19 September 2016)	How 2021: disease control (risk ratio)	D2 Bias in selection of participants into the study	Moderate	excluded ... loss to follow-up without any documentation	How et al. (2021) | PDF pp.3–4 | Methods/Patient population	Serious	Eligibility required treatment initiation, but patients without post-initiation outcome documentation could be excluded.	Figure 3; complete result-level details retained in this table
How 2021	ROBINS-I 2016 2016 original cohort-type version (19 September 2016)	How 2021: objective response (risk ratio)	D2 Bias in selection of participants into the study	Moderate	excluded ... loss to follow-up without any documentation	How et al. (2021) | PDF pp.3–4 | Methods/Patient population	Serious	Eligibility required treatment initiation, but patients without post-initiation outcome documentation could be excluded.	Figure 3; complete result-level details retained in this table
How 2021	ROBINS-I 2016 2016 original cohort-type version (19 September 2016)	How 2021: disease control (risk ratio)	D3 Bias in classification of interventions	Low	lenvatinib starting dosage	How et al. (2021) | PDF pp.3–5 | Clinical data collection/Results	Serious	Starting dose was clearly defined and abstracted from contemporaneous electronic medical records.	Figure 3; complete result-level details retained in this table
How 2021	ROBINS-I 2016 2016 original cohort-type version (19 September 2016)	How 2021: objective response (risk ratio)	D3 Bias in classification of interventions	Low	lenvatinib starting dosage	How et al. (2021) | PDF pp.3–5 | Clinical data collection/Results	Serious	Starting dose was clearly defined and abstracted from contemporaneous electronic medical records.	Figure 3; complete result-level details retained in this table
How 2021	ROBINS-I 2016 2016 original cohort-type version (19 September 2016)	How 2021: disease control (risk ratio)	D4 Bias due to deviations from intended interventions	Low	based on initial lenvatinib dosage level	How et al. (2021) | PDF p.4 | Statistical analysis	Serious	The prespecified estimand is assignment to the starting strategy; usual-practice later reductions and interruptions are outcomes rather than protocol deviations.	Figure 3; complete result-level details retained in this table
How 2021	ROBINS-I 2016 2016 original cohort-type version (19 September 2016)	How 2021: objective response (risk ratio)	D4 Bias due to deviations from intended interventions	Low	based on initial lenvatinib dosage level	How et al. (2021) | PDF p.4 | Statistical analysis	Serious	The prespecified estimand is assignment to the starting strategy; usual-practice later reductions and interruptions are outcomes rather than protocol deviations.	Figure 3; complete result-level details retained in this table
How 2021	ROBINS-I 2016 2016 original cohort-type version (19 September 2016)	How 2021: disease control (risk ratio)	D5 Bias due to missing data	Moderate	61 of 70 patients were evaluable for treatment response	How et al. (2021) | PDF pp.4–6 | Outcome availability/missingness	Serious	Similar missing-response proportions were present, but response was not available for all participants.	Figure 3; complete result-level details retained in this table

Study	Tool	Outcome or result	Domain	Judgment	Support for judgment	Information source	Overall judgment	Additional rationale	Figure link
How 2021	ROBINS-I 2016 2016 original cohort-type version (19 September 2016)	How 2021: objective response (risk ratio)	D5 Bias due to missing data	Moderate	61 of 70 patients were evaluable for treatment response	How et al. (2021) | PDF pp.4–6 | Outcome availability/missingness	Serious	Similar missing-response proportions were present, but response was not available for all participants.	Figure 3; complete result-level details retained in this table
How 2021	ROBINS-I 2016 2016 original cohort-type version (19 September 2016)	How 2021: disease control (risk ratio)	D6 Bias in measurement of outcomes	Moderate	61 of 70 patients were evaluable for treatment response	How et al. (2021) | PDF pp.4–6 | Outcome measurement	Serious	Radiologic response was used without a stated uniform RECIST/blinded central-review process.	Figure 3; complete result-level details retained in this table
How 2021	ROBINS-I 2016 2016 original cohort-type version (19 September 2016)	How 2021: objective response (risk ratio)	D6 Bias in measurement of outcomes	Moderate	61 of 70 patients were evaluable for treatment response	How et al. (2021) | PDF pp.4–6 | Outcome measurement	Serious	Radiologic response was used without a stated uniform RECIST/blinded central-review process.	Figure 3; complete result-level details retained in this table
How 2021	ROBINS-I 2016 2016 original cohort-type version (19 September 2016)	How 2021: disease control (risk ratio)	D7 Bias in selection of the reported result	Moderate	Subgroup analyses were performed for ... toxicity, response, and survival	How et al. (2021) | PDF p.4 | Statistical analysis	Serious	No prespecified public analysis plan was located; multiple outcomes and subgroup analyses were available.	Figure 3; complete result-level details retained in this table
How 2021	ROBINS-I 2016 2016 original cohort-type version (19 September 2016)	How 2021: objective response (risk ratio)	D7 Bias in selection of the reported result	Moderate	Subgroup analyses were performed for ... toxicity, response, and survival	How et al. (2021) | PDF p.4 | Statistical analysis	Serious	No prespecified public analysis plan was located; multiple outcomes and subgroup analyses were available.	Figure 3; complete result-level details retained in this table
How 2021	ROBINS-I 2016 2016 original cohort-type version (19 September 2016)	How 2021: disease control (risk ratio)	Overall Overall risk of bias	Serious	at least one domain was judged Serious	How et al. (2021) | See linked D1-D7 anchors | ROBINS-I overall algorithm	Serious	ROBINS-I 2016 overall judgment equals the highest domain when no domain is Critical.	Figure 3; complete result-level details retained in this table
How 2021	ROBINS-I 2016 2016 original cohort-type version (19 September 2016)	How 2021: objective response (risk ratio)	Overall Overall risk of bias	Serious	at least one domain was judged Serious	How et al. (2021) | See linked D1-D7 anchors | ROBINS-I overall algorithm	Serious	ROBINS-I 2016 overall judgment equals the highest domain when no domain is Critical.	Figure 3; complete result-level details retained in this table
Zammarrelli 2023	ROBINS-I 2016 2016 original cohort-type version (19 September 2016)	Zammarrelli 2023: grade 3 or higher adverse events (risk ratio)	D1 Bias due to confounding	Serious	patients were older and received more lines of treatment	Zammarrelli et al. (2023) | PDF pp.7–9 | Results/Discussion/limitations	Serious	Reduced-dose recipients were older and more heavily treated; analyses of dose groups were unadjusted and reasons for reduction were incompletely captured.	Figure 3; complete result-level details retained in this table
Zammarrelli 2023	ROBINS-I 2016 2016 original cohort-type version (19 September 2016)	Zammarrelli 2023: grade 3 or higher adverse events (risk ratio)	D2 Bias in selection of participants into the study	Low	43 patients included for analysis	Zammarrelli et al. (2023) | PDF pp.4–5; Supplementary Figure S1 | Patient population/flow diagram	Serious	Consensus rationale: The supplementary flow shows exclusions for miscoding, pembrolizumab-only treatment, or trial treatment; follow-up began at combination initiation.	Figure 3; complete result-level details retained in this table
Zammarrelli 2023	ROBINS-I 2016 2016 original cohort-type version (19 September 2016)	Zammarrelli 2023: grade 3 or higher adverse events (risk ratio)	D3 Bias in classification of interventions	Low	starting doses ... collected from chemotherapy logs	Zammarrelli et al. (2023) | PDF pp.4–5 | Clinical data/Treatment characteristics	Serious	Dose at initiation was clearly recorded in chemotherapy logs and physician documentation.	Figure 3; complete result-level details retained in this table
Zammarrelli 2023	ROBINS-I 2016 2016 original cohort-type version (19 September 2016)	Zammarrelli 2023: grade 3 or higher adverse events (risk ratio)	D4 Bias due to deviations from intended interventions	Low	primary dose reduction ... starting dose less than the 20 mg standard dose	Zammarrelli et al. (2023) | PDF pp.4–7 | Methods/Results	Serious	The effect of assignment to baseline dose was analyzed; routine later modifications were not used to reclassify exposure.	Figure 3; complete result-level details retained in this table
Zammarrelli 2023	ROBINS-I 2016 2016 original cohort-type version (19 September 2016)	Zammarrelli 2023: grade 3 or higher adverse events (risk ratio)	D5 Bias due to missing data	Moderate	AEs were graded using CTCAE version 5.0	Zammarrelli et al. (2023) | PDF pp.4,6–7 | Outcome availability/missingness	Serious	Outcome availability was not explicitly quantified by dose for every AE.	Figure 3; complete result-level details retained in this table
Zammarrelli	ROBINS-I 2016 2016	Zammarrelli 2023: grade	D6 Bias in measurement of	Moderate	AEs were graded using	Zammarrelli et al. (2023) |	Serious	CTCAE, laboratory, vital-	Figure 3; complete result-

Study	Tool	Outcome or result	Domain	Judgment	Support for judgment	Information source	Overall judgment	Additional rationale	Figure link
2023	original cohort-type version (19 September 2016)	3 or higher adverse events (risk ratio)	outcomes		CTCAE version 5.0	PDF pp.4,6–7 | Outcome measurement		sign and physician records were used, but retrospective unblinded capture remains.	level details retained in this table
Zammarrelli 2023	ROBINS-I 2016 2016 original cohort-type version (19 September 2016)	Zammarrelli 2023: grade 3 or higher adverse events (risk ratio)	D7 Bias in selection of the reported result	Moderate	log-rank test was used to compare cohorts	Zammarrelli et al. (2023) | PDF pp.4–5 | Statistical analysis	Serious	No public protocol or statistical analysis plan for the retrospective dose comparison was located.	Figure 3; complete result-level details retained in this table
Zammarrelli 2023	ROBINS-I 2016 2016 original cohort-type version (19 September 2016)	Zammarrelli 2023: grade 3 or higher adverse events (risk ratio)	Overall Overall risk of bias	Serious	at least one domain was judged Serious	Zammarrelli et al. (2023) | See linked D1-D7 anchors | ROBINS-I overall algorithm	Serious	ROBINS-I 2016 overall judgment equals the highest domain when no domain is Critical.	Figure 3; complete result-level details retained in this table
Zammarrelli 2023	ROBINS-I 2016 2016 original cohort-type version (19 September 2016)	Zammarrelli 2023: overall survival (reported unadjusted HR)	D1 Bias due to confounding	Serious	patients were older and received more lines of treatment	Zammarrelli et al. (2023) | PDF pp.7–9 | Results/Discussion/limitations	Serious	Reduced-dose recipients were older and more heavily treated; analyses of dose groups were unadjusted and reasons for reduction were incompletely captured.	Figure 3; complete result-level details retained in this table
Zammarrelli 2023	ROBINS-I 2016 2016 original cohort-type version (19 September 2016)	Zammarrelli 2023: overall survival (reported unadjusted HR)	D2 Bias in selection of participants into the study	Low	43 patients included for analysis	Zammarrelli et al. (2023) | PDF pp.4–5; Supplementary Figure S1 | Patient population/flow diagram	Serious	Consensus rationale: The supplementary flow shows exclusions for miscoding, pembrolizumab-only treatment, or trial treatment; follow-up began at combination initiation.	Figure 3; complete result-level details retained in this table
Zammarrelli 2023	ROBINS-I 2016 2016 original cohort-type version (19 September 2016)	Zammarrelli 2023: overall survival (reported unadjusted HR)	D3 Bias in classification of interventions	Low	starting doses ... collected from chemotherapy logs	Zammarrelli et al. (2023) | PDF pp.4–5 | Clinical data/Treatment characteristics	Serious	Dose at initiation was clearly recorded in chemotherapy logs and physician documentation.	Figure 3; complete result-level details retained in this table
Zammarrelli 2023	ROBINS-I 2016 2016 original cohort-type version (19 September 2016)	Zammarrelli 2023: overall survival (reported unadjusted HR)	D4 Bias due to deviations from intended interventions	Low	primary dose reduction ... starting dose less than the 20 mg standard dose	Zammarrelli et al. (2023) | PDF pp.4–7 | Methods/Results	Serious	The effect of assignment to baseline dose was analyzed; routine later modifications were not used to reclassify exposure.	Figure 3; complete result-level details retained in this table
Zammarrelli 2023	ROBINS-I 2016 2016 original cohort-type version (19 September 2016)	Zammarrelli 2023: overall survival (reported unadjusted HR)	D5 Bias due to missing data	Low	OS ... treatment initiation ... death or last follow-up	Zammarrelli et al. (2023) | PDF pp.4–7 | Outcome availability/missingness	Serious	Vital-status follow-up was reported.	Figure 3; complete result-level details retained in this table
Zammarrelli 2023	ROBINS-I 2016 2016 original cohort-type version (19 September 2016)	Zammarrelli 2023: overall survival (reported unadjusted HR)	D6 Bias in measurement of outcomes	Low	OS ... treatment initiation ... death or last follow-up	Zammarrelli et al. (2023) | PDF pp.4–7 | Outcome measurement	Serious	Death is objective and ascertainment was comparable across groups.	Figure 3; complete result-level details retained in this table
Zammarrelli 2023	ROBINS-I 2016 2016 original cohort-type version (19 September 2016)	Zammarrelli 2023: overall survival (reported unadjusted HR)	D7 Bias in selection of the reported result	Moderate	log-rank test was used to compare cohorts	Zammarrelli et al. (2023) | PDF pp.4–5 | Statistical analysis	Serious	No public protocol or statistical analysis plan for the retrospective dose comparison was located.	Figure 3; complete result-level details retained in this table
Zammarrelli 2023	ROBINS-I 2016 2016 original cohort-type version (19 September 2016)	Zammarrelli 2023: overall survival (reported unadjusted HR)	Overall Overall risk of bias	Serious	at least one domain was judged Serious	Zammarrelli et al. (2023) | See linked D1-D7 anchors | ROBINS-I overall algorithm	Serious	ROBINS-I 2016 overall judgment equals the highest domain when no domain is Critical.	Figure 3; complete result-level details retained in this table
Zammarrelli 2023	ROBINS-I 2016 2016 original cohort-type version (19 September 2016)	Zammarrelli 2023: progression-free survival (reported unadjusted HR)	D1 Bias due to confounding	Serious	patients were older and received more lines of treatment	Zammarrelli et al. (2023) | PDF pp.7–9 | Results/Discussion/limitations	Serious	Reduced-dose recipients were older and more heavily treated; analyses of dose groups were unadjusted and reasons for reduction were incompletely captured.	Figure 3; complete result-level details retained in this table
Zammarrelli 2023	ROBINS-I 2016 2016 original cohort-type version (19 September 2016)	Zammarrelli 2023: progression-free survival (reported unadjusted HR)	D2 Bias in selection of participants into the study	Low	43 patients included for analysis	Zammarrelli et al. (2023) | PDF pp.4–5; Supplementary Figure S1 | Patient	Serious	Consensus rationale: The supplementary flow shows exclusions for miscoding,	Figure 3; complete result-level details retained in this table

Study	Tool	Outcome or result	Domain	Judgment	Support for judgment	Information source	Overall judgment	Additional rationale	Figure link
						population/flow diagram		pembrolizumab-only treatment, or trial treatment; follow-up began at combination initiation.	
Zammarrelli 2023	ROBINS-I 2016 2016 original cohort-type version (19 September 2016)	Zammarrelli 2023: progression-free survival (reported unadjusted HR)	D3 Bias in classification of interventions	Low	starting doses ... collected from chemotherapy logs	Zammarrelli et al. (2023) | PDF pp.4–5 | Clinical data/Treatment characteristics	Serious	Dose at initiation was clearly recorded in chemotherapy logs and physician documentation.	Figure 3; complete result-level details retained in this table
Zammarrelli 2023	ROBINS-I 2016 2016 original cohort-type version (19 September 2016)	Zammarrelli 2023: progression-free survival (reported unadjusted HR)	D4 Bias due to deviations from intended interventions	Low	primary dose reduction ... starting dose less than the 20 mg standard dose	Zammarrelli et al. (2023) | PDF pp.4–7 | Methods/Results	Serious	The effect of assignment to baseline dose was analyzed; routine later modifications were not used to reclassify exposure.	Figure 3; complete result-level details retained in this table
Zammarrelli 2023	ROBINS-I 2016 2016 original cohort-type version (19 September 2016)	Zammarrelli 2023: progression-free survival (reported unadjusted HR)	D5 Bias due to missing data	Low	PFS ... treatment initiation ... progression, death, or last follow-up	Zammarrelli et al. (2023) | PDF pp.4–7 | Outcome availability/missingness	Serious	PFS follow-up was available for the cohort.	Figure 3; complete result-level details retained in this table
Zammarrelli 2023	ROBINS-I 2016 2016 original cohort-type version (19 September 2016)	Zammarrelli 2023: progression-free survival (reported unadjusted HR)	D6 Bias in measurement of outcomes	Moderate	PFS ... treatment initiation ... progression, death, or last follow-up	Zammarrelli et al. (2023) | PDF pp.4–7 | Outcome measurement	Serious	RECIST imaging was reviewed consistently, but dose blinding was not reported.	Figure 3; complete result-level details retained in this table
Zammarrelli 2023	ROBINS-I 2016 2016 original cohort-type version (19 September 2016)	Zammarrelli 2023: progression-free survival (reported unadjusted HR)	D7 Bias in selection of the reported result	Moderate	log-rank test was used to compare cohorts	Zammarrelli et al. (2023) | PDF pp.4–5 | Statistical analysis	Serious	No public protocol or statistical analysis plan for the retrospective dose comparison was located.	Figure 3; complete result-level details retained in this table
Zammarrelli 2023	ROBINS-I 2016 2016 original cohort-type version (19 September 2016)	Zammarrelli 2023: progression-free survival (reported unadjusted HR)	Overall Overall risk of bias	Serious	at least one domain was judged Serious	Zammarrelli et al. (2023) | See linked D1-D7 anchors | ROBINS-I overall algorithm	Serious	ROBINS-I 2016 overall judgment equals the highest domain when no domain is Critical.	Figure 3; complete result-level details retained in this table
Zammarrelli 2023	ROBINS-I 2016 2016 original cohort-type version (19 September 2016)	Zammarrelli 2023: treatment discontinuation toxicity-attributed outcome definition	D1 Bias due to confounding	Serious	patients were older and received more lines of treatment	Zammarrelli et al. (2023) | PDF pp.7–9 | Results/Discussion/limitations	Serious	Reduced-dose recipients were older and more heavily treated; analyses of dose groups were unadjusted and reasons for reduction were incompletely captured.	Figure 3; complete result-level details retained in this table
Zammarrelli 2023	ROBINS-I 2016 2016 original cohort-type version (19 September 2016)	Zammarrelli 2023: treatment interruption (risk ratio)	D1 Bias due to confounding	Serious	patients were older and received more lines of treatment	Zammarrelli et al. (2023) | PDF pp.7–9 | Results/Discussion/limitations	Serious	Reduced-dose recipients were older and more heavily treated; analyses of dose groups were unadjusted and reasons for reduction were incompletely captured.	Figure 3; complete result-level details retained in this table
Zammarrelli 2023	ROBINS-I 2016 2016 original cohort-type version (19 September 2016)	Zammarrelli 2023: subsequent lenvatinib dose reduction (risk ratio)	D1 Bias due to confounding	Serious	patients were older and received more lines of treatment	Zammarrelli et al. (2023) | PDF pp.7–9 | Results/Discussion/limitations	Serious	Reduced-dose recipients were older and more heavily treated; analyses of dose groups were unadjusted and reasons for reduction were incompletely captured.	Figure 3; complete result-level details retained in this table
Zammarrelli 2023	ROBINS-I 2016 2016 original cohort-type version (19 September 2016)	Zammarrelli 2023: treatment discontinuation toxicity-attributed outcome definition	D2 Bias in selection of participants into the study	Low	43 patients included for analysis	Zammarrelli et al. (2023) | PDF pp.4–5; Supplementary Figure S1 | Patient population/flow diagram	Serious	Consensus rationale: The supplementary flow shows exclusions for miscoding, pembrolizumab-only treatment, or trial treatment; follow-up began at combination initiation.	Figure 3; complete result-level details retained in this table
Zammarrelli 2023	ROBINS-I 2016 2016 original cohort-type version (19 September 2016)	Zammarrelli 2023: treatment interruption (risk ratio)	D2 Bias in selection of participants into the study	Low	43 patients included for analysis	Zammarrelli et al. (2023) | PDF pp.4–5; Supplementary Figure S1 | Patient population/flow diagram	Serious	Consensus rationale: The supplementary flow shows exclusions for miscoding, pembrolizumab-only	Figure 3; complete result-level details retained in this table

Study	Tool	Outcome or result	Domain	Judgment	Support for judgment	Information source	Overall judgment	Additional rationale	Figure link
								treatment, or trial treatment; follow-up began at combination initiation.	
Zammarrelli 2023	ROBINS-I 2016 2016 original cohort-type version (19 September 2016)	Zammarrelli 2023: subsequent lenvatinib dose reduction (risk ratio)	D2 Bias in selection of participants into the study	Low	43 patients included for analysis	Zammarrelli et al. (2023) | PDF pp.4–5; Supplementary Figure S1 | Patient population/flow diagram	Serious	Consensus rationale: The supplementary flow shows exclusions for miscoding, pembrolizumab-only treatment, or trial treatment; follow-up began at combination initiation.	Figure 3; complete result-level details retained in this table
Zammarrelli 2023	ROBINS-I 2016 2016 original cohort-type version (19 September 2016)	Zammarrelli 2023: treatment discontinuation toxicity-attributed outcome definition	D3 Bias in classification of interventions	Low	starting doses ... collected from chemotherapy logs	Zammarrelli et al. (2023) | PDF pp.4–5 | Clinical data/Treatment characteristics	Serious	Dose at initiation was clearly recorded in chemotherapy logs and physician documentation.	Figure 3; complete result-level details retained in this table
Zammarrelli 2023	ROBINS-I 2016 2016 original cohort-type version (19 September 2016)	Zammarrelli 2023: treatment interruption (risk ratio)	D3 Bias in classification of interventions	Low	starting doses ... collected from chemotherapy logs	Zammarrelli et al. (2023) | PDF pp.4–5 | Clinical data/Treatment characteristics	Serious	Dose at initiation was clearly recorded in chemotherapy logs and physician documentation.	Figure 3; complete result-level details retained in this table
Zammarrelli 2023	ROBINS-I 2016 2016 original cohort-type version (19 September 2016)	Zammarrelli 2023: subsequent lenvatinib dose reduction (risk ratio)	D3 Bias in classification of interventions	Low	starting doses ... collected from chemotherapy logs	Zammarrelli et al. (2023) | PDF pp.4–5 | Clinical data/Treatment characteristics	Serious	Dose at initiation was clearly recorded in chemotherapy logs and physician documentation.	Figure 3; complete result-level details retained in this table
Zammarrelli 2023	ROBINS-I 2016 2016 original cohort-type version (19 September 2016)	Zammarrelli 2023: treatment discontinuation toxicity-attributed outcome definition	D4 Bias due to deviations from intended interventions	Low	primary dose reduction ... starting dose less than the 20 mg standard dose	Zammarrelli et al. (2023) | PDF pp.4–7 | Methods/Results	Serious	The effect of assignment to baseline dose was analyzed; routine later modifications were not used to reclassify exposure.	Figure 3; complete result-level details retained in this table
Zammarrelli 2023	ROBINS-I 2016 2016 original cohort-type version (19 September 2016)	Zammarrelli 2023: treatment interruption (risk ratio)	D4 Bias due to deviations from intended interventions	Low	primary dose reduction ... starting dose less than the 20 mg standard dose	Zammarrelli et al. (2023) | PDF pp.4–7 | Methods/Results	Serious	The effect of assignment to baseline dose was analyzed; routine later modifications were not used to reclassify exposure.	Figure 3; complete result-level details retained in this table
Zammarrelli 2023	ROBINS-I 2016 2016 original cohort-type version (19 September 2016)	Zammarrelli 2023: subsequent lenvatinib dose reduction (risk ratio)	D4 Bias due to deviations from intended interventions	Low	primary dose reduction ... starting dose less than the 20 mg standard dose	Zammarrelli et al. (2023) | PDF pp.4–7 | Methods/Results	Serious	The effect of assignment to baseline dose was analyzed; routine later modifications were not used to reclassify exposure.	Figure 3; complete result-level details retained in this table
Zammarrelli 2023	ROBINS-I 2016 2016 original cohort-type version (19 September 2016)	Zammarrelli 2023: treatment discontinuation toxicity-attributed outcome definition	D5 Bias due to missing data	Moderate	collected from chemotherapy logs and physician documentation	Zammarrelli et al. (2023) | PDF pp.4,6–7 | Outcome availability/missingness	Serious	Records supported the endpoints, but completeness was not formally demonstrated.	Figure 3; complete result-level details retained in this table
Zammarrelli 2023	ROBINS-I 2016 2016 original cohort-type version (19 September 2016)	Zammarrelli 2023: treatment interruption (risk ratio)	D5 Bias due to missing data	Moderate	collected from chemotherapy logs and physician documentation	Zammarrelli et al. (2023) | PDF pp.4,6–7 | Outcome availability/missingness	Serious	Records supported the endpoints, but completeness was not formally demonstrated.	Figure 3; complete result-level details retained in this table
Zammarrelli 2023	ROBINS-I 2016 2016 original cohort-type version (19 September 2016)	Zammarrelli 2023: subsequent lenvatinib dose reduction (risk ratio)	D5 Bias due to missing data	Moderate	collected from chemotherapy logs and physician documentation	Zammarrelli et al. (2023) | PDF pp.4,6–7 | Outcome availability/missingness	Serious	Records supported the endpoints, but completeness was not formally demonstrated.	Figure 3; complete result-level details retained in this table
Zammarrelli 2023	ROBINS-I 2016 2016 original cohort-type version (19 September 2016)	Zammarrelli 2023: treatment discontinuation toxicity-attributed outcome definition	D6 Bias in measurement of outcomes	Serious	collected from chemotherapy logs and physician documentation	Zammarrelli et al. (2023) | PDF pp.4,6–7 | Outcome measurement	Serious	Clinical management and toxicity attribution were unblinded and could differ by starting dose or perceived frailty.	Figure 3; complete result-level details retained in this table
Zammarrelli 2023	ROBINS-I 2016 2016 original cohort-type version	Zammarrelli 2023: treatment interruption	D6 Bias in measurement of outcomes	Serious	collected from chemotherapy logs and physician	Zammarrelli et al. (2023) | PDF pp.4,6–7 | Outcome	Serious	Clinical management and toxicity attribution were	Figure 3; complete result-level details retained in this

Study	Tool	Outcome or result	Domain	Judgment	Support for judgment	Information source	Overall judgment	Additional rationale	Figure link
	(19 September 2016)	(risk ratio)			documentation	measurement		unblinded and could differ by starting dose or perceived frailty.	table
Zammarrelli 2023	ROBINS-I 2016 2016 original cohort-type version (19 September 2016)	Zammarrelli 2023: subsequent lenvatinib dose reduction (risk ratio)	D6 Bias in measurement of outcomes	Serious	collected from chemotherapy logs and physician documentation	Zammarrelli et al. (2023) | PDF pp.4,6–7 | Outcome measurement	Serious	Clinical management and toxicity attribution were unblinded and could differ by starting dose or perceived frailty.	Figure 3; complete result-level details retained in this table
Zammarrelli 2023	ROBINS-I 2016 2016 original cohort-type version (19 September 2016)	Zammarrelli 2023: treatment discontinuation toxicity-attributed outcome definition	D7 Bias in selection of the reported result	Moderate	log-rank test was used to compare cohorts	Zammarrelli et al. (2023) | PDF pp.4–5 | Statistical analysis	Serious	No public protocol or statistical analysis plan for the retrospective dose comparison was located.	Figure 3; complete result-level details retained in this table
Zammarrelli 2023	ROBINS-I 2016 2016 original cohort-type version (19 September 2016)	Zammarrelli 2023: treatment interruption (risk ratio)	D7 Bias in selection of the reported result	Moderate	log-rank test was used to compare cohorts	Zammarrelli et al. (2023) | PDF pp.4–5 | Statistical analysis	Serious	No public protocol or statistical analysis plan for the retrospective dose comparison was located.	Figure 3; complete result-level details retained in this table
Zammarrelli 2023	ROBINS-I 2016 2016 original cohort-type version (19 September 2016)	Zammarrelli 2023: subsequent lenvatinib dose reduction (risk ratio)	D7 Bias in selection of the reported result	Moderate	log-rank test was used to compare cohorts	Zammarrelli et al. (2023) | PDF pp.4–5 | Statistical analysis	Serious	No public protocol or statistical analysis plan for the retrospective dose comparison was located.	Figure 3; complete result-level details retained in this table
Zammarrelli 2023	ROBINS-I 2016 2016 original cohort-type version (19 September 2016)	Zammarrelli 2023: treatment discontinuation toxicity-attributed outcome definition	Overall Overall risk of bias	Serious	at least one domain was judged Serious	Zammarrelli et al. (2023) | See linked D1-D7 anchors | ROBINS-I overall algorithm	Serious	ROBINS-I 2016 overall judgment equals the highest domain when no domain is Critical.	Figure 3; complete result-level details retained in this table
Zammarrelli 2023	ROBINS-I 2016 2016 original cohort-type version (19 September 2016)	Zammarrelli 2023: treatment interruption (risk ratio)	Overall Overall risk of bias	Serious	at least one domain was judged Serious	Zammarrelli et al. (2023) | See linked D1-D7 anchors | ROBINS-I overall algorithm	Serious	ROBINS-I 2016 overall judgment equals the highest domain when no domain is Critical.	Figure 3; complete result-level details retained in this table
Zammarrelli 2023	ROBINS-I 2016 2016 original cohort-type version (19 September 2016)	Zammarrelli 2023: subsequent lenvatinib dose reduction (risk ratio)	Overall Overall risk of bias	Serious	at least one domain was judged Serious	Zammarrelli et al. (2023) | See linked D1-D7 anchors | ROBINS-I overall algorithm	Serious	ROBINS-I 2016 overall judgment equals the highest domain when no domain is Critical.	Figure 3; complete result-level details retained in this table
Zammarrelli 2023	ROBINS-I 2016 2016 original cohort-type version (19 September 2016)	Zammarrelli 2023: disease control (risk ratio)	D1 Bias due to confounding	Serious	patients were older and received more lines of treatment	Zammarrelli et al. (2023) | PDF pp.7–9 | Results/Discussion/limitations	Serious	Reduced-dose recipients were older and more heavily treated; analyses of dose groups were unadjusted and reasons for reduction were incompletely captured.	Figure 3; complete result-level details retained in this table
Zammarrelli 2023	ROBINS-I 2016 2016 original cohort-type version (19 September 2016)	Zammarrelli 2023: objective response (risk ratio)	D1 Bias due to confounding	Serious	patients were older and received more lines of treatment	Zammarrelli et al. (2023) | PDF pp.7–9 | Results/Discussion/limitations	Serious	Reduced-dose recipients were older and more heavily treated; analyses of dose groups were unadjusted and reasons for reduction were incompletely captured.	Figure 3; complete result-level details retained in this table
Zammarrelli 2023	ROBINS-I 2016 2016 original cohort-type version (19 September 2016)	Zammarrelli 2023: disease control (risk ratio)	D2 Bias in selection of participants into the study	Low	43 patients included for analysis	Zammarrelli et al. (2023) | PDF pp.4–5; Supplementary Figure S1 | Patient population/flow diagram	Serious	Consensus rationale: The supplementary flow shows exclusions for miscoding, pembrolizumab-only treatment, or trial treatment; follow-up began at combination initiation.	Figure 3; complete result-level details retained in this table
Zammarrelli 2023	ROBINS-I 2016 2016 original cohort-type version (19 September 2016)	Zammarrelli 2023: objective response (risk ratio)	D2 Bias in selection of participants into the study	Low	43 patients included for analysis	Zammarrelli et al. (2023) | PDF pp.4–5; Supplementary Figure S1 | Patient population/flow diagram	Serious	Consensus rationale: The supplementary flow shows exclusions for miscoding, pembrolizumab-only treatment, or trial treatment; follow-up began at	Figure 3; complete result-level details retained in this table

Study	Tool	Outcome or result	Domain	Judgment	Support for judgment	Information source	Overall judgment	Additional rationale	Figure link
								combination initiation.	
Zammarrelli 2023	ROBINS-I 2016 2016 original cohort-type version (19 September 2016)	Zammarrelli 2023: disease control (risk ratio)	D3 Bias in classification of interventions	Low	starting doses ... collected from chemotherapy logs	Zammarrelli et al. (2023) | PDF pp.4–5 | Clinical data/Treatment characteristics	Serious	Dose at initiation was clearly recorded in chemotherapy logs and physician documentation.	Figure 3; complete result-level details retained in this table
Zammarrelli 2023	ROBINS-I 2016 2016 original cohort-type version (19 September 2016)	Zammarrelli 2023: objective response (risk ratio)	D3 Bias in classification of interventions	Low	starting doses ... collected from chemotherapy logs	Zammarrelli et al. (2023) | PDF pp.4–5 | Clinical data/Treatment characteristics	Serious	Dose at initiation was clearly recorded in chemotherapy logs and physician documentation.	Figure 3; complete result-level details retained in this table
Zammarrelli 2023	ROBINS-I 2016 2016 original cohort-type version (19 September 2016)	Zammarrelli 2023: disease control (risk ratio)	D4 Bias due to deviations from intended interventions	Low	primary dose reduction ... starting dose less than the 20 mg standard dose	Zammarrelli et al. (2023) | PDF pp.4–7 | Methods/Results	Serious	The effect of assignment to baseline dose was analyzed; routine later modifications were not used to reclassify exposure.	Figure 3; complete result-level details retained in this table
Zammarrelli 2023	ROBINS-I 2016 2016 original cohort-type version (19 September 2016)	Zammarrelli 2023: objective response (risk ratio)	D4 Bias due to deviations from intended interventions	Low	primary dose reduction ... starting dose less than the 20 mg standard dose	Zammarrelli et al. (2023) | PDF pp.4–7 | Methods/Results	Serious	The effect of assignment to baseline dose was analyzed; routine later modifications were not used to reclassify exposure.	Figure 3; complete result-level details retained in this table
Zammarrelli 2023	ROBINS-I 2016 2016 original cohort-type version (19 September 2016)	Zammarrelli 2023: disease control (risk ratio)	D5 Bias due to missing data	Moderate	41 were evaluable for treatment response	Zammarrelli et al. (2023) | PDF pp.4,6 | Outcome availability/missingness	Serious	Two standard-dose participants lacked evaluable response.	Figure 3; complete result-level details retained in this table
Zammarrelli 2023	ROBINS-I 2016 2016 original cohort-type version (19 September 2016)	Zammarrelli 2023: objective response (risk ratio)	D5 Bias due to missing data	Moderate	41 were evaluable for treatment response	Zammarrelli et al. (2023) | PDF pp.4,6 | Outcome availability/missingness	Serious	Two standard-dose participants lacked evaluable response.	Figure 3; complete result-level details retained in this table
Zammarrelli 2023	ROBINS-I 2016 2016 original cohort-type version (19 September 2016)	Zammarrelli 2023: disease control (risk ratio)	D6 Bias in measurement of outcomes	Moderate	41 were evaluable for treatment response	Zammarrelli et al. (2023) | PDF pp.4,6 | Outcome measurement	Serious	A dedicated radiologist used RECIST 1.1, but blinding to dose was not reported.	Figure 3; complete result-level details retained in this table
Zammarrelli 2023	ROBINS-I 2016 2016 original cohort-type version (19 September 2016)	Zammarrelli 2023: objective response (risk ratio)	D6 Bias in measurement of outcomes	Moderate	41 were evaluable for treatment response	Zammarrelli et al. (2023) | PDF pp.4,6 | Outcome measurement	Serious	A dedicated radiologist used RECIST 1.1, but blinding to dose was not reported.	Figure 3; complete result-level details retained in this table
Zammarrelli 2023	ROBINS-I 2016 2016 original cohort-type version (19 September 2016)	Zammarrelli 2023: disease control (risk ratio)	D7 Bias in selection of the reported result	Moderate	log-rank test was used to compare cohorts	Zammarrelli et al. (2023) | PDF pp.4–5 | Statistical analysis	Serious	No public protocol or statistical analysis plan for the retrospective dose comparison was located.	Figure 3; complete result-level details retained in this table
Zammarrelli 2023	ROBINS-I 2016 2016 original cohort-type version (19 September 2016)	Zammarrelli 2023: objective response (risk ratio)	D7 Bias in selection of the reported result	Moderate	log-rank test was used to compare cohorts	Zammarrelli et al. (2023) | PDF pp.4–5 | Statistical analysis	Serious	No public protocol or statistical analysis plan for the retrospective dose comparison was located.	Figure 3; complete result-level details retained in this table
Zammarrelli 2023	ROBINS-I 2016 2016 original cohort-type version (19 September 2016)	Zammarrelli 2023: disease control (risk ratio)	Overall Overall risk of bias	Serious	at least one domain was judged Serious	Zammarrelli et al. (2023) | See linked D1-D7 anchors | ROBINS-I overall algorithm	Serious	ROBINS-I 2016 overall judgment equals the highest domain when no domain is Critical.	Figure 3; complete result-level details retained in this table
Zammarrelli 2023	ROBINS-I 2016 2016 original cohort-type version (19 September 2016)	Zammarrelli 2023: objective response (risk ratio)	Overall Overall risk of bias	Serious	at least one domain was judged Serious	Zammarrelli et al. (2023) | See linked D1-D7 anchors | ROBINS-I overall algorithm	Serious	ROBINS-I 2016 overall judgment equals the highest domain when no domain is Critical.	Figure 3; complete result-level details retained in this table
Ta 2025	ROBINS-I 2016 2016 original cohort-type version (19 September 2016)	Ta 2025: hospitalization all-cause outcome definition	D1 Bias due to confounding	Serious	lower starting dose based on patient-specific factors affecting tolerability	Ta et al. (2025) | PDF pp.2,6 | Methods/limitations	Serious	Starting dose reflected clinician assessment; ECOG was largely unknown, prior therapy differed, and the reported dose comparisons were unadjusted.	Figure 3; complete result-level details retained in this table
Ta 2025	ROBINS-I 2016 2016	Ta 2025: hospitalization	D2 Bias in selection of	Low	adult KP members ... who	Ta et al. (2025) | PDF p.2 |	Serious	The integrated-care cohort	Figure 3; complete result-

Study	Tool	Outcome or result	Domain	Judgment	Support for judgment	Information source	Overall judgment	Additional rationale	Figure link
	original cohort-type version (19 September 2016)	all-cause outcome definition	participants into the study		initiated combination therapy	Methods/Results		included initiators and used pre-intervention exclusions; follow-up began at the first pembrolizumab administration.	level details retained in this table
Ta 2025	ROBINS-I 2016 2016 original cohort-type version (19 September 2016)	Ta 2025: hospitalization all-cause outcome definition	D3 Bias in classification of interventions	Low	grouped based on their initial lenvatinib dose	Ta et al. (2025) | PDF p.2 | Methods	Serious	The initial-dose groups were explicit and obtained from the EHR before outcome occurrence.	Figure 3; complete result-level details retained in this table
Ta 2025	ROBINS-I 2016 2016 original cohort-type version (19 September 2016)	Ta 2025: hospitalization all-cause outcome definition	D4 Bias due to deviations from intended interventions	Low	This was an intent-to-treat analysis	Ta et al. (2025) | PDF pp.2,6 | Methods/limitations	Serious	The starting-dose assignment effect was retained; later doses were not used to reclassify participants.	Figure 3; complete result-level details retained in this table
Ta 2025	ROBINS-I 2016 2016 original cohort-type version (19 September 2016)	Ta 2025: hospitalization all-cause outcome definition	D5 Bias due to missing data	Low	hospitalizations ... within six months	Ta et al. (2025) | PDF pp.2–3 | Outcome availability/missingness	Serious	Six-month hospitalization was reported for both groups.	Figure 3; complete result-level details retained in this table
Ta 2025	ROBINS-I 2016 2016 original cohort-type version (19 September 2016)	Ta 2025: hospitalization all-cause outcome definition	D6 Bias in measurement of outcomes	Low	hospitalizations ... within six months	Ta et al. (2025) | PDF pp.2–3 | Outcome measurement	Serious	Hospital admission is objective and the common six-month window was applied to both groups.	Figure 3; complete result-level details retained in this table
Ta 2025	ROBINS-I 2016 2016 original cohort-type version (19 September 2016)	Ta 2025: hospitalization all-cause outcome definition	D7 Bias in selection of the reported result	Moderate	6-month progression-free survival and best overall response ... were reported	Ta et al. (2025) | PDF pp.2,6; ASCO companion abstract | Statistical analysis/limitations	Serious	The main report and companion were consistent, but no public prespecified analysis plan was located.	Figure 3; complete result-level details retained in this table
Ta 2025	ROBINS-I 2016 2016 original cohort-type version (19 September 2016)	Ta 2025: hospitalization all-cause outcome definition	Overall Overall risk of bias	Serious	at least one domain was judged Serious	Ta et al. (2025) | See linked D1-D7 anchors | ROBINS-I overall algorithm	Serious	ROBINS-I 2016 overall judgment equals the highest domain when no domain is Critical.	Figure 3; complete result-level details retained in this table
Ta 2025	ROBINS-I 2016 2016 original cohort-type version (19 September 2016)	Ta 2025: progression-free survival (reported unadjusted HR)	D1 Bias due to confounding	Serious	lower starting dose based on patient-specific factors affecting tolerability	Ta et al. (2025) | PDF pp.2,6 | Methods/limitations	Serious	Starting dose reflected clinician assessment; ECOG was largely unknown, prior therapy differed, and the reported dose comparisons were unadjusted.	Figure 3; complete result-level details retained in this table
Ta 2025	ROBINS-I 2016 2016 original cohort-type version (19 September 2016)	Ta 2025: progression-free survival (risk ratio)	D1 Bias due to confounding	Serious	lower starting dose based on patient-specific factors affecting tolerability	Ta et al. (2025) | PDF pp.2,6 | Methods/limitations	Serious	Starting dose reflected clinician assessment; ECOG was largely unknown, prior therapy differed, and the reported dose comparisons were unadjusted.	Figure 3; complete result-level details retained in this table
Ta 2025	ROBINS-I 2016 2016 original cohort-type version (19 September 2016)	Ta 2025: progression-free survival (reported unadjusted HR)	D2 Bias in selection of participants into the study	Low	adult KP members ... who initiated combination therapy	Ta et al. (2025) | PDF p.2 | Methods/Results	Serious	The integrated-care cohort included initiators and used pre-intervention exclusions; follow-up began at the first pembrolizumab administration.	Figure 3; complete result-level details retained in this table
Ta 2025	ROBINS-I 2016 2016 original cohort-type version (19 September 2016)	Ta 2025: progression-free survival (risk ratio)	D2 Bias in selection of participants into the study	Low	adult KP members ... who initiated combination therapy	Ta et al. (2025) | PDF p.2 | Methods/Results	Serious	The integrated-care cohort included initiators and used pre-intervention exclusions; follow-up began at the first pembrolizumab administration.	Figure 3; complete result-level details retained in this table
Ta 2025	ROBINS-I 2016 original cohort-type version (19 September 2016)	Ta 2025: progression-free survival (reported unadjusted HR)	D3 Bias in classification of interventions	Low	grouped based on their initial lenvatinib dose	Ta et al. (2025) | PDF p.2 | Methods	Serious	The initial-dose groups were explicit and obtained from the EHR before outcome occurrence.	Figure 3; complete result-level details retained in this table

Study	Tool	Outcome or result	Domain	Judgment	Support for judgment	Information source	Overall judgment	Additional rationale	Figure link
Ta 2025	ROBINS-I 2016 2016 original cohort-type version (19 September 2016)	Ta 2025: progression-free survival (risk ratio)	D3 Bias in classification of interventions	Low	grouped based on their initial lenvatinib dose	Ta et al. (2025) | PDF p.2 | Methods	Serious	The initial-dose groups were explicit and obtained from the EHR before outcome occurrence.	Figure 3; complete result-level details retained in this table
Ta 2025	ROBINS-I 2016 2016 original cohort-type version (19 September 2016)	Ta 2025: progression-free survival (reported unadjusted HR)	D4 Bias due to deviations from intended interventions	Low	This was an intent-to-treat analysis	Ta et al. (2025) | PDF pp.2,6 | Methods/limitations	Serious	The starting-dose assignment effect was retained; later doses were not used to reclassify participants.	Figure 3; complete result-level details retained in this table
Ta 2025	ROBINS-I 2016 2016 original cohort-type version (19 September 2016)	Ta 2025: progression-free survival (risk ratio)	D4 Bias due to deviations from intended interventions	Low	This was an intent-to-treat analysis	Ta et al. (2025) | PDF pp.2,6 | Methods/limitations	Serious	The starting-dose assignment effect was retained; later doses were not used to reclassify participants.	Figure 3; complete result-level details retained in this table
Ta 2025	ROBINS-I 2016 2016 original cohort-type version (19 September 2016)	Ta 2025: progression-free survival (reported unadjusted HR)	D5 Bias due to missing data	Moderate	ability to assess ... progression was limited by whether and when ... imaging	Ta et al. (2025) | PDF pp.2,4,6 | Outcome availability/missingness	Serious	Health-plan loss and variable imaging can create incomplete outcome observation.	Figure 3; complete result-level details retained in this table
Ta 2025	ROBINS-I 2016 2016 original cohort-type version (19 September 2016)	Ta 2025: progression-free survival (risk ratio)	D5 Bias due to missing data	Moderate	ability to assess ... progression was limited by whether and when ... imaging	Ta et al. (2025) | PDF pp.2,4,6 | Outcome availability/missingness	Serious	Health-plan loss and variable imaging can create incomplete outcome observation.	Figure 3; complete result-level details retained in this table
Ta 2025	ROBINS-I 2016 2016 original cohort-type version (19 September 2016)	Ta 2025: progression-free survival (reported unadjusted HR)	D6 Bias in measurement of outcomes	Serious	ability to assess ... progression was limited by whether and when ... imaging	Ta et al. (2025) | PDF pp.2,4,6 | Outcome measurement	Serious	Nonuniform imaging timing and criteria can differentially affect progression detection.	Figure 3; complete result-level details retained in this table
Ta 2025	ROBINS-I 2016 2016 original cohort-type version (19 September 2016)	Ta 2025: progression-free survival (risk ratio)	D6 Bias in measurement of outcomes	Serious	ability to assess ... progression was limited by whether and when ... imaging	Ta et al. (2025) | PDF pp.2,4,6 | Outcome measurement	Serious	Nonuniform imaging timing and criteria can differentially affect progression detection.	Figure 3; complete result-level details retained in this table
Ta 2025	ROBINS-I 2016 2016 original cohort-type version (19 September 2016)	Ta 2025: progression-free survival (reported unadjusted HR)	D7 Bias in selection of the reported result	Moderate	6-month progression-free survival and best overall response ... were reported	Ta et al. (2025) | PDF pp.2,6; ASCO companion abstract | Statistical analysis/limitations	Serious	The main report and companion were consistent, but no public prespecified analysis plan was located.	Figure 3; complete result-level details retained in this table
Ta 2025	ROBINS-I 2016 2016 original cohort-type version (19 September 2016)	Ta 2025: progression-free survival (risk ratio)	D7 Bias in selection of the reported result	Moderate	6-month progression-free survival and best overall response ... were reported	Ta et al. (2025) | PDF pp.2,6; ASCO companion abstract | Statistical analysis/limitations	Serious	The main report and companion were consistent, but no public prespecified analysis plan was located.	Figure 3; complete result-level details retained in this table
Ta 2025	ROBINS-I 2016 2016 original cohort-type version (19 September 2016)	Ta 2025: progression-free survival (reported unadjusted HR)	Overall Overall risk of bias	Serious	at least one domain was judged Serious	Ta et al. (2025) | See linked D1-D7 anchors | ROBINS-I overall algorithm	Serious	ROBINS-I 2016 overall judgment equals the highest domain when no domain is Critical.	Figure 3; complete result-level details retained in this table
Ta 2025	ROBINS-I 2016 2016 original cohort-type version (19 September 2016)	Ta 2025: progression-free survival (risk ratio)	Overall Overall risk of bias	Serious	at least one domain was judged Serious	Ta et al. (2025) | See linked D1-D7 anchors | ROBINS-I overall algorithm	Serious	ROBINS-I 2016 overall judgment equals the highest domain when no domain is Critical.	Figure 3; complete result-level details retained in this table
Ta 2025	ROBINS-I 2016 2016 original cohort-type version (19 September 2016)	DISC RATE | Result-level assessment: Ta 2025: treatment discontinuation (risk ratio)	D1 Bias due to confounding	Serious	lower starting dose based on patient-specific factors affecting tolerability	Ta et al. (2025) | PDF pp.2,6 | Methods/limitations	Serious	Starting dose reflected clinician assessment; ECOG was largely unknown, prior therapy differed, and the reported dose comparisons were unadjusted.	Figure 3; complete result-level details retained in this table
Ta 2025	ROBINS-I 2016 2016 original cohort-type version (19 September 2016)	REDUCTION RATE | Result-level assessment: Ta 2025: subsequent lenvatinib dose reduction (risk ratio)	D1 Bias due to confounding	Serious	lower starting dose based on patient-specific factors affecting tolerability	Ta et al. (2025) | PDF pp.2,6 | Methods/limitations	Serious	Starting dose reflected clinician assessment; ECOG was largely unknown, prior therapy differed, and the reported dose comparisons were unadjusted.	Figure 3; complete result-level details retained in this table
Ta 2025	ROBINS-I 2016 2016	DISC RATE | Result-	D2 Bias in selection of	Low	adult KP members ... who	Ta et al. (2025) | PDF p.2 |	Serious	The integrated-care cohort	Figure 3; complete result-

Study	Tool	Outcome or result	Domain	Judgment	Support for judgment	Information source	Overall judgment	Additional rationale	Figure link
	original cohort-type version (19 September 2016)	level assessment: Ta 2025: treatment discontinuation (risk ratio)	participants into the study		initiated combination therapy	Methods/Results		included initiators and used pre-intervention exclusions; follow-up began at the first pembrolizumab administration.	level details retained in this table
Ta 2025	ROBINS-I 2016 2016 original cohort-type version (19 September 2016)	REDUCTION RATE | Result-level assessment: Ta 2025: subsequent lenvatinib dose reduction (risk ratio)	D2 Bias in selection of participants into the study	Low	adult KP members ... who initiated combination therapy	Ta et al. (2025) | PDF p.2 | Methods/Results	Serious	The integrated-care cohort included initiators and used pre-intervention exclusions; follow-up began at the first pembrolizumab administration.	Figure 3; complete result-level details retained in this table
Ta 2025	ROBINS-I 2016 2016 original cohort-type version (19 September 2016)	DISC RATE | Result-level assessment: Ta 2025: treatment discontinuation (risk ratio)	D3 Bias in classification of interventions	Low	grouped based on their initial lenvatinib dose	Ta et al. (2025) | PDF p.2 | Methods	Serious	The initial-dose groups were explicit and obtained from the EHR before outcome occurrence.	Figure 3; complete result-level details retained in this table
Ta 2025	ROBINS-I 2016 2016 original cohort-type version (19 September 2016)	REDUCTION RATE | Result-level assessment: Ta 2025: subsequent lenvatinib dose reduction (risk ratio)	D3 Bias in classification of interventions	Low	grouped based on their initial lenvatinib dose	Ta et al. (2025) | PDF p.2 | Methods	Serious	The initial-dose groups were explicit and obtained from the EHR before outcome occurrence.	Figure 3; complete result-level details retained in this table
Ta 2025	ROBINS-I 2016 2016 original cohort-type version (19 September 2016)	DISC RATE | Result-level assessment: Ta 2025: treatment discontinuation (risk ratio)	D4 Bias due to deviations from intended interventions	Low	This was an intent-to-treat analysis	Ta et al. (2025) | PDF pp.2,6 | Methods/limitations	Serious	The starting-dose assignment effect was retained; later doses were not used to reclassify participants.	Figure 3; complete result-level details retained in this table
Ta 2025	ROBINS-I 2016 2016 original cohort-type version (19 September 2016)	REDUCTION RATE | Result-level assessment: Ta 2025: subsequent lenvatinib dose reduction (risk ratio)	D4 Bias due to deviations from intended interventions	Low	This was an intent-to-treat analysis	Ta et al. (2025) | PDF pp.2,6 | Methods/limitations	Serious	The starting-dose assignment effect was retained; later doses were not used to reclassify participants.	Figure 3; complete result-level details retained in this table
Ta 2025	ROBINS-I 2016 2016 original cohort-type version (19 September 2016)	DISC RATE | Result-level assessment: Ta 2025: treatment discontinuation (risk ratio)	D5 Bias due to missing data	Moderate	events were not mutually exclusive	Ta et al. (2025) | PDF pp.2,5–6 | Outcome availability/missingness	Serious	Complete person-time and repeat-event structure were not provided for independent reconstruction.	Figure 3; complete result-level details retained in this table
Ta 2025	ROBINS-I 2016 2016 original cohort-type version (19 September 2016)	REDUCTION RATE | Result-level assessment: Ta 2025: subsequent lenvatinib dose reduction (risk ratio)	D5 Bias due to missing data	Moderate	events were not mutually exclusive	Ta et al. (2025) | PDF pp.2,5–6 | Outcome availability/missingness	Serious	Complete person-time and repeat-event structure were not provided for independent reconstruction.	Figure 3; complete result-level details retained in this table
Ta 2025	ROBINS-I 2016 2016 original cohort-type version (19 September 2016)	DISC RATE | Result-level assessment: Ta 2025: treatment discontinuation (risk ratio)	D6 Bias in measurement of outcomes	Serious	events were not mutually exclusive	Ta et al. (2025) | PDF pp.2,5–6 | Outcome measurement	Serious	Dose changes and toxicity-related discontinuations were clinician-determined in variable chart documentation.	Figure 3; complete result-level details retained in this table
Ta 2025	ROBINS-I 2016 2016 original cohort-type version (19 September 2016)	REDUCTION RATE | Result-level assessment: Ta 2025: subsequent lenvatinib dose reduction (risk ratio)	D6 Bias in measurement of outcomes	Serious	events were not mutually exclusive	Ta et al. (2025) | PDF pp.2,5–6 | Outcome measurement	Serious	Dose changes and toxicity-related discontinuations were clinician-determined in variable chart documentation.	Figure 3; complete result-level details retained in this table
Ta 2025	ROBINS-I 2016 2016 original cohort-type version (19 September 2016)	DISC RATE | Result-level assessment: Ta 2025: treatment discontinuation (risk ratio)	D7 Bias in selection of the reported result	Moderate	6-month progression-free survival and best overall response ... were reported	Ta et al. (2025) | PDF pp.2,6; ASCO companion abstract | Statistical analysis/limitations	Serious	The main report and companion were consistent, but no public prespecified analysis plan was located.	Figure 3; complete result-level details retained in this table
Ta 2025	ROBINS-I 2016 2016	REDUCTION RATE |	D7 Bias in selection of the	Moderate	6-month progression-free	Ta et al. (2025) | PDF pp.2,6;	Serious	The main report and	Figure 3; complete result-

Study	Tool	Outcome or result	Domain	Judgment	Support for judgment	Information source	Overall judgment	Additional rationale	Figure link
	original cohort-type version (19 September 2016)	Result-level assessment: Ta 2025: subsequent lenvatinib dose reduction (risk ratio)	reported result		survival and best overall response ... were reported	ASCO companion abstract | Statistical analysis/limitations		companion were consistent, but no public prespecified analysis plan was located.	level details retained in this table
Ta 2025	ROBINS-I 2016 2016 original cohort-type version (19 September 2016)	DISC RATE | Result-level assessment: Ta 2025: treatment discontinuation (risk ratio)	Overall Overall risk of bias	Serious	at least one domain was judged Serious	Ta et al. (2025) | See linked D1-D7 anchors | ROBINS-I overall algorithm	Serious	ROBINS-I 2016 overall judgment equals the highest domain when no domain is Critical.	Figure 3; complete result-level details retained in this table
Ta 2025	ROBINS-I 2016 2016 original cohort-type version (19 September 2016)	REDUCTION RATE | Result-level assessment: Ta 2025: subsequent lenvatinib dose reduction (risk ratio)	Overall Overall risk of bias	Serious	at least one domain was judged Serious	Ta et al. (2025) | See linked D1-D7 anchors | ROBINS-I overall algorithm	Serious	ROBINS-I 2016 overall judgment equals the highest domain when no domain is Critical.	Figure 3; complete result-level details retained in this table
Ta 2025	ROBINS-I 2016 2016 original cohort-type version (19 September 2016)	Ta 2025: disease control (risk ratio)	D1 Bias due to confounding	Serious	lower starting dose based on patient-specific factors affecting tolerability	Ta et al. (2025) | PDF pp.2,6 | Methods/limitations	Serious	Starting dose reflected clinician assessment; ECOG was largely unknown, prior therapy differed, and the reported dose comparisons were unadjusted.	Figure 3; complete result-level details retained in this table
Ta 2025	ROBINS-I 2016 2016 original cohort-type version (19 September 2016)	Ta 2025: objective response (risk ratio)	D1 Bias due to confounding	Serious	lower starting dose based on patient-specific factors affecting tolerability	Ta et al. (2025) | PDF pp.2,6 | Methods/limitations	Serious	Starting dose reflected clinician assessment; ECOG was largely unknown, prior therapy differed, and the reported dose comparisons were unadjusted.	Figure 3; complete result-level details retained in this table
Ta 2025	ROBINS-I 2016 2016 original cohort-type version (19 September 2016)	Ta 2025: disease control (risk ratio)	D2 Bias in selection of participants into the study	Low	adult KP members ... who initiated combination therapy	Ta et al. (2025) | PDF p.2 | Methods/Results	Serious	The integrated-care cohort included initiators and used pre-intervention exclusions; follow-up began at the first pembrolizumab administration.	Figure 3; complete result-level details retained in this table
Ta 2025	ROBINS-I 2016 2016 original cohort-type version (19 September 2016)	Ta 2025: objective response (risk ratio)	D2 Bias in selection of participants into the study	Low	adult KP members ... who initiated combination therapy	Ta et al. (2025) | PDF p.2 | Methods/Results	Serious	The integrated-care cohort included initiators and used pre-intervention exclusions; follow-up began at the first pembrolizumab administration.	Figure 3; complete result-level details retained in this table
Ta 2025	ROBINS-I 2016 2016 original cohort-type version (19 September 2016)	Ta 2025: disease control (risk ratio)	D3 Bias in classification of interventions	Low	grouped based on their initial lenvatinib dose	Ta et al. (2025) | PDF p.2 | Methods	Serious	The initial-dose groups were explicit and obtained from the EHR before outcome occurrence.	Figure 3; complete result-level details retained in this table
Ta 2025	ROBINS-I 2016 2016 original cohort-type version (19 September 2016)	Ta 2025: objective response (risk ratio)	D3 Bias in classification of interventions	Low	grouped based on their initial lenvatinib dose	Ta et al. (2025) | PDF p.2 | Methods	Serious	The initial-dose groups were explicit and obtained from the EHR before outcome occurrence.	Figure 3; complete result-level details retained in this table
Ta 2025	ROBINS-I 2016 2016 original cohort-type version (19 September 2016)	Ta 2025: disease control (risk ratio)	D4 Bias due to deviations from intended interventions	Low	This was an intent-to-treat analysis	Ta et al. (2025) | PDF pp.2,6 | Methods/limitations	Serious	The starting-dose assignment effect was retained; later doses were not used to reclassify participants.	Figure 3; complete result-level details retained in this table
Ta 2025	ROBINS-I 2016 2016 original cohort-type version (19 September 2016)	Ta 2025: objective response (risk ratio)	D4 Bias due to deviations from intended interventions	Low	This was an intent-to-treat analysis	Ta et al. (2025) | PDF pp.2,6 | Methods/limitations	Serious	The starting-dose assignment effect was retained; later doses were not used to reclassify participants.	Figure 3; complete result-level details retained in this table
Ta 2025	ROBINS-I 2016 2016 original cohort-type version (19 September 2016)	Ta 2025: disease control (risk ratio)	D5 Bias due to missing data	Moderate	N/A ... 33 (8.9%)	Ta et al. (2025) | PDF pp.2,4,6 | Outcome availability/missingness	Serious	Response was unavailable for 8.9% with some imbalance between dose groups.	Figure 3; complete result-level details retained in this table

Study	Tool	Outcome or result	Domain	Judgment	Support for judgment	Information source	Overall judgment	Additional rationale	Figure link
Ta 2025	ROBINS-I 2016 2016 original cohort-type version (19 September 2016)	Ta 2025: objective response (risk ratio)	D5 Bias due to missing data	Moderate	N/A ... 33 (8.9%)	Ta et al. (2025) | PDF pp.2,4,6 | Outcome availability/missingness	Serious	Response was unavailable for 8.9% with some imbalance between dose groups.	Figure 3; complete result-level details retained in this table
Ta 2025	ROBINS-I 2016 2016 original cohort-type version (19 September 2016)	Ta 2025: disease control (risk ratio)	D6 Bias in measurement of outcomes	Serious	N/A ... 33 (8.9%)	Ta et al. (2025) | PDF pp.2,4,6 | Outcome measurement	Serious	The authors report nonuniform imaging schedules and no uniform adherence to RECIST.	Figure 3; complete result-level details retained in this table
Ta 2025	ROBINS-I 2016 2016 original cohort-type version (19 September 2016)	Ta 2025: objective response (risk ratio)	D6 Bias in measurement of outcomes	Serious	N/A ... 33 (8.9%)	Ta et al. (2025) | PDF pp.2,4,6 | Outcome measurement	Serious	The authors report nonuniform imaging schedules and no uniform adherence to RECIST.	Figure 3; complete result-level details retained in this table
Ta 2025	ROBINS-I 2016 2016 original cohort-type version (19 September 2016)	Ta 2025: disease control (risk ratio)	D7 Bias in selection of the reported result	Moderate	6-month progression-free survival and best overall response ... were reported	Ta et al. (2025) | PDF pp.2,6; ASCO companion abstract | Statistical analysis/limitations	Serious	The main report and companion were consistent, but no public prespecified analysis plan was located.	Figure 3; complete result-level details retained in this table
Ta 2025	ROBINS-I 2016 original cohort-type version (19 September 2016)	Ta 2025: objective response (risk ratio)	D7 Bias in selection of the reported result	Moderate	6-month progression-free survival and best overall response ... were reported	Ta et al. (2025) | PDF pp.2,6; ASCO companion abstract | Statistical analysis/limitations	Serious	The main report and companion were consistent, but no public prespecified analysis plan was located.	Figure 3; complete result-level details retained in this table
Ta 2025	ROBINS-I 2016 2016 original cohort-type version (19 September 2016)	Ta 2025: disease control (risk ratio)	Overall Overall risk of bias	Serious	at least one domain was judged Serious	Ta et al. (2025) | See linked D1-D7 anchors | ROBINS-I overall algorithm	Serious	ROBINS-I 2016 overall judgment equals the highest domain when no domain is Critical.	Figure 3; complete result-level details retained in this table
Ta 2025	ROBINS-I 2016 2016 original cohort-type version (19 September 2016)	Ta 2025: objective response (risk ratio)	Overall Overall risk of bias	Serious	at least one domain was judged Serious	Ta et al. (2025) | See linked D1-D7 anchors | ROBINS-I overall algorithm	Serious	ROBINS-I 2016 overall judgment equals the highest domain when no domain is Critical.	Figure 3; complete result-level details retained in this table
Corr 2025	ROBINS-I 2016 2016 original cohort-type version (19 September 2016)	Corr 2025: grade 3 or higher adverse events (risk ratio)	D1 Bias due to confounding	Serious	starting doses ... were made at the treating providers' discretion	Corr et al. (2025) | PDF pp.2–4 | Methods/Results/Discussion	Serious	Provider-selected dose, age imbalance, and absence of multivariable adjustment leave important confounding uncontrolled.	Figure 3; complete result-level details retained in this table
Corr 2025	ROBINS-I 2016 2016 original cohort-type version (19 September 2016)	Corr 2025: grade 3 or higher adverse events (risk ratio)	D2 Bias in selection of participants into the study	Moderate	18 patients were excluded due to incomplete data	Corr et al. (2025) | PDF p.3 | Results/Patient characteristics	Serious	Post-identification incomplete-data exclusions and exclusion of uncommon dose groups may select the analyzed cohort.	Figure 3; complete result-level details retained in this table
Corr 2025	ROBINS-I 2016 2016 original cohort-type version (19 September 2016)	Corr 2025: grade 3 or higher adverse events (risk ratio)	D3 Bias in classification of interventions	Low	Patients were categorized by lenvatinib starting doses	Corr et al. (2025) | PDF pp.2–3 | Methods/Regimen	Serious	Four starting-dose groups were explicitly defined from chart data at treatment initiation.	Figure 3; complete result-level details retained in this table
Corr 2025	ROBINS-I 2016 2016 original cohort-type version (19 September 2016)	Corr 2025: grade 3 or higher adverse events (risk ratio)	D4 Bias due to deviations from intended interventions	Low	dose modifications ... were made at the treating providers' discretion	Corr et al. (2025) | PDF pp.2–3 | Methods/Regimen	Serious	For the assignment estimand, routine modifications reflect usual practice and were not used to redefine baseline exposure.	Figure 3; complete result-level details retained in this table
Corr 2025	ROBINS-I 2016 2016 original cohort-type version (19 September 2016)	Corr 2025: grade 3 or higher adverse events (risk ratio)	D5 Bias due to missing data	Serious	toxicity data is less commonly reported ... harder to abstract	Corr et al. (2025) | PDF pp.2–4,6 | Outcome availability/missingness	Serious	Consensus rationale: Observed AE capture was markedly incomplete and 18 patients with incomplete data were excluded.	Figure 3; complete result-level details retained in this table
Corr 2025	ROBINS-I 2016 original cohort-type version (19 September 2016)	Corr 2025: grade 3 or higher adverse events (risk ratio)	D6 Bias in measurement of outcomes	Serious	toxicity data is less commonly reported ... harder to abstract	Corr et al. (2025) | PDF pp.2–4,6 | Outcome measurement	Serious	Chart-based retrospective toxicity capture was acknowledged to underreport events and was not blinded.	Figure 3; complete result-level details retained in this table

Study	Tool	Outcome or result	Domain	Judgment	Support for judgment	Information source	Overall judgment	Additional rationale	Figure link
Corr 2025	ROBINS-I 2016 2016 original cohort-type version (19 September 2016)	Corr 2025: grade 3 or higher adverse events (risk ratio)	D7 Bias in selection of the reported result	Moderate	No adjustments were made for multiple comparisons	Corr et al. (2025) | PDF pp.2,4 | Methods/Discussion	Serious	Multiple doses, time points, outcomes, and race subgroups were analyzed without a public prespecified plan.	Figure 3; complete result-level details retained in this table
Corr 2025	ROBINS-I 2016 2016 original cohort-type version (19 September 2016)	Corr 2025: grade 3 or higher adverse events (risk ratio)	Overall Overall risk of bias	Serious	at least one domain was judged Serious	Corr et al. (2025) | See linked D1-D7 anchors | ROBINS-I overall algorithm	Serious	ROBINS-I 2016 overall judgment equals the highest domain when no domain is Critical.	Figure 3; complete result-level details retained in this table
Corr 2025	ROBINS-I 2016 2016 original cohort-type version (19 September 2016)	Corr 2025: treatment discontinuation all-cause outcome definition	D1 Bias due to confounding	Serious	starting doses ... were made at the treating providers' discretion	Corr et al. (2025) | PDF pp.2–4 | Methods/Results/Discussion	Serious	Provider-selected dose, age imbalance, and absence of multivariable adjustment leave important confounding uncontrolled.	Figure 3; complete result-level details retained in this table
Corr 2025	ROBINS-I 2016 2016 original cohort-type version (19 September 2016)	Corr 2025: treatment discontinuation toxicity-attributed outcome definition	D1 Bias due to confounding	Serious	starting doses ... were made at the treating providers' discretion	Corr et al. (2025) | PDF pp.2–4 | Methods/Results/Discussion	Serious	Provider-selected dose, age imbalance, and absence of multivariable adjustment leave important confounding uncontrolled.	Figure 3; complete result-level details retained in this table
Corr 2025	ROBINS-I 2016 2016 original cohort-type version (19 September 2016)	Corr 2025: treatment discontinuation all-cause outcome definition	D2 Bias in selection of participants into the study	Moderate	18 patients were excluded due to incomplete data	Corr et al. (2025) | PDF p.3 | Results/Patient characteristics	Serious	Post-identification incomplete-data exclusions and exclusion of uncommon dose groups may select the analyzed cohort.	Figure 3; complete result-level details retained in this table
Corr 2025	ROBINS-I 2016 2016 original cohort-type version (19 September 2016)	Corr 2025: treatment discontinuation toxicity-attributed outcome definition	D2 Bias in selection of participants into the study	Moderate	18 patients were excluded due to incomplete data	Corr et al. (2025) | PDF p.3 | Results/Patient characteristics	Serious	Post-identification incomplete-data exclusions and exclusion of uncommon dose groups may select the analyzed cohort.	Figure 3; complete result-level details retained in this table
Corr 2025	ROBINS-I 2016 2016 original cohort-type version (19 September 2016)	Corr 2025: treatment discontinuation all-cause outcome definition	D3 Bias in classification of interventions	Low	Patients were categorized by lenvatinib starting doses	Corr et al. (2025) | PDF pp.2–3 | Methods/Regimen	Serious	Four starting-dose groups were explicitly defined from chart data at treatment initiation.	Figure 3; complete result-level details retained in this table
Corr 2025	ROBINS-I 2016 2016 original cohort-type version (19 September 2016)	Corr 2025: treatment discontinuation toxicity-attributed outcome definition	D3 Bias in classification of interventions	Low	Patients were categorized by lenvatinib starting doses	Corr et al. (2025) | PDF pp.2–3 | Methods/Regimen	Serious	Four starting-dose groups were explicitly defined from chart data at treatment initiation.	Figure 3; complete result-level details retained in this table
Corr 2025	ROBINS-I 2016 2016 original cohort-type version (19 September 2016)	Corr 2025: treatment discontinuation all-cause outcome definition	D4 Bias due to deviations from intended interventions	Low	dose modifications ... were made at the treating providers' discretion	Corr et al. (2025) | PDF pp.2–3 | Methods/Regimen	Serious	For the assignment estimand, routine modifications reflect usual practice and were not used to redefine baseline exposure.	Figure 3; complete result-level details retained in this table
Corr 2025	ROBINS-I 2016 2016 original cohort-type version (19 September 2016)	Corr 2025: treatment discontinuation toxicity-attributed outcome definition	D4 Bias due to deviations from intended interventions	Low	dose modifications ... were made at the treating providers' discretion	Corr et al. (2025) | PDF pp.2–3 | Methods/Regimen	Serious	For the assignment estimand, routine modifications reflect usual practice and were not used to redefine baseline exposure.	Figure 3; complete result-level details retained in this table
Corr 2025	ROBINS-I 2016 2016 original cohort-type version (19 September 2016)	Corr 2025: treatment discontinuation all-cause outcome definition	D5 Bias due to missing data	Moderate	reason ... abstracted ... where data was available	Corr et al. (2025) | PDF pp.3–4 | Outcome availability/missingness	Serious	Reasons were not available for all discontinuations.	Figure 3; complete result-level details retained in this table
Corr 2025	ROBINS-I 2016 2016 original cohort-type version (19 September 2016)	Corr 2025: treatment discontinuation toxicity-attributed outcome definition	D5 Bias due to missing data	Moderate	reason ... abstracted ... where data was available	Corr et al. (2025) | PDF pp.3–4 | Outcome availability/missingness	Serious	Reasons were not available for all discontinuations.	Figure 3; complete result-level details retained in this table
Corr 2025	ROBINS-I 2016 original cohort-type version (19 September 2016)	Corr 2025: treatment discontinuation all-cause outcome definition	D6 Bias in measurement of outcomes	Serious	reason ... abstracted ... where data was available	Corr et al. (2025) | PDF pp.3–4 | Outcome measurement	Serious	Discontinuation cause and tolerability were provider/chart judgments	Figure 3; complete result-level details retained in this table

Study	Tool	Outcome or result	Domain	Judgment	Support for judgment	Information source	Overall judgment	Additional rationale	Figure link
								without blinding.	
Corr 2025	ROBINS-I 2016 2016 original cohort-type version (19 September 2016)	Corr 2025: treatment discontinuation toxicity-attributed outcome definition	D6 Bias in measurement of outcomes	Serious	reason ... abstracted ... where data was available	Corr et al. (2025) | PDF pp.3–4 | Outcome measurement	Serious	Discontinuation cause and tolerability were provider/chart judgments without blinding.	Figure 3; complete result-level details retained in this table
Corr 2025	ROBINS-I 2016 2016 original cohort-type version (19 September 2016)	Corr 2025: treatment discontinuation all-cause outcome definition	D7 Bias in selection of the reported result	Moderate	No adjustments were made for multiple comparisons	Corr et al. (2025) | PDF pp.2,4 | Methods/Discussion	Serious	Multiple doses, time points, outcomes, and race subgroups were analyzed without a public prespecified plan.	Figure 3; complete result-level details retained in this table
Corr 2025	ROBINS-I 2016 2016 original cohort-type version (19 September 2016)	Corr 2025: treatment discontinuation toxicity-attributed outcome definition	D7 Bias in selection of the reported result	Moderate	No adjustments were made for multiple comparisons	Corr et al. (2025) | PDF pp.2,4 | Methods/Discussion	Serious	Multiple doses, time points, outcomes, and race subgroups were analyzed without a public prespecified plan.	Figure 3; complete result-level details retained in this table
Corr 2025	ROBINS-I 2016 2016 original cohort-type version (19 September 2016)	Corr 2025: treatment discontinuation all-cause outcome definition	Overall Overall risk of bias	Serious	at least one domain was judged Serious	Corr et al. (2025) | See linked D1-D7 anchors | ROBINS-I overall algorithm	Serious	ROBINS-I 2016 overall judgment equals the highest domain when no domain is Critical.	Figure 3; complete result-level details retained in this table
Corr 2025	ROBINS-I 2016 2016 original cohort-type version (19 September 2016)	Corr 2025: treatment discontinuation toxicity-attributed outcome definition	Overall Overall risk of bias	Serious	at least one domain was judged Serious	Corr et al. (2025) | See linked D1-D7 anchors | ROBINS-I overall algorithm	Serious	ROBINS-I 2016 overall judgment equals the highest domain when no domain is Critical.	Figure 3; complete result-level details retained in this table
Corr 2025	ROBINS-I 2016 2016 original cohort-type version (19 September 2016)	OS | Result-level assessment: Corr 2025: overall survival (Kaplan–Meier curve available; HR not derived)	D1 Bias due to confounding	Serious	starting doses ... were made at the treating providers' discretion	Corr et al. (2025) | PDF pp.2–4 | Methods/Results/Discussion	Serious	Provider-selected dose, age imbalance, and absence of multivariable adjustment leave important confounding uncontrolled.	Figure 3; complete result-level details retained in this table
Corr 2025	ROBINS-I 2016 2016 original cohort-type version (19 September 2016)	OS | Result-level assessment: Corr 2025: overall survival (Kaplan–Meier curve available; HR not derived)	D2 Bias in selection of participants into the study	Moderate	18 patients were excluded due to incomplete data	Corr et al. (2025) | PDF p.3 | Results/Patient characteristics	Serious	Post-identification incomplete-data exclusions and exclusion of uncommon dose groups may select the analyzed cohort.	Figure 3; complete result-level details retained in this table
Corr 2025	ROBINS-I 2016 2016 original cohort-type version (19 September 2016)	OS | Result-level assessment: Corr 2025: overall survival (Kaplan–Meier curve available; HR not derived)	D3 Bias in classification of interventions	Low	Patients were categorized by lenvatinib starting doses	Corr et al. (2025) | PDF pp.2–3 | Methods/Regimen	Serious	Four starting-dose groups were explicitly defined from chart data at treatment initiation.	Figure 3; complete result-level details retained in this table
Corr 2025	ROBINS-I 2016 2016 original cohort-type version (19 September 2016)	OS | Result-level assessment: Corr 2025: overall survival (Kaplan–Meier curve available; HR not derived)	D4 Bias due to deviations from intended interventions	Low	dose modifications ... were made at the treating providers' discretion	Corr et al. (2025) | PDF pp.2–3 | Methods/Regimen	Serious	For the assignment estimand, routine modifications reflect usual practice and were not used to redefine baseline exposure.	Figure 3; complete result-level details retained in this table
Corr 2025	ROBINS-I 2016 2016 original cohort-type version (19 September 2016)	OS | Result-level assessment: Corr 2025: overall survival (Kaplan–Meier curve available; HR not derived)	D5 Bias due to missing data	Serious	OS ... only estimated for patients with available data	Corr et al. (2025) | PDF pp.2–4 | Outcome availability/missingness	Serious	Consensus rationale: Available-case survival analysis lacked group-specific completeness information.	Figure 3; complete result-level details retained in this table
Corr 2025	ROBINS-I 2016 2016 original cohort-type version (19 September 2016)	OS | Result-level assessment: Corr 2025: overall survival (Kaplan–Meier curve available; HR not derived)	D6 Bias in measurement of outcomes	Low	OS ... only estimated for patients with available data	Corr et al. (2025) | PDF pp.2–4 | Outcome measurement	Serious	Death is objective, although missing vital-status data are handled in the missing-data domain.	Figure 3; complete result-level details retained in this table
Corr 2025	ROBINS-I 2016 2016 original cohort-type version (19 September 2016)	OS | Result-level assessment: Corr 2025: overall survival (Kaplan–Meier curve available; HR not derived)	D7 Bias in selection of the reported result	Moderate	No adjustments were made for multiple comparisons	Corr et al. (2025) | PDF pp.2,4 | Methods/Discussion	Serious	Multiple doses, time points, outcomes, and race subgroups were analyzed without a public prespecified plan.	Figure 3; complete result-level details retained in this table

Study	Tool	Outcome or result	Domain	Judgment	Support for judgment	Information source	Overall judgment	Additional rationale	Figure link
Corr 2025	ROBINS-I 2016 2016 original cohort-type version (19 September 2016)	OS | Result-level assessment: Corr 2025: overall survival (Kaplan–Meier curve available; HR not derived)	Overall Overall risk of bias	Serious	at least one domain was judged Serious	Corr et al. (2025) | See linked D1-D7 anchors | ROBINS-I overall algorithm	Serious	ROBINS-I 2016 overall judgment equals the highest domain when no domain is Critical.	Figure 3; complete result-level details retained in this table
Corr 2025	ROBINS-I 2016 2016 original cohort-type version (19 September 2016)	PFS | Result-level assessment: Corr 2025: progression-free survival (Kaplan–Meier curve available; HR not derived)	D1 Bias due to confounding	Serious	starting doses ... were made at the treating providers' discretion	Corr et al. (2025) | PDF pp.2–4 | Methods/Results/Discussion	Serious	Provider-selected dose, age imbalance, and absence of multivariable adjustment leave important confounding uncontrolled.	Figure 3; complete result-level details retained in this table
Corr 2025	ROBINS-I 2016 2016 original cohort-type version (19 September 2016)	PFS | Result-level assessment: Corr 2025: progression-free survival (Kaplan–Meier curve available; HR not derived)	D2 Bias in selection of participants into the study	Moderate	18 patients were excluded due to incomplete data	Corr et al. (2025) | PDF p.3 | Results/Patient characteristics	Serious	Post-identification incomplete-data exclusions and exclusion of uncommon dose groups may select the analyzed cohort.	Figure 3; complete result-level details retained in this table
Corr 2025	ROBINS-I 2016 2016 original cohort-type version (19 September 2016)	PFS | Result-level assessment: Corr 2025: progression-free survival (Kaplan–Meier curve available; HR not derived)	D3 Bias in classification of interventions	Low	Patients were categorized by lenvatinib starting doses	Corr et al. (2025) | PDF pp.2–3 | Methods/Regimen	Serious	Four starting-dose groups were explicitly defined from chart data at treatment initiation.	Figure 3; complete result-level details retained in this table
Corr 2025	ROBINS-I 2016 2016 original cohort-type version (19 September 2016)	PFS | Result-level assessment: Corr 2025: progression-free survival (Kaplan–Meier curve available; HR not derived)	D4 Bias due to deviations from intended interventions	Low	dose modifications ... were made at the treating providers' discretion	Corr et al. (2025) | PDF pp.2–3 | Methods/Regimen	Serious	For the assignment estimand, routine modifications reflect usual practice and were not used to redefine baseline exposure.	Figure 3; complete result-level details retained in this table
Corr 2025	ROBINS-I 2016 2016 original cohort-type version (19 September 2016)	PFS | Result-level assessment: Corr 2025: progression-free survival (Kaplan–Meier curve available; HR not derived)	D5 Bias due to missing data	Serious	PFS ... only estimated for patients with available data	Corr et al. (2025) | PDF pp.2–4,6 | Outcome availability/missingness	Serious	Consensus rationale: The number and distribution of participants with available survival data were not reported.	Figure 3; complete result-level details retained in this table
Corr 2025	ROBINS-I 2016 2016 original cohort-type version (19 September 2016)	PFS | Result-level assessment: Corr 2025: progression-free survival (Kaplan–Meier curve available; HR not derived)	D6 Bias in measurement of outcomes	Serious	PFS ... only estimated for patients with available data	Corr et al. (2025) | PDF pp.2–4,6 | Outcome measurement	Serious	Follow-up and imaging were nonuniform and no centralized radiology review was used.	Figure 3; complete result-level details retained in this table
Corr 2025	ROBINS-I 2016 2016 original cohort-type version (19 September 2016)	PFS | Result-level assessment: Corr 2025: progression-free survival (Kaplan–Meier curve available; HR not derived)	D7 Bias in selection of the reported result	Moderate	No adjustments were made for multiple comparisons	Corr et al. (2025) | PDF pp.2,4 | Methods/Discussion	Serious	Multiple doses, time points, outcomes, and race subgroups were analyzed without a public prespecified plan.	Figure 3; complete result-level details retained in this table
Corr 2025	ROBINS-I 2016 2016 original cohort-type version (19 September 2016)	PFS | Result-level assessment: Corr 2025: progression-free survival (Kaplan–Meier curve available; HR not derived)	Overall Overall risk of bias	Serious	at least one domain was judged Serious	Corr et al. (2025) | See linked D1-D7 anchors | ROBINS-I overall algorithm	Serious	ROBINS-I 2016 overall judgment equals the highest domain when no domain is Critical.	Figure 3; complete result-level details retained in this table
Corr 2025	ROBINS-I 2016 2016 original cohort-type version (19 September 2016)	Corr 2025: subsequent lenvatinib dose reduction (risk ratio)	D1 Bias due to confounding	Serious	starting doses ... were made at the treating providers' discretion	Corr et al. (2025) | PDF pp.2–4 | Methods/Results/Discussion	Serious	Provider-selected dose, age imbalance, and absence of multivariable adjustment leave important confounding uncontrolled.	Figure 3; complete result-level details retained in this table

Study	Tool	Outcome or result	Domain	Judgment	Support for judgment	Information source	Overall judgment	Additional rationale	Figure link
Corr 2025	ROBINS-I 2016 2016 original cohort-type version (19 September 2016)	Corr 2025: subsequent lenvatinib dose reduction (risk ratio)	D2 Bias in selection of participants into the study	Moderate	18 patients were excluded due to incomplete data	Corr et al. (2025) | PDF p.3 | Results/Patient characteristics	Serious	Post-identification incomplete-data exclusions and exclusion of uncommon dose groups may select the analyzed cohort.	Figure 3; complete result-level details retained in this table
Corr 2025	ROBINS-I 2016 2016 original cohort-type version (19 September 2016)	Corr 2025: subsequent lenvatinib dose reduction (risk ratio)	D3 Bias in classification of interventions	Low	Patients were categorized by lenvatinib starting doses	Corr et al. (2025) | PDF pp.2–3 | Methods/Regimen	Serious	Four starting-dose groups were explicitly defined from chart data at treatment initiation.	Figure 3; complete result-level details retained in this table
Corr 2025	ROBINS-I 2016 2016 original cohort-type version (19 September 2016)	Corr 2025: subsequent lenvatinib dose reduction (risk ratio)	D4 Bias due to deviations from intended interventions	Low	dose modifications ... were made at the treating providers' discretion	Corr et al. (2025) | PDF pp.2–3 | Methods/Regimen	Serious	For the assignment estimand, routine modifications reflect usual practice and were not used to redefine baseline exposure.	Figure 3; complete result-level details retained in this table
Corr 2025	ROBINS-I 2016 2016 original cohort-type version (19 September 2016)	Corr 2025: subsequent lenvatinib dose reduction (risk ratio)	D5 Bias due to missing data	Moderate	Dose reductions occurred in 84	Corr et al. (2025) | PDF pp.2–4 | Outcome availability/missingness	Serious	Counts were reported for the included cohort, but incomplete-data exclusions remain.	Figure 3; complete result-level details retained in this table
Corr 2025	ROBINS-I 2016 2016 original cohort-type version (19 September 2016)	Corr 2025: subsequent lenvatinib dose reduction (risk ratio)	D6 Bias in measurement of outcomes	Serious	Dose reductions occurred in 84	Corr et al. (2025) | PDF pp.2–4 | Outcome measurement	Serious	Provider-driven reductions are management decisions influenced by starting dose and perceived tolerability.	Figure 3; complete result-level details retained in this table
Corr 2025	ROBINS-I 2016 2016 original cohort-type version (19 September 2016)	Corr 2025: subsequent lenvatinib dose reduction (risk ratio)	D7 Bias in selection of the reported result	Moderate	No adjustments were made for multiple comparisons	Corr et al. (2025) | PDF pp.2,4 | Methods/Discussion	Serious	Multiple doses, time points, outcomes, and race subgroups were analyzed without a public prespecified plan.	Figure 3; complete result-level details retained in this table
Corr 2025	ROBINS-I 2016 2016 original cohort-type version (19 September 2016)	Corr 2025: subsequent lenvatinib dose reduction (risk ratio)	Overall Overall risk of bias	Serious	at least one domain was judged Serious	Corr et al. (2025) | See linked D1-D7 anchors | ROBINS-I overall algorithm	Serious	ROBINS-I 2016 overall judgment equals the highest domain when no domain is Critical.	Figure 3; complete result-level details retained in this table
Corr 2025	ROBINS-I 2016 2016 original cohort-type version (19 September 2016)	Corr 2025: disease control (risk ratio)	D1 Bias due to confounding	Serious	starting doses ... were made at the treating providers' discretion	Corr et al. (2025) | PDF pp.2–4 | Methods/Results/Discussion	Serious	Provider-selected dose, age imbalance, and absence of multivariable adjustment leave important confounding uncontrolled.	Figure 3; complete result-level details retained in this table
Corr 2025	ROBINS-I 2016 2016 original cohort-type version (19 September 2016)	Corr 2025: objective response (risk ratio)	D1 Bias due to confounding	Serious	starting doses ... were made at the treating providers' discretion	Corr et al. (2025) | PDF pp.2–4 | Methods/Results/Discussion	Serious	Provider-selected dose, age imbalance, and absence of multivariable adjustment leave important confounding uncontrolled.	Figure 3; complete result-level details retained in this table
Corr 2025	ROBINS-I 2016 2016 original cohort-type version (19 September 2016)	Corr 2025: disease control (risk ratio)	D2 Bias in selection of participants into the study	Moderate	18 patients were excluded due to incomplete data	Corr et al. (2025) | PDF p.3 | Results/Patient characteristics	Serious	Post-identification incomplete-data exclusions and exclusion of uncommon dose groups may select the analyzed cohort.	Figure 3; complete result-level details retained in this table
Corr 2025	ROBINS-I 2016 2016 original cohort-type version (19 September 2016)	Corr 2025: objective response (risk ratio)	D2 Bias in selection of participants into the study	Moderate	18 patients were excluded due to incomplete data	Corr et al. (2025) | PDF p.3 | Results/Patient characteristics	Serious	Post-identification incomplete-data exclusions and exclusion of uncommon dose groups may select the analyzed cohort.	Figure 3; complete result-level details retained in this table
Corr 2025	ROBINS-I 2016 2016 original cohort-type version (19 September 2016)	Corr 2025: disease control (risk ratio)	D3 Bias in classification of interventions	Low	Patients were categorized by lenvatinib starting doses	Corr et al. (2025) | PDF pp.2–3 | Methods/Regimen	Serious	Four starting-dose groups were explicitly defined from chart data at treatment initiation.	Figure 3; complete result-level details retained in this table
Corr 2025	ROBINS-I 2016 2016 original cohort-type version	Corr 2025: objective response (risk ratio)	D3 Bias in classification of interventions	Low	Patients were categorized by lenvatinib starting doses	Corr et al. (2025) | PDF pp.2–3 | Methods/Regimen	Serious	Four starting-dose groups were explicitly defined from	Figure 3; complete result-level details retained in this

Study	Tool	Outcome or result	Domain	Judgment	Support for judgment	Information source	Overall judgment	Additional rationale	Figure link
	(19 September 2016)							chart data at treatment initiation.	table
Corr 2025	ROBINS-I 2016 2016 original cohort-type version (19 September 2016)	Corr 2025: disease control (risk ratio)	D4 Bias due to deviations from intended interventions	Low	dose modifications ... were made at the treating providers' discretion	Corr et al. (2025) | PDF pp.2–3 | Methods/Regimen	Serious	For the assignment estimand, routine modifications reflect usual practice and were not used to redefine baseline exposure.	Figure 3; complete result-level details retained in this table
Corr 2025	ROBINS-I 2016 2016 original cohort-type version (19 September 2016)	Corr 2025: objective response (risk ratio)	D4 Bias due to deviations from intended interventions	Low	dose modifications ... were made at the treating providers' discretion	Corr et al. (2025) | PDF pp.2–3 | Methods/Regimen	Serious	For the assignment estimand, routine modifications reflect usual practice and were not used to redefine baseline exposure.	Figure 3; complete result-level details retained in this table
Corr 2025	ROBINS-I 2016 2016 original cohort-type version (19 September 2016)	Corr 2025: disease control (risk ratio)	D5 Bias due to missing data	Serious	Response to therapy was determined by provider or chart abstraction	Corr et al. (2025) | PDF pp.2–4,6 | Outcome availability/missingness	Serious	Consensus rationale: Incomplete-data exclusions and denominator/category gaps materially affect response availability.	Figure 3; complete result-level details retained in this table
Corr 2025	ROBINS-I 2016 2016 original cohort-type version (19 September 2016)	Corr 2025: objective response (risk ratio)	D5 Bias due to missing data	Serious	Response to therapy was determined by provider or chart abstraction	Corr et al. (2025) | PDF pp.2–4,6 | Outcome availability/missingness	Serious	Consensus rationale: Incomplete-data exclusions and denominator/category gaps materially affect response availability.	Figure 3; complete result-level details retained in this table
Corr 2025	ROBINS-I 2016 2016 original cohort-type version (19 September 2016)	Corr 2025: disease control (risk ratio)	D6 Bias in measurement of outcomes	Serious	Response to therapy was determined by provider or chart abstraction	Corr et al. (2025) | PDF pp.2–4,6 | Outcome measurement	Serious	No formal RECIST or centralized review was used; provider/chart assessment was unblinded.	Figure 3; complete result-level details retained in this table
Corr 2025	ROBINS-I 2016 2016 original cohort-type version (19 September 2016)	Corr 2025: objective response (risk ratio)	D6 Bias in measurement of outcomes	Serious	Response to therapy was determined by provider or chart abstraction	Corr et al. (2025) | PDF pp.2–4,6 | Outcome measurement	Serious	No formal RECIST or centralized review was used; provider/chart assessment was unblinded.	Figure 3; complete result-level details retained in this table
Corr 2025	ROBINS-I 2016 2016 original cohort-type version (19 September 2016)	Corr 2025: disease control (risk ratio)	D7 Bias in selection of the reported result	Moderate	No adjustments were made for multiple comparisons	Corr et al. (2025) | PDF pp.2,4 | Methods/Discussion	Serious	Multiple doses, time points, outcomes, and race subgroups were analyzed without a public prespecified plan.	Figure 3; complete result-level details retained in this table
Corr 2025	ROBINS-I 2016 2016 original cohort-type version (19 September 2016)	Corr 2025: objective response (risk ratio)	D7 Bias in selection of the reported result	Moderate	No adjustments were made for multiple comparisons	Corr et al. (2025) | PDF pp.2,4 | Methods/Discussion	Serious	Multiple doses, time points, outcomes, and race subgroups were analyzed without a public prespecified plan.	Figure 3; complete result-level details retained in this table
Corr 2025	ROBINS-I 2016 2016 original cohort-type version (19 September 2016)	Corr 2025: disease control (risk ratio)	Overall Overall risk of bias	Serious	at least one domain was judged Serious	Corr et al. (2025) | See linked D1-D7 anchors | ROBINS-I overall algorithm	Serious	ROBINS-I 2016 overall judgment equals the highest domain when no domain is Critical.	Figure 3; complete result-level details retained in this table
Corr 2025	ROBINS-I 2016 2016 original cohort-type version (19 September 2016)	Corr 2025: objective response (risk ratio)	Overall Overall risk of bias	Serious	at least one domain was judged Serious	Corr et al. (2025) | See linked D1-D7 anchors | ROBINS-I overall algorithm	Serious	ROBINS-I 2016 overall judgment equals the highest domain when no domain is Critical.	Figure 3; complete result-level details retained in this table
Boku 2026	ROBINS-I 2016 2016 original cohort-type version (19 September 2016)	Boku 2026: overall survival (adjusted HR)	D1 Bias due to confounding	Serious	confounding by indication	Boku et al. (2026) | PDF pp.5–7; Supplementary Table 4 | Results/limitations	Serious	Large imbalances in age, ECOG, nutrition and prior therapy remained; PFS adjustment included age only and OS modeling was sparse.	Figure 3; complete result-level details retained in this table
Boku 2026	ROBINS-I 2016 2016 original cohort-type version (19 September 2016)	Boku 2026: overall survival (adjusted HR)	D2 Bias in selection of participants into the study	Moderate	A total of 33 patients ... were included	Boku et al. (2026) | PDF pp.4–5 | Study design/Results	Serious	The report does not provide a complete screened-to-included flow, although follow-up was defined from LP initiation.	Figure 3; complete result-level details retained in this table

Study	Tool	Outcome or result	Domain	Judgment	Support for judgment	Information source	Overall judgment	Additional rationale	Figure link
Boku 2026	ROBINS-I 2016 2016 original cohort-type version (19 September 2016)	Boku 2026: overall survival (adjusted HR)	D3 Bias in classification of interventions	Low	treatment history including the starting dose	Boku et al. (2026) | PDF p.4 | Data collection	Serious	Starting dose was abstracted from contemporaneous medical records and the groups were explicit.	Figure 3; complete result-level details retained in this table
Boku 2026	ROBINS-I 2016 2016 original cohort-type version (19 September 2016)	Boku 2026: overall survival (adjusted HR)	D4 Bias due to deviations from intended interventions	Low	time from the initiation of LP therapy	Boku et al. (2026) | PDF p.4 | Endpoints	Serious	The target is assignment to starting dose; later usual-practice dose modifications do not redefine the group.	Figure 3; complete result-level details retained in this table
Boku 2026	ROBINS-I 2016 2016 original cohort-type version (19 September 2016)	Boku 2026: overall survival (adjusted HR)	D5 Bias due to missing data	Low	OS ... death from any cause	Boku et al. (2026) | PDF pp.4–7; Supplementary Table 3 | Outcome availability/missingness	Serious	Vital-status follow-up was reported for all participants.	Figure 3; complete result-level details retained in this table
Boku 2026	ROBINS-I 2016 2016 original cohort-type version (19 September 2016)	Boku 2026: overall survival (adjusted HR)	D6 Bias in measurement of outcomes	Low	OS ... death from any cause	Boku et al. (2026) | PDF pp.4–7; Supplementary Table 3 | Outcome measurement	Serious	Death is objective; model-selection concerns are assessed in D7.	Figure 3; complete result-level details retained in this table
Boku 2026	ROBINS-I 2016 2016 original cohort-type version (19 September 2016)	Boku 2026: overall survival (adjusted HR)	D7 Bias in selection of the reported result	Serious	univariate and multivariate analyses for prognostic factors	Boku et al. (2026) | PDF pp.5–7; Supplementary Tables 2–4 | Statistical analysis/limitations	Serious	Small-sample exploratory models and different covariate sets create multiple analysis choices without a public prespecified plan.	Figure 3; complete result-level details retained in this table
Boku 2026	ROBINS-I 2016 2016 original cohort-type version (19 September 2016)	Boku 2026: overall survival (adjusted HR)	Overall Overall risk of bias	Serious	at least one domain was judged Serious	Boku et al. (2026) | See linked D1-D7 anchors | ROBINS-I overall algorithm	Serious	ROBINS-I 2016 overall judgment equals the highest domain when no domain is Critical.	Figure 3; complete result-level details retained in this table
Boku 2026	ROBINS-I 2016 2016 original cohort-type version (19 September 2016)	Boku 2026: progression-free survival (adjusted HR)	D1 Bias due to confounding	Serious	confounding by indication	Boku et al. (2026) | PDF pp.5–7; Supplementary Table 4 | Results/limitations	Serious	Large imbalances in age, ECOG, nutrition and prior therapy remained; PFS adjustment included age only and OS modeling was sparse.	Figure 3; complete result-level details retained in this table
Boku 2026	ROBINS-I 2016 2016 original cohort-type version (19 September 2016)	Boku 2026: progression-free survival (adjusted HR)	D2 Bias in selection of participants into the study	Moderate	A total of 33 patients ... were included	Boku et al. (2026) | PDF pp.4–5 | Study design/Results	Serious	The report does not provide a complete screened-to-included flow, although follow-up was defined from LP initiation.	Figure 3; complete result-level details retained in this table
Boku 2026	ROBINS-I 2016 2016 original cohort-type version (19 September 2016)	Boku 2026: progression-free survival (adjusted HR)	D3 Bias in classification of interventions	Low	treatment history including the starting dose	Boku et al. (2026) | PDF p.4 | Data collection	Serious	Starting dose was abstracted from contemporaneous medical records and the groups were explicit.	Figure 3; complete result-level details retained in this table
Boku 2026	ROBINS-I 2016 2016 original cohort-type version (19 September 2016)	Boku 2026: progression-free survival (adjusted HR)	D4 Bias due to deviations from intended interventions	Low	time from the initiation of LP therapy	Boku et al. (2026) | PDF p.4 | Endpoints	Serious	The target is assignment to starting dose; later usual-practice dose modifications do not redefine the group.	Figure 3; complete result-level details retained in this table
Boku 2026	ROBINS-I 2016 2016 original cohort-type version (19 September 2016)	Boku 2026: progression-free survival (adjusted HR)	D5 Bias due to missing data	Low	multivariate analysis ... starting dose and age	Boku et al. (2026) | PDF pp.4–7; Supplementary Tables 2 and 4 | Outcome availability/missingness	Serious	PFS follow-up was reported for all participants.	Figure 3; complete result-level details retained in this table
Boku 2026	ROBINS-I 2016 2016 original cohort-type version (19 September 2016)	Boku 2026: progression-free survival (adjusted HR)	D6 Bias in measurement of outcomes	Moderate	multivariate analysis ... starting dose and age	Boku et al. (2026) | PDF pp.4–7; Supplementary Tables 2 and 4 | Outcome measurement	Serious	Progression was attending-physician assessed and blinding to dose was not reported.	Figure 3; complete result-level details retained in this table
Boku 2026	ROBINS-I 2016 2016 original cohort-type version (19 September 2016)	Boku 2026: progression-free survival (adjusted HR)	D7 Bias in selection of the reported result	Serious	univariate and multivariate analyses for prognostic factors	Boku et al. (2026) | PDF pp.5–7; Supplementary Tables 2–4 | Statistical analysis/limitations	Serious	Small-sample exploratory models and different covariate sets create multiple analysis choices without a public prespecified plan.	Figure 3; complete result-level details retained in this table

Study	Tool	Outcome or result	Domain	Judgment	Support for judgment	Information source	Overall judgment	Additional rationale	Figure link
Boku 2026	ROBINS-I 2016 2016 original cohort-type version (19 September 2016)	Boku 2026: progression-free survival (adjusted HR)	Overall Overall risk of bias	Serious	at least one domain was judged Serious	Boku et al. (2026) | See linked D1-D7 anchors | ROBINS-I overall algorithm	Serious	ROBINS-I 2016 overall judgment equals the highest domain when no domain is Critical.	Figure 3; complete result-level details retained in this table
Boku 2026	ROBINS-I 2016 2016 original cohort-type version (19 September 2016)	Boku 2026: disease control (risk ratio)	D1 Bias due to confounding	Serious	confounding by indication	Boku et al. (2026) | PDF pp.5–7; Supplementary Table 4 | Results/limitations	Serious	Large imbalances in age, ECOG, nutrition and prior therapy remained; PFS adjustment included age only and OS modeling was sparse.	Figure 3; complete result-level details retained in this table
Boku 2026	ROBINS-I 2016 2016 original cohort-type version (19 September 2016)	Boku 2026: objective response (risk ratio)	D1 Bias due to confounding	Serious	confounding by indication	Boku et al. (2026) | PDF pp.5–7; Supplementary Table 4 | Results/limitations	Serious	Large imbalances in age, ECOG, nutrition and prior therapy remained; PFS adjustment included age only and OS modeling was sparse.	Figure 3; complete result-level details retained in this table
Boku 2026	ROBINS-I 2016 2016 original cohort-type version (19 September 2016)	Boku 2026: disease control (risk ratio)	D2 Bias in selection of participants into the study	Moderate	A total of 33 patients ... were included	Boku et al. (2026) | PDF pp.4–5 | Study design/Results	Serious	The report does not provide a complete screened-to-included flow, although follow-up was defined from LP initiation.	Figure 3; complete result-level details retained in this table
Boku 2026	ROBINS-I 2016 2016 original cohort-type version (19 September 2016)	Boku 2026: objective response (risk ratio)	D2 Bias in selection of participants into the study	Moderate	A total of 33 patients ... were included	Boku et al. (2026) | PDF pp.4–5 | Study design/Results	Serious	The report does not provide a complete screened-to-included flow, although follow-up was defined from LP initiation.	Figure 3; complete result-level details retained in this table
Boku 2026	ROBINS-I 2016 2016 original cohort-type version (19 September 2016)	Boku 2026: disease control (risk ratio)	D3 Bias in classification of interventions	Low	treatment history including the starting dose	Boku et al. (2026) | PDF p.4 | Data collection	Serious	Starting dose was abstracted from contemporaneous medical records and the groups were explicit.	Figure 3; complete result-level details retained in this table
Boku 2026	ROBINS-I 2016 2016 original cohort-type version (19 September 2016)	Boku 2026: objective response (risk ratio)	D3 Bias in classification of interventions	Low	treatment history including the starting dose	Boku et al. (2026) | PDF p.4 | Data collection	Serious	Starting dose was abstracted from contemporaneous medical records and the groups were explicit.	Figure 3; complete result-level details retained in this table
Boku 2026	ROBINS-I 2016 2016 original cohort-type version (19 September 2016)	Boku 2026: disease control (risk ratio)	D4 Bias due to deviations from intended interventions	Low	time from the initiation of LP therapy	Boku et al. (2026) | PDF p.4 | Endpoints	Serious	The target is assignment to starting dose; later usual-practice dose modifications do not redefine the group.	Figure 3; complete result-level details retained in this table
Boku 2026	ROBINS-I 2016 2016 original cohort-type version (19 September 2016)	Boku 2026: objective response (risk ratio)	D4 Bias due to deviations from intended interventions	Low	time from the initiation of LP therapy	Boku et al. (2026) | PDF p.4 | Endpoints	Serious	The target is assignment to starting dose; later usual-practice dose modifications do not redefine the group.	Figure 3; complete result-level details retained in this table
Boku 2026	ROBINS-I 2016 2016 original cohort-type version (19 September 2016)	Boku 2026: disease control (risk ratio)	D5 Bias due to missing data	Low	33 patients ... included	Boku et al. (2026) | PDF pp.4–6; Supplementary Table 1 | Outcome availability/missingness	Serious	Response categories were reported for all 33 participants.	Figure 3; complete result-level details retained in this table
Boku 2026	ROBINS-I 2016 2016 original cohort-type version (19 September 2016)	Boku 2026: objective response (risk ratio)	D5 Bias due to missing data	Low	33 patients ... included	Boku et al. (2026) | PDF pp.4–6; Supplementary Table 1 | Outcome availability/missingness	Serious	Response categories were reported for all 33 participants.	Figure 3; complete result-level details retained in this table
Boku 2026	ROBINS-I 2016 2016 original cohort-type version (19 September 2016)	Boku 2026: disease control (risk ratio)	D6 Bias in measurement of outcomes	Moderate	33 patients ... included	Boku et al. (2026) | PDF pp.4–6; Supplementary Table 1 | Outcome measurement	Serious	Attending physicians used RECIST 1.1, but blinded or independent review was not reported.	Figure 3; complete result-level details retained in this table
Boku 2026	ROBINS-I 2016 2016 original cohort-type version (19 September 2016)	Boku 2026: objective response (risk ratio)	D6 Bias in measurement of outcomes	Moderate	33 patients ... included	Boku et al. (2026) | PDF pp.4–6; Supplementary Table 1 | Outcome measurement	Serious	Attending physicians used RECIST 1.1, but blinded or independent review was not reported.	Figure 3; complete result-level details retained in this table

Study	Tool	Outcome or result	Domain	Judgment	Support for judgment	Information source	Overall judgment	Additional rationale	Figure link
Boku 2026	ROBINS-I 2016 2016 original cohort-type version (19 September 2016)	Boku 2026: disease control (risk ratio)	D7 Bias in selection of the reported result	Moderate	univariate and multivariate analyses for prognostic factors	Boku et al. (2026) | PDF pp.5–7; Supplementary Tables 2–4 | Statistical analysis/limitations	Serious	Small-sample exploratory models and different covariate sets create multiple analysis choices without a public prespecified plan.	Figure 3; complete result-level details retained in this table
Boku 2026	ROBINS-I 2016 2016 original cohort-type version (19 September 2016)	Boku 2026: objective response (risk ratio)	D7 Bias in selection of the reported result	Moderate	univariate and multivariate analyses for prognostic factors	Boku et al. (2026) | PDF pp.5–7; Supplementary Tables 2–4 | Statistical analysis/limitations	Serious	Small-sample exploratory models and different covariate sets create multiple analysis choices without a public prespecified plan.	Figure 3; complete result-level details retained in this table
Boku 2026	ROBINS-I 2016 2016 original cohort-type version (19 September 2016)	Boku 2026: disease control (risk ratio)	Overall Overall risk of bias	Serious	at least one domain was judged Serious	Boku et al. (2026) | See linked D1-D7 anchors | ROBINS-I overall algorithm	Serious	ROBINS-I 2016 overall judgment equals the highest domain when no domain is Critical.	Figure 3; complete result-level details retained in this table
Boku 2026	ROBINS-I 2016 2016 original cohort-type version (19 September 2016)	Boku 2026: objective response (risk ratio)	Overall Overall risk of bias	Serious	at least one domain was judged Serious	Boku et al. (2026) | See linked D1-D7 anchors | ROBINS-I overall algorithm	Serious	ROBINS-I 2016 overall judgment equals the highest domain when no domain is Critical.	Figure 3; complete result-level details retained in this table
Barbi 2025	ROBINS-I 2016 2016 original cohort-type version (19 September 2016)	Barbi 2025: result (risk ratio)	D1 Bias due to confounding	Serious	clinician-guided based on age, performance status, and anticipated tolerability	Barbi et al. (2025) | PDF p.3 | Statistical analysis/Results	Serious	Dose was explicitly chosen using prognostic factors; backward elimination did not retain all important confounders.	Figure 3; complete result-level details retained in this table
Barbi 2025	ROBINS-I 2016 2016 original cohort-type version (19 September 2016)	Barbi 2025: result (risk ratio)	D2 Bias in selection of participants into the study	Serious	received at least three cycles	Barbi et al. (2025) | PDF p.2 | Patients and data collection	Serious	Requiring three cycles selects on post-initiation survival/tolerability and can exclude early toxicity or progression related to dose and outcome.	Figure 3; complete result-level details retained in this table
Barbi 2025	ROBINS-I 2016 2016 original cohort-type version (19 September 2016)	Barbi 2025: result (risk ratio)	D3 Bias in classification of interventions	Low	stratified into reduced and higher-dose groups	Barbi et al. (2025) | PDF pp.2–3 | Methods/Results	Serious	Baseline dose was recorded in the medical record and threshold groups were clearly defined.	Figure 3; complete result-level details retained in this table
Barbi 2025	ROBINS-I 2016 2016 original cohort-type version (19 September 2016)	Barbi 2025: result (risk ratio)	D4 Bias due to deviations from intended interventions	Low	starting dose ... pragmatic surrogate	Barbi et al. (2025) | PDF p.2 | Methods	Serious	The assignment effect uses initial dose; routine later changes were treated as outcomes.	Figure 3; complete result-level details retained in this table
Barbi 2025	ROBINS-I 2016 2016 original cohort-type version (19 September 2016)	Barbi 2025: result (risk ratio)	D5 Bias due to missing data	Moderate	Grade >=2 AEs were reported	Barbi et al. (2025) | PDF pp.2–5 | Outcome availability/missingness	Serious	Retrospective records may incompletely capture adverse events.	Figure 3; complete result-level details retained in this table
Barbi 2025	ROBINS-I 2016 2016 original cohort-type version (19 September 2016)	Barbi 2025: result (risk ratio)	D6 Bias in measurement of outcomes	Serious	Grade >=2 AEs were reported	Barbi et al. (2025) | PDF pp.2–5 | Outcome measurement	Serious	Unblinded CTCAE/clinical documentation and clinician decisions can differ by dose group.	Figure 3; complete result-level details retained in this table
Barbi 2025	ROBINS-I 2016 2016 original cohort-type version (19 September 2016)	Barbi 2025: result (risk ratio)	D7 Bias in selection of the reported result	Moderate	backward elimination procedure	Barbi et al. (2025) | PDF p.3; 2023 ASCO companion | Statistical analysis	Serious	Data-driven model reduction and an expanding cohort create multiple analysis choices; the final report was preferred.	Figure 3; complete result-level details retained in this table
Barbi 2025	ROBINS-I 2016 2016 original cohort-type version (19 September 2016)	Barbi 2025: result (risk ratio)	Overall Overall risk of bias	Serious	at least one domain was judged Serious	Barbi et al. (2025) | See linked D1-D7 anchors | ROBINS-I overall algorithm	Serious	ROBINS-I 2016 overall judgment equals the highest domain when no domain is Critical.	Figure 3; complete result-level details retained in this table
Barbi 2025	ROBINS-I 2016 2016 original cohort-type version (19 September 2016)	Barbi 2025: progression-free survival (adjusted HR)	D1 Bias due to confounding	Serious	clinician-guided based on age, performance status, and anticipated tolerability	Barbi et al. (2025) | PDF p.3 | Statistical analysis/Results	Serious	Dose was explicitly chosen using prognostic factors; backward elimination did not	Figure 3; complete result-level details retained in this table

Study	Tool	Outcome or result	Domain	Judgment	Support for judgment	Information source	Overall judgment	Additional rationale	Figure link
								retain all important confounders.	
Barbi 2025	ROBINS-I 2016 2016 original cohort-type version (19 September 2016)	Barbi 2025: progression-free survival (adjusted HR)	D2 Bias in selection of participants into the study	Serious	received at least three cycles	Barbi et al. (2025) | PDF p.2 | Patients and data collection	Serious	Requiring three cycles selects on post-initiation survival/tolerability and can exclude early toxicity or progression related to dose and outcome.	Figure 3; complete result-level details retained in this table
Barbi 2025	ROBINS-I 2016 2016 original cohort-type version (19 September 2016)	Barbi 2025: progression-free survival (adjusted HR)	D3 Bias in classification of interventions	Low	stratified into reduced and higher-dose groups	Barbi et al. (2025) | PDF pp.2–3 | Methods/Results	Serious	Baseline dose was recorded in the medical record and threshold groups were clearly defined.	Figure 3; complete result-level details retained in this table
Barbi 2025	ROBINS-I 2016 2016 original cohort-type version (19 September 2016)	Barbi 2025: progression-free survival (adjusted HR)	D4 Bias due to deviations from intended interventions	Low	starting dose ... pragmatic surrogate	Barbi et al. (2025) | PDF p.2 | Methods	Serious	The assignment effect uses initial dose; routine later changes were treated as outcomes.	Figure 3; complete result-level details retained in this table
Barbi 2025	ROBINS-I 2016 2016 original cohort-type version (19 September 2016)	Barbi 2025: progression-free survival (adjusted HR)	D5 Bias due to missing data	Moderate	progression or death was determined based on treating physician documentation	Barbi et al. (2025) | PDF pp.2–3,6–7 | Outcome availability/missingness	Serious	Incomplete capture and minimum-three-cycle eligibility can remove early outcomes.	Figure 3; complete result-level details retained in this table
Barbi 2025	ROBINS-I 2016 2016 original cohort-type version (19 September 2016)	Barbi 2025: progression-free survival (adjusted HR)	D6 Bias in measurement of outcomes	Serious	progression or death was determined based on treating physician documentation	Barbi et al. (2025) | PDF pp.2–3,6–7 | Outcome measurement	Serious	Treating-physician documentation and nonstandardized imaging can be influenced by known dose.	Figure 3; complete result-level details retained in this table
Barbi 2025	ROBINS-I 2016 2016 original cohort-type version (19 September 2016)	Barbi 2025: progression-free survival (adjusted HR)	D7 Bias in selection of the reported result	Serious	backward elimination procedure	Barbi et al. (2025) | PDF p.3; 2023 ASCO companion | Statistical analysis	Serious	Data-driven model reduction and an expanding cohort create multiple analysis choices; the final report was preferred.	Figure 3; complete result-level details retained in this table
Barbi 2025	ROBINS-I 2016 2016 original cohort-type version (19 September 2016)	Barbi 2025: progression-free survival (adjusted HR)	Overall Overall risk of bias	Serious	at least one domain was judged Serious	Barbi et al. (2025) | See linked D1-D7 anchors | ROBINS-I overall algorithm	Serious	ROBINS-I 2016 overall judgment equals the highest domain when no domain is Critical.	Figure 3; complete result-level details retained in this table
Barbi 2025	ROBINS-I 2016 2016 original cohort-type version (19 September 2016)	Barbi 2025: treatment discontinuation toxicity-attributed outcome definition	D1 Bias due to confounding	Serious	clinician-guided based on age, performance status, and anticipated tolerability	Barbi et al. (2025) | PDF p.3 | Statistical analysis/Results	Serious	Dose was explicitly chosen using prognostic factors; backward elimination did not retain all important confounders.	Figure 3; complete result-level details retained in this table
Barbi 2025	ROBINS-I 2016 2016 original cohort-type version (19 September 2016)	Barbi 2025: treatment interruption (risk ratio)	D1 Bias due to confounding	Serious	clinician-guided based on age, performance status, and anticipated tolerability	Barbi et al. (2025) | PDF p.3 | Statistical analysis/Results	Serious	Dose was explicitly chosen using prognostic factors; backward elimination did not retain all important confounders.	Figure 3; complete result-level details retained in this table
Barbi 2025	ROBINS-I 2016 2016 original cohort-type version (19 September 2016)	Barbi 2025: subsequent lenvatinib dose reduction (risk ratio)	D1 Bias due to confounding	Serious	clinician-guided based on age, performance status, and anticipated tolerability	Barbi et al. (2025) | PDF p.3 | Statistical analysis/Results	Serious	Dose was explicitly chosen using prognostic factors; backward elimination did not retain all important confounders.	Figure 3; complete result-level details retained in this table
Barbi 2025	ROBINS-I 2016 2016 original cohort-type version (19 September 2016)	Barbi 2025: treatment discontinuation toxicity-attributed outcome definition	D2 Bias in selection of participants into the study	Serious	received at least three cycles	Barbi et al. (2025) | PDF p.2 | Patients and data collection	Serious	Requiring three cycles selects on post-initiation survival/tolerability and can exclude early toxicity or progression related to dose and outcome.	Figure 3; complete result-level details retained in this table
Barbi 2025	ROBINS-I 2016 2016 original cohort-type version	Barbi 2025: treatment interruption (risk ratio)	D2 Bias in selection of participants into the study	Serious	received at least three cycles	Barbi et al. (2025) | PDF p.2 | Patients and data collection	Serious	Requiring three cycles selects on post-initiation	Figure 3; complete result-level details retained in this

Study	Tool	Outcome or result	Domain	Judgment	Support for judgment	Information source	Overall judgment	Additional rationale	Figure link
	(19 September 2016)							survival/tolerability and can exclude early toxicity or progression related to dose and outcome.	table
Barbi 2025	ROBINS-I 2016 2016 original cohort-type version (19 September 2016)	Barbi 2025: subsequent lenvatinib dose reduction (risk ratio)	D2 Bias in selection of participants into the study	Serious	received at least three cycles	Barbi et al. (2025) | PDF p.2 | Patients and data collection	Serious	Requiring three cycles selects on post-initiation survival/tolerability and can exclude early toxicity or progression related to dose and outcome.	Figure 3; complete result-level details retained in this table
Barbi 2025	ROBINS-I 2016 2016 original cohort-type version (19 September 2016)	Barbi 2025: treatment discontinuation toxicity-attributed outcome definition	D3 Bias in classification of interventions	Low	stratified into reduced and higher-dose groups	Barbi et al. (2025) | PDF pp.2–3 | Methods/Results	Serious	Baseline dose was recorded in the medical record and threshold groups were clearly defined.	Figure 3; complete result-level details retained in this table
Barbi 2025	ROBINS-I 2016 2016 original cohort-type version (19 September 2016)	Barbi 2025: treatment interruption (risk ratio)	D3 Bias in classification of interventions	Low	stratified into reduced and higher-dose groups	Barbi et al. (2025) | PDF pp.2–3 | Methods/Results	Serious	Baseline dose was recorded in the medical record and threshold groups were clearly defined.	Figure 3; complete result-level details retained in this table
Barbi 2025	ROBINS-I 2016 2016 original cohort-type version (19 September 2016)	Barbi 2025: subsequent lenvatinib dose reduction (risk ratio)	D3 Bias in classification of interventions	Low	stratified into reduced and higher-dose groups	Barbi et al. (2025) | PDF pp.2–3 | Methods/Results	Serious	Baseline dose was recorded in the medical record and threshold groups were clearly defined.	Figure 3; complete result-level details retained in this table
Barbi 2025	ROBINS-I 2016 2016 original cohort-type version (19 September 2016)	Barbi 2025: treatment discontinuation toxicity-attributed outcome definition	D4 Bias due to deviations from intended interventions	Low	starting dose ... pragmatic surrogate	Barbi et al. (2025) | PDF p.2 | Methods	Serious	The assignment effect uses initial dose; routine later changes were treated as outcomes.	Figure 3; complete result-level details retained in this table
Barbi 2025	ROBINS-I 2016 2016 original cohort-type version (19 September 2016)	Barbi 2025: treatment interruption (risk ratio)	D4 Bias due to deviations from intended interventions	Low	starting dose ... pragmatic surrogate	Barbi et al. (2025) | PDF p.2 | Methods	Serious	The assignment effect uses initial dose; routine later changes were treated as outcomes.	Figure 3; complete result-level details retained in this table
Barbi 2025	ROBINS-I 2016 2016 original cohort-type version (19 September 2016)	Barbi 2025: subsequent lenvatinib dose reduction (risk ratio)	D4 Bias due to deviations from intended interventions	Low	starting dose ... pragmatic surrogate	Barbi et al. (2025) | PDF p.2 | Methods	Serious	The assignment effect uses initial dose; routine later changes were treated as outcomes.	Figure 3; complete result-level details retained in this table
Barbi 2025	ROBINS-I 2016 2016 original cohort-type version (19 September 2016)	Barbi 2025: treatment discontinuation toxicity-attributed outcome definition	D5 Bias due to missing data	Moderate	AEs necessitating dose modifications	Barbi et al. (2025) | PDF pp.2–5 | Outcome availability/missingness	Serious	Three-cycle eligibility and chart capture can miss early process outcomes.	Figure 3; complete result-level details retained in this table
Barbi 2025	ROBINS-I 2016 2016 original cohort-type version (19 September 2016)	Barbi 2025: treatment interruption (risk ratio)	D5 Bias due to missing data	Moderate	AEs necessitating dose modifications	Barbi et al. (2025) | PDF pp.2–5 | Outcome availability/missingness	Serious	Three-cycle eligibility and chart capture can miss early process outcomes.	Figure 3; complete result-level details retained in this table
Barbi 2025	ROBINS-I 2016 2016 original cohort-type version (19 September 2016)	Barbi 2025: subsequent lenvatinib dose reduction (risk ratio)	D5 Bias due to missing data	Moderate	AEs necessitating dose modifications	Barbi et al. (2025) | PDF pp.2–5 | Outcome availability/missingness	Serious	Three-cycle eligibility and chart capture can miss early process outcomes.	Figure 3; complete result-level details retained in this table
Barbi 2025	ROBINS-I 2016 2016 original cohort-type version (19 September 2016)	Barbi 2025: treatment discontinuation toxicity-attributed outcome definition	D6 Bias in measurement of outcomes	Serious	AEs necessitating dose modifications	Barbi et al. (2025) | PDF pp.2–5 | Outcome measurement	Serious	These are clinician-driven management outcomes with known dose and anticipated tolerability.	Figure 3; complete result-level details retained in this table
Barbi 2025	ROBINS-I 2016 2016 original cohort-type version (19 September 2016)	Barbi 2025: treatment interruption (risk ratio)	D6 Bias in measurement of outcomes	Serious	AEs necessitating dose modifications	Barbi et al. (2025) | PDF pp.2–5 | Outcome measurement	Serious	These are clinician-driven management outcomes with known dose and anticipated tolerability.	Figure 3; complete result-level details retained in this table
Barbi 2025	ROBINS-I 2016 original cohort-type version (19 September 2016)	Barbi 2025: subsequent lenvatinib dose reduction (risk ratio)	D6 Bias in measurement of outcomes	Serious	AEs necessitating dose modifications	Barbi et al. (2025) | PDF pp.2–5 | Outcome measurement	Serious	These are clinician-driven management outcomes with known dose and anticipated	Figure 3; complete result-level details retained in this table

Study	Tool	Outcome or result	Domain	Judgment	Support for judgment	Information source	Overall judgment	Additional rationale	Figure link
								tolerability.	
Barbi 2025	ROBINS-I 2016 2016 original cohort-type version (19 September 2016)	Barbi 2025: treatment discontinuation toxicity-attributed outcome definition	D7 Bias in selection of the reported result	Moderate	backward elimination procedure	Barbi et al. (2025) | PDF p.3; 2023 ASCO companion | Statistical analysis	Serious	Data-driven model reduction and an expanding cohort create multiple analysis choices; the final report was preferred.	Figure 3; complete result-level details retained in this table
Barbi 2025	ROBINS-I 2016 2016 original cohort-type version (19 September 2016)	Barbi 2025: treatment interruption (risk ratio)	D7 Bias in selection of the reported result	Moderate	backward elimination procedure	Barbi et al. (2025) | PDF p.3; 2023 ASCO companion | Statistical analysis	Serious	Data-driven model reduction and an expanding cohort create multiple analysis choices; the final report was preferred.	Figure 3; complete result-level details retained in this table
Barbi 2025	ROBINS-I 2016 2016 original cohort-type version (19 September 2016)	Barbi 2025: subsequent lenvatinib dose reduction (risk ratio)	D7 Bias in selection of the reported result	Moderate	backward elimination procedure	Barbi et al. (2025) | PDF p.3; 2023 ASCO companion | Statistical analysis	Serious	Data-driven model reduction and an expanding cohort create multiple analysis choices; the final report was preferred.	Figure 3; complete result-level details retained in this table
Barbi 2025	ROBINS-I 2016 2016 original cohort-type version (19 September 2016)	Barbi 2025: treatment discontinuation toxicity-attributed outcome definition	Overall Overall risk of bias	Serious	at least one domain was judged Serious	Barbi et al. (2025) | See linked D1-D7 anchors | ROBINS-I overall algorithm	Serious	ROBINS-I 2016 overall judgment equals the highest domain when no domain is Critical.	Figure 3; complete result-level details retained in this table
Barbi 2025	ROBINS-I 2016 2016 original cohort-type version (19 September 2016)	Barbi 2025: treatment interruption (risk ratio)	Overall Overall risk of bias	Serious	at least one domain was judged Serious	Barbi et al. (2025) | See linked D1-D7 anchors | ROBINS-I overall algorithm	Serious	ROBINS-I 2016 overall judgment equals the highest domain when no domain is Critical.	Figure 3; complete result-level details retained in this table
Barbi 2025	ROBINS-I 2016 2016 original cohort-type version (19 September 2016)	Barbi 2025: subsequent lenvatinib dose reduction (risk ratio)	Overall Overall risk of bias	Serious	at least one domain was judged Serious	Barbi et al. (2025) | See linked D1-D7 anchors | ROBINS-I overall algorithm	Serious	ROBINS-I 2016 overall judgment equals the highest domain when no domain is Critical.	Figure 3; complete result-level details retained in this table
Dzienny 2026	ROBINS-I 2016 2016 original cohort-type version (19 September 2016)	Dzienny 2026: treatment discontinuation (risk ratio)	D1 Bias due to confounding	Serious	dose ... based on clinical judgment ... ECOG ... anticipated ability to tolerate	Dzienny et al. (2026) | PDF pp.2,7; Supplementary Table S1 | Methods/limitations	Serious	Dose selection used strong prognostic factors and dose groups remained substantially imbalanced despite exploratory matching.	Figure 3; complete result-level details retained in this table
Dzienny 2026	ROBINS-I 2016 2016 original cohort-type version (19 September 2016)	Dzienny 2026: treatment discontinuation toxicity-attributed outcome definition	D1 Bias due to confounding	Serious	dose ... based on clinical judgment ... ECOG ... anticipated ability to tolerate	Dzienny et al. (2026) | PDF pp.2,7; Supplementary Table S1 | Methods/limitations	Serious	Dose selection used strong prognostic factors and dose groups remained substantially imbalanced despite exploratory matching.	Figure 3; complete result-level details retained in this table
Dzienny 2026	ROBINS-I 2016 2016 original cohort-type version (19 September 2016)	Dzienny 2026: treatment discontinuation (risk ratio)	D2 Bias in selection of participants into the study	Moderate	excluded if they had incomplete medical records	Dzienny et al. (2026) | PDF p.2 | Patient population	Serious	Incomplete-record exclusions were possible, but treatment and follow-up began at combination initiation for included patients.	Figure 3; complete result-level details retained in this table
Dzienny 2026	ROBINS-I 2016 2016 original cohort-type version (19 September 2016)	Dzienny 2026: treatment discontinuation toxicity-attributed outcome definition	D2 Bias in selection of participants into the study	Moderate	excluded if they had incomplete medical records	Dzienny et al. (2026) | PDF p.2 | Patient population	Serious	Incomplete-record exclusions were possible, but treatment and follow-up began at combination initiation for included patients.	Figure 3; complete result-level details retained in this table
Dzienny 2026	ROBINS-I 2016 2016 original cohort-type version (19 September 2016)	Dzienny 2026: treatment discontinuation (risk ratio)	D3 Bias in classification of interventions	Low	dose-stratified analyses ... <=10 mg vs >10 mg	Dzienny et al. (2026) | PDF pp.2–3 | Clinical data/Statistical analysis	Serious	Initial dose was clearly recorded and threshold groups were defined before outcome assessment.	Figure 3; complete result-level details retained in this table
Dzienny 2026	ROBINS-I 2016 2016 original cohort-type version (19 September 2016)	Dzienny 2026: treatment discontinuation toxicity-attributed outcome definition	D3 Bias in classification of interventions	Low	dose-stratified analyses ... <=10 mg vs >10 mg	Dzienny et al. (2026) | PDF pp.2–3 | Clinical data/Statistical analysis	Serious	Initial dose was clearly recorded and threshold groups were defined before outcome assessment.	Figure 3; complete result-level details retained in this table

Study	Tool	Outcome or result	Domain	Judgment	Support for judgment	Information source	Overall judgment	Additional rationale	Figure link
Dzienny 2026	ROBINS-I 2016 2016 original cohort-type version (19 September 2016)	Dzienny 2026: treatment discontinuation (risk ratio)	D4 Bias due to deviations from intended interventions	Low	starting lenvatinib dose	Dzienny et al. (2026) | PDF pp.2–3 | Methods	Serious	The prespecified threshold estimand is baseline dose assignment; later routine modifications are downstream outcomes.	Figure 3; complete result-level details retained in this table
Dzienny 2026	ROBINS-I 2016 2016 original cohort-type version (19 September 2016)	Dzienny 2026: treatment discontinuation toxicity-attributed outcome definition	D4 Bias due to deviations from intended interventions	Low	starting lenvatinib dose	Dzienny et al. (2026) | PDF pp.2–3 | Methods	Serious	The prespecified threshold estimand is baseline dose assignment; later routine modifications are downstream outcomes.	Figure 3; complete result-level details retained in this table
Dzienny 2026	ROBINS-I 2016 2016 original cohort-type version (19 September 2016)	Dzienny 2026: treatment discontinuation (risk ratio)	D5 Bias due to missing data	Moderate	lack of systematic documentation of toxicity	Dzienny et al. (2026) | PDF pp.3–4,7; Supplementary Table S6 | Outcome availability/missingness	Serious	Toxicity documentation completeness was not established.	Figure 3; complete result-level details retained in this table
Dzienny 2026	ROBINS-I 2016 2016 original cohort-type version (19 September 2016)	Dzienny 2026: treatment discontinuation toxicity-attributed outcome definition	D5 Bias due to missing data	Moderate	lack of systematic documentation of toxicity	Dzienny et al. (2026) | PDF pp.3–4,7; Supplementary Table S6 | Outcome availability/missingness	Serious	Toxicity documentation completeness was not established.	Figure 3; complete result-level details retained in this table
Dzienny 2026	ROBINS-I 2016 2016 original cohort-type version (19 September 2016)	Dzienny 2026: treatment discontinuation (risk ratio)	D6 Bias in measurement of outcomes	Serious	lack of systematic documentation of toxicity	Dzienny et al. (2026) | PDF pp.3–4,7; Supplementary Table S6 | Outcome measurement	Serious	Symptoms and discontinuation attribution were subjective and clinician determined with dose known.	Figure 3; complete result-level details retained in this table
Dzienny 2026	ROBINS-I 2016 2016 original cohort-type version (19 September 2016)	Dzienny 2026: treatment discontinuation toxicity-attributed outcome definition	D6 Bias in measurement of outcomes	Serious	lack of systematic documentation of toxicity	Dzienny et al. (2026) | PDF pp.3–4,7; Supplementary Table S6 | Outcome measurement	Serious	Symptoms and discontinuation attribution were subjective and clinician determined with dose known.	Figure 3; complete result-level details retained in this table
Dzienny 2026	ROBINS-I 2016 2016 original cohort-type version (19 September 2016)	Dzienny 2026: treatment discontinuation (risk ratio)	D7 Bias in selection of the reported result	Serious	exploratory non-inferiority framework	Dzienny et al. (2026) | PDF pp.3–4; Supplementary Figures S3-S4 | Statistical analysis/Results	Serious	Consensus rationale: Multiple dose cutoffs, post hoc noninferiority, matching, and subgroup analyses create a high risk of result selection.	Figure 3; complete result-level details retained in this table
Dzienny 2026	ROBINS-I 2016 2016 original cohort-type version (19 September 2016)	Dzienny 2026: treatment discontinuation toxicity-attributed outcome definition	D7 Bias in selection of the reported result	Serious	exploratory non-inferiority framework	Dzienny et al. (2026) | PDF pp.3–4; Supplementary Figures S3-S4 | Statistical analysis/Results	Serious	Consensus rationale: Multiple dose cutoffs, post hoc noninferiority, matching, and subgroup analyses create a high risk of result selection.	Figure 3; complete result-level details retained in this table
Dzienny 2026	ROBINS-I 2016 2016 original cohort-type version (19 September 2016)	Dzienny 2026: treatment discontinuation (risk ratio)	Overall Overall risk of bias	Serious	at least one domain was judged Serious	Dzienny et al. (2026) | See linked D1-D7 anchors | ROBINS-I overall algorithm	Serious	ROBINS-I 2016 overall judgment equals the highest domain when no domain is Critical.	Figure 3; complete result-level details retained in this table
Dzienny 2026	ROBINS-I 2016 2016 original cohort-type version (19 September 2016)	Dzienny 2026: treatment discontinuation toxicity-attributed outcome definition	Overall Overall risk of bias	Serious	at least one domain was judged Serious	Dzienny et al. (2026) | See linked D1-D7 anchors | ROBINS-I overall algorithm	Serious	ROBINS-I 2016 overall judgment equals the highest domain when no domain is Critical.	Figure 3; complete result-level details retained in this table
Dzienny 2026	ROBINS-I 2016 2016 original cohort-type version (19 September 2016)	Dzienny 2026: progression-free survival (reported unadjusted HR)	D1 Bias due to confounding	Serious	dose ... based on clinical judgment ... ECOG ... anticipated ability to tolerate	Dzienny et al. (2026) | PDF pp.2,7; Supplementary Table S1 | Methods/limitations	Serious	Dose selection used strong prognostic factors and dose groups remained substantially imbalanced despite exploratory matching.	Figure 3; complete result-level details retained in this table
Dzienny 2026	ROBINS-I 2016 2016 original cohort-type version (19 September 2016)	Dzienny 2026: progression-free survival (reported unadjusted HR)	D2 Bias in selection of participants into the study	Moderate	excluded if they had incomplete medical records	Dzienny et al. (2026) | PDF p.2 | Patient population	Serious	Incomplete-record exclusions were possible, but treatment and follow-up began at combination initiation for included patients.	Figure 3; complete result-level details retained in this table
Dzienny 2026	ROBINS-I 2016 2016 original cohort-type version	Dzienny 2026: progression-free survival	D3 Bias in classification of interventions	Low	dose-stratified analyses ... <=10 mg vs >10 mg	Dzienny et al. (2026) | PDF pp.2–3 | Clinical	Serious	Initial dose was clearly recorded and threshold groups	Figure 3; complete result-level details retained in this

Study	Tool	Outcome or result	Domain	Judgment	Support for judgment	Information source	Overall judgment	Additional rationale	Figure link
	(19 September 2016)	(reported unadjusted HR)				data/Statistical analysis		were defined before outcome assessment.	table
Dzienny 2026	ROBINS-I 2016 2016 original cohort-type version (19 September 2016)	Dzienny 2026: progression-free survival (reported unadjusted HR)	D4 Bias due to deviations from intended interventions	Low	starting lenvatinib dose	Dzienny et al. (2026) | PDF pp.2–3 | Methods	Serious	The prespecified threshold estimand is baseline dose assignment; later routine modifications are downstream outcomes.	Figure 3; complete result-level details retained in this table
Dzienny 2026	ROBINS-I 2016 2016 original cohort-type version (19 September 2016)	Dzienny 2026: progression-free survival (reported unadjusted HR)	D5 Bias due to missing data	Low	PFS ... radiographic progression or death	Dzienny et al. (2026) | PDF pp.2–4 | Outcome availability/missingness	Serious	All 59 participants contributed to survival follow-up.	Figure 3; complete result-level details retained in this table
Dzienny 2026	ROBINS-I 2016 2016 original cohort-type version (19 September 2016)	Dzienny 2026: progression-free survival (reported unadjusted HR)	D6 Bias in measurement of outcomes	Moderate	PFS ... radiographic progression or death	Dzienny et al. (2026) | PDF pp.2–4 | Outcome measurement	Serious	Imaging was scheduled and independently reviewed, but assessors were not reported as blinded to dose.	Figure 3; complete result-level details retained in this table
Dzienny 2026	ROBINS-I 2016 2016 original cohort-type version (19 September 2016)	Dzienny 2026: progression-free survival (reported unadjusted HR)	D7 Bias in selection of the reported result	Serious	exploratory non-inferiority framework	Dzienny et al. (2026) | PDF pp.3–4; Supplementary Figures S3-S4 | Statistical analysis/Results	Serious	Consensus rationale: Multiple dose cutoffs, post hoc noninferiority, matching, and subgroup analyses create a high risk of result selection.	Figure 3; complete result-level details retained in this table
Dzienny 2026	ROBINS-I 2016 2016 original cohort-type version (19 September 2016)	Dzienny 2026: progression-free survival (reported unadjusted HR)	Overall Overall risk of bias	Serious	at least one domain was judged Serious	Dzienny et al. (2026) | See linked D1-D7 anchors | ROBINS-I overall algorithm	Serious	ROBINS-I 2016 overall judgment equals the highest domain when no domain is Critical.	Figure 3; complete result-level details retained in this table
Dzienny 2026	ROBINS-I 2016 2016 original cohort-type version (19 September 2016)	Dzienny 2026: disease control (risk ratio)	D1 Bias due to confounding	Serious	dose ... based on clinical judgment ... ECOG ... anticipated ability to tolerate	Dzienny et al. (2026) | PDF pp.2,7; Supplementary Table S1 | Methods/limitations	Serious	Dose selection used strong prognostic factors and dose groups remained substantially imbalanced despite exploratory matching.	Figure 3; complete result-level details retained in this table
Dzienny 2026	ROBINS-I 2016 2016 original cohort-type version (19 September 2016)	Dzienny 2026: objective response (risk ratio)	D1 Bias due to confounding	Serious	dose ... based on clinical judgment ... ECOG ... anticipated ability to tolerate	Dzienny et al. (2026) | PDF pp.2,7; Supplementary Table S1 | Methods/limitations	Serious	Dose selection used strong prognostic factors and dose groups remained substantially imbalanced despite exploratory matching.	Figure 3; complete result-level details retained in this table
Dzienny 2026	ROBINS-I 2016 2016 original cohort-type version (19 September 2016)	Dzienny 2026: disease control (risk ratio)	D2 Bias in selection of participants into the study	Moderate	excluded if they had incomplete medical records	Dzienny et al. (2026) | PDF p.2 | Patient population	Serious	Incomplete-record exclusions were possible, but treatment and follow-up began at combination initiation for included patients.	Figure 3; complete result-level details retained in this table
Dzienny 2026	ROBINS-I 2016 2016 original cohort-type version (19 September 2016)	Dzienny 2026: objective response (risk ratio)	D2 Bias in selection of participants into the study	Moderate	excluded if they had incomplete medical records	Dzienny et al. (2026) | PDF p.2 | Patient population	Serious	Incomplete-record exclusions were possible, but treatment and follow-up began at combination initiation for included patients.	Figure 3; complete result-level details retained in this table
Dzienny 2026	ROBINS-I 2016 2016 original cohort-type version (19 September 2016)	Dzienny 2026: disease control (risk ratio)	D3 Bias in classification of interventions	Low	dose-stratified analyses ... <=10 mg vs >10 mg	Dzienny et al. (2026) | PDF pp.2–3 | Clinical data/Statistical analysis	Serious	Initial dose was clearly recorded and threshold groups were defined before outcome assessment.	Figure 3; complete result-level details retained in this table
Dzienny 2026	ROBINS-I 2016 2016 original cohort-type version (19 September 2016)	Dzienny 2026: objective response (risk ratio)	D3 Bias in classification of interventions	Low	dose-stratified analyses ... <=10 mg vs >10 mg	Dzienny et al. (2026) | PDF pp.2–3 | Clinical data/Statistical analysis	Serious	Initial dose was clearly recorded and threshold groups were defined before outcome assessment.	Figure 3; complete result-level details retained in this table
Dzienny 2026	ROBINS-I 2016 2016 original cohort-type version (19 September 2016)	Dzienny 2026: disease control (risk ratio)	D4 Bias due to deviations from intended interventions	Low	starting lenvatinib dose	Dzienny et al. (2026) | PDF pp.2–3 | Methods	Serious	The prespecified threshold estimand is baseline dose assignment; later routine modifications are downstream outcomes.	Figure 3; complete result-level details retained in this table

Study	Tool	Outcome or result	Domain	Judgment	Support for judgment	Information source	Overall judgment	Additional rationale	Figure link
Dzienny 2026	ROBINS-I 2016 2016 original cohort-type version (19 September 2016)	Dzienny 2026: objective response (risk ratio)	D4 Bias due to deviations from intended interventions	Low	starting lenvatinib dose	Dzienny et al. (2026) | PDF pp.2–3 | Methods	Serious	The prespecified threshold estimand is baseline dose assignment; later routine modifications are downstream outcomes.	Figure 3; complete result-level details retained in this table
Dzienny 2026	ROBINS-I 2016 2016 original cohort-type version (19 September 2016)	Dzienny 2026: disease control (risk ratio)	D5 Bias due to missing data	Low	images were independently reviewed by a gynecologic oncologist and a radiologist	Dzienny et al. (2026) | PDF pp.2–4; Supplementary Table S2 | Outcome availability/missingness	Serious	Early discontinuers were explicitly classified as nonresponders and all 59 contributed.	Figure 3; complete result-level details retained in this table
Dzienny 2026	ROBINS-I 2016 2016 original cohort-type version (19 September 2016)	Dzienny 2026: objective response (risk ratio)	D5 Bias due to missing data	Low	images were independently reviewed by a gynecologic oncologist and a radiologist	Dzienny et al. (2026) | PDF pp.2–4; Supplementary Table S2 | Outcome availability/missingness	Serious	Early discontinuers were explicitly classified as nonresponders and all 59 contributed.	Figure 3; complete result-level details retained in this table
Dzienny 2026	ROBINS-I 2016 2016 original cohort-type version (19 September 2016)	Dzienny 2026: disease control (risk ratio)	D6 Bias in measurement of outcomes	Moderate	images were independently reviewed by a gynecologic oncologist and a radiologist	Dzienny et al. (2026) | PDF pp.2–4; Supplementary Table S2 | Outcome measurement	Serious	Regular imaging and dual review support comparability, but blinding to starting dose was not reported.	Figure 3; complete result-level details retained in this table
Dzienny 2026	ROBINS-I 2016 2016 original cohort-type version (19 September 2016)	Dzienny 2026: objective response (risk ratio)	D6 Bias in measurement of outcomes	Moderate	images were independently reviewed by a gynecologic oncologist and a radiologist	Dzienny et al. (2026) | PDF pp.2–4; Supplementary Table S2 | Outcome measurement	Serious	Regular imaging and dual review support comparability, but blinding to starting dose was not reported.	Figure 3; complete result-level details retained in this table
Dzienny 2026	ROBINS-I 2016 2016 original cohort-type version (19 September 2016)	Dzienny 2026: disease control (risk ratio)	D7 Bias in selection of the reported result	Serious	exploratory non-inferiority framework	Dzienny et al. (2026) | PDF pp.3–4; Supplementary Figures S3-S4 | Statistical analysis/Results	Serious	Consensus rationale: Multiple dose cutoffs, post hoc noninferiority, matching, and subgroup analyses create a high risk of result selection.	Figure 3; complete result-level details retained in this table
Dzienny 2026	ROBINS-I 2016 2016 original cohort-type version (19 September 2016)	Dzienny 2026: objective response (risk ratio)	D7 Bias in selection of the reported result	Serious	exploratory non-inferiority framework	Dzienny et al. (2026) | PDF pp.3–4; Supplementary Figures S3-S4 | Statistical analysis/Results	Serious	Consensus rationale: Multiple dose cutoffs, post hoc noninferiority, matching, and subgroup analyses create a high risk of result selection.	Figure 3; complete result-level details retained in this table
Dzienny 2026	ROBINS-I 2016 2016 original cohort-type version (19 September 2016)	Dzienny 2026: disease control (risk ratio)	Overall Overall risk of bias	Serious	at least one domain was judged Serious	Dzienny et al. (2026) | See linked D1-D7 anchors | ROBINS-I overall algorithm	Serious	ROBINS-I 2016 overall judgment equals the highest domain when no domain is Critical.	Figure 3; complete result-level details retained in this table
Dzienny 2026	ROBINS-I 2016 2016 original cohort-type version (19 September 2016)	Dzienny 2026: objective response (risk ratio)	Overall Overall risk of bias	Serious	at least one domain was judged Serious	Dzienny et al. (2026) | See linked D1-D7 anchors | ROBINS-I overall algorithm	Serious	ROBINS-I 2016 overall judgment equals the highest domain when no domain is Critical.	Figure 3; complete result-level details retained in this table
Nagase 2025	ROBINS-I 2016 2016 original cohort-type version (19 September 2016)	Nagase 2025: overall survival (reported unadjusted HR)	D1 Bias due to confounding	Serious	decisions regarding dose reduction or interruption	Nagase et al. (2025) | PDF pp.2,5,8 | Study definitions/RDI results/limitations	Serious	Achieved 8-week RDI was determined by early toxicity, response and clinical decisions; no time-varying-confounding method was used.	Figure 3; complete result-level details retained in this table
Nagase 2025	ROBINS-I 2016 2016 original cohort-type version (19 September 2016)	Nagase 2025: overall survival (reported unadjusted HR)	D2 Bias in selection of participants into the study	Serious	poor adherence ... irregular clinic visits ... insufficient medical records ... excluded	Nagase et al. (2025) | PDF p.2 | Eligibility criteria	Serious	Post-initiation monitoring and adherence determined inclusion, and exposure classification was completed after follow-up began.	Figure 3; complete result-level details retained in this table
Nagase 2025	ROBINS-I 2016 2016 original cohort-type version (19 September 2016)	Nagase 2025: overall survival (reported unadjusted HR)	D3 Bias in classification of interventions	Serious	dose administered in the first 8 weeks	Nagase et al. (2025) | PDF pp.2–3 | Study definitions	Serious	Consensus rationale: RDI classification used future post-initiation dosing and could be affected by early outcomes or prognostic events.	Figure 3; complete result-level details retained in this table

Study	Tool	Outcome or result	Domain	Judgment	Support for judgment	Information source	Overall judgment	Additional rationale	Figure link
Nagase 2025	ROBINS-I 2016 2016 original cohort-type version (19 September 2016)	Nagase 2025: overall survival (reported unadjusted HR)	D4 Bias due to deviations from intended interventions	Serious	84.8% ... required a dose reduction or interruption	Nagase et al. (2025) | PDF pp.2–5 | Study definitions/RDI results	Serious	The adherence/exposure strategy was not successfully maintained for many participants and no per-protocol causal method adjusted for deviations.	Figure 3; complete result-level details retained in this table
Nagase 2025	ROBINS-I 2016 2016 original cohort-type version (19 September 2016)	Nagase 2025: overall survival (reported unadjusted HR)	D5 Bias due to missing data	Low	OS ... death from any cause	Nagase et al. (2025) | PDF pp.2–6 | Outcome availability/missingness	Serious	Vital-status follow-up was reported for all 105 analyzed participants.	Figure 3; complete result-level details retained in this table
Nagase 2025	ROBINS-I 2016 2016 original cohort-type version (19 September 2016)	Nagase 2025: overall survival (reported unadjusted HR)	D6 Bias in measurement of outcomes	Low	OS ... death from any cause	Nagase et al. (2025) | PDF pp.2–6 | Outcome measurement	Serious	Death is objective; postbaseline exposure bias is assessed in D1-D4.	Figure 3; complete result-level details retained in this table
Nagase 2025	ROBINS-I 2016 2016 original cohort-type version (19 September 2016)	Nagase 2025: overall survival (reported unadjusted HR)	D7 Bias in selection of the reported result	Serious	cutoff ... determined ... 48% ... convenient cutoff value of 50%	Nagase et al. (2025) | PDF p.5; Supplementary Figure S5 | RDI results	Serious	The exposure threshold was derived from the same cohort using ORR and then applied to response and survival outcomes.	Figure 3; complete result-level details retained in this table
Nagase 2025	ROBINS-I 2016 2016 original cohort-type version (19 September 2016)	Nagase 2025: overall survival (reported unadjusted HR)	Overall Overall risk of bias	Serious	at least one domain was judged Serious	Nagase et al. (2025) | See linked D1-D7 anchors | ROBINS-I overall algorithm	Serious	ROBINS-I 2016 overall judgment equals the highest domain when no domain is Critical.	Figure 3; complete result-level details retained in this table
Nagase 2025	ROBINS-I 2016 2016 original cohort-type version (19 September 2016)	Nagase 2025: progression-free survival (adjusted HR)	D1 Bias due to confounding	Serious	decisions regarding dose reduction or interruption	Nagase et al. (2025) | PDF pp.2,5,8 | Study definitions/RDI results/limitations	Serious	Achieved 8-week RDI was determined by early toxicity, response and clinical decisions; no time-varying-confounding method was used.	Figure 3; complete result-level details retained in this table
Nagase 2025	ROBINS-I 2016 2016 original cohort-type version (19 September 2016)	Nagase 2025: progression-free survival (adjusted HR)	D2 Bias in selection of participants into the study	Serious	poor adherence ... irregular clinic visits ... insufficient medical records ... excluded	Nagase et al. (2025) | PDF p.2 | Eligibility criteria	Serious	Post-initiation monitoring and adherence determined inclusion, and exposure classification was completed after follow-up began.	Figure 3; complete result-level details retained in this table
Nagase 2025	ROBINS-I 2016 2016 original cohort-type version (19 September 2016)	Nagase 2025: progression-free survival (adjusted HR)	D3 Bias in classification of interventions	Serious	dose administered in the first 8 weeks	Nagase et al. (2025) | PDF pp.2–3 | Study definitions	Serious	Consensus rationale: RDI classification used future post-initiation dosing and could be affected by early outcomes or prognostic events.	Figure 3; complete result-level details retained in this table
Nagase 2025	ROBINS-I 2016 2016 original cohort-type version (19 September 2016)	Nagase 2025: progression-free survival (adjusted HR)	D4 Bias due to deviations from intended interventions	Serious	84.8% ... required a dose reduction or interruption	Nagase et al. (2025) | PDF pp.2–5 | Study definitions/RDI results	Serious	The adherence/exposure strategy was not successfully maintained for many participants and no per-protocol causal method adjusted for deviations.	Figure 3; complete result-level details retained in this table
Nagase 2025	ROBINS-I 2016 2016 original cohort-type version (19 September 2016)	Nagase 2025: progression-free survival (adjusted HR)	D5 Bias due to missing data	Low	multivariate Cox ... 8w-RDI	Nagase et al. (2025) | PDF pp.2–6,8 | Outcome availability/missingness	Serious	Survival outcomes were reported for all 105 analyzed participants.	Figure 3; complete result-level details retained in this table
Nagase 2025	ROBINS-I 2016 2016 original cohort-type version (19 September 2016)	Nagase 2025: progression-free survival (adjusted HR)	D6 Bias in measurement of outcomes	Serious	multivariate Cox ... 8w-RDI	Nagase et al. (2025) | PDF pp.2–6,8 | Outcome measurement	Serious	Adjustment does not correct nonstandardized progression measurement or assessor awareness.	Figure 3; complete result-level details retained in this table
Nagase 2025	ROBINS-I 2016 2016 original cohort-type version (19 September 2016)	Nagase 2025: progression-free survival (adjusted HR)	D7 Bias in selection of the reported result	Serious	cutoff ... determined ... 48% ... convenient cutoff value of 50%	Nagase et al. (2025) | PDF p.5; Supplementary Figure S5 | RDI results	Serious	The exposure threshold was derived from the same cohort using ORR and then applied to response and survival outcomes.	Figure 3; complete result-level details retained in this table

Study	Tool	Outcome or result	Domain	Judgment	Support for judgment	Information source	Overall judgment	Additional rationale	Figure link
Nagase 2025	ROBINS-I 2016 2016 original cohort-type version (19 September 2016)	Nagase 2025: progression-free survival (adjusted HR)	Overall Overall risk of bias	Serious	at least one domain was judged Serious	Nagase et al. (2025) | See linked D1-D7 anchors | ROBINS-I overall algorithm	Serious	ROBINS-I 2016 overall judgment equals the highest domain when no domain is Critical.	Figure 3; complete result-level details retained in this table
Nagase 2025	ROBINS-I 2016 2016 original cohort-type version (19 September 2016)	Nagase 2025: progression-free survival (reported unadjusted HR)	D1 Bias due to confounding	Serious	decisions regarding dose reduction or interruption	Nagase et al. (2025) | PDF pp.2,5,8 | Study definitions/RDI results/limitations	Serious	Achieved 8-week RDI was determined by early toxicity, response and clinical decisions; no time-varying-confounding method was used.	Figure 3; complete result-level details retained in this table
Nagase 2025	ROBINS-I 2016 2016 original cohort-type version (19 September 2016)	Nagase 2025: progression-free survival (reported unadjusted HR)	D2 Bias in selection of participants into the study	Serious	poor adherence ... irregular clinic visits ... insufficient medical records ... excluded	Nagase et al. (2025) | PDF p.2 | Eligibility criteria	Serious	Post-initiation monitoring and adherence determined inclusion, and exposure classification was completed after follow-up began.	Figure 3; complete result-level details retained in this table
Nagase 2025	ROBINS-I 2016 original cohort-type version (19 September 2016)	Nagase 2025: progression-free survival (reported unadjusted HR)	D3 Bias in classification of interventions	Serious	dose administered in the first 8 weeks	Nagase et al. (2025) | PDF pp.2–3 | Study definitions	Serious	Consensus rationale: RDI classification used future post-initiation dosing and could be affected by early outcomes or prognostic events.	Figure 3; complete result-level details retained in this table
Nagase 2025	ROBINS-I 2016 2016 original cohort-type version (19 September 2016)	Nagase 2025: progression-free survival (reported unadjusted HR)	D4 Bias due to deviations from intended interventions	Serious	84.8% ... required a dose reduction or interruption	Nagase et al. (2025) | PDF pp.2–5 | Study definitions/RDI results	Serious	The adherence/exposure strategy was not successfully maintained for many participants and no per-protocol causal method adjusted for deviations.	Figure 3; complete result-level details retained in this table
Nagase 2025	ROBINS-I 2016 2016 original cohort-type version (19 September 2016)	Nagase 2025: progression-free survival (reported unadjusted HR)	D5 Bias due to missing data	Low	PFS ... initiation ... progression or death	Nagase et al. (2025) | PDF pp.2–6,8 | Outcome availability/missingness	Serious	Survival outcomes were reported for all 105 analyzed participants.	Figure 3; complete result-level details retained in this table
Nagase 2025	ROBINS-I 2016 2016 original cohort-type version (19 September 2016)	Nagase 2025: progression-free survival (reported unadjusted HR)	D6 Bias in measurement of outcomes	Serious	PFS ... initiation ... progression or death	Nagase et al. (2025) | PDF pp.2–6,8 | Outcome measurement	Serious	Imaging timing and RECIST/iRECIST choice were not standardized across 15 centers.	Figure 3; complete result-level details retained in this table
Nagase 2025	ROBINS-I 2016 2016 original cohort-type version (19 September 2016)	Nagase 2025: progression-free survival (reported unadjusted HR)	D7 Bias in selection of the reported result	Serious	cutoff ... determined ... 48% ... convenient cutoff value of 50%	Nagase et al. (2025) | PDF p.5; Supplementary Figure S5 | RDI results	Serious	The exposure threshold was derived from the same cohort using ORR and then applied to response and survival outcomes.	Figure 3; complete result-level details retained in this table
Nagase 2025	ROBINS-I 2016 2016 original cohort-type version (19 September 2016)	Nagase 2025: progression-free survival (reported unadjusted HR)	Overall Overall risk of bias	Serious	at least one domain was judged Serious	Nagase et al. (2025) | See linked D1-D7 anchors | ROBINS-I overall algorithm	Serious	ROBINS-I 2016 overall judgment equals the highest domain when no domain is Critical.	Figure 3; complete result-level details retained in this table
Nagase 2025	ROBINS-I 2016 2016 original cohort-type version (19 September 2016)	Nagase 2025: disease control (risk ratio)	D1 Bias due to confounding	Serious	decisions regarding dose reduction or interruption	Nagase et al. (2025) | PDF pp.2,5,8 | Study definitions/RDI results/limitations	Serious	Achieved 8-week RDI was determined by early toxicity, response and clinical decisions; no time-varying-confounding method was used.	Figure 3; complete result-level details retained in this table
Nagase 2025	ROBINS-I 2016 2016 original cohort-type version (19 September 2016)	Nagase 2025: objective response (risk ratio)	D1 Bias due to confounding	Serious	decisions regarding dose reduction or interruption	Nagase et al. (2025) | PDF pp.2,5,8 | Study definitions/RDI results/limitations	Serious	Achieved 8-week RDI was determined by early toxicity, response and clinical decisions; no time-varying-confounding method was used.	Figure 3; complete result-level details retained in this table
Nagase 2025	ROBINS-I 2016 2016 original cohort-type version	Nagase 2025: disease control (risk ratio)	D2 Bias in selection of participants into the study	Serious	poor adherence ... irregular clinic visits ... insufficient	Nagase et al. (2025) | PDF p.2 | Eligibility criteria	Serious	Post-initiation monitoring and adherence determined	Figure 3; complete result-level details retained in this

Study	Tool	Outcome or result	Domain	Judgment	Support for judgment	Information source	Overall judgment	Additional rationale	Figure link
	(19 September 2016)				medical records ... excluded			inclusion, and exposure classification was completed after follow-up began.	table
Nagase 2025	ROBINS-I 2016 2016 original cohort-type version (19 September 2016)	Nagase 2025: objective response (risk ratio)	D2 Bias in selection of participants into the study	Serious	poor adherence ... irregular clinic visits ... insufficient medical records ... excluded	Nagase et al. (2025) | PDF p.2 | Eligibility criteria	Serious	Post-initiation monitoring and adherence determined inclusion, and exposure classification was completed after follow-up began.	Figure 3; complete result-level details retained in this table
Nagase 2025	ROBINS-I 2016 2016 original cohort-type version (19 September 2016)	Nagase 2025: disease control (risk ratio)	D3 Bias in classification of interventions	Serious	dose administered in the first 8 weeks	Nagase et al. (2025) | PDF pp.2–3 | Study definitions	Serious	Consensus rationale: RDI classification used future post-initiation dosing and could be affected by early outcomes or prognostic events.	Figure 3; complete result-level details retained in this table
Nagase 2025	ROBINS-I 2016 2016 original cohort-type version (19 September 2016)	Nagase 2025: objective response (risk ratio)	D3 Bias in classification of interventions	Serious	dose administered in the first 8 weeks	Nagase et al. (2025) | PDF pp.2–3 | Study definitions	Serious	Consensus rationale: RDI classification used future post-initiation dosing and could be affected by early outcomes or prognostic events.	Figure 3; complete result-level details retained in this table
Nagase 2025	ROBINS-I 2016 2016 original cohort-type version (19 September 2016)	Nagase 2025: disease control (risk ratio)	D4 Bias due to deviations from intended interventions	Serious	84.8% ... required a dose reduction or interruption	Nagase et al. (2025) | PDF pp.2–5 | Study definitions/RDI results	Serious	The adherence/exposure strategy was not successfully maintained for many participants and no per-protocol causal method adjusted for deviations.	Figure 3; complete result-level details retained in this table
Nagase 2025	ROBINS-I 2016 2016 original cohort-type version (19 September 2016)	Nagase 2025: objective response (risk ratio)	D4 Bias due to deviations from intended interventions	Serious	84.8% ... required a dose reduction or interruption	Nagase et al. (2025) | PDF pp.2–5 | Study definitions/RDI results	Serious	The adherence/exposure strategy was not successfully maintained for many participants and no per-protocol causal method adjusted for deviations.	Figure 3; complete result-level details retained in this table
Nagase 2025	ROBINS-I 2016 2016 original cohort-type version (19 September 2016)	Nagase 2025: disease control (risk ratio)	D5 Bias due to missing data	Moderate	3 discontinued ... before their initial radiology assessment	Nagase et al. (2025) | PDF pp.2–5,8 | Outcome availability/missingness	Serious	Three outcomes were not assessed and monitoring-related exclusions occurred before analysis.	Figure 3; complete result-level details retained in this table
Nagase 2025	ROBINS-I 2016 2016 original cohort-type version (19 September 2016)	Nagase 2025: objective response (risk ratio)	D5 Bias due to missing data	Moderate	3 discontinued ... before their initial radiology assessment	Nagase et al. (2025) | PDF pp.2–5,8 | Outcome availability/missingness	Serious	Three outcomes were not assessed and monitoring-related exclusions occurred before analysis.	Figure 3; complete result-level details retained in this table
Nagase 2025	ROBINS-I 2016 2016 original cohort-type version (19 September 2016)	Nagase 2025: disease control (risk ratio)	D6 Bias in measurement of outcomes	Serious	3 discontinued ... before their initial radiology assessment	Nagase et al. (2025) | PDF pp.2–5,8 | Outcome measurement	Serious	Imaging was nonstandardized and RECIST or iRECIST was chosen at physician discretion.	Figure 3; complete result-level details retained in this table
Nagase 2025	ROBINS-I 2016 2016 original cohort-type version (19 September 2016)	Nagase 2025: objective response (risk ratio)	D6 Bias in measurement of outcomes	Serious	3 discontinued ... before their initial radiology assessment	Nagase et al. (2025) | PDF pp.2–5,8 | Outcome measurement	Serious	Imaging was nonstandardized and RECIST or iRECIST was chosen at physician discretion.	Figure 3; complete result-level details retained in this table
Nagase 2025	ROBINS-I 2016 2016 original cohort-type version (19 September 2016)	Nagase 2025: disease control (risk ratio)	D7 Bias in selection of the reported result	Serious	cutoff ... determined ... 48% ... convenient cutoff value of 50%	Nagase et al. (2025) | PDF p.5; Supplementary Figure S5 | RDI results	Serious	The exposure threshold was derived from the same cohort using ORR and then applied to response and survival outcomes.	Figure 3; complete result-level details retained in this table
Nagase 2025	ROBINS-I 2016 2016 original cohort-type version (19 September 2016)	Nagase 2025: objective response (risk ratio)	D7 Bias in selection of the reported result	Serious	cutoff ... determined ... 48% ... convenient cutoff value of 50%	Nagase et al. (2025) | PDF p.5; Supplementary Figure S5 | RDI results	Serious	The exposure threshold was derived from the same cohort using ORR and then applied to response and survival	Figure 3; complete result-level details retained in this table

Study	Tool	Outcome or result	Domain	Judgment	Support for judgment	Information source	Overall judgment	Additional rationale	Figure link
								outcomes.	
Nagase 2025	ROBINS-I 2016 2016 original cohort-type version (19 September 2016)	Nagase 2025: disease control (risk ratio)	Overall Overall risk of bias	Serious	at least one domain was judged Serious	Nagase et al. (2025) | See linked D1-D7 anchors | ROBINS-I overall algorithm	Serious	ROBINS-I 2016 overall judgment equals the highest domain when no domain is Critical.	Figure 3; complete result-level details retained in this table
Nagase 2025	ROBINS-I 2016 2016 original cohort-type version (19 September 2016)	Nagase 2025: objective response (risk ratio)	Overall Overall risk of bias	Serious	at least one domain was judged Serious	Nagase et al. (2025) | See linked D1-D7 anchors | ROBINS-I overall algorithm	Serious	ROBINS-I 2016 overall judgment equals the highest domain when no domain is Critical.	Figure 3; complete result-level details retained in this table
Tochigi 2024	ROBINS-I 2016 2016 original cohort-type version (19 September 2016)	RDI NON8W | Result-level assessment: Tochigi 2024: relative dose intensity (risk ratio)	D1 Bias due to confounding	Serious	all 15 patients required AE-related treatment interruptions	Tochigi et al. (2024) | PDF pp.2,5 | Methods/RDI results	Serious	RDI was strongly determined by postbaseline toxicity and treatment duration; no baseline or time-varying confounding adjustment was performed.	Figure 3; complete result-level details retained in this table
Tochigi 2024	ROBINS-I 2016 2016 original cohort-type version (19 September 2016)	RDI NON8W | Result-level assessment: Tochigi 2024: relative dose intensity (risk ratio)	D2 Bias in selection of participants into the study	Serious	initiated ... by the end of December 2022	Tochigi et al. (2024) | PDF pp.2–3 | Participants/Results	Serious	Consensus rationale: Follow-up began at treatment initiation while whole-course RDI could only be known later, creating time-origin and survivor-selection concerns.	Figure 3; complete result-level details retained in this table
Tochigi 2024	ROBINS-I 2016 2016 original cohort-type version (19 September 2016)	RDI NON8W | Result-level assessment: Tochigi 2024: relative dose intensity (risk ratio)	D3 Bias in classification of interventions	Serious	RDI ... delivered dose intensity ... number of days of treatment	Tochigi et al. (2024) | PDF p.2 | Methodological considerations	Serious	RDI classification depended on future treatment duration and was therefore susceptible to early outcome-related classification.	Figure 3; complete result-level details retained in this table
Tochigi 2024	ROBINS-I 2016 2016 original cohort-type version (19 September 2016)	RDI NON8W | Result-level assessment: Tochigi 2024: relative dose intensity (risk ratio)	D4 Bias due to deviations from intended interventions	Serious	13 patients required lenvatinib dose reduction	Tochigi et al. (2024) | PDF pp.2,5 | Methods/RDI results	Serious	The achieved exposure was altered by interruptions and reductions, with no method estimating a controlled adherence strategy.	Figure 3; complete result-level details retained in this table
Tochigi 2024	ROBINS-I 2016 2016 original cohort-type version (19 September 2016)	RDI NON8W | Result-level assessment: Tochigi 2024: relative dose intensity (risk ratio)	D5 Bias due to missing data	Low	Fifteen patients were enrolled	Tochigi et al. (2024) | PDF pp.2–5,9 | Outcome availability/missingness	Serious	All 15 participants had treatment-course and PFS data reported.	Figure 3; complete result-level details retained in this table
Tochigi 2024	ROBINS-I 2016 2016 original cohort-type version (19 September 2016)	RDI NON8W | Result-level assessment: Tochigi 2024: relative dose intensity (risk ratio)	D6 Bias in measurement of outcomes	Moderate	Fifteen patients were enrolled	Tochigi et al. (2024) | PDF pp.2–5,9 | Outcome measurement	Serious	PFS and iRECIST assessment were clinically determined; blinding to achieved RDI was not reported.	Figure 3; complete result-level details retained in this table
Tochigi 2024	ROBINS-I 2016 2016 original cohort-type version (19 September 2016)	RDI NON8W | Result-level assessment: Tochigi 2024: relative dose intensity (risk ratio)	D7 Bias in selection of the reported result	Serious	RDI of neither the first 4 weeks nor the entire treatment period	Tochigi et al. (2024) | PDF pp.2,5 | Statistical analysis/RDI results	Serious	Two RDI windows and several exploratory clinical comparisons were reported without a public prespecified analysis plan.	Figure 3; complete result-level details retained in this table
Tochigi 2024	ROBINS-I 2016 2016 original cohort-type version (19 September 2016)	RDI NON8W | Result-level assessment: Tochigi 2024: relative dose intensity (risk ratio)	Overall Overall risk of bias	Serious	at least one domain was judged Serious	Tochigi et al. (2024) | See linked D1-D7 anchors | ROBINS-I overall algorithm	Serious	ROBINS-I 2016 overall judgment equals the highest domain when no domain is Critical.	Figure 3; complete result-level details retained in this table
Takahashi 2026	ROBINS-I 2016 (study-level confounding-control summary; result-level judgments remain below)	First-8-week relative dose intensity, 56% cutoff (narrative); all eligible effects from this cohort	Confounding-control summary	Serious	Achieved first-8-week RDI was determined after treatment initiation by early toxicity, interruption, response and clinical deterioration; the model adjusted only for histology and platinum-free interval in addition to RDI.	Takahashi et al. (2026) | PDF pp.2,4,6–7 | Methods, Results, Table 3 and limitations	Serious	Early-toxicity, survivor-selection, immortal-time, reverse-causality and time-varying-confounding concerns were not addressed with a day-56 landmark, time-dependent exposure or marginal structural model.	Figure 3; result-level traffic-light data and signaling-domain records retained below

Study	Tool	Outcome or result	Domain	Judgment	Support for judgment	Information source	Overall judgment	Additional rationale	Figure link
Takahashi 2026	ROBINS-I 2016 2016 original cohort-type version (19 September 2016)	Takahashi 2026: objective response (risk ratio)	D1 Bias due to confounding	Serious	Achieved RDI is determined by early toxicity, interruptions, early response/progression and clinician management; only histology and PFI were included with RDI,	Takahashi et al. (2026) | PDF pp.2,4,6–7	Serious	Achieved RDI is determined by early toxicity, interruptions, early response/progression and clinician management; only histology and PFI were included with RDI, with no time-varying-confounding method.	Figure 3; complete result-level details retained in this table
Takahashi 2026	ROBINS-I 2016 2016 original cohort-type version (19 September 2016)	Takahashi 2026: objective response (risk ratio)	D2 Bias in selection of participants into the study	Serious	Eligibility required at least two treatment cycles and the 56-day exposure is known only after treatment begins, producing survivor/selection and immortal-time	Takahashi et al. (2026) | PDF p.2	Serious	Eligibility required at least two treatment cycles and the 56-day exposure is known only after treatment begins, producing survivor/selection and immortal-time concerns without a landmark design.	Figure 3; complete result-level details retained in this table
Takahashi 2026	ROBINS-I 2016 2016 original cohort-type version (19 September 2016)	Takahashi 2026: objective response (risk ratio)	D3 Bias in classification of interventions	Serious	The exposure category is assigned from future post-initiation dose delivery and can be altered by prognostic events; it is not a baseline intervention classific	Takahashi et al. (2026) | PDF pp.2,4	Serious	The exposure category is assigned from future post-initiation dose delivery and can be altered by prognostic events; it is not a baseline intervention classification.	Figure 3; complete result-level details retained in this table
Takahashi 2026	ROBINS-I 2016 2016 original cohort-type version (19 September 2016)	Takahashi 2026: objective response (risk ratio)	D4 Bias due to deviations from intended interventions	Serious	Dose interruption/reduction and weekend-off dosing were physician-directed parts of achieved RDI; no per-protocol or time-varying causal strategy adjusted for d	Takahashi et al. (2026) | PDF pp.2,4	Serious	Dose interruption/reduction and weekend-off dosing were physician-directed parts of achieved RDI; no per-protocol or time-varying causal strategy adjusted for deviations.	Figure 3; complete result-level details retained in this table
Takahashi 2026	ROBINS-I 2016 2016 original cohort-type version (19 September 2016)	Takahashi 2026: objective response (risk ratio)	D5 Bias due to missing data	Moderate	PFS model used all 26 participants, but response analysis included 18 measurable-lesion participants and group-specific reasons for non-evaluability were not fu	Takahashi et al. (2026) | PDF pp.3–4,6	Serious	PFS model used all 26 participants, but response analysis included 18 measurable-lesion participants and group-specific reasons for non-evaluability were not fully characterized.	Figure 3; complete result-level details retained in this table
Takahashi 2026	ROBINS-I 2016 2016 original cohort-type version (19 September 2016)	Takahashi 2026: objective response (risk ratio)	D6 Bias in measurement of outcomes	Serious	PFS/response were retrospectively assessed with no reported blinded independent central review or uniform assessment schedule; exposure status could be known to	Takahashi et al. (2026) | PDF pp.2,4	Serious	PFS/response were retrospectively assessed with no reported blinded independent central review or uniform assessment schedule; exposure status could be known to assessors.	Figure 3; complete result-level details retained in this table
Takahashi 2026	ROBINS-I 2016 2016 original cohort-type version (19 September 2016)	Takahashi 2026: objective response (risk ratio)	D7 Bias in selection of the reported result	Serious	The 56% threshold was selected from the same cohort using ORR (AUC 0.55); multiple thresholds or models could have been reported and no public prespecified analysi	Takahashi et al. (2026) | PDF pp.4,7–8	Serious	The 56% threshold was selected from the same cohort using ORR (AUC 0.55); multiple thresholds or models could have been reported and no public prespecified analysis plan was identified. The unadjusted HR direction also depends on unavailable Figure S3B.	Figure 3; complete result-level details retained in this table
Takahashi 2026	ROBINS-I 2016 2016 original cohort-type version (19 September 2016)	Takahashi 2026: objective response (risk ratio)	Overall Overall risk of bias	Serious	At least one ROBINS-I 2016 domain is Serious and no domain is judged Critical; overall judgment is Serious.	Takahashi et al. (2026) | linked D1-D7	Serious	At least one ROBINS-I 2016 domain is Serious and no domain is judged Critical; overall judgment is Serious.	Figure 3; complete result-level details retained in this table
Takahashi 2026	ROBINS-I 2016 2016 original cohort-type version	Takahashi 2026: progression-free survival	D1 Bias due to confounding	Serious	Achieved RDI is determined by early toxicity,	Takahashi et al. (2026) | PDF pp.2,4,6–7	Serious	Achieved RDI is determined by early toxicity,	Figure 3; complete result-level details retained in this

Study	Tool	Outcome or result	Domain	Judgment	Support for judgment	Information source	Overall judgment	Additional rationale	Figure link
	(19 September 2016)	(adjusted HR)			interruptions, early response/progression and clinician management; only histology and PFI were included with RDI,			interruptions, early response/progression and clinician management; only histology and PFI were included with RDI, with no time-varying-confounding method.	table
Takahashi 2026	ROBINS-I 2016 2016 original cohort-type version (19 September 2016)	Takahashi 2026: progression-free survival (adjusted HR)	D2 Bias in selection of participants into the study	Serious	Eligibility required at least two treatment cycles and the 56-day exposure is known only after treatment begins, producing survivor/selection and immortal-time	Takahashi et al. (2026) | PDF p.2	Serious	Eligibility required at least two treatment cycles and the 56-day exposure is known only after treatment begins, producing survivor/selection and immortal-time concerns without a landmark design.	Figure 3; complete result-level details retained in this table
Takahashi 2026	ROBINS-I 2016 2016 original cohort-type version (19 September 2016)	Takahashi 2026: progression-free survival (adjusted HR)	D3 Bias in classification of interventions	Serious	The exposure category is assigned from future post-initiation dose delivery and can be altered by prognostic events; it is not a baseline intervention classific	Takahashi et al. (2026) | PDF pp.2,4	Serious	The exposure category is assigned from future post-initiation dose delivery and can be altered by prognostic events; it is not a baseline intervention classification.	Figure 3; complete result-level details retained in this table
Takahashi 2026	ROBINS-I 2016 2016 original cohort-type version (19 September 2016)	Takahashi 2026: progression-free survival (adjusted HR)	D4 Bias due to deviations from intended interventions	Serious	Dose interruption/reduction and weekend-off dosing were physician-directed parts of achieved RDI; no per-protocol or time-varying causal strategy adjusted for d	Takahashi et al. (2026) | PDF pp.2,4	Serious	Dose interruption/reduction and weekend-off dosing were physician-directed parts of achieved RDI; no per-protocol or time-varying causal strategy adjusted for deviations.	Figure 3; complete result-level details retained in this table
Takahashi 2026	ROBINS-I 2016 2016 original cohort-type version (19 September 2016)	Takahashi 2026: progression-free survival (adjusted HR)	D5 Bias due to missing data	Low	PFS model used all 26 participants, but response analysis included 18 measurable-lesion participants and group-specific reasons for non-evaluability were not fu	Takahashi et al. (2026) | PDF pp.3–4,6	Serious	PFS model used all 26 participants, but response analysis included 18 measurable-lesion participants and group-specific reasons for non-evaluability were not fully characterized.	Figure 3; complete result-level details retained in this table
Takahashi 2026	ROBINS-I 2016 2016 original cohort-type version (19 September 2016)	Takahashi 2026: progression-free survival (adjusted HR)	D6 Bias in measurement of outcomes	Serious	PFS/response were retrospectively assessed with no reported blinded independent central review or uniform assessment schedule; exposure status could be known to	Takahashi et al. (2026) | PDF pp.2,4	Serious	PFS/response were retrospectively assessed with no reported blinded independent central review or uniform assessment schedule; exposure status could be known to assessors.	Figure 3; complete result-level details retained in this table
Takahashi 2026	ROBINS-I 2016 2016 original cohort-type version (19 September 2016)	Takahashi 2026: progression-free survival (adjusted HR)	D7 Bias in selection of the reported result	Serious	The 56% threshold was selected from the same cohort using ORR (AUC 0.55); multiple thresholds or models could have been reported and no public prespecified analysi	Takahashi et al. (2026) | PDF pp.4,7–8	Serious	The 56% threshold was selected from the same cohort using ORR (AUC 0.55); multiple thresholds or models could have been reported and no public prespecified analysis plan was identified. The unadjusted HR direction also depends on unavailable Figure S3B.	Figure 3; complete result-level details retained in this table
Takahashi 2026	ROBINS-I 2016 2016 original cohort-type version (19 September 2016)	Takahashi 2026: progression-free survival (adjusted HR)	Overall Overall risk of bias	Serious	At least one ROBINS-I 2016 domain is Serious and no domain is judged Critical; overall judgment is Serious.	Takahashi et al. (2026) | linked D1-D7	Serious	At least one ROBINS-I 2016 domain is Serious and no domain is judged Critical; overall judgment is Serious.	Figure 3; complete result-level details retained in this table
Takahashi 2026	ROBINS-I 2016 2016 original cohort-type version (19 September 2016)	Takahashi 2026: progression-free survival (reported unadjusted HR)	D1 Bias due to confounding	Serious	Achieved RDI is determined by early toxicity, interruptions, early response/progression and	Takahashi et al. (2026) | PDF pp.2,4,6–7	Serious	Achieved RDI is determined by early toxicity, interruptions, early response/progression and	Figure 3; complete result-level details retained in this table

Study	Tool	Outcome or result	Domain	Judgment	Support for judgment	Information source	Overall judgment	Additional rationale	Figure link
					clinician management; only histology and PFI were included with RDI,			clinician management; only histology and PFI were included with RDI, with no time-varying-confounding method.	
Takahashi 2026	ROBINS-I 2016 2016 original cohort-type version (19 September 2016)	Takahashi 2026: progression-free survival (reported unadjusted HR)	D2 Bias in selection of participants into the study	Serious	Eligibility required at least two treatment cycles and the 56-day exposure is known only after treatment begins, producing survivor/selection and immortal-time	Takahashi et al. (2026) | PDF p.2	Serious	Eligibility required at least two treatment cycles and the 56-day exposure is known only after treatment begins, producing survivor/selection and immortal-time concerns without a landmark design.	Figure 3; complete result-level details retained in this table
Takahashi 2026	ROBINS-I 2016 2016 original cohort-type version (19 September 2016)	Takahashi 2026: progression-free survival (reported unadjusted HR)	D3 Bias in classification of interventions	Serious	The exposure category is assigned from future post-initiation dose delivery and can be altered by prognostic events; it is not a baseline intervention classific	Takahashi et al. (2026) | PDF pp.2,4	Serious	The exposure category is assigned from future post-initiation dose delivery and can be altered by prognostic events; it is not a baseline intervention classification.	Figure 3; complete result-level details retained in this table
Takahashi 2026	ROBINS-I 2016 2016 original cohort-type version (19 September 2016)	Takahashi 2026: progression-free survival (reported unadjusted HR)	D4 Bias due to deviations from intended interventions	Serious	Dose interruption/reduction and weekend-off dosing were physician-directed parts of achieved RDI; no per-protocol or time-varying causal strategy adjusted for d	Takahashi et al. (2026) | PDF pp.2,4	Serious	Dose interruption/reduction and weekend-off dosing were physician-directed parts of achieved RDI; no per-protocol or time-varying causal strategy adjusted for deviations.	Figure 3; complete result-level details retained in this table
Takahashi 2026	ROBINS-I 2016 2016 original cohort-type version (19 September 2016)	Takahashi 2026: progression-free survival (reported unadjusted HR)	D5 Bias due to missing data	Low	PFS model used all 26 participants, but response analysis included 18 measurable-lesion participants and group-specific reasons for non-evaluability were not fu	Takahashi et al. (2026) | PDF pp.3–4,6	Serious	PFS model used all 26 participants, but response analysis included 18 measurable-lesion participants and group-specific reasons for non-evaluability were not fully characterized.	Figure 3; complete result-level details retained in this table
Takahashi 2026	ROBINS-I 2016 2016 original cohort-type version (19 September 2016)	Takahashi 2026: progression-free survival (reported unadjusted HR)	D6 Bias in measurement of outcomes	Serious	PFS/response were retrospectively assessed with no reported blinded independent central review or uniform assessment schedule; exposure status could be known to	Takahashi et al. (2026) | PDF pp.2,4	Serious	PFS/response were retrospectively assessed with no reported blinded independent central review or uniform assessment schedule; exposure status could be known to assessors.	Figure 3; complete result-level details retained in this table
Takahashi 2026	ROBINS-I 2016 2016 original cohort-type version (19 September 2016)	Takahashi 2026: progression-free survival (reported unadjusted HR)	D7 Bias in selection of the reported result	Serious	The 56% threshold was selected from the same cohort using ORR (AUC 0.55); multiple thresholds or models could have been reported and no public prespecified analysi	Takahashi et al. (2026) | PDF pp.4,7–8	Serious	The 56% threshold was selected from the same cohort using ORR (AUC 0.55); multiple thresholds or models could have been reported and no public prespecified analysis plan was identified. The unadjusted HR direction also depends on unavailable Figure S3B.	Figure 3; complete result-level details retained in this table
Takahashi 2026	ROBINS-I 2016 2016 original cohort-type version (19 September 2016)	Takahashi 2026: progression-free survival (reported unadjusted HR)	Overall Overall risk of bias	Serious	At least one ROBINS-I 2016 domain is Serious and no domain is judged Critical; overall judgment is Serious.	Takahashi et al. (2026) | linked D1-D7	Serious	At least one ROBINS-I 2016 domain is Serious and no domain is judged Critical; overall judgment is Serious.	Figure 3; complete result-level details retained in this table

Note: The first rows summarize reported adjustment and uncontrolled critical confounders by cohort; subsequent rows report every result-level ROBINS-I domain judgment.

Supplementary Table C4. Extracted effects, provenance, adjustment, and causal-interpretation boundaries

Source-availability note: No extractable adjusted comparative RR or OR for an eligible binary contrast was identified in the available full reports and retrieved supplementary materials. Adjusted survival HRs remain separate. This statement is limited to the retrieved corpus; Takahashi Supplementary Figure S3B was unavailable.

Study	Effect descriptor	Synthesis	Exposure contrast	Outcome	Time point	Group or contrast	Reported metric	Raw values	Extracted effect	SE or CI	Adjustment status	Model or covariates	Quantitative synthesis status	Analysis role	Dependency or shared control	Confounding ROBINS-I	Causal interpretation boundary	Source location	Extraction confidence
How 2021	How 2021: Objective response rate; RR; Unadjusted; raw counts	Objective response, primary starting-dose analysis	Starting dose <20 mg versus 20 mg	Objective response rate	best response during treatment	<20 mg starting dose versus 20 mg starting dose	RR	18/47 vs 4/14	1.3404255319148937	SE 0.4613571158899614	Unadjusted; raw counts	none	Included in at least one prespecified quantitative synthesis	Main analysis	Same-cohort dependency; one effect was contributed per cohort.	Serious; predicted direction: Unpredictable	Crude or reported unadjusted observational association; meta-analysis cannot remove within-study confounding	How 2021, p. 19, Results, Table 3A	high
Zammarrelli 2023	Zammarrelli 2023: Objective response rate; RR; Unadjusted; raw counts	Objective response, primary starting-dose analysis; Objective response using total-arm denominators (sensitivity)	Starting dose <20 mg versus 20 mg	Objective response rate	best response during treatment	10 or 14 mg starting dose versus 20 mg starting dose	RR	6/20 vs 7/23	0.9857142857142857	SE 0.4648070015116428	Unadjusted; raw counts	none	Included in at least one prespecified quantitative synthesis	Main analysis; Sensitivity analysis	Same-cohort dependency; one effect was contributed per cohort.	Serious; predicted direction: Unpredictable	Crude or reported unadjusted observational association; meta-analysis cannot remove within-study confounding	Zammarrelli 2023, p. 18, Results, Table 3	high
Ta 2025	Ta 2025: Objective response rate; RR; Unadjusted; raw counts	Objective response, primary starting-dose analysis; Objective response using total-arm denominators (sensitivity)	Starting dose <20 mg versus 20 mg	Objective response rate	best response during treatment	<20 mg once daily versus 20 mg once daily	RR	52/154 vs 80/204	0.861038961038961	SE 0.14260190024312094	Unadjusted; raw counts	none	Included in at least one prespecified quantitative synthesis	Main analysis; Sensitivity analysis	Same-cohort dependency; one effect was contributed per cohort.	Serious; predicted direction: Unpredictable	Crude or reported unadjusted observational association; meta-analysis cannot remove within-study confounding	Ta 2025, p. 4, Results, Table 2	high
Corr 2025	Corr 2025: Objective response rate; RR; Unadjusted; raw counts	Objective response, primary starting-dose analysis; Objective response using total-arm denominators (sensitivity)	Starting dose <20 mg versus 20 mg	Objective response rate	best response during treatment	10/14/18 mg starting dose versus 20 mg starting dose	RR	59/151 vs 10/37	1.4456953642384105	SE 0.2886167163541545	Unadjusted; raw counts	none	Included in at least one prespecified quantitative synthesis	Main analysis; Sensitivity analysis	Same-cohort dependency; the shared 20-mg control was aggregated once, and one effect was contributed per cohort.	Serious; predicted direction: Unpredictable	Crude or reported unadjusted observational association; meta-analysis cannot remove within-study confounding	Corr 2025, p. 4, Results, Table 2	high
Boku 2026	Boku 2026: Objective response rate; RR; Unadjusted; raw counts	Objective response, primary starting-dose analysis; Objective response using total-arm	Starting dose <20 mg versus 20 mg	Objective response rate	best response during treatment	<20 mg starting dose versus 20 mg starting dose	RR	7/17 vs 9/16	0.7320261437908496	SE 0.36420423467676655	Unadjusted; raw counts	none	Included in at least one prespecified quantitative synthesis	Main analysis; Sensitivity analysis	Same-cohort dependency; one effect was contributed per cohort.	Serious; predicted direction: Unpredictable	Crude or reported unadjusted observational association; meta-analysis cannot	Boku 2026, p. Supplement DOCX, Results, Supplementary Table 1	high

Study	Effect descriptor	Synthesis	Exposure contrast	Outcome	Time point	Group or contrast	Reported metric	Raw values	Extracted effect	SE or CI	Adjustment status	Model or covariates	Quantitative synthesis status	Analysis role	Dependency or shared control	Confounding ROBINS-I	Causal interpretation boundary	Source location	Extraction confidence
		denominators (sensitivity)															remove within-study confounding		
How 2021	How 2021: Disease control rate; RR; Unadjusted; raw counts	Disease control, supplementary starting-dose analysis	Starting dose <20 mg versus 20 mg	Disease control rate	best response during treatment	<20 mg starting dose versus 20 mg starting dose	RR	34/47 vs 8/14	1.2659574468085109	SE 0.24840812694561762	Unadjusted; raw counts	none	Included in at least one prespecified quantitative synthesis	Supplementary exploratory	Same-cohort dependency; one effect was contributed per cohort.	Serious; predicted direction: Unpredictable	Crude or reported unadjusted observational association; meta-analysis cannot remove within-study confounding	How 2021, p. 19, Results, Table 3A	high
Zammarrelli 2023	Zammarrelli 2023: Disease control rate; RR; Unadjusted; raw counts	Disease control, supplementary starting-dose analysis	Starting dose <20 mg versus 20 mg	Disease control rate	best response during treatment	10 or 14 mg starting dose versus 20 mg starting dose	RR	14/20 vs 16/23	1.0062499999999999	SE 0.20112262567649172	Unadjusted; raw counts	none	Included in at least one prespecified quantitative synthesis	Supplementary exploratory	Same-cohort dependency; one effect was contributed per cohort.	Serious; predicted direction: Unpredictable	Crude or reported unadjusted observational association; meta-analysis cannot remove within-study confounding	Zammarrelli 2023, p. 18, Results, Table 3	high
Ta 2025	Ta 2025: Disease control rate; RR; Unadjusted; raw counts	Disease control, supplementary starting-dose analysis	Starting dose <20 mg versus 20 mg	Disease control rate	best response during treatment	<20 mg once daily versus 20 mg once daily	RR	76/154 vs 111/204	0.9069849069849071	SE 0.1037855311111857	Unadjusted; raw counts	none	Included in at least one prespecified quantitative synthesis	Supplementary exploratory	Same-cohort dependency; one effect was contributed per cohort.	Serious; predicted direction: Unpredictable	Crude or reported unadjusted observational association; meta-analysis cannot remove within-study confounding	Ta 2025, p. 4, Results, Table 2	high
Corr 2025	Corr 2025: Disease control rate; RR; Unadjusted; raw counts	Disease control, supplementary starting-dose analysis	Starting dose <20 mg versus 20 mg	Disease control rate	best response during treatment	10/14/18 mg starting dose versus 20 mg starting dose	RR	89/151 vs 20/37	1.0903973509933775	SE 0.16609157556258342	Unadjusted; raw counts	none	Included in at least one prespecified quantitative synthesis	Supplementary exploratory	Same-cohort dependency; the shared 20-mg control was aggregated once, and one effect was contributed per cohort.	Serious; predicted direction: Unpredictable	Crude or reported unadjusted observational association; meta-analysis cannot remove within-study confounding	Corr 2025, p. 4, Results, Table 2	high
Boku 2026	Boku 2026: Disease control rate; RR; Unadjusted; raw counts	Disease control, supplementary starting-dose analysis	Starting dose <20 mg versus 20 mg	Disease control rate	best response during treatment	<20 mg starting dose versus 20 mg starting dose	RR	14/17 vs 15/16	0.8784313725490196	SE 0.12950563186005998	Unadjusted; raw counts	none	Included in at least one prespecified quantitative synthesis	Supplementary exploratory	Same-cohort dependency; one effect was contributed per cohort.	Serious; predicted direction: Unpredictable	Crude or reported unadjusted observational association; meta-analysis cannot remove within-study confounding	Boku 2026, p. Supplement DOCX, Results, Supplementary Table 1	high
Zammarre	Zammarrelli	Grade 3 or	Starting	Grade 3 or	during	10 or 14	RR	18/20	1.15	SE	Unadjuste	none	Included in	Narrative	Same-	Serious;	Crude or	Zammarrelli	high

Study	Effect descriptor	Synthesis	Exposure contrast	Outcome	Time point	Group or contrast	Reported metric	Raw values	Extracted effect	SE or CI	Adjustment status	Model or covariates	Quantitative synthesis status	Analysis role	Dependency or shared control	Confounding ROBINS-I	Causal interpretation boundary	Source location	Extraction confidence
lli 2023	2023: Grade 3 or higher adverse event; RR; Unadjusted; raw counts	higher adverse events (exploratory)	dose <20 mg versus 20 mg	higher adverse event	treatment	mg starting dose versus 20 mg starting dose		vs 18/23		0.13278874290219742	d; raw counts		at least one prespecified quantitative synthesis	plus exploratory pooling	cohort dependency; one effect was contributed per cohort.	predicted direction: Unpredictable	reported unadjusted observational association; meta-analysis cannot remove within-study confounding	2023, p. 18, Results, Table 3	
Corr 2025	Corr 2025: Grade 3 or higher adverse event; RR; Unadjusted; raw counts	Grade 3 or higher adverse events (exploratory)	Starting dose <20 mg versus 20 mg	Grade 3 or higher adverse event	during treatment	10/14/18 mg starting dose versus 20 mg starting dose	RR	18/151 vs 5/37	0.8821192052980131	SE 0.471069009776951	Unadjusted; raw counts	none	Included in at least one prespecified quantitative synthesis	Narrative plus exploratory pooling	Same-cohort dependency; the shared 20-mg control was aggregated once, and one effect was contributed per cohort.	Serious; predicted direction: Unpredictable	Crude or reported unadjusted observational association; meta-analysis cannot remove within-study confounding	Corr 2025, p. 5, Results, Table 4	high
How 2021	How 2021: Subsequent lenvatinib dose reduction; RR; Unadjusted; raw counts	Subsequent lenvatinib dose reduction, primary starting-dose analysis	Starting dose <20 mg versus 20 mg	Subsequent lenvatinib dose reduction	during treatment	<20 mg starting dose versus 20 mg starting dose	RR	20/54 vs 10/16	0.5925925925925926	SE 0.2626432589682847	Unadjusted; raw counts	none	Included in at least one prespecified quantitative synthesis	Main analysis	Same-cohort dependency; one effect was contributed per cohort.	Serious; predicted direction: Unpredictable	Crude or reported unadjusted observational association; meta-analysis cannot remove within-study confounding	How 2021, p. 18, Results, Table 2	high
Zammarrelli 2023	Zammarrelli 2023: Subsequent lenvatinib dose reduction; RR; Unadjusted; raw counts	Subsequent lenvatinib dose reduction, primary starting-dose analysis	Starting dose <20 mg versus 20 mg	Subsequent lenvatinib dose reduction	during treatment	10 or 14 mg starting dose versus 20 mg starting dose	RR	11/20 vs 13/23	0.9730769230769233	SE 0.27267912821226825	Unadjusted; raw counts	none	Included in at least one prespecified quantitative synthesis	Main analysis	Same-cohort dependency; one effect was contributed per cohort.	Serious; predicted direction: Unpredictable	Crude or reported unadjusted observational association; meta-analysis cannot remove within-study confounding	Zammarrelli 2023, p. 15, Results, Table 1	high
Corr 2025	Corr 2025: Subsequent lenvatinib dose reduction; RR; Unadjusted; raw counts	Subsequent lenvatinib dose reduction, primary starting-dose analysis	Starting dose <20 mg versus 20 mg	Subsequent lenvatinib dose reduction	during treatment	10/14/18 mg starting dose versus 20 mg starting dose	RR	59/151 vs 25/37	0.5782781456953643	SE 0.1526420943221576	Unadjusted; raw counts	none	Included in at least one prespecified quantitative synthesis	Main analysis	Same-cohort dependency; the shared 20-mg control was aggregated once, and one effect was contributed per cohort.	Serious; predicted direction: Unpredictable	Crude or reported unadjusted observational association; meta-analysis cannot remove within-study confounding	Corr 2025, p. 4, Results, Table 2	high
How 2021	How 2021: Lenvatinib interruption or hold; RR;	Treatment interruption, supplementary starting-	Starting dose <20 mg versus	Lenvatinib interruption or hold	during treatment	<20 mg starting dose versus 20	RR	43/54 vs 15/16	0.8493827160493826	SE 0.094360808080667255	Unadjusted; raw counts	none	Included in at least one prespecified	Supplementary exploratory	Same-cohort dependency; one	Serious; predicted direction: Unpredicta	Crude or reported unadjusted observationa	How 2021, p. 18, Results, Table 2	high

Study	Effect descriptor	Synthesis	Exposure contrast	Outcome	Time point	Group or contrast	Reported metric	Raw values	Extracted effect	SE or CI	Adjustment status	Model or covariates	Quantitative synthesis status	Analysis role	Dependency or shared control	Confounding ROBINS-I	Causal interpretation boundary	Source location	Extraction confidence
	Unadjusted; raw counts	dose analysis	20 mg			mg starting dose							quantitative synthesis		effect was contributed per cohort.	ble	l association; meta-analysis cannot remove within-study confounding		
Zammarrelli 2023	Zammarrelli 2023: Lenvatinib interruption or hold; RR; Unadjusted; raw counts	Treatment interruption, supplementary starting-dose analysis	Starting dose <20 mg versus 20 mg	Lenvatinib interruption or hold	during treatment	10 or 14 mg starting dose versus 20 mg starting dose	RR	9/20 vs 8/23	1.2937500000000002	SE 0.377667645214077	Unadjusted; raw counts	none	Included in at least one prespecified quantitative synthesis	Supplementary exploratory	Same-cohort dependency; one effect was contributed per cohort.	Serious; predicted direction: Unpredictable	Crude or reported unadjusted observational association; meta-analysis cannot remove within-study confounding	Zammarrelli 2023, p. 15, Results, Table 1	high
How 2021	How 2021: Permanent discontinuation due to toxicity or intolerance; RR; Unadjusted; raw counts	Permanent discontinuation due to combination-treatment toxicity (exploratory)	Starting dose <20 mg versus 20 mg	Permanent discontinuation due to toxicity or intolerance	during treatment	<20 mg starting dose versus 20 mg starting dose	RR	19/54 vs 8/16	0.7037037037037037	SE 0.3108264152687958	Unadjusted; raw counts	none	Included in at least one prespecified quantitative synthesis	Narrative plus exploratory pooling	Same-cohort dependency; one effect was contributed per cohort.	Serious; predicted direction: Unpredictable	Crude or reported unadjusted observational association; meta-analysis cannot remove within-study confounding	Source report; exact location recorded during extraction toxicity-attributed outcome definition	high
Zammarrelli 2023	Zammarrelli 2023: Permanent discontinuation due to toxicity or intolerance; RR; Unadjusted; raw counts	Permanent lenvatinib discontinuation due to toxicity (narrative)	Starting dose <20 mg versus 20 mg	Permanent discontinuation due to toxicity or intolerance	during treatment	10 or 14 mg starting dose versus 20 mg starting dose	RR	0/20 vs 3/23	No executable comparative effect	No reported uncertainty; not executable for pooling	Unadjusted; raw counts	none	Not pooled in the prespecified quantitative syntheses; retained in narrative, SWiM, sensitivity context, or no-analysis route	Narrative only	Same-cohort dependency; one effect was contributed per cohort.	Serious; predicted direction: Unpredictable	Crude or reported unadjusted observational association; meta-analysis cannot remove within-study confounding	Source report; exact location recorded during extraction toxicity-attributed outcome definition	high
Corr 2025	Corr 2025: Permanent discontinuation due to toxicity or intolerance; RR; Unadjusted; raw counts	Permanent discontinuation due to combination-treatment toxicity (exploratory)	Starting dose <20 mg versus 20 mg	Permanent discontinuation due to toxicity or intolerance	during treatment	10/14/18 mg starting dose versus 20 mg starting dose	RR	32/151 vs 9/37	0.8712288447387785	SE 0.32971437264364545	Unadjusted; raw counts	none	Included in at least one prespecified quantitative synthesis	Narrative plus exploratory pooling	Same-cohort dependency; the shared 20-mg control was aggregated once, and one effect was contributed per cohort.	Serious; predicted direction: Unpredictable	Crude or reported unadjusted observational association; meta-analysis cannot remove within-study confounding	Source report; exact location recorded during extraction toxicity-attributed outcome definition	high
Corr 2025	Corr 2025: Permanent discontinuation, all cause; RR; Unadjusted; raw counts	Permanent discontinuation for any cause (narrative)	Starting dose <20 mg versus 20 mg	Permanent discontinuation, all cause	during treatment	10/14/18 mg starting dose versus 20 mg starting	RR	118/151 vs 33/37	0.8761790086293397	SE 0.07161049497733084	Unadjusted; raw counts	none	Not pooled in the prespecified quantitative syntheses;	Narrative only	Same-cohort dependency; the shared 20-mg control was	Serious; predicted direction: Unpredictable	Crude or reported unadjusted observational association; meta-	Source report; exact location recorded during extraction all-cause	high

Study	Effect descriptor	Synthesis	Exposure contrast	Outcome	Time point	Group or contrast	Reported metric	Raw values	Extracted effect	SE or CI	Adjustment status	Model or covariates	Quantitative synthesis status	Analysis role	Dependency or shared control	Confounding ROBINS-I	Causal interpretation boundary	Source location	Extraction confidence
						dose							retained in narrative, SWiM, sensitivity context, or no-analysis route		aggregated once, and one effect was contributed per cohort.		analysis cannot remove within-study confounding	outcome definition	
How 2021	How 2021: All-cause hospitalization; RR; Unadjusted; raw counts	Hospitalization for any cause (narrative)	Starting dose <20 mg versus 20 mg	All-cause hospitalization	reported follow-up window	<20 mg starting dose versus 20 mg starting dose	RR	30/54 vs 8/16	1.1111111111111112	SE 0.27805541680538215	Unadjusted; raw counts	none	Not pooled in the prespecified quantitative syntheses; retained in narrative, SWiM, sensitivity context, or no-analysis route	Narrative only	Same-cohort dependency; one effect was contributed per cohort.	Serious; predicted direction: Unpredictable	Crude or reported unadjusted observational association; meta-analysis cannot remove within-study confounding	Source report; exact location recorded during extraction all-cause outcome definition	high
Ta 2025	Ta 2025: All-cause hospitalization; RR; Unadjusted; raw counts	Hospitalization for any cause (narrative)	Starting dose <20 mg versus 20 mg	All-cause hospitalization	6 months	<20 mg once daily versus 20 mg once daily	RR	21/154 vs 39/204	0.7132867132867133	SE 0.24872596563739185	Unadjusted; raw counts	none	Not pooled in the prespecified quantitative syntheses; retained in narrative, SWiM, sensitivity context, or no-analysis route	Narrative only	Same-cohort dependency; one effect was contributed per cohort.	Serious; predicted direction: Unpredictable	Crude or reported unadjusted observational association; meta-analysis cannot remove within-study confounding	Source report; exact location recorded during extraction all-cause outcome definition	high
How 2021	How 2021: Toxicity-related hospitalization; RR; Unadjusted; raw counts	Hospitalization due to toxicity (narrative)	Starting dose <20 mg versus 20 mg	Toxicity-related hospitalization	during treatment	<20 mg starting dose versus 20 mg starting dose	RR	19/54 vs 4/16	1.4074074074074074	SE 0.47075796374448076	Unadjusted; raw counts	none	Not pooled in the prespecified quantitative syntheses; retained in narrative, SWiM, sensitivity context, or no-analysis route	Narrative only	Same-cohort dependency; one effect was contributed per cohort.	Serious; predicted direction: Unpredictable	Crude or reported unadjusted observational association; meta-analysis cannot remove within-study confounding	Source report; exact location recorded during extraction toxicity-attributed outcome definition	high
Ta 2025	Ta 2025: Six-month progression-free survival; RR; Unadjusted; raw counts	Six-month progression-free survival, <20 mg versus 20 mg (narrative)	Starting dose <20 mg versus 20 mg	Six-month progression-free survival	6 months	<20 mg once daily versus 20 mg once daily	RR	52/154 vs 82/204	0.8400380107697181	SE 0.14152888010638862	Unadjusted; raw counts	none	Not pooled in the prespecified quantitative syntheses; retained in narrative, SWiM, sensitivity context, or no-analysis route	Narrative only	Same-cohort dependency; one effect was contributed per cohort.	Serious; predicted direction: Unpredictable	Crude or reported unadjusted observational association; meta-analysis cannot remove within-study confounding	Ta 2025, p. 4, Results, Table 2	high
Dzienny 2026	Dzienny 2026: Objective response	Objective response, ≤10 mg versus >10	Starting dose ≤10 mg versus >	Objective response rate	best response during	<=10 mg starting dose versus >1	RR	14/28 vs 8/31	1.9375	SE 0.35840789778987386	Unadjusted; raw counts	none	Not pooled in the prespecified	Sensitivity analysis	Same-cohort dependency; one	Serious; predicted direction: Unpredicta	Crude or reported unadjusted observationa	Dzienny 2026, p. 4; Supplement slide 2,	high

Study	Effect descriptor	Synthesis	Exposure contrast	Outcome	Time point	Group or contrast	Reported metric	Raw values	Extracted effect	SE or CI	Adjustment status	Model or covariates	Quantitative synthesis status	Analysis role	Dependency or shared control	Confounding ROBINS-I	Causal interpretation boundary	Source location	Extraction confidence
	rate; RR; Unadjusted; raw counts	mg (sensitivity)	10 mg		treatment	0 mg starting dose							quantitative syntheses; retained in narrative, SWiM, sensitivity context, or no-analysis route		effect was contributed per cohort.	ble	l association; meta-analysis cannot remove within-study confounding	Results, Table 2; Table S2	
Dzienny 2026	Dzienny 2026: Disease control rate; RR; Unadjusted; raw counts	Disease control, ≤10 mg versus >10 mg (sensitivity)	Starting dose ≤10 mg versus > 10 mg	Disease control rate	best response during treatment	<=10 mg starting dose versus >10 mg starting dose	RR	14/28 vs 14/31	1.1071428571428572	SE 0.2736508589913946	Unadjusted; raw counts	none	Not pooled in the prespecified quantitative syntheses; retained in narrative, SWiM, sensitivity context, or no-analysis route	Sensitivity analysis	Same-cohort dependency; one effect was contributed per cohort.	Serious; predicted direction: Unpredictable	Crude or reported unadjusted observational association; meta-analysis cannot remove within-study confounding	Dzienny 2026, p. 4; Supplement slide 2, Results, Table 2; Table S2	high
Barbi 2025	Barbi 2025: Grade 2 or higher adverse event; RR; Unadjusted; raw counts	Grade 2 or higher adverse events, ≤10 mg versus >10 mg (context only)	Starting dose ≤10 mg versus > 10 mg	Grade 2 or higher adverse event	during treatment	<=10 mg starting dose versus >10 mg starting dose	RR	42/57 vs 26/35	0.9919028340080971	SE 0.1271053659390936	Unadjusted; raw counts	none	Not pooled in the prespecified quantitative syntheses; retained in narrative, SWiM, sensitivity context, or no-analysis route	no analysis	Same-cohort dependency; one effect was contributed per cohort.	Serious; predicted direction: Unpredictable	Crude or reported unadjusted observational association; meta-analysis cannot remove within-study confounding	Barbi 2025, p. 5, Results, Table 2	high
Barbi 2025	Barbi 2025: Subsequent lenvatinib dose reduction; RR; Unadjusted; raw counts	Subsequent lenvatinib dose reduction, ≤10 mg versus >10 mg (sensitivity)	Starting dose ≤10 mg versus > 10 mg	Subsequent lenvatinib dose reduction	during treatment	<=10 mg starting dose versus >10 mg starting dose	RR	26/57 vs 13/35	1.2280701754385963	SE 0.2631906669395099	Unadjusted; raw counts	none	Not pooled in the prespecified quantitative syntheses; retained in narrative, SWiM, sensitivity context, or no-analysis route	Sensitivity analysis	Same-cohort dependency; one effect was contributed per cohort.	Serious; predicted direction: Unpredictable	Crude or reported unadjusted observational association; meta-analysis cannot remove within-study confounding	Barbi 2025, p. 5, Results, Table 2	high
Barbi 2025	Barbi 2025: Lenvatinib interruption or hold; RR; Unadjusted; raw counts	Treatment interruption, ≤10 mg versus >10 mg (sensitivity)	Starting dose ≤10 mg versus > 10 mg	Lenvatinib interruption or hold	during treatment	<=10 mg starting dose versus >10 mg starting dose	RR	29/57 vs 17/35	1.0474716202270382	SE 0.21723489547469804	Unadjusted; raw counts	none	Not pooled in the prespecified quantitative syntheses; retained in narrative, SWiM, sensitivity context, or no-analysis route	Sensitivity analysis	Same-cohort dependency; one effect was contributed per cohort.	Serious; predicted direction: Unpredictable	Crude or reported unadjusted observational association; meta-analysis cannot remove within-study confounding	Barbi 2025, p. 5, Results, Table 2	high
Barbi	Barbi 2025:	Any-toxicity	Starting	Permanent	during	<=10 mg	RR	21/57	1.0745614035087	SE	Unadjuste	none	Not pooled	Sensitivity	Same-	Serious;	Crude or	Source	high

Study	Effect descriptor	Synthesis	Exposure contrast	Outcome	Time point	Group or contrast	Reported metric	Raw values	Extracted effect	SE or CI	Adjustment status	Model or covariates	Quantitative synthesis status	Analysis role	Dependency or shared control	Confounding ROBINS-I	Causal interpretation boundary	Source location	Extraction confidence
2025	Permanent discontinuation due to toxicity or intolerance; RR; Unadjusted; raw counts	discontinuation, ≤10 mg versus >10 mg (sensitivity)	dose ≤10 mg versus > 10 mg	discontinuation due to toxicity or intolerance	treatment	starting dose versus >10 mg starting dose		vs 12/35	718	0.2912680770902118	d; raw counts		in the prespecified quantitative syntheses; retained in narrative, SWiM, sensitivity context, or no-analysis route	analysis	cohort dependency; one effect was contributed per cohort.	predicted direction: Unpredictable	reported unadjusted observational association; meta-analysis cannot remove within-study confounding	report; exact location recorded during extraction toxicity-attributed outcome definition	
Dzienny 2026	Dzienny 2026: Permanent discontinuation due to toxicity or intolerance; RR; Unadjusted; raw counts	Permanent lenvatinib discontinuation, ≤10 mg versus >10 mg (sensitivity)	Starting dose ≤10 mg versus > 10 mg	Permanent discontinuation due to toxicity or intolerance	during treatment	<=10 mg starting dose versus >10 mg starting dose	RR	3/28 vs 8/31	0.4151785714285714	SE 0.624788750781349	Unadjusted; raw counts	none	Not pooled in the prespecified quantitative syntheses; retained in narrative, SWiM, sensitivity context, or no-analysis route	Sensitivity analysis	Same-cohort dependency; one effect was contributed per cohort.	Serious; predicted direction: Unpredictable	Crude or reported unadjusted observational association; meta-analysis cannot remove within-study confounding	Source report; exact location recorded during extraction toxicity-attributed outcome definition	high
Dzienny 2026	Dzienny 2026: Permanent discontinuation due to toxicity or intolerance; RR; Unadjusted; raw counts	Any-toxicity discontinuation, ≤10 mg versus >10 mg (sensitivity)	Starting dose ≤10 mg versus > 10 mg	Permanent discontinuation due to toxicity or intolerance	during treatment	<=10 mg starting dose versus >10 mg starting dose	RR	9/28 vs 10/31	0.9964285714285716	SE 0.37833683521525674	Unadjusted; raw counts	none	Not pooled in the prespecified quantitative syntheses; retained in narrative, SWiM, sensitivity context, or no-analysis route	Sensitivity analysis	Same-cohort dependency; one effect was contributed per cohort.	Serious; predicted direction: Unpredictable	Crude or reported unadjusted observational association; meta-analysis cannot remove within-study confounding	Dzienny 2026, p. Supplement slide 6, Results, Table S6	high
Nagase 2025	Nagase 2025: Objective response rate; RR; Unadjusted; raw counts	Objective response, first-8-week RDI	First-8-week relative dose intensity	Objective response rate	best response during treatment	first-8-week RDI >=50 % versus first-8-week RDI <50%	RR	29/72 vs 4/33	3.3229166666666665	SE 0.4901946954310812	Unadjusted; raw counts	none	Not pooled in the prespecified quantitative syntheses; retained in narrative, SWiM, sensitivity context, or no-analysis route	Supplementary exploratory	Same-cohort dependency; one effect was contributed per cohort.	Serious; predicted direction: Unpredictable	Postbaseline exposure-response association; not a baseline causal contrast because early toxicity, survivor selection, immortal time, and time-varying confounding may determine exposure	Nagase 2025, p. 5, Results, Results	high
Nagase 2025	Nagase 2025: Disease control rate; RR; Unadjusted; raw counts	Disease control, first-8-week RDI	First-8-week relative dose intensity	Disease control rate	best response during treatment	first-8-week RDI >=50 % versus first-8-week RDI	RR	61/72 vs 19/33	1.4714912280701753	SE 0.15758522258892185	Unadjusted; raw counts	none	Not pooled in the prespecified quantitative	Supplementary exploratory	Same-cohort dependency; one effect was contribute	Serious; predicted direction: Unpredictable	Postbaseline exposure-response association; not a baseline	Nagase 2025, p. 5, Results, Results	high

Study	Effect descriptor	Synthesis	Exposure contrast	Outcome	Time point	Group or contrast	Reported metric	Raw values	Extracted effect	SE or CI	Adjustment status	Model or covariates	Quantitative synthesis status	Analysis role	Dependency or shared control	Confounding ROBINS-I	Causal interpretation boundary	Source location	Extraction confidence
						<50%							syntheses; retained in narrative, SWiM, sensitivity context, or no-analysis route		d per cohort.		causal contrast because early toxicity, survivor selection, immortal time, and time-varying confounding may determine exposure		
Zammarrelli 2023	Zammarrelli 2023: Progression-free survival; HR; reported unadjusted	Progression-free survival, reported unadjusted HR (exploratory)	Starting dose <20 mg versus 20 mg	Progression-free survival	full follow-up	<20 mg versus 20 mg	HR	No arm-level raw values reported for this effect	0.448430493274	95% CI 0.223713646532 to 0.900900900901	reported unadjusted	none	Included in at least one prespecified quantitative synthesis	Narrative plus exploratory pooling	Same-cohort dependency; one effect was contributed per cohort.	Serious; predicted direction: Unpredictable	Crude or reported unadjusted observational association; meta-analysis cannot remove within-study confounding	Zammarrelli 2023, p. 19, Results, Table 4	high
Zammarrelli 2023	Zammarrelli 2023: Overall survival; HR; reported unadjusted	Overall survival, reported unadjusted HR, <20 mg versus 20 mg (narrative)	Starting dose <20 mg versus 20 mg	Overall survival	full follow-up	<20 mg versus 20 mg	HR	No arm-level raw values reported for this effect	0.625	95% CI 0.269541778976 to 1.44927536232	reported unadjusted	none	Not pooled in the prespecified quantitative syntheses; retained in narrative, SWiM, sensitivity context, or no-analysis route	Narrative only	Same-cohort dependency; one effect was contributed per cohort.	Serious; predicted direction: Unpredictable	Crude or reported unadjusted observational association; meta-analysis cannot remove within-study confounding	Zammarrelli 2023, p. 19, Results, Table 4	high
Ta 2025	Ta 2025: Progression-free survival; HR; reported unadjusted	Progression-free survival, reported unadjusted HR (exploratory)	Starting dose <20 mg versus 20 mg	Progression-free survival	full follow-up	<20 mg daily versus 20 mg daily	HR	No arm-level raw values reported for this effect	0.990099009901	95% CI 0.787401574803 to 1.23456790123	reported unadjusted	none	Included in at least one prespecified quantitative synthesis	Narrative plus exploratory pooling	Same-cohort dependency; one effect was contributed per cohort.	Serious; predicted direction: Unpredictable	Crude or reported unadjusted observational association; meta-analysis cannot remove within-study confounding	Ta 2025, p. 4, Results, Results/Kaplan-Meier comparison	high
Boku 2026	Boku 2026: Progression-free survival; HR; adjusted	Progression-free survival, adjusted HR, <20 mg versus 20 mg (narrative)	Starting dose <20 mg versus 20 mg	Progression-free survival	full follow-up	<20 mg versus 20 mg	HR	No arm-level raw values reported for this effect	5.83	95% CI 1.84 to 18.43	adjusted	age >=75 years; ECOG PS >=1	Not pooled in the prespecified quantitative syntheses; retained in narrative, SWiM, sensitivity context, or no-analysis route	Narrative only	Same-cohort dependency; one effect was contributed per cohort.	Serious; predicted direction: Unpredictable	Study-reported adjusted observational association; residual and unmeasured confounding remain, and the estimate is not equivalent to randomized	Boku 2026, pp. 6,14; published XML, Results, Table 3	high

Study	Effect descriptor	Synthesis	Exposure contrast	Outcome	Time point	Group or contrast	Reported metric	Raw values	Extracted effect	SE or CI	Adjustment status	Model or covariates	Quantitative synthesis status	Analysis role	Dependency or shared control	Confounding ROBINS-I	Causal interpretation boundary	Source location	Extraction confidence
																	assignment		
Boku 2026	Boku 2026: Overall survival; HR; adjusted	Overall survival, adjusted HR, <20 mg versus 20 mg (narrative)	Starting dose <20 mg versus 20 mg	Overall survival	full follow-up	<20 mg versus 20 mg	HR	No arm-level raw values reported for this effect	1.04	95% CI 0.18 to 5.93	adjusted	peritoneal metastasis; age >=75 years; starting dose	Not pooled in the prespecified quantitative syntheses; retained in narrative, SWiM, sensitivity context, or no-analysis route	Narrative only	Same-cohort dependency; one effect was contributed per cohort.	Serious; predicted direction: Unpredictable	Study-reported adjusted observational association; residual and unmeasured confounding remain, and the estimate is not equivalent to randomized assignment	Boku 2026, pp. 6,14; published XML, Results, Table 4/VOR XML	high
Barbi 2025	Barbi 2025: Progression-free survival; HR; adjusted	Progression-free survival, adjusted HR, ≤10 mg versus >10 mg (sensitivity)	Starting dose ≤10 mg versus > 10 mg	Progression-free survival	full follow-up	<=10 mg versus >10 mg	HR	No arm-level raw values reported for this effect	2.92	95% CI 1.32 to 6.44	adjusted	age at lenvatinib start	Not pooled in the prespecified quantitative syntheses; retained in narrative, SWiM, sensitivity context, or no-analysis route	Sensitivity analysis	Same-cohort dependency; one effect was contributed per cohort.	Serious; predicted direction: Unpredictable	Study-reported adjusted observational association; residual and unmeasured confounding remain, and the estimate is not equivalent to randomized assignment	Barbi 2025, p. 7, Results, Figure 3; Table 3	high
Dzienny 2026	Dzienny 2026: Progression-free survival; HR; reported unadjusted	Progression-free survival, reported unadjusted HR, ≤10 mg versus >10 mg (sensitivity)	Starting dose ≤10 mg versus > 10 mg	Progression-free survival	full follow-up	<=10 mg versus >10 mg	HR	No arm-level raw values reported for this effect	0.79	95% CI 0.435 to 1.44	reported unadjusted	none	Not pooled in the prespecified quantitative syntheses; retained in narrative, SWiM, sensitivity context, or no-analysis route	Sensitivity analysis	Same-cohort dependency; one effect was contributed per cohort.	Serious; predicted direction: Unpredictable	Crude or reported unadjusted observational association; meta-analysis cannot remove within-study confounding	Dzienny 2026, p. 4, Results, Figure 2a	high
Nagase 2025	Nagase 2025: Progression-free survival; HR; reported unadjusted	Progression-free survival, reported unadjusted HR, first-8-week RDI	First-8-week relative dose intensity	Progression-free survival	full follow-up	8w-RDI >=50% versus <50%	HR	No arm-level raw values reported for this effect	0.45	95% CI 0.28 to 0.73	reported unadjusted	none	Not pooled in the prespecified quantitative syntheses; retained in narrative, SWiM, sensitivity context, or no-analysis route	Supplementary exploratory	Same-cohort dependency; one effect was contributed per cohort.	Serious; predicted direction: Unpredictable	Postbaseline exposure-response association; not a baseline causal contrast because early toxicity, survivor selection, immortal time, and time-varying confounding may determine exposure	Nagase 2025, p. 5–6, Results, Figure 3A; Table 3	high

Study	Effect descriptor	Synthesis	Exposure contrast	Outcome	Time point	Group or contrast	Reported metric	Raw values	Extracted effect	SE or CI	Adjustment status	Model or covariates	Quantitative synthesis status	Analysis role	Dependency or shared control	Confounding ROBINS-I	Causal interpretation boundary	Source location	Extraction confidence
Nagase 2025	Nagase 2025: Progression-free survival; HR; adjusted	Progression-free survival, adjusted HR, first-8-week RDI	First-8-week relative dose intensity	Progression-free survival	full follow-up	8w-RDI >=50 % versus <50%	HR	No arm-level raw values reported for this effect	0.53	95% CI 0.31 to 0.91	adjusted	age; ECOG PS; PFI; histology; FIGO stage; MMR status; 8w-RDI	Not pooled in the prespecified quantitative syntheses; retained in narrative, SWiM, sensitivity context, or no-analysis route	Supplementary exploratory	Same-cohort dependency; one effect was contributed per cohort.	Serious; predicted direction: Unpredictable	Postbaseline exposure-response association; not a baseline causal contrast because early toxicity, survivor selection, immortal time, and time-varying confounding may determine exposure	Nagase 2025, p. 6, Results, Table 3	high
Nagase 2025	Nagase 2025: Overall survival; HR; reported unadjusted	Overall survival, reported unadjusted HR, first-8-week RDI	First-8-week relative dose intensity	Overall survival	full follow-up	8w-RDI >=50 % versus <50%	HR	No arm-level raw values reported for this effect	0.53	95% CI 0.3 to 0.92	reported unadjusted	none	Not pooled in the prespecified quantitative syntheses; retained in narrative, SWiM, sensitivity context, or no-analysis route	Supplementary exploratory	Same-cohort dependency; one effect was contributed per cohort.	Serious; predicted direction: Unpredictable	Postbaseline exposure-response association; not a baseline causal contrast because early toxicity, survivor selection, immortal time, and time-varying confounding may determine exposure	Nagase 2025, pp. 1,5–6, Results, Abstract; Figure 3B	high
How 2021	How 2021: Objective response rate; RR; Unadjusted; raw counts	Objective response using total-arm denominators (sensitivity)	Starting dose <20 mg versus 20 mg	Objective response rate	best response during treatment	<20 mg starting dose versus 20 mg starting dose	RR	18/54 vs 4/16	1.3333333333333333	SE 0.4738533919231106	Unadjusted; raw counts	none	Not pooled in the prespecified quantitative syntheses; retained in narrative, SWiM, sensitivity context, or no-analysis route	Sensitivity analysis	Same-cohort dependency; one effect was contributed per cohort.	No effect-specific D1 row; see study-level summary; predicted direction: Direction unpredictable	Crude or reported unadjusted observational association; meta-analysis cannot remove within-study confounding	How 2021, p. 18–19, Results, Table 2 and Table 3A	high
Takahashi 2026	Takahashi 2026: Objective response rate; RR; Unadjusted; raw counts	Objective response, first-8-week RDI 56% cutoff (narrative)	First-8-week relative dose intensity, 56% cutoff (narrative)	Objective response rate	best response during treatment	first-8-week RDI >=56 % versus first-8-week RDI <56%	RR	4/11 vs 1/7	2.54545454545	95% CI 0.352912260688 to 18.3596308905	Unadjusted; raw counts	none	Not pooled; source-specific narrative only	Supplementary exploratory; narrative only	Same-cohort dependency across objective-response and progression-free-survival results; no external shared	Serious; predicted direction unpredictable	Postbaseline association only; no causal, equivalence, noninferiority or maintained-efficacy claim	Takahashi 2026, p. PDF p.4 (journal 707), Results, Figure S3A cited	high

Study	Effect descriptor	Synthesis	Exposure contrast	Outcome	Time point	Group or contrast	Reported metric	Raw values	Extracted effect	SE or CI	Adjustment status	Model or covariates	Quantitative synthesis status	Analysis role	Dependency or shared control	Confounding ROBINS-I	Causal interpretation boundary	Source location	Extraction confidence
															control; cohort counted once.				
Takahashi 2026	Takahashi 2026: Progression-free survival; HR; adjusted	Progression-free survival, adjusted HR, first-8-week RDI 56% cutoff (narrative)	First-8-week relative dose intensity, 56% cutoff (narrative)	Progression-free survival	full follow-up	first-8-week RDI >=56 % versus first-8-week RDI <56%	HR	12/17 vs 4/9	1.49253731343	95% CI 0.408163265306 to 5.26315789474	adjusted	histology; platinum-free interval; first-8-week RDI	Not pooled; source-specific narrative only	Supplementary exploratory; narrative only	Same-cohort dependency across objective-response and progression-free-survival results; no external shared control; cohort counted once.	Serious; predicted direction unpredictable	Postbaseline association only; no causal, equivalence, noninferiority or maintained-efficacy claim	Takahashi 2026, p. PDF p.6 (journal 709), Results, Table 3	high
Takahashi 2026	Takahashi 2026: Progression-free survival; HR; reported unadjusted	No quantitative synthesis assigned; source direction unresolved; narrative only	First-8-week relative dose intensity, 56% cutoff (narrative)	Progression-free survival	full follow-up	unclear without Figure S3B versus unclear without Figure S3B	HR	No arm-level raw values reported for this effect	Reported original 0.96	95% CI 0.30 to 3.05	reported unadjusted	none	Not pooled; source-specific narrative only	Narrative only	Same-cohort dependency across objective-response and progression-free-survival results; no external shared control; cohort counted once.	Serious; predicted direction unpredictable	Postbaseline association only; no causal, equivalence, noninferiority or maintained-efficacy claim	Takahashi 2026, p. PDF p.4 (journal 707), Results, Figure S3B cited but unavailable	low for direction high for numbers

Note: Crude arm-count effects, reported unadjusted effects, and adjusted effects remain distinct provenance layers.

Supplementary Table C5. Effect-size processing and direction harmonization

Study	Outcome	Original metric	Processing step	Derivation rule	Converted metric	Direction harmonization	Reference group handling	Assumption	Sensitivity requirement	Source location	Verification status
How 2021	Objective response rate	RR	calculated harmonized from reported categories or arms; prespecified binary RR derivation	Log risk ratio and sampling variance derived from two-arm event counts	RR	<20 mg starting dose vs 20 mg starting dose -> <20 mg starting dose vs 20 mg starting dose	Original 20 mg starting dose; target How 2021 comparison group (20 mg)	no continuity correction; source groups mutually exclusive; any category/arm aggregation is documented in effect notes	yes	How 2021, p. 19, Results, Table 3A	Verified against the source; included in quantitative synthesis; no additional transformation.
Zammarrelli 2023	Objective response rate	RR	calculated harmonized from reported categories or arms; prespecified binary RR derivation	Log risk ratio and sampling variance derived from two-arm event counts	RR	10 or 14 mg starting dose vs 20 mg starting dose -> 10 or 14 mg starting dose vs 20 mg starting dose	Original 20 mg starting dose; target Zammarrelli 2023 comparison group (20 mg)	no continuity correction; source groups mutually exclusive; any category/arm aggregation is documented in effect notes	yes	Zammarrelli 2023, p. 18, Results, Table 3	Verified against the source; included in quantitative synthesis; no additional transformation.
Ta 2025	Objective response rate	RR	calculated harmonized from reported categories or arms; prespecified binary RR derivation	Log risk ratio and sampling variance derived from two-arm event counts	RR	<20 mg once daily vs 20 mg once daily -> <20 mg once daily vs 20 mg once daily	Original 20 mg once daily; target Ta 2025 comparison group (20 mg)	no continuity correction; source groups mutually exclusive; any category/arm aggregation is documented in effect notes	yes	Ta 2025, p. 4, Results, Table 2	Verified against the source; included in quantitative synthesis; no additional transformation.
Corr 2025	Objective response rate	RR	calculated harmonized from reported categories or arms; prespecified binary RR derivation	Log risk ratio and sampling variance derived from two-arm event counts	RR	10/14/18 mg starting dose vs 20 mg starting dose -> 10/14/18 mg starting dose vs 20 mg starting dose	Original 20 mg starting dose; target Corr 2025 comparison group (20 mg)	no continuity correction; source groups mutually exclusive; any category/arm aggregation is documented in effect notes	yes	Corr 2025, p. 4, Results, Table 2	Verified against the source; included in quantitative synthesis; no additional transformation.
Boku 2026	Objective response rate	RR	calculated from reported arm events; prespecified binary RR derivation	Log risk ratio and sampling variance derived from two-arm event counts	RR	<20 mg starting dose vs 20 mg starting dose -> <20 mg starting dose vs 20 mg starting dose	Original 20 mg starting dose; target Boku 2026 comparison group (20 mg)	no continuity correction; source groups mutually exclusive; any category/arm aggregation is documented in effect notes	yes	Boku 2026, p. Supplement DOCX, Results, Supplementary Table 1	Verified against the source; included in quantitative synthesis; no additional transformation.
How 2021	Disease control rate	RR	calculated harmonized from reported categories or arms; prespecified binary RR derivation	Log risk ratio and sampling variance derived from two-arm event counts	RR	<20 mg starting dose vs 20 mg starting dose -> <20 mg starting dose vs 20 mg starting dose	Original 20 mg starting dose; target How 2021 comparison group (20 mg)	no continuity correction; source groups mutually exclusive; any category/arm aggregation is documented in effect notes	yes	How 2021, p. 19, Results, Table 3A	Verified against the source; included in quantitative synthesis; no additional transformation.
Zammarrelli 2023	Disease control rate	RR	calculated harmonized from reported categories or arms; prespecified binary RR derivation	Log risk ratio and sampling variance derived from two-arm event counts	RR	10 or 14 mg starting dose vs 20 mg starting dose -> 10 or 14 mg starting dose vs 20 mg starting dose	Original 20 mg starting dose; target Zammarrelli 2023 comparison group (20 mg)	no continuity correction; source groups mutually exclusive; any category/arm aggregation is documented in effect notes	yes	Zammarrelli 2023, p. 18, Results, Table 3	Verified against the source; included in quantitative synthesis; no additional transformation.
Ta 2025	Disease control	RR	calculated harmonized	Log risk ratio and	RR	<20 mg once daily vs	Original 20 mg once	no continuity	yes	Ta 2025, p. 4,	Verified against the

Study	Outcome	Original metric	Processing step	Derivation rule	Converted metric	Direction harmonization	Reference group handling	Assumption	Sensitivity requirement	Source location	Verification status
	rate		from reported categories or arms; prespecified binary RR derivation	sampling variance derived from two-arm event counts		20 mg once daily -> <20 mg once daily vs 20 mg once daily	daily; target Ta 2025 comparison group (20 mg)	correction; source groups mutually exclusive; any category/arm aggregation is documented in effect notes		Results, Table 2	source; included in quantitative synthesis; no additional transformation.
Corr 2025	Disease control rate	RR	calculated harmonized from reported categories or arms; prespecified binary RR derivation	Log risk ratio and sampling variance derived from two-arm event counts	RR	10/14/18 mg starting dose vs 20 mg starting dose -> 10/14/18 mg starting dose vs 20 mg starting dose	Original 20 mg starting dose; target Corr 2025 comparison group (20 mg)	no continuity correction; source groups mutually exclusive; any category/arm aggregation is documented in effect notes	yes	Corr 2025, p. 4, Results, Table 2	Verified against the source; included in quantitative synthesis; no additional transformation.
Boku 2026	Disease control rate	RR	calculated from reported arm events; prespecified binary RR derivation	Log risk ratio and sampling variance derived from two-arm event counts	RR	<20 mg starting dose vs 20 mg starting dose -> <20 mg starting dose vs 20 mg starting dose	Original 20 mg starting dose; target Boku 2026 comparison group (20 mg)	no continuity correction; source groups mutually exclusive; any category/arm aggregation is documented in effect notes	yes	Boku 2026, p. Supplement DOCX, Results, Supplementary Table 1	Verified against the source; included in quantitative synthesis; no additional transformation.
Zammarrelli 2023	Grade 3 or higher adverse event	RR	calculated from reported arm events; prespecified binary RR derivation	Log risk ratio and sampling variance derived from two-arm event counts	RR	10 or 14 mg starting dose vs 20 mg starting dose -> 10 or 14 mg starting dose vs 20 mg starting dose	Original 20 mg starting dose; target Zammarrelli 2023 comparison group (20 mg)	no continuity correction; source groups mutually exclusive; any category/arm aggregation is documented in effect notes	yes	Zammarrelli 2023, p. 18, Results, Table 3	Verified against the source; included in quantitative synthesis; no additional transformation.
Corr 2025	Grade 3 or higher adverse event	RR	calculated harmonized from reported categories or arms; prespecified binary RR derivation	Log risk ratio and sampling variance derived from two-arm event counts	RR	10/14/18 mg starting dose vs 20 mg starting dose -> 10/14/18 mg starting dose vs 20 mg starting dose	Original 20 mg starting dose; target Corr 2025 comparison group (20 mg)	no continuity correction; source groups mutually exclusive; any category/arm aggregation is documented in effect notes	yes	Corr 2025, p. 5, Results, Table 4	Verified against the source; included in quantitative synthesis; no additional transformation.
How 2021	Subsequent lenvatinib dose reduction	RR	calculated harmonized from reported categories or arms; prespecified binary RR derivation	Log risk ratio and sampling variance derived from two-arm event counts	RR	<20 mg starting dose vs 20 mg starting dose -> <20 mg starting dose vs 20 mg starting dose	Original 20 mg starting dose; target How 2021 comparison group (20 mg)	no continuity correction; source groups mutually exclusive; any category/arm aggregation is documented in effect notes	yes	How 2021, p. 18, Results, Table 2	Verified against the source; included in quantitative synthesis; no additional transformation.
Zammarrelli 2023	Subsequent lenvatinib dose reduction	RR	calculated from reported arm events; prespecified binary RR derivation	Log risk ratio and sampling variance derived from two-arm event counts	RR	10 or 14 mg starting dose vs 20 mg starting dose -> 10 or 14 mg starting dose vs 20 mg starting dose	Original 20 mg starting dose; target Zammarrelli 2023 comparison group (20 mg)	no continuity correction; source groups mutually exclusive; any category/arm aggregation is documented in effect notes	yes	Zammarrelli 2023, p. 15, Results, Table 1	Verified against the source; included in quantitative synthesis; no additional transformation.
Corr 2025	Subsequent lenvatinib dose reduction	RR	calculated from reported arm events; prespecified binary RR derivation	Log risk ratio and sampling variance derived from two-arm event counts	RR	10/14/18 mg starting dose vs 20 mg starting dose -> 10/14/18 mg starting dose vs 20 mg starting dose	Original 20 mg starting dose; target Corr 2025 comparison group (20 mg)	no continuity correction; source groups mutually exclusive; any category/arm aggregation is	yes	Corr 2025, p. 4, Results, Table 2	Verified against the source; included in quantitative synthesis; no additional transformation.

Study	Outcome	Original metric	Processing step	Derivation rule	Converted metric	Direction harmonization	Reference group handling	Assumption	Sensitivity requirement	Source location	Verification status
								documented in effect notes			
How 2021	Lenvatinib interruption or hold	RR	calculated from reported arm events; prespecified binary RR derivation	Log risk ratio and sampling variance derived from two-arm event counts	RR	<20 mg starting dose vs 20 mg starting dose -> <20 mg starting dose vs 20 mg starting dose	Original 20 mg starting dose; target How 2021 comparison group (20 mg)	no continuity correction; source groups mutually exclusive; any category/arm aggregation is documented in effect notes	yes	How 2021, p. 18, Results, Table 2	Verified against the source; included in quantitative synthesis; no additional transformation.
Zammarrelli 2023	Lenvatinib interruption or hold	RR	calculated harmonized from reported categories or arms; prespecified binary RR derivation	Log risk ratio and sampling variance derived from two-arm event counts	RR	10 or 14 mg starting dose vs 20 mg starting dose -> 10 or 14 mg starting dose vs 20 mg starting dose	Original 20 mg starting dose; target Zammarrelli 2023 comparison group (20 mg)	no continuity correction; source groups mutually exclusive; any category/arm aggregation is documented in effect notes	yes	Zammarrelli 2023, p. 15, Results, Table 1	Verified against the source; included in quantitative synthesis; no additional transformation.
How 2021	Permanent discontinuation due to toxicity or intolerance	RR	calculated harmonized from reported categories or arms; prespecified binary RR derivation	Log risk ratio and sampling variance derived from two-arm event counts	RR	<20 mg starting dose vs 20 mg starting dose -> <20 mg starting dose vs 20 mg starting dose	Original 20 mg starting dose; target How 2021 comparison group (20 mg)	no continuity correction; source groups mutually exclusive; any category/arm aggregation is documented in effect notes	yes	Source report; exact location recorded during extraction toxicity-attributed outcome definition	Verified against the source; included in quantitative synthesis; no additional transformation.
Zammarrelli 2023	Permanent discontinuation due to toxicity or intolerance	RR	calculated harmonized from reported categories or arms; prespecified binary RR derivation	Log risk ratio and sampling variance derived from two-arm event counts	RR	10 or 14 mg starting dose vs 20 mg starting dose -> 10 or 14 mg starting dose vs 20 mg starting dose	Original 20 mg starting dose; target Zammarrelli 2023 comparison group (20 mg)	no continuity correction; source groups mutually exclusive; any category/arm aggregation is documented in effect notes	yes	Source report; exact location recorded during extraction toxicity-attributed outcome definition	Not derived: a single-zero-cell risk ratio would require an unplanned correction.
Corr 2025	Permanent discontinuation due to toxicity or intolerance	RR	calculated harmonized from reported categories or arms; prespecified binary RR derivation	Log risk ratio and sampling variance derived from two-arm event counts	RR	10/14/18 mg starting dose vs 20 mg starting dose -> 10/14/18 mg starting dose vs 20 mg starting dose	Original 20 mg starting dose; target Corr 2025 comparison group (20 mg)	no continuity correction; source groups mutually exclusive; any category/arm aggregation is documented in effect notes	yes	Source report; exact location recorded during extraction toxicity-attributed outcome definition	Verified against the source; included in quantitative synthesis; no additional transformation.
Corr 2025	Permanent discontinuation, all cause	RR	calculated from reported arm events; prespecified binary RR derivation	Log risk ratio and sampling variance derived from two-arm event counts	RR	10/14/18 mg starting dose vs 20 mg starting dose -> 10/14/18 mg starting dose vs 20 mg starting dose	Original 20 mg starting dose; target Corr 2025 comparison group (20 mg)	no continuity correction; source groups mutually exclusive; any category/arm aggregation is documented in effect notes	yes	Source report; exact location recorded during extraction all-cause outcome definition	Verified against the source; retained as a separate analysis layer or narrative synthesis.
How 2021	All-cause hospitalization	RR	calculated harmonized from reported categories or arms; prespecified binary RR derivation	Log risk ratio and sampling variance derived from two-arm event counts	RR	<20 mg starting dose vs 20 mg starting dose -> <20 mg starting dose vs 20 mg starting dose	Original 20 mg starting dose; target How 2021 comparison group (20 mg)	no continuity correction; source groups mutually exclusive; any category/arm aggregation is documented in effect notes	yes	Source report; exact location recorded during extraction all-cause outcome definition	Verified against the source; retained as a separate analysis layer or narrative synthesis.
Ta 2025	All-cause hospitalization	RR	calculated harmonized from reported	Log risk ratio and sampling variance	RR	<20 mg once daily vs 20 mg once daily ->	Original 20 mg once daily; target Ta 2025	no continuity correction; source	yes	Source report; exact location	Verified against the source; retained as a

Study	Outcome	Original metric	Processing step	Derivation rule	Converted metric	Direction harmonization	Reference group handling	Assumption	Sensitivity requirement	Source location	Verification status
			categories or arms; prespecified binary RR derivation	derived from two-arm event counts		<20 mg once daily vs 20 mg once daily	comparison group (20 mg)	groups mutually exclusive; any category/arm aggregation is documented in effect notes		recorded during extraction all-cause outcome definition	separate analysis layer or narrative synthesis.
How 2021	Toxicity-related hospitalization	RR	calculated from reported arm events; prespecified binary RR derivation	Log risk ratio and sampling variance derived from two-arm event counts	RR	<20 mg starting dose vs 20 mg starting dose -> <20 mg starting dose vs 20 mg starting dose	Original 20 mg starting dose; target How 2021 comparison group (20 mg)	no continuity correction; source groups mutually exclusive; any category/arm aggregation is documented in effect notes	yes	Source report; exact location recorded during extraction toxicity-attributed outcome definition	Verified against the source; retained as a separate analysis layer or narrative synthesis.
Ta 2025	Six-month progression-free survival	RR	calculated from reported arm events; prespecified binary RR derivation	Log risk ratio and sampling variance derived from two-arm event counts	RR	<20 mg once daily vs 20 mg once daily -> <20 mg once daily vs 20 mg once daily	Original 20 mg once daily; target Ta 2025 comparison group (20 mg)	no continuity correction; source groups mutually exclusive; any category/arm aggregation is documented in effect notes	yes	Ta 2025, p. 4, Results, Table 2	Verified against the source; retained as a separate analysis layer or narrative synthesis.
Dzienny 2026	Objective response rate	RR	calculated from reported arm events; prespecified binary RR derivation	Log risk ratio and sampling variance derived from two-arm event counts	RR	<=10 mg starting dose vs >10 mg starting dose -> <=10 mg starting dose vs >10 mg starting dose	Original >10 mg starting dose; target Dzienny 2026 comparison group (>10 mg)	no continuity correction; source groups mutually exclusive; any category/arm aggregation is documented in effect notes	yes	Dzienny 2026, p. 4; Supplement slide 2, Results, Table 2; Table S2	Verified against the source; retained as a separate analysis layer or narrative synthesis.
Dzienny 2026	Disease control rate	RR	calculated harmonized from reported categories or arms; prespecified binary RR derivation	Log risk ratio and sampling variance derived from two-arm event counts	RR	<=10 mg starting dose vs >10 mg starting dose -> <=10 mg starting dose vs >10 mg starting dose	Original >10 mg starting dose; target Dzienny 2026 comparison group (>10 mg)	no continuity correction; source groups mutually exclusive; any category/arm aggregation is documented in effect notes	yes	Dzienny 2026, p. 4; Supplement slide 2, Results, Table 2; Table S2	Verified against the source; retained as a separate analysis layer or narrative synthesis.
Barbi 2025	Grade 2 or higher adverse event	RR	calculated harmonized from reported categories or arms; prespecified binary RR derivation	Log risk ratio and sampling variance derived from two-arm event counts	RR	<=10 mg starting dose vs >10 mg starting dose -> <=10 mg starting dose vs >10 mg starting dose	Original >10 mg starting dose; target Barbi 2025 comparison group (>10 mg)	no continuity correction; source groups mutually exclusive; any category/arm aggregation is documented in effect notes	yes	Barbi 2025, p. 5, Results, Table 2	Verified against the source; retained as a separate analysis layer or narrative synthesis.
Barbi 2025	Subsequent lenvatinib dose reduction	RR	calculated from reported arm events; prespecified binary RR derivation	Log risk ratio and sampling variance derived from two-arm event counts	RR	<=10 mg starting dose vs >10 mg starting dose -> <=10 mg starting dose vs >10 mg starting dose	Original >10 mg starting dose; target Barbi 2025 comparison group (>10 mg)	no continuity correction; source groups mutually exclusive; any category/arm aggregation is documented in effect notes	yes	Barbi 2025, p. 5, Results, Table 2	Verified against the source; retained as a separate analysis layer or narrative synthesis.
Barbi 2025	Lenvatinib interruption or hold	RR	calculated from reported arm events; prespecified binary RR derivation	Log risk ratio and sampling variance derived from two-arm event counts	RR	<=10 mg starting dose vs >10 mg starting dose -> <=10 mg starting dose vs >10 mg starting dose	Original >10 mg starting dose; target Barbi 2025 comparison group (>10 mg)	no continuity correction; source groups mutually exclusive; any category/arm aggregation is documented in effect	yes	Barbi 2025, p. 5, Results, Table 2	Verified against the source; retained as a separate analysis layer or narrative synthesis.

Study	Outcome	Original metric	Processing step	Derivation rule	Converted metric	Direction harmonization	Reference group handling	Assumption	Sensitivity requirement	Source location	Verification status
								notes			
Barbi 2025	Permanent discontinuation due to toxicity or intolerance	RR	calculated harmonized from reported categories or arms; prespecified binary RR derivation	Log risk ratio and sampling variance derived from two-arm event counts	RR	<=10 mg starting dose vs >10 mg starting dose -> <=10 mg starting dose vs >10 mg starting dose	Original >10 mg starting dose; target Barbi 2025 comparison group (>10 mg)	no continuity correction; source groups mutually exclusive; any category/arm aggregation is documented in effect notes	yes	Source report; exact location recorded during extraction toxicity-attributed outcome definition	Verified against the source; retained as a separate analysis layer or narrative synthesis.
Dzienny 2026	Permanent discontinuation due to toxicity or intolerance	RR	calculated harmonized from reported categories or arms; prespecified binary RR derivation	Log risk ratio and sampling variance derived from two-arm event counts	RR	<=10 mg starting dose vs >10 mg starting dose -> <=10 mg starting dose vs >10 mg starting dose	Original >10 mg starting dose; target Dzienny 2026 comparison group (>10 mg)	no continuity correction; source groups mutually exclusive; any category/arm aggregation is documented in effect notes	yes	Source report; exact location recorded during extraction toxicity-attributed outcome definition	Verified against the source; retained as a separate analysis layer or narrative synthesis.
Dzienny 2026	Permanent discontinuation due to toxicity or intolerance	RR	calculated harmonized from reported categories or arms; prespecified binary RR derivation	Log risk ratio and sampling variance derived from two-arm event counts	RR	<=10 mg starting dose vs >10 mg starting dose -> <=10 mg starting dose vs >10 mg starting dose	Original >10 mg starting dose; target Dzienny 2026 comparison group (>10 mg)	no continuity correction; source groups mutually exclusive; any category/arm aggregation is documented in effect notes	yes	Dzienny 2026, p. Supplement slide 6, Results, Table S6	Verified against the source; retained in the prespecified sensitivity analysis.
Nagase 2025	Objective response rate	RR	calculated from exact reported percent and group n; prespecified binary RR derivation	Log risk ratio and sampling variance derived from two-arm event counts	RR	first-8-week RDI >=50% vs first-8-week RDI <50% -> first-8-week RDI >=50% vs first-8-week RDI <50%	Original first-8-week RDI <50%; target Nagase 2025 comparison group (RDI)-LOW	no continuity correction; source groups mutually exclusive; any category/arm aggregation is documented in effect notes	yes	Nagase 2025, p. 5, Results, Results	Verified against the source; retained as a separate analysis layer or narrative synthesis.
Nagase 2025	Disease control rate	RR	calculated from exact reported percent and group n; prespecified binary RR derivation	Log risk ratio and sampling variance derived from two-arm event counts	RR	first-8-week RDI >=50% vs first-8-week RDI <50% -> first-8-week RDI >=50% vs first-8-week RDI <50%	Original first-8-week RDI <50%; target Nagase 2025 comparison group (RDI)-LOW	no continuity correction; source groups mutually exclusive; any category/arm aggregation is documented in effect notes	yes	Nagase 2025, p. 5, Results, Results	Verified against the source; retained as a separate analysis layer or narrative synthesis.
Zammarrelli 2023	Progression-free survival	HR	reference reciprocal then prespecified log-CI derivation	Log ratio and standard error derived from the reported estimate and 95% CI	HR	20 mg vs 10/14 mg -> <20 mg vs 20 mg	Original 20 mg; target Zammarrelli 2023 comparison group (20 mg)	no continuity correction; source groups mutually exclusive; any category/arm aggregation is documented in effect notes	yes	Zammarrelli 2023, p. 19, Results, Table 4	Verified against the source; included in quantitative synthesis; no additional transformation.
Zammarrelli 2023	Overall survival	HR	reference reciprocal then prespecified log-CI derivation	Log ratio and standard error derived from the reported estimate and 95% CI	HR	20 mg vs 10/14 mg -> <20 mg vs 20 mg	Original 20 mg; target Zammarrelli 2023 comparison group (20 mg)	no continuity correction; source groups mutually exclusive; any category/arm aggregation is documented in effect notes	yes	Zammarrelli 2023, p. 19, Results, Table 4	Verified against the source; retained as a separate analysis layer or narrative synthesis.
Ta 2025	Progression-free survival	HR	reference reciprocal then prespecified log-CI derivation	Log ratio and standard error derived from the reported estimate and	HR	20 mg daily vs <20 mg daily -> <20 mg daily vs 20 mg daily	Original 20 mg daily; target Ta 2025 comparison group (20	no continuity correction; source groups mutually	yes	Ta 2025, p. 4, Results, Results/Kaplan-	Verified against the source; included in quantitative synthesis;

Study	Outcome	Original metric	Processing step	Derivation rule	Converted metric	Direction harmonization	Reference group handling	Assumption	Sensitivity requirement	Source location	Verification status
				95% CI			mg)	exclusive; any category/arm aggregation is documented in effect notes		Meier comparison	no additional transformation.
Boku 2026	Progression-free survival	HR	prespecified log-CI derivation	Log ratio and standard error derived from the reported estimate and 95% CI	HR	<20 mg vs 20 mg -> <20 mg vs 20 mg	Original 20 mg; target Boku 2026 comparison group (20 mg)	no continuity correction; source groups mutually exclusive; any category/arm aggregation is documented in effect notes	yes	Boku 2026, pp. 6,14; published XML, Results, Table 3	Verified against the source; retained as a separate analysis layer or narrative synthesis.
Boku 2026	Overall survival	HR	prespecified log-CI derivation	Log ratio and standard error derived from the reported estimate and 95% CI	HR	<20 mg vs 20 mg -> <20 mg vs 20 mg	Original 20 mg; target Boku 2026 comparison group (20 mg)	no continuity correction; source groups mutually exclusive; any category/arm aggregation is documented in effect notes	yes	Boku 2026, pp. 6,14; published XML, Results, Table 4/VOR XML	Verified against the source; retained as a separate analysis layer or narrative synthesis.
Barbi 2025	Progression-free survival	HR	prespecified log-CI derivation	Log ratio and standard error derived from the reported estimate and 95% CI	HR	<=10 mg vs >10 mg -> <=10 mg vs >10 mg	Original >10 mg; target Barbi 2025 comparison group (>10 mg)	no continuity correction; source groups mutually exclusive; any category/arm aggregation is documented in effect notes	yes	Barbi 2025, p. 7, Results, Figure 3; Table 3	Verified against the source; retained as a separate analysis layer or narrative synthesis.
Dzienny 2026	Progression-free survival	HR	prespecified log-CI derivation	Log ratio and standard error derived from the reported estimate and 95% CI	HR	<=10 mg vs >10 mg -> <=10 mg vs >10 mg	Original >10 mg; target Dzienny 2026 comparison group (>10 mg)	no continuity correction; source groups mutually exclusive; any category/arm aggregation is documented in effect notes	yes	Dzienny 2026, p. 4, Results, Figure 2a	Verified against the source; retained as a separate analysis layer or narrative synthesis.
Nagase 2025	Progression-free survival	HR	prespecified log-CI derivation	Log ratio and standard error derived from the reported estimate and 95% CI	HR	8w-RDI >=50% vs <50% -> 8w-RDI >=50% vs <50%	Original <50%; target Nagase 2025 comparison group (RDI)-LOW	no continuity correction; source groups mutually exclusive; any category/arm aggregation is documented in effect notes	yes	Nagase 2025, p. 5–6, Results, Figure 3A; Table 3	Verified against the source; retained as a separate analysis layer or narrative synthesis.
Nagase 2025	Progression-free survival	HR	prespecified log-CI derivation	Log ratio and standard error derived from the reported estimate and 95% CI	HR	8w-RDI >=50% vs <50% -> 8w-RDI >=50% vs <50%	Original <50%; target Nagase 2025 comparison group (RDI)-LOW	no continuity correction; source groups mutually exclusive; any category/arm aggregation is documented in effect notes	yes	Nagase 2025, p. 6, Results, Table 3	Verified against the source; retained as a separate analysis layer or narrative synthesis.
Nagase 2025	Overall survival	HR	prespecified log-CI derivation	Log ratio and standard error derived from the reported estimate and 95% CI	HR	8w-RDI >=50% vs <50% -> 8w-RDI >=50% vs <50%	Original <50%; target Nagase 2025 comparison group (RDI)-LOW	no continuity correction; source groups mutually exclusive; any category/arm aggregation is documented in effect notes	yes	Nagase 2025, pp. 1,5–6, Results, Abstract; Figure 3B	Verified against the source; retained as a separate analysis layer or narrative synthesis.

Study	Outcome	Original metric	Processing step	Derivation rule	Converted metric	Direction harmonization	Reference group handling	Assumption	Sensitivity requirement	Source location	Verification status
How 2021	Objective response rate	RR	response-denominator sensitivity check with the prespecified log risk-ratio and sampling-variance formula for two-arm event counts	Log risk ratio and sampling variance derived from two-arm event counts: log RR=log[(a/n1)/(c/n0)]; variance=1/a-1/n1+1/c-1/n0	RR	<20 mg starting dose vs 20 mg starting dose -> <20 mg starting dose vs 20 mg starting dose	Original 20 mg starting dose; target How 2021 comparison group (20 mg)	Non-evaluable patients remain in the total starting-dose-arm denominator; events unchanged; no continuity correction or imputation.	yes	How 2021, p. 18–19, Results, Table 2 and Table 3A	Verified against the source; retained in the prespecified sensitivity analysis.
Takahashi 2026	Objective response rate	events/denominators	prespecified binary RR formula	Log risk ratio and sampling variance derived from two-arm event counts; log RR SE=sqrt(1/a-1/n1+1/c-1/n0)	RR	>=56% vs <56% -> >=56% vs <56%	Original <56%; target <56%	no continuity correction; mutually exclusive response-evaluable groups	no quantitative execution	Takahashi 2026, p. PDF p.4 (journal 707), Results, Figure S3A cited	Verified against the source; narrative only.
Takahashi 2026	Progression-free survival	HR	reciprocal reference harmonization; no pooling	HR target=1/HR source; CI target=(1/upper,1/lower)	HR	<56% vs >=56% -> >=56% vs <56%	Original >=56%; target <56%	source table reference order is explicit	no quantitative execution	Takahashi 2026, p. PDF p.6 (journal 709), Results, Table 3	Verified against the source; narrative only.
Takahashi 2026	Progression-free survival	HR	none	none	HR	unresolved -> >=56% vs <56%	Original unclear; target <56%	no direction assumption permitted	no	Takahashi 2026, p. PDF p.4 (journal 707), Results, Figure S3B cited but unavailable	No quantitative transformation; narrative only.

Note: All transformations preserve the raw extracted value and the prespecified effect direction; no hidden continuity correction or unplanned conversion was used.

Supplementary Table C6. Full-text exclusions

Report number	Citation	DOI or PMID	Full text assessed	Exclusion category	Specific exclusion reason	Decision date	Screening review process	Notes
1	Blackman et al. (2023). Current practices using pembrolizumab and lenvatinib for advanced uterine cancer at a high-volume gynecologic oncology referral center in the Southeast.	DOI: 10.1016/j.ygyno.2023.06.241	Yes	No usable effect size	23-patient cohort mentions starting-dose practice and a non-significant PFS comparison but provides no arm-level counts, HR/CI, convertible event data, or usable KM curve.	2026–07–12	Two reviewers independently assessed the full text; disagreements were resolved by consensus.	Full report available.
2	Tyulyandina et al. (2021). Effectiveness and safety of lenvatinib and pembrolizumab (LenPem) therapy for endometrial cancer (EC): results from a Russian multicenter database.	DOI: 10.1136/ijgc-2021-ESGO.197	Yes	Wrong comparator	Single-arm regimen outcomes; reductions after toxicity are not baseline starting-dose groups.	2026–07–12	Two reviewers independently assessed the full text; disagreements were resolved by consensus.	Full report and companion report available.
3	Ozawa et al. (2023). The efficacy and safety of lenvatinib plus pembrolizumab in patients with recurrent endometrial cancer: a Japanese single institutional experience.	DOI: 10.1136/ijgc-2023-IGCS.247	Yes	Wrong comparator	Overall LP outcomes only; no eligible starting-dose or RDI comparison.	2026–07–12	Two reviewers independently assessed the full text; disagreements were resolved by consensus.	Full report available.
4	Bello Roufai et al. (2025). Efficacy and safety of pembrolizumab–lenvatinib in metastatic endometrial cancer: results from the French multicenter early access program (LARENA).	DOI: 10.1016/j.annonc.2025.08.1759	Yes	Wrong comparator	Starting-dose distribution reported, but outcomes are not stratified by baseline starting dose.	2026–07–12	Two reviewers independently assessed the full text; disagreements were resolved by consensus.	Full report available.
5	Mendez et al. (2025). Evaluation of factors leading to survival benefits in patients with advanced endometrial cancer treated with combination therapy of pembrolizumab plus lenvatinib.	DOI: 10.1016/j.ygyno.2025.04.429	Yes	Wrong exposure or intervention	Prognostic-factor study; no eligible baseline starting-dose or RDI effect.	2026–07–12	Two reviewers independently assessed the full text; disagreements were resolved by consensus.	Full report available.

Report number	Citation	DOI or PMID	Full text assessed	Exclusion category	Specific exclusion reason	Decision date	Screening review process	Notes
6	Ta et al. (2024). Outcomes of reduced dosing of lenvatinib with pembrolizumab for advanced endometrial cancer.	DOI: 10.1200/JCO.2024.42.16 suppl.5588	Yes	duplicate cohort	Conference report is the antecedent/companion of screening record Kaiser cohort; retained as supporting material, not counted twice.	2026–07–12	Two reviewers independently assessed the full text; disagreements were resolved by consensus.	Full report available.
7	Madár et al. (2026). Systemic Treatment Strategies Beyond Chemotherapy in Recurrent or Advanced Endometrial Cancer: A Systematic Review and Meta-Analysis.	DOI: 10.3390/cancers18132091	Yes	review or meta analysis	Broad systemic-treatment review, not an eligible original baseline dose-comparison report; used only for contextual and backward citation chasing.	2026–07–12	Two reviewers independently assessed the full text; disagreements were resolved by consensus.	Full report available.

Note: Reports were excluded using prespecified, auditable reasons; companion reports and duplicate cohorts were not counted as independent studies.

Supplementary Table C7. Analysis-set membership and study roles

Study or evidence gap	Outcome	Effect descriptor	Main analysis	Sensitivity analysis	Subgroup analysis	Exploratory or supplementary role	Narrative-only role	Exclusion from quantitative synthesis reason	Decision rationale
Zammarrelli 2023	PFS	Zammarrelli 2023: PFS	No	No	No prespecified subgroup meta-analysis	Narrative plus exploratory pooling	No	Not excluded from its prespecified synthesis	Primary clinical outcome but only two unadjusted observational cohorts; pooling is explicitly exploratory. Estimand: Starting dose <20 mg versus 20 mg
Ta 2025	PFS	Ta 2025: PFS	No	No	No prespecified subgroup meta-analysis	Narrative plus exploratory pooling	No	Not excluded from its prespecified synthesis	Primary clinical outcome but only two unadjusted observational cohorts; pooling is explicitly exploratory. Estimand: Starting dose <20 mg versus 20 mg
Boku 2026	PFS	Boku 2026: PFS	No	No	No prespecified subgroup meta-analysis	Not applicable	Narrative only	Adjusted layer is k = 1 and cannot mix with reported unadjusted HR.	Adjusted layer is k = 1 and cannot mix with reported unadjusted HR. Estimand: Starting dose <20 mg versus 20 mg
Evidence gap	PFS	No derivable comparative PFS effect	No	No	No prespecified subgroup meta-analysis	Narrative plus exploratory pooling	Narrative plus exploratory pooling	No HR was derived from the published Kaplan–Meier curve under the prespecified analysis plan.	No HR was derived from the published Kaplan–Meier curve under the prespecified analysis plan. Estimand: Starting dose <20 mg versus 20 mg
Ta 2025	PFS6	Ta 2025: PFS6	No	No	No prespecified subgroup meta-analysis	Not applicable	Narrative only	Single fixed-time contrast cannot be converted to or pooled with HR.	Single fixed-time contrast cannot be converted to or pooled with HR. Estimand: Starting dose <20 mg versus 20 mg
Zammarrelli 2023	OS	Zammarrelli 2023: OS	No	No	No prespecified subgroup meta-analysis	Not applicable	Narrative only	Reported-unadjusted OS layer is k = 1.	Reported-unadjusted OS layer is k = 1. Estimand: Starting dose <20 mg versus 20 mg
Boku 2026	OS	Boku 2026: OS	No	No	No prespecified subgroup meta-analysis	Not applicable	Narrative only	Adjusted OS layer is k = 1 and remains separate.	Adjusted OS layer is k = 1 and remains separate. Estimand: Starting dose <20 mg versus 20 mg
Evidence gap	OS	No derivable comparative OS effect	No	No	No prespecified subgroup meta-analysis	Narrative plus exploratory pooling	Narrative plus exploratory pooling	No HR was derived from the published Kaplan–Meier curve under the prespecified analysis plan.	No HR was derived from the published Kaplan–Meier curve under the prespecified analysis plan. Estimand: Starting dose <20 mg versus 20 mg
How 2021	ORR	How 2021: ORR	No	No	No prespecified subgroup meta-analysis	Not applicable	No	Not excluded from its prespecified synthesis	Five independent cohorts; denominator-definition sensitivity is prespecified and result-independent. Estimand: Starting dose <20 mg versus 20 mg
Zammarrelli 2023	ORR	Zammarrelli 2023: ORR	No	No	No prespecified subgroup meta-analysis	Not applicable	No	Not excluded from its prespecified synthesis	Five independent cohorts; denominator-definition sensitivity is prespecified and result-independent. Estimand: Starting dose <20 mg versus 20 mg
Ta 2025	ORR	Ta 2025: ORR	No	No	No prespecified subgroup meta-analysis	Not applicable	No	Not excluded from its prespecified synthesis	Five independent cohorts; denominator-definition sensitivity is prespecified and result-independent. Estimand: Starting dose <20 mg versus 20 mg
Corr 2025	ORR	Corr 2025: ORR	No	No	No prespecified subgroup meta-analysis	Not applicable	No	Not excluded from its prespecified synthesis	Five independent cohorts; denominator-definition sensitivity is prespecified and result-independent. Estimand: Starting dose <20 mg

Study or evidence gap	Outcome	Effect descriptor	Main analysis	Sensitivity analysis	Subgroup analysis	Exploratory or supplementary role	Narrative-only role	Exclusion from quantitative synthesis reason	Decision rationale
									versus 20 mg
Boku 2026	ORR	Boku 2026: ORR	No	No	No prespecified subgroup meta-analysis	Not applicable	No	Not excluded from its prespecified synthesis	Five independent cohorts; denominator-definition sensitivity is prespecified and result-independent. Estimand: Starting dose <20 mg versus 20 mg
How 2021	DCR	How 2021: DCR	No	No	No prespecified subgroup meta-analysis	Not applicable	No	Not excluded from its prespecified synthesis	Five cohorts share the harmonized CR+PR+SD construct; still labelled supplementary because source terminology varied. Estimand: Starting dose <20 mg versus 20 mg
Zammarrelli 2023	DCR	Zammarrelli 2023: DCR	No	No	No prespecified subgroup meta-analysis	Not applicable	No	Not excluded from its prespecified synthesis	Five cohorts share the harmonized CR+PR+SD construct; still labelled supplementary because source terminology varied. Estimand: Starting dose <20 mg versus 20 mg
Ta 2025	DCR	Ta 2025: DCR	No	No	No prespecified subgroup meta-analysis	Not applicable	No	Not excluded from its prespecified synthesis	Five cohorts share the harmonized CR+PR+SD construct; still labelled supplementary because source terminology varied. Estimand: Starting dose <20 mg versus 20 mg
Corr 2025	DCR	Corr 2025: DCR	No	No	No prespecified subgroup meta-analysis	Not applicable	No	Not excluded from its prespecified synthesis	Five cohorts share the harmonized CR+PR+SD construct; still labelled supplementary because source terminology varied. Estimand: Starting dose <20 mg versus 20 mg
Boku 2026	DCR	Boku 2026: DCR	No	No	No prespecified subgroup meta-analysis	Not applicable	No	Not excluded from its prespecified synthesis	Five cohorts share the harmonized CR+PR+SD construct; still labelled supplementary because source terminology varied. Estimand: Starting dose <20 mg versus 20 mg
Zammarrelli 2023	AE3	Zammarrelli 2023: AE3	No	No	No prespecified subgroup meta-analysis	Narrative plus exploratory pooling	No	Not excluded from its prespecified synthesis	Two compatible cohorts; retrospective underascertainment precludes a confirmatory role. Estimand: Starting dose <20 mg versus 20 mg
Corr 2025	AE3	Corr 2025: AE3	No	No	No prespecified subgroup meta-analysis	Narrative plus exploratory pooling	No	Not excluded from its prespecified synthesis	Two compatible cohorts; retrospective underascertainment precludes a confirmatory role. Estimand: Starting dose <20 mg versus 20 mg
How 2021	REDUCTION	How 2021: REDUCTION	No	No	No prespecified subgroup meta-analysis	Not applicable	No	Not excluded from its prespecified synthesis	Three unique-patient binary contrasts answer the main tolerability question; recurrent-event rate evidence stays separate. Estimand: Starting dose <20 mg versus 20 mg
Zammarrelli 2023	REDUCTION	Zammarrelli 2023: REDUCTION	No	No	No prespecified subgroup meta-analysis	Not applicable	No	Not excluded from its prespecified synthesis	Three unique-patient binary contrasts answer the main tolerability question; recurrent-event rate evidence stays separate. Estimand: Starting dose <20 mg versus 20 mg
Corr 2025	REDUCTION	Corr 2025: REDUCTION	No	No	No prespecified subgroup meta-analysis	Not applicable	No	Not excluded from its prespecified synthesis	Three unique-patient binary contrasts answer the main tolerability question; recurrent-event rate evidence stays separate.

Study or evidence gap	Outcome	Effect descriptor	Main analysis	Sensitivity analysis	Subgroup analysis	Exploratory or supplementary role	Narrative-only role	Exclusion from quantitative synthesis reason	Decision rationale
									Estimand: Starting dose <20 mg versus 20 mg
How 2021	INTERRUPTION	How 2021: INTERRUPTION	No	No	No prespecified subgroup meta-analysis	Not applicable	No	Not excluded from its prespecified synthesis	Two cohorts use compatible interruption/hold constructs; small k mandates an exploratory label. Estimand: Starting dose <20 mg versus 20 mg
Zammarrelli 2023	INTERRUPTION	Zammarrelli 2023: INTERRUPTION	No	No	No prespecified subgroup meta-analysis	Not applicable	No	Not excluded from its prespecified synthesis	Two cohorts use compatible interruption/hold constructs; small k mandates an exploratory label. Estimand: Starting dose <20 mg versus 20 mg
How 2021	DISC TOX	How 2021: DISC TOX	No	No	No prespecified subgroup meta-analysis	Narrative plus exploratory pooling	No	Not excluded from its prespecified synthesis	Combination-level toxicity/intolerance definitions are closest; lenvatinib-specific Zammarrelli 2023 is kept separate. Estimand: Starting dose <20 mg versus 20 mg
Corr 2025	DISC TOX	Corr 2025: DISC TOX	No	No	No prespecified subgroup meta-analysis	Narrative plus exploratory pooling	No	Not excluded from its prespecified synthesis	Combination-level toxicity/intolerance definitions are closest; lenvatinib-specific Zammarrelli 2023 is kept separate. Estimand: Starting dose <20 mg versus 20 mg
Zammarrelli 2023	DISC TOX	Zammarrelli 2023: DISC TOX	No	No	No prespecified subgroup meta-analysis	Not applicable	Narrative only	One single-zero lenvatinib-specific contrast cannot enter the conventional log-RR input.	One single-zero lenvatinib-specific contrast cannot enter the conventional log-RR input. Estimand: Starting dose <20 mg versus 20 mg
Corr 2025	DISC ALL	Corr 2025: DISC ALL	No	No	No prespecified subgroup meta-analysis	Not applicable	Narrative only	All-cause and toxicity-specific cessation are different constructs.	All-cause and toxicity-specific cessation are different constructs. Estimand: Starting dose <20 mg versus 20 mg
How 2021	HOSP ALL	How 2021: HOSP ALL	No	No	No prespecified subgroup meta-analysis	Not applicable	Narrative only	Reported follow-up and fixed six-month hospitalization windows are not interchangeable.	Reported follow-up and fixed six-month hospitalization windows are not interchangeable. Estimand: Starting dose <20 mg versus 20 mg
Ta 2025	HOSP ALL	Ta 2025: HOSP ALL	No	No	No prespecified subgroup meta-analysis	Not applicable	Narrative only	Reported follow-up and fixed six-month hospitalization windows are not interchangeable.	Reported follow-up and fixed six-month hospitalization windows are not interchangeable. Estimand: Starting dose <20 mg versus 20 mg
How 2021	HOSP TOX	How 2021: HOSP TOX	No	No	No prespecified subgroup meta-analysis	Not applicable	Narrative only	Only one toxicity-specific hospitalization contrast.	Only one toxicity-specific hospitalization contrast. Estimand: Starting dose <20 mg versus 20 mg
Evidence gap	PERSISTENCE	No compatible treatment-persistence effect	No	No	No prespecified subgroup meta-analysis	Not applicable	Narrative only	Reported medians, treatment duration, and time-to-event constructs were not compatible.	Medians, duration and time-to-event constructs are not compatible. Estimand: Starting dose <20 mg versus 20 mg
Barbi 2025	PFS	Barbi 2025: PFS	No	No	No prespecified subgroup meta-analysis	Not applicable	No	Adjusted threshold PFS is a one-cohort sensitivity result.	Adjusted threshold PFS is a one-cohort sensitivity result. Estimand: Starting dose ≤10 mg versus >10 mg
Dzienny 2026	PFS	Dzienny 2026: PFS	No	No	No prespecified subgroup meta-analysis	Not applicable	No	Unadjusted threshold PFS remains separate from adjusted Barbi 2025.	Unadjusted threshold PFS remains separate from adjusted Barbi 2025. Estimand: Starting dose ≤10 mg versus >10 mg

Study or evidence gap	Outcome	Effect descriptor	Main analysis	Sensitivity analysis	Subgroup analysis	Exploratory or supplementary role	Narrative-only role	Exclusion from quantitative synthesis reason	Decision rationale
Dzienny 2026	ORR	Dzienny 2026: ORR	No	No	No prespecified subgroup meta-analysis	Not applicable	No	One independent threshold cohort.	One independent threshold cohort. Estimand: Starting dose ≤10 mg versus >10 mg
Dzienny 2026	DCR	Dzienny 2026: DCR	No	No	No prespecified subgroup meta-analysis	Not applicable	No	One independent threshold cohort.	One independent threshold cohort. Estimand: Starting dose ≤10 mg versus >10 mg
Barbi 2025	REDUCTION	Barbi 2025: REDUCTION	No	No	No prespecified subgroup meta-analysis	Not applicable	No	One threshold cohort; Dzienny 2026 grouped value is qualitative only.	One threshold cohort; Dzienny 2026 grouped value is qualitative only. Estimand: Starting dose ≤10 mg versus >10 mg
Barbi 2025	INTERRUPTION	Barbi 2025: INTERRUPTION	No	No	No prespecified subgroup meta-analysis	Not applicable	No	One threshold cohort; Dzienny 2026 grouped value is qualitative only.	One threshold cohort; Dzienny 2026 grouped value is qualitative only. Estimand: Starting dose ≤10 mg versus >10 mg
Barbi 2025	DISC TOX	Barbi 2025: DISC TOX	No	Yes	No prespecified subgroup meta-analysis	Not applicable	No	Not excluded from its prespecified synthesis	Two independent threshold cohorts reported a compatible any-treatment toxicity-discontinuation construct. Estimand: Starting dose ≤10 mg versus >10 mg
Dzienny 2026	DISC TOX	Dzienny 2026: DISC TOX	No	Yes	No prespecified subgroup meta-analysis	Not applicable	No	Not excluded from its prespecified synthesis	Two independent threshold cohorts reported a compatible any-treatment toxicity-discontinuation construct. Estimand: Starting dose ≤10 mg versus >10 mg
Dzienny 2026	DISC TOX	Dzienny 2026: DISC TOX	No	No	No prespecified subgroup meta-analysis	Not applicable	No	Dependent alternative effect retained for construct sensitivity only.	Dependent alternative effect retained for construct sensitivity only. Estimand: Starting dose ≤10 mg versus >10 mg
Barbi 2025	AE2	Barbi 2025: AE2	No	No	No prespecified subgroup meta-analysis	Not applicable	No	No valid grade conversion exists.	No valid grade conversion exists. Estimand: Starting dose ≤10 mg versus >10 mg
Nagase 2025	PFS	Nagase 2025: PFS	No	No	No prespecified subgroup meta-analysis	Not applicable	No	Exact first-eight-week RDI but postbaseline exposure and k = 1.	Exact first-eight-week RDI but postbaseline exposure and k = 1. Estimand: First-8-week relative dose intensity
Nagase 2025	PFS	Nagase 2025: PFS	No	No	No prespecified subgroup meta-analysis	Not applicable	No	Exact first-eight-week RDI but postbaseline exposure and k = 1.	Exact first-eight-week RDI but postbaseline exposure and k = 1. Estimand: First-8-week relative dose intensity
Nagase 2025	OS	Nagase 2025: OS	No	No	No prespecified subgroup meta-analysis	Not applicable	No	Exact first-eight-week RDI but postbaseline exposure and k = 1.	Exact first-eight-week RDI but postbaseline exposure and k = 1. Estimand: First-8-week relative dose intensity
Nagase 2025	ORR	Nagase 2025: ORR	No	No	No prespecified subgroup meta-analysis	Not applicable	No	One postbaseline RDI cohort; result remains association-only.	One postbaseline RDI cohort; result remains association-only. Estimand: First-8-week relative dose intensity
Nagase 2025	DCR	Nagase 2025: DCR	No	No	No prespecified subgroup meta-analysis	Not applicable	No	One postbaseline RDI cohort; result remains association-only.	One postbaseline RDI cohort; result remains association-only. Estimand: First-8-week relative dose intensity
Evidence gap	AE3 PROCESS	No RDI-stratified grade 3 or higher adverse-event effect	No	No	No prespecified subgroup meta-analysis	Not applicable	Narrative only	Overall-cohort safety could not be attributed to relative-dose-intensity strata.	Overall-cohort safety cannot be attributed to RDI strata. Estimand: First-8-week relative dose intensity
Tochigi 2024	PFS	Tochigi 2024: progression-free	No	No	No prespecified subgroup meta-analysis	Not applicable	Narrative only	The non-8-week relative-dose-intensity definition could not be	Tochigi 2024 remains separate narrative context and could not

Study or evidence gap	Outcome	Effect descriptor	Main analysis	Sensitivity analysis	Subgroup analysis	Exploratory or supplementary role	Narrative-only role	Exclusion from quantitative synthesis reason	Decision rationale
		survival						converted to the first-8-week estimand.	enter the first-8-week relative dose intensity. Estimand: Non-8-week relative dose intensity (narrative)
How 2021	ORR	How 2021: ORR	No	Yes	No prespecified subgroup meta-analysis	Not applicable	No	Not excluded from its prespecified synthesis	Prespecified total-starting-dose-arm denominator sensitivity; mutually exclusive from the source-defined main ORR effect for this cohort. Estimand: Starting dose <20 mg versus 20 mg
Zammarrelli 2023	ORR	Zammarrelli 2023: ORR	No	Yes	No prespecified subgroup meta-analysis	Not applicable	No	Not excluded from its prespecified synthesis	Prespecified total-starting-dose-arm denominator sensitivity; mutually exclusive from the source-defined main ORR effect for this cohort. Estimand: Starting dose <20 mg versus 20 mg
Ta 2025	ORR	Ta 2025: ORR	No	Yes	No prespecified subgroup meta-analysis	Not applicable	No	Not excluded from its prespecified synthesis	Prespecified total-starting-dose-arm denominator sensitivity; mutually exclusive from the source-defined main ORR effect for this cohort. Estimand: Starting dose <20 mg versus 20 mg
Corr 2025	ORR	Corr 2025: ORR	No	Yes	No prespecified subgroup meta-analysis	Not applicable	No	Not excluded from its prespecified synthesis	Prespecified total-starting-dose-arm denominator sensitivity; mutually exclusive from the source-defined main ORR effect for this cohort. Estimand: Starting dose <20 mg versus 20 mg
Boku 2026	ORR	Boku 2026: ORR	No	Yes	No prespecified subgroup meta-analysis	Not applicable	No	Not excluded from its prespecified synthesis	Prespecified total-starting-dose-arm denominator sensitivity; mutually exclusive from the source-defined main ORR effect for this cohort. Estimand: Starting dose <20 mg versus 20 mg
Takahashi 2026	PFS	Takahashi 2026: PFS	No	No	No prespecified subgroup meta-analysis	Supplementary exploratory; narrative only	Yes; source-specific 56% RDI evidence	Independent first-8-week RDI cohort, but its same-cohort outcome-derived 56% threshold is incompatible with the prespecified 50% contrast; sparse adjusted model and postbaseline bias preclude pooling.	Independent first-8-week RDI cohort, but its same-cohort outcome-derived 56% threshold is incompatible with the prespecified 50% contrast; sparse adjusted model and postbaseline bias preclude pooling. Estimand First-8-week relative dose intensity, 56% cutoff (narrative).
Takahashi 2026	ORR	Takahashi 2026: ORR	No	No	No prespecified subgroup meta-analysis	Supplementary exploratory; narrative only	Yes; source-specific 56% RDI evidence	Independent first-8-week RDI cohort, but 56% is outcome-derived and incompatible with the prespecified 50% contrast; response-evaluable denominators also differ from total RDI strata.	Independent first-8-week RDI cohort, but 56% is outcome-derived and incompatible with the prespecified 50% contrast; response-evaluable denominators also differ from total RDI strata. Estimand First-8-week relative dose intensity, 56% cutoff (narrative).

Note: Analysis-set membership reflects compatibility and prespecified role, not causal validity.

Supplementary Table C8. Participant and baseline characteristics

Study	Group or exposure level	N	Age	Sex	Disease stage or severity	Key comorbidity	Baseline outcome value	Treatment background	Follow-up	Missing baseline data	Source report and location
How 2021	overall	70	median 65.5 years	female population	advanced or recurrent endometrial cancer	not systematically reported	ECOG 0–1: 91.3%; nutrition: not systematically reported	median 2 prior systemic lines	median 12.5 months	report-specific missingness retained	How 2021, p. 5, Results, Table 1
How 2021	<20 mg	54	median 66 years (range 31–77)	female population	advanced or recurrent endometrial cancer	not systematically reported	ECOG 0/1/2/3: 26/23/4/1; nutrition: not systematically reported	median 2 prior systemic lines (range 1–9)	overall median 12.5 months	report-specific missingness retained	How 2021, p. 5, Results, Table 1
How 2021	20 mg	16	median 64.5 years (range 55–77)	female population	advanced or recurrent endometrial cancer	not systematically reported	ECOG 0/1/2/3: 5/9/0/1; one ECOG missing; nutrition: not systematically reported	median 2 prior systemic lines (range 1–6)	overall median 12.5 months	report-specific missingness retained	How 2021, p. 5, Results, Table 1
Zammarrelli 2023	overall	43	median 67 years (range 54–85)	female population	advanced or recurrent endometrial cancer	not systematically reported	ECOG 0 n11; 1 n28; 2 n1; missing n3; nutrition: not systematically reported	median 2 prior cytotoxic lines	not consistently reported as one summary	report-specific missingness retained	Zammarrelli 2023, p. 6, Results, Table 1
Zammarrelli 2023	10/14 mg	20	median 69 years (range 61–85)	female population	advanced or recurrent endometrial cancer	not systematically reported	ECOG 0: 4/20; remaining ECOG categories retained in Table 1; nutrition: not systematically reported	median 2 prior systemic lines (range 1–10)	not reported by arm	report-specific missingness retained	Zammarrelli 2023, p. 6, Results, Table 1
Zammarrelli 2023	20 mg	23	median 64 years (range 54–78)	female population	advanced or recurrent endometrial cancer	not systematically reported	ECOG 0: 7/23; remaining ECOG categories retained in Table 1; nutrition: not systematically reported	median 2 prior systemic lines (range 1–8)	not reported by arm	report-specific missingness retained	Zammarrelli 2023, p. 6, Results, Table 1
Ta 2025	overall	369	median 68 years	female population	advanced or recurrent endometrial cancer	not systematically reported	ECOG unknown 60.4%; nutrition: not systematically reported	prior-line distributions differ across groups	follow-up to 2023–12–31/death/disenrollment	report-specific missingness retained	Ta 2025, p. 3, Results, Table 1
Ta 2025	20 mg daily	204	median 68 years (IQR 10)	female population	advanced or recurrent endometrial cancer	not systematically reported	ECOG 0/1/2/3/unknown: 34/42/17/2/109; nutrition: not systematically reported	median 1 prior line (IQR 1)	follow-up to 2023–12–31/death/disenrollment	report-specific missingness retained	Ta 2025, p. 3, Results, Table 1
Ta 2025	Published reduced group; see C2	154	median 68 years (IQR 11)	female population	advanced or recurrent endometrial cancer	not systematically reported	ECOG 0/1/2/3/unknown: 17/18/5/4/110; nutrition: not systematically reported	median 1 prior line (IQR 1)	follow-up to 2023–12–31/death/disenrollment	report-specific missingness retained	Ta 2025, p. 3, Results, Table 1
Ta 2025	20 mg five days/week; descriptive only, excluded from pools	11	median 69 years (IQR 12)	female population	advanced or recurrent endometrial cancer	not systematically reported	ECOG 0/1/2/3/unknown: 3/4/0/0/4; nutrition: not systematically reported	median 1 prior line (IQR 0)	follow-up to 2023–12–31/death/disenrollment	report-specific missingness retained	Ta 2025, p. 3, Results, Table 1
Corr 2025	overall	188	median 69.0 years	female population	advanced or recurrent endometrial cancer	not systematically reported	not reported in main baseline table; nutrition: not systematically reported	median regimen number 3	median 18.4 months	report-specific missingness retained	Corr 2025, p. 3, Results, Tables 1–2
Corr 2025	10 mg	34	median 71.1 years (IQR 67.0–79.5)	female population	advanced or recurrent endometrial cancer	not systematically reported	not reported; nutrition: not systematically reported	median regimen number 3 (IQR 2–3)	overall median 18.4 months	report-specific missingness retained	Corr 2025, p. 3, Results, Tables 1–2
Corr 2025	14 mg	89	median 69.2 years	female	advanced or	not	not reported; nutrition:	median regimen	overall median 18.4 months	report-specific	Corr 2025, p.

Study	Group or exposure level	N	Age	Sex	Disease stage or severity	Key comorbidity	Baseline outcome value	Treatment background	Follow-up	Missing baseline data	Source report and location
			(IQR 62.5–74.1)	population	recurrent endometrial cancer	systematically reported	not systematically reported	number 3 (IQR 2–4)		missingness retained	3, Results, Tables 1–2
Corr 2025	18 mg	28	median 66.4 years (IQR 62.3–69.0)	female population	advanced or recurrent endometrial cancer	not systematically reported	not reported; nutrition: not systematically reported	median regimen number 3 (IQR 2–4)	overall median 18.4 months	report-specific missingness retained	Corr 2025, p. 3, Results, Tables 1–2
Corr 2025	20 mg	37	median 68.7 years (IQR 64.1–72.5)	female population	advanced or recurrent endometrial cancer	not systematically reported	not reported; nutrition: not systematically reported	median regimen number 3 (IQR 2–4)	overall median 18.4 months	report-specific missingness retained	Corr 2025, p. 3, Results, Tables 1–2
Boku 2026	<20 mg	17	median 76.7 years	female population	advanced or recurrent endometrial cancer	not systematically reported	ECOG 0: 4/17 (23.5%); nutrition: not systematically reported	all age >=75 patients were in this group	overall median follow-up 27.6 months	report-specific missingness retained	Boku 2026, p. 4–5, Results, Table 1; Tables 3–4; Supplementary Table 1
Boku 2026	20 mg	16	median 60.0 years	female population	advanced or recurrent endometrial cancer	not systematically reported	ECOG 0: 13/16 (81.3%); nutrition: not systematically reported	substantial confounding by indication	overall median follow-up 27.6 months	report-specific missingness retained	Boku 2026, p. 4–5, Results, Table 1; Tables 3–4; Supplementary Table 1
Barbi 2025	<=10 mg	57	median 71.2 years (IQR 67.5–78.2)	female population	advanced or recurrent endometrial cancer	not systematically reported	ECOG 0/1/2/3: 23/17/15/2; nutrition: not systematically reported	prior lines 0/1/2/>=3: 2/32/18/5	overall median 8.6 months	report-specific missingness retained	Barbi 2025, p. 3–4, Results, Table 1
Barbi 2025	>10 mg	35	median 67.5 years (IQR 63.6–73.0)	female population	advanced or recurrent endometrial cancer	not systematically reported	ECOG 0/1/2/3: 17/10/7/1; nutrition: not systematically reported	prior lines 0/1/2/>=3: 0/22/11/2	overall median 8.6 months	report-specific missingness retained	Barbi 2025, p. 3–4, Results, Table 1
Dzienny 2026	<=10 mg	28	median 68.5 years	female population	advanced or recurrent endometrial cancer	not systematically reported	ECOG 0–1: 23/28; ECOG >=2: 5/28; nutrition: not systematically reported	median 2 prior regimens; high-grade 27/28	not reported by arm	report-specific missingness retained	Dzienny 2026, p. 2–3, Results, Table 1; Supplementary Tables S1-S2
Dzienny 2026	>10 mg	31	median 68.0 years	female population	advanced or recurrent endometrial cancer	not systematically reported	ECOG 0–1: 28/31; ECOG >=2: 3/31; nutrition: not systematically reported	median 2 prior regimens; high-grade 19/31	not reported by arm	report-specific missingness retained	Dzienny 2026, p. 2–3, Results, Table 1; Supplementary Tables S1-S2
Nagase 2025	overall	105	median 64 years (range 30–84)	female population	advanced or recurrent endometrial cancer	not systematically reported	ECOG 0 n85; ECOG 1 n20; nutrition: not systematically reported	all had prior platinum-containing chemotherapy	median 11.3 months	report-specific missingness retained	Nagase 2025, p. 2–3, Results, Methods; Figure 3; Table 3
Tochigi 2024	overall	15	median 66 years (range 37–78)	female population	advanced or recurrent endometrial cancer	not systematically reported	not reported; nutrition: not systematically reported	1/2/3 prior regimens: 9/4/2	median 340 days	report-specific missingness retained	Tochigi 2024, p. 3, Results, Methods; Figure 5
Takahashi 2026	overall	26	mean 61.2 years (SD 10.6) in nonendometrioid and 54.5 years (SD	female population	recurrent endometrial cancer; 15 endometrioid and 11 nonendometrioid	not systematically reported	ECOG 0 n=23; ECOG 1 n=3; nutrition not systematically reported	all progressed after platinum-containing chemotherapy; prior immunotherapy	histology-stratified median follow-up 6.4 and 13.6 months; overall summary not reported	ORR RDI comparison uses 18 patients with measurable	Takahashi 2026, p. PDF p.3 (journal 706), Results,

Study	Group or exposure level	N	Age	Sex	Disease stage or severity	Key comorbidity	Baseline outcome value	Treatment background	Follow-up	Missing baseline data	Source report and location
			10.8) in endometrioid groups; overall mean not reported					excluded		lesions; PFS model uses all 26	Table 1

Note: Not reported indicates report-specific missingness; values were not inferred from other cohorts.

Supplementary Table C9. Sensitivity and exploratory analyses

Outcome	Analysis type	Change from main analysis	Studies	Effect measure	Pooled estimate	95% CI	Heterogeneity	Interpretation constraint	Conclusion impact	Role label
Progression-free survival	exploratory	Separate prespecified analysis role; the main-analysis population/model was not replaced	2	HR	0.716	0.005 to 100.981	I^2=77.8%; tau²=0.244	The 95% CI spans more than four orders of magnitude; the pooled estimate is quantitatively uninformative.	Retained to document the prespecified exploratory calculation; interpret study-level results and limitations.	exploratory
Disease control	supplementary	Separate prespecified analysis role; the main-analysis population/model was not replaced	5	RR	0.959	0.826 to 1.113	I^2=0.0%; tau²=0.000	Non-significant or null-crossing intervals are not equivalence/noninferiority evidence.	Supports interpretation of robustness/uncertainty only; does not establish equivalence, non-inferiority, or preserved efficacy	supplementary
Grade 3 or higher adverse events	exploratory	Separate prespecified analysis role; the main-analysis population/model was not replaced	2	RR	1.128	0.468 to 2.719	I^2=0.0%; tau²=0.000	Non-significant or null-crossing intervals are not equivalence/noninferiority evidence.	Supports interpretation of robustness/uncertainty only; does not establish equivalence, non-inferiority, or preserved efficacy	exploratory
Treatment interruption	supplementary	Separate prespecified analysis role; the main-analysis population/model was not replaced	2	RR	0.894	0.155 to 5.157	I^2=14.4%; tau²=0.013	Non-significant or null-crossing intervals are not equivalence/noninferiority evidence.	Supports interpretation of robustness/uncertainty only; does not establish equivalence, non-inferiority, or preserved efficacy	supplementary
Toxicity-related discontinuation	exploratory	Separate prespecified analysis role; the main-analysis population/model was not replaced	2	RR	0.778	0.201 to 3.014	I^2=0.0%; tau²=0.000	Non-significant or null-crossing intervals are not equivalence/noninferiority evidence.	Supports interpretation of robustness/uncertainty only; does not establish equivalence, non-inferiority, or preserved efficacy	exploratory
Objective response	sensitivity	Total treated-arm denominator replaces the radiologically evaluable ORR denominator	5	RR	0.969	0.702 to 1.339	I^2=11.4%; tau²=0.012	Non-significant or null-crossing intervals are not equivalence/noninferiority evidence. Denominator sensitivity point estimate is close to main ORR, but this does not prove preserved efficacy.	Supports interpretation of robustness/uncertainty only; does not establish equivalence, non-inferiority, or preserved efficacy	sensitivity
Toxicity-related discontinuation	sensitivity	Dose threshold is ≤10 mg versus >10 mg and the outcome is any-toxicity discontinuation	2	RR	1.045	0.657 to 1.661	I^2=0.0%; tau²=0.000	Non-significant or null-crossing intervals are not equivalence/noninferiority evidence. Direct-only route excluding the derived Dzienny 2026 aggregate is k=1 and is not pooled.	Supports interpretation of robustness/uncertainty only; does not establish equivalence, non-inferiority, or preserved efficacy	sensitivity

Note: Sensitivity and exploratory estimates do not replace the prespecified main analyses and do not establish equivalence, noninferiority, or preserved efficacy.

Supplementary Table C10. Leave-one-out and influence analyses

Outcome	Omitted study	Studies remaining	Pooled estimate	95% CI	Heterogeneity	Change from main estimate	Influence flag	Interpretation
ORR	How 2021	4	0.945	0.627 to 1.425	I^2=13.3%; tau^2=0.012	Leave-one-out estimate 0.945 versus main random-effects estimate 0.971	Not formally classified; no prespecified threshold	Diagnostic only; no automatic exclusion
ORR	Zammarrelli 2023	4	0.984	0.617 to 1.570	I^2=23.3%; tau^2=0.024	Leave-one-out estimate 0.984 versus main random-effects estimate 0.971	Not formally classified; no prespecified threshold	Diagnostic only; no automatic exclusion
ORR	Ta 2025	4	1.124	0.664 to 1.903	I^2=0.7%; tau^2=0.001	Leave-one-out estimate 1.124 versus main random-effects estimate 0.971	Not formally classified; no prespecified threshold	Diagnostic only; no automatic exclusion
ORR	Corr 2025	4	0.881	0.690 to 1.125	I^2=0.0%; tau^2=0.000	Leave-one-out estimate 0.881 versus main random-effects estimate 0.971	Not formally classified; no prespecified threshold	Diagnostic only; no automatic exclusion
ORR	Boku 2026	4	1.041	0.683 to 1.588	I^2=25.9%; tau^2=0.031	Leave-one-out estimate 1.041 versus main random-effects estimate 0.971	Not formally classified; no prespecified threshold	Diagnostic only; no automatic exclusion
DCR	How 2021	4	0.939	0.813 to 1.084	I^2=0.0%; tau^2=0.000	Leave-one-out estimate 0.939 versus main random-effects estimate 0.959	Not formally classified; no prespecified threshold	Diagnostic only; no automatic exclusion
DCR	Zammarrelli 2023	4	0.953	0.775 to 1.172	I^2=0.0%; tau^2=0.000	Leave-one-out estimate 0.953 versus main random-effects estimate 0.959	Not formally classified; no prespecified threshold	Diagnostic only; no automatic exclusion
DCR	Ta 2025	4	0.996	0.790 to 1.255	I^2=0.0%; tau^2=0.000	Leave-one-out estimate 0.996 versus main random-effects estimate 0.959	Not formally classified; no prespecified threshold	Diagnostic only; no automatic exclusion
DCR	Corr 2025	4	0.936	0.779 to 1.125	I^2=0.0%; tau^2=0.000	Leave-one-out estimate 0.936 versus main random-effects estimate 0.959	Not formally classified; no prespecified threshold	Diagnostic only; no automatic exclusion
DCR	Boku 2026	4	0.989	0.809 to 1.209	I^2=0.0%; tau^2=0.000	Leave-one-out estimate 0.989 versus main random-effects estimate 0.959	Not formally classified; no prespecified threshold	Diagnostic only; no automatic exclusion
REDUCTION	How 2021	2	0.714	0.028 to 18.364	I^2=63.9%; tau^2=0.087	Leave-one-out estimate 0.714 versus main random-effects estimate 0.657	Not formally classified; no prespecified threshold	Diagnostic only; no automatic exclusion
REDUCTION	Zammarrelli 2023	2	0.582	0.508 to 0.666	I^2=0.0%; tau^2=0.000	Leave-one-out estimate 0.582 versus main random-effects estimate 0.657	Not formally classified; no prespecified threshold	Diagnostic only; no automatic exclusion
REDUCTION	Corr 2025	2	0.755	0.032 to 17.627	I^2=41.7%; tau^2=0.051	Leave-one-out estimate 0.755 versus main random-effects estimate 0.657	Not formally classified; no prespecified threshold	Diagnostic only; no automatic exclusion

Note: Leave-one-out estimates are diagnostic and were not used to select the primary model.

Supplementary Table C11. Risk of bias due to missing evidence

Synthesis	Outcome	k	ROB-ME judgment	Likely direction
Progression-free survival, reported unadjusted HR (exploratory)	PFS	2	Some concerns	unpredictable
Objective response, primary starting-dose analysis	ORR	5	Some concerns	unpredictable
Disease control, supplementary starting-dose analysis	DCR	5	Some concerns	unpredictable
Grade 3 or higher adverse events (exploratory)	AE3	2	Some concerns	unpredictable
Subsequent lenvatinib dose reduction, primary starting-dose analysis	REDUCTION	3	Some concerns	unpredictable
Treatment interruption, supplementary starting-dose analysis	INTERRUPTION	2	Some concerns	unpredictable
Permanent discontinuation due to combination-treatment toxicity (exploratory)	DISC TOX	2	Some concerns	unpredictable
Objective response using total-arm denominators (sensitivity)	ORR	5	Some concerns	unpredictable
Any-toxicity discontinuation, ≤10 mg versus >10 mg (sensitivity)	DISC TOX	2	Some concerns	unpredictable

Note: ROB-ME was applied to the nine quantitative syntheses; narrative-only evidence did not trigger a formal ROB-ME judgment.

Supplementary Table C12. Summary of findings

Outcome	Anticipated absolute effect or baseline risk	Relative effect	Participants	Studies	Certainty	Reasons for certainty rating	Interpretation
Progression-free survival (exploratory)	Not calculated; baseline risk and follow-up windows were heterogeneous	HR 0.72 (0.005 to 101.0)	401	2	Very low	Serious ROBINS-I risk and imprecision; observational association only. Null-crossing intervals are not equivalence or noninferiority evidence.	Quantitatively uninformative: the 95% CI spans more than four orders of magnitude. Interpret the study-level estimates and their limitations; no equivalence or noninferiority conclusion.
Objective response (main)	Not calculated; baseline risk and follow-up windows were heterogeneous	RR 0.97 (0.70 to 1.34)	683	5	Very low	Serious ROBINS-I risk and imprecision; observational association only. Null-crossing intervals are not equivalence or noninferiority evidence.	Observational association only; the interval does not establish equivalence or noninferiority.
Disease control (supplementary)	Not calculated; baseline risk and follow-up windows were heterogeneous	RR 0.96 (0.83 to 1.11)	683	5	Very low	Serious ROBINS-I risk and imprecision; observational association only. Null-crossing intervals are not equivalence or noninferiority evidence.	Observational association only; the interval does not establish equivalence or noninferiority.
Grade 3 or higher adverse events (exploratory)	Not calculated; baseline risk and follow-up windows were heterogeneous	RR 1.13 (0.47 to 2.72)	231	2	Very low	Serious ROBINS-I risk and imprecision; observational association only. Null-crossing intervals are not equivalence or noninferiority evidence.	Observational association only; the interval does not establish equivalence or noninferiority.
Subsequent lenvatinib dose reduction (main)	Not calculated; baseline risk and follow-up windows were heterogeneous	RR 0.66 (0.34 to 1.27)	301	3	Very low	Serious ROBINS-I risk and imprecision; observational association only. Null-crossing intervals are not equivalence or noninferiority evidence.	Observational association only; the interval does not establish equivalence or noninferiority.
Treatment interruption (supplementary)	Not calculated; baseline risk and follow-up windows were heterogeneous	RR 0.89 (0.16 to 5.16)	113	2	Very low	Serious ROBINS-I risk and imprecision; observational association only. Null-crossing intervals are not equivalence or noninferiority evidence.	Observational association only; the interval does not establish equivalence or noninferiority.
Combination-treatment toxicity discontinuation (exploratory)	Not calculated; baseline risk and follow-up windows were heterogeneous	RR 0.78 (0.20 to 3.01)	258	2	Very low	Serious ROBINS-I risk and imprecision; observational association only. Null-crossing intervals are not equivalence or noninferiority evidence.	Observational association only; the interval does not establish equivalence or noninferiority.
Objective response, total-arm denominator (sensitivity)	Not calculated; baseline risk and follow-up windows were heterogeneous	RR 0.97 (0.70 to 1.34)	692	5	Very low	Serious ROBINS-I risk and imprecision; observational association only. Null-crossing intervals are not equivalence or noninferiority evidence.	Observational association only; the interval does not establish equivalence or noninferiority.
Any-toxicity discontinuation, 10-mg threshold (sensitivity)	Not calculated; baseline risk and follow-up windows were heterogeneous	RR 1.04 (0.66 to 1.66)	151	2	Very low	Serious ROBINS-I risk and imprecision; observational association only. Null-crossing intervals are not equivalence or noninferiority evidence.	Observational association only; the interval does not establish equivalence or noninferiority.

Note: Certainty ratings follow the prespecified observational-evidence route; every pooled comparison was rated very low certainty.

Supplementary Table C13. Eligibility status of selected clinically relevant reports

Report	DOI	Review stage and material checked	Eligibility status	Reason and role in this review
Shang et al. (2024)	10.1136/ijgc-2024-005331	Title and abstract	Not included in the evidence set	Single-arm pilot of lenvatinib 8 or 12 mg plus anti-PD-1 therapy in a mixed ovarian and endometrial cancer population; no 20-mg lenvatinib comparator and the checkpoint inhibitor was not restricted to pembrolizumab. Contextual literature only.
Makker et al. (2023)	10.1200/JCO.22.02152	Title and abstract; pivotal report checked for clinical context	Not included in the evidence set; retained as background	Randomized comparison of lenvatinib 20 mg plus pembrolizumab versus chemotherapy; no reduced starting-dose or prespecified relative-dose-intensity contrast.
Chiba et al. (2024)	10.1093/jjco/hyad192	Title and abstract; second-pass screening review; targeted full report and supplementary-material review	Not included in the evidence set	No baseline starting-dose comparison. Supplementary Table S2 reported a 6-week relative-dose-intensity analysis at a 64% cutoff, which is incompatible with the prespecified first-8-week estimand. Clinical and molecular context only.
Kim et al. (2022)	10.1016/j.ygyno.2022.02.020	Title and abstract; second-pass screening review	Not included in the evidence set	Single-cohort overall outcomes and treatment-emergent dose reduction or interruption; no baseline starting-dose comparison. Contextual literature only.

Note: These four reports were excluded at title/abstract screening. Subsequent targeted source checks are identified separately below and are not part of the full-text exclusion count in Supplementary Table C6.
